# Unveiling the Origin of Regioselectivity in Iron(III)-Promoted Friedel–Crafts Synthesis of Cannabinoids: Theoretical Studies and Synthetic Applications

**DOI:** 10.1021/acs.joc.6c01142

**Published:** 2026-07-23

**Authors:** Mohamed El Hllafi, Iván Cheng-Sánchez, Irama Fuentes-Pino, Antonio Sánchez-Ruiz, Juan Manuel López-Romero, Juan Suárez, Mohamed Amin El Amrani, Francisco Sarabia

**Affiliations:** † Department of Organic Chemistry, Faculty of Sciences, 16752 University of Málaga, Campus de Teatinos s/n, Málaga 29071, Spain; ‡ Laboratory of Applied Chemistry, Microbiology, and Biotechnology, Faculty of Sciences, 531751 Abdelmalek Essaadi University, Tetouan 93000, Morocco; § Biomedical Research Institute of Málaga − IBIMA Plataforma BIONAND, Málaga 29590, Spain; ∥ Department of Human Anatomy, Legal Medicine and History of Science, University of Málaga, Málaga 29071, Spain

## Abstract

Phytocannabinoids, particularly cannabidiol (CBD), are important targets in pharmaceutical and industrial development due to their promising biological properties. However, access to CBD and related cannabinoids remains limited by their low abundance and variable composition in plant sources, whereas chemical synthesis of CBD is often hampered by the poor regioselectivity and modest yields associated with conventional Friedel–Crafts approaches. We recently disclosed an FeCl_3_·6H_2_O-catalyzed reaction that enables diastereoselective and highly regioselective preparation of (−)-CBD under mild and scalable conditions. Here, we report experimental and computational mechanistic studies that provide insight into the origin of this regioselectivity and support the involvement of a catalytically relevant iron­(III)-olivetol complex, which reacts regioselectively with the carbocation generated from the terpenic unit. We also define the scope and limitations of the method and demonstrate its synthetic utility through the efficient formal synthesis of bioactive cannabinoid derivatives, including HU-210 and ajulemic acid.

## Graphical abstract

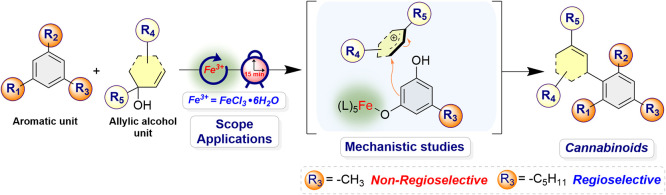

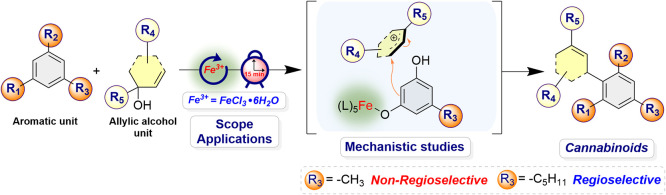

## Introduction

Phytocannabinoids are natural constituents found primarily in plants of the Cannabaceae family, particularly *Cannabis sativa*.^1^ More than 500 compounds have been identified from this species, including over 125 phytocannabinoids, together with terpenes, flavonoids, phenolics, and alkaloids.^2^ Among the major and best-characterized phytocannabinoids are cannabidiol (CBD, **1**), Δ^9^-tetrahydrocannabinol (Δ^9^-THC, **2**), cannabigerol (CBG, **3**), cannabinodiol (CBND, **4**), cannabinol (CBN, **5**), cannabichromene (CBC, **6**), and cannabielsoin (CBE, **7**) (Figure 1A).^3^ By contrast, many so-called rare cannabinoids occur only in trace amounts in the plant and are therefore difficult to access directly from natural sources, despite their interesting biological properties. These include Δ^8^-THC (**8**), cannabidivarin (CBDV, **9**), Δ^9^-tetrahydrocannabiorcinol (Δ^9^-THCO, **10**), Δ^9^-tetrahydrocannabivarin (Δ^9^-THCV, **11**), Δ^9^-tetrahydrocannabutol (Δ^9^-THCB, **12**) and Δ^9^-tetrahydrocannabiforol, (Δ^9^-THCP, **13**) (Figure 1B).^4^ Structurally, compounds **10**–**13** differ from Δ^9^-THC primarily in the length of the alkyl side chain, underscoring the importance of this pharmacophore in modulating biological activity. Their low natural abundance has made chemical synthesis an attractive, and often necessary, strategy for securing reliable access to them in useful quantities for biological evaluation.^5^

**Figure 1:**
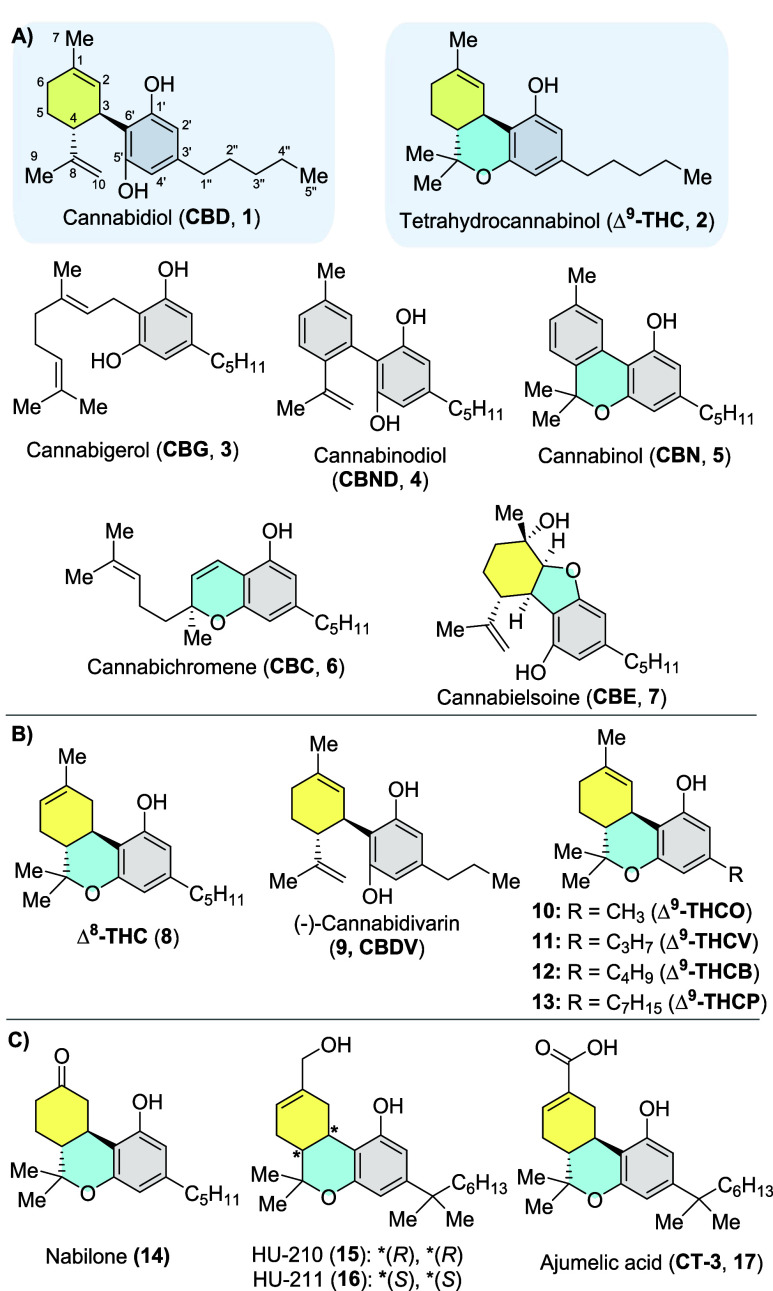
A) Molecular structures of the major natural cannabinoids from *C. sativa*. B) Molecular structures of rare cannabinoids. C) Selected synthetic cannabinoids.

In addition, synthetic cannabinoids and cannabinoid-inspired analogues have emerged as potential valuable therapeutic agents and pharmacological tools.^6^ Representative examples include Nabilone (**14**), HU-210 (**15**), HU-211 (**16**), and ajulemic acid (CT-3, **17**) (Figure 1C). Nabilone is an FDA-approved cannabinoid used as an antiemetic for chemotherapy-induced nausea and vomiting in patients who do not respond adequately to conventional therapy.^7^ HU-211 (dexanabinol, **16**),^8^ the nonpsychotropic unnatural (+)-enantiomer of HU-210 (**15**),^9^ has been investigated primarily for neuroprotective and anti-inflammatory properties. By contrast, HU-210 is a highly potent synthetic cannabinoid used as a pharmacological probe to study neurotransmitter release, immune regulation and synaptic function. Furthermore, ajulemic acid (CT-3, **17**), a synthetic analogue of Δ^9^-THC-11-oic acid, has been reported to exhibit analgesic and anti-inflammatory properties with reduced psychotropic effects relative to the parent compound.^9,10^ Within this broader landscape, (−)-CBD occupies a particularly prominent position because of its broad spectrum of biological activity and the absence of the psychotropic effects commonly associated with Δ^9^-THC (**2**).^11^ Interest in CBD has been driven by both its established clinical utility and its broader therapeutic potential. CBD is approved for the treatment of specific refractory epilepsies,^12^ while its therapeutic potential in neurodegenerative pathologies, such as Parkinson’s and Alzheimer’s diseases, remains under active preclinical investigation.^13^ Furthermore, preliminary studies suggest promising antitumoral^14^ and antibacterial activities for this scaffold.^15^ Consequently, CBD has become an important target in medicinal chemistry, chemical biology, and industrial process development, driven by the growing demand for its commercialization.^16^ However, direct isolation of CBD and minor cannabinoids from plant material presents significant practical limitations, including the variable composition of the natural source, the low abundance of many target compounds, and the need for laborious purification from structurally related metabolites.^17^

Consequently, stereoselective chemical synthesis has become a central approach for the rapid and reliable preparation of natural cannabinoids and structurally modified analogues.^18^ Several synthetic strategies have been developed for asymmetric CBD synthesis,^19^ including: a) Friedel–Crafts alkylation of olivetol or related derivatives with a suitable chiral limonene-type monoterpene,^19a–19d^ b) cross-coupling approaches involving organometallic derivatives of olivetol,^19e^ and c) construction of the cyclohexene ring through stereocontrolled cyclization strategies, including ring closing metathesis from chiral precursors^20^ or enantioselective Diels–Alder cycloaddition.^21^ Although the latter two approaches have enabled elegant syntheses of CBD, their multistep nature or operational complexity, including the need to handle unstable organometallic intermediates, can limit their practicality and broader application. Consequently, Friedel–Crafts alkylation of olivetol remains the most widely used strategy for CBD asymmetric synthesis. This approach originated in the pioneering work of Petrzilka and coworkers,^22^ who employed *p*-(+)-*cis,trans*-mentha-2,8-dien-1-ol as the terpenoid component in the presence of oxalic acid. Since then, numerous groups have explored this strategy using Brønsted acids,^23^ Lewis acids,^24^ as well as catalyst-free conditions based on hexafluoro-2-propanol.^25^ However, the direct use of olivetol as the nucleophilic partner in this reaction continues to suffer from poor regioselectivity leading to formation of several side products, including the cyclized psychotropic derivative Δ^9^-THC (**2**), the abnormal isomer of CBD (abn-CBD, **20**) and the dialkylated product (bis-CBD, **21**) (Scheme 1A). As a consequence, CBD is often obtained in suboptimal yield, and product isolation from these complex mixtures remains challenging, particularly for large-scale and practical applications. Accordingly, efficient, regioselective, enantioselective, and scalable syntheses of (−)-CBD that proceed under operationally simple conditions remain highly desirable, both for access to the natural product itself and for the preparation of structurally diverse cannabinoid analogues of medicinal and industrial interest. Recently, we reported a new synthesis of (−)-CBD that meets these criteria and employs FeCl_3_·6H_2_O as the catalyst.^26^ Herein, we provide mechanistic studies on this protocol, define its scope and limitations, and demonstrate its utility in the formal preparation of related cannabinoids, such as HU-210 (**15**) and ajulemic acid (CT-3, **17**).

**Scheme 1:**
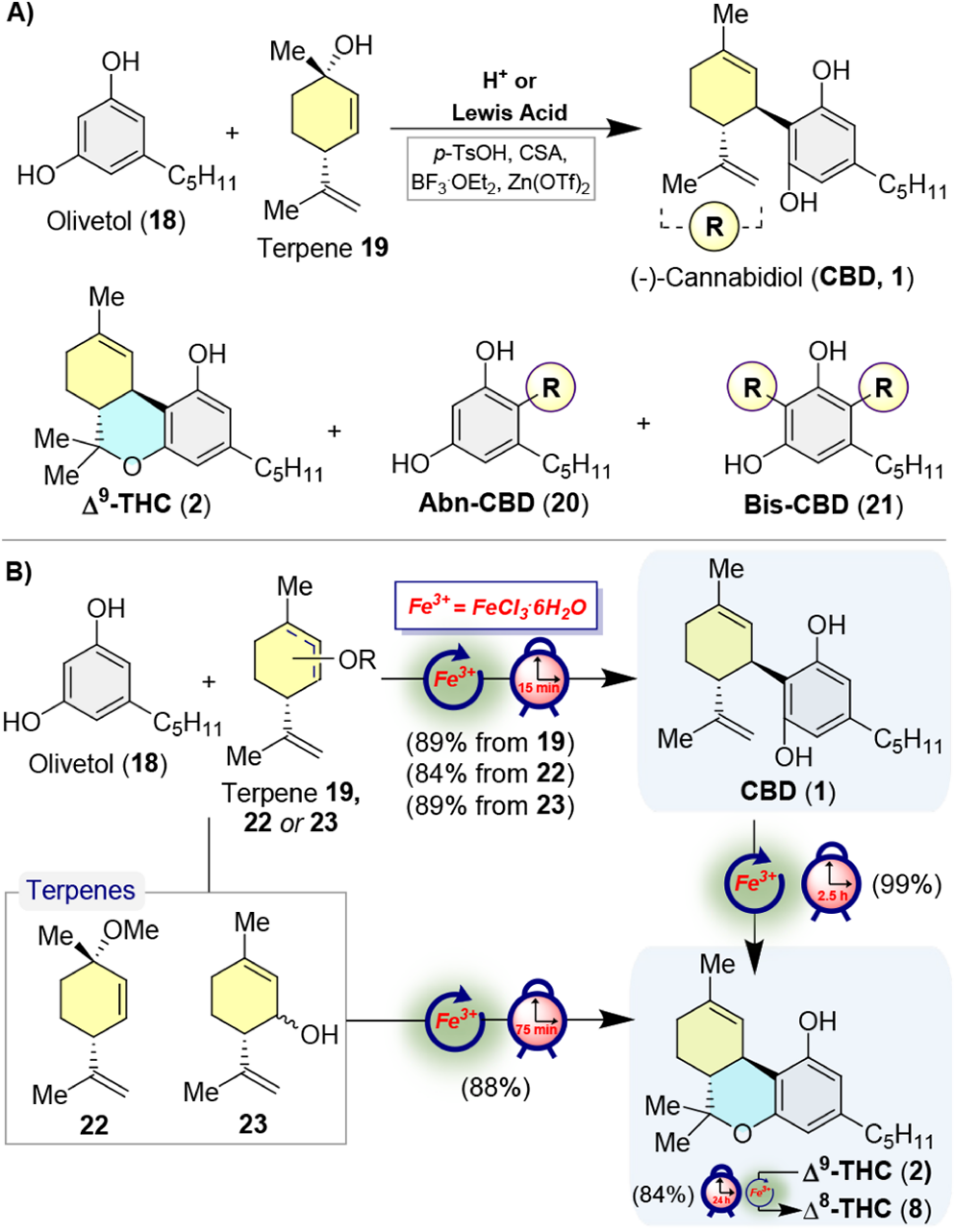
A) General Synthesis of (−)-CBD by Friedel–Crafts Alkylation of Olivetol (**18**) and the Major Side Products; B) FeCl_3_·6H_2_O-Catalyzed Synthesis of CBD by Friedel–Crafts Alkylation of Olivetol (**18**), Developed by Sarabia and Co-Workers^a^

a Standard reaction conditions for the synthesis of (−)-CBD (**1**): olivetol (**18**) (1.0 equiv), terpene (1.0 equiv), FeCl_3_·6H_2_O (10 mol %), CH_2_Cl_2_, 25 °C, 15 min. Reaction conditions for the synthesis of Δ^9^-THC (**2**) from olivetol (**18**): terpene (1.0 equiv), FeCl_3_·6H_2_O (0.6 equiv), CH_2_Cl_2_, 25 °C, 75 min. Reaction conditions for the synthesis of Δ^9^-THC (**2**) from (−)-CBD (**1**): FeCl_3_·6H_2_O (0.6 equiv), CH_2_Cl_2_, 25 °C, 2.5 h. Reaction conditions for the synthesis of Δ^8^-THC (**8**) from olivetol (**18**): terpene (1.0 equiv), FeCl_3_·6H_2_O (1.2 equiv), CH_2_Cl_2_, 25 °C, 24 h. Reaction conditions for the synthesis of Δ^8^-THC (**8**) from Δ^9^-THC (**2**): FeCl_3_·6H_2_O (0.6 equiv), CH_2_Cl_2_, 25 °C, 24 h.

## Results and Discussion

In the course of our efforts to develop an optimal synthesis of (−)-CBD through the Friedel–Crafts coupling of olivetol (**18**) with a suitable chiral terpenic unit (**19**, **22** or **23**), we found after extensive optimization that FeCl_3_·6H_2_O was uniquely effective among the Brønsted and Lewis acids examined, affording CBD in 89% yield under mild reaction conditions (23 °C and CH_2_Cl_2_ as solvent) and in only 15 min (Scheme 1B).^26^ More importantly, only trace amounts of the bis-alkylated derivative (bis-CBD, **21**), Δ^9^-THC (**2**), and abn-CBD (**20**) were detected. The FeCl_3_·6H_2_O-catalyzed reaction also proceeded in comparable yield in alternative solvents, such as *tert*-butyl methyl ether, and could be readily scaled to the multigram level. Interestingly, other iron salts, such as Fe­(acac)_3_ and Fe­(NO_3_)_3_, were ineffective for CBD formation: Fe­(acac)_3_ led primarily to recovery of the starting materials, whereas Fe­(NO_3_)_3_ afforded a high proportion of abn-CBD (**20**). Likewise, anhydrous FeCl_3_ gave a markedly inferior outcome, furnishing CBD in only 30% yield together with abn-CBD (16%), bis-CBD (10%), and Δ^9^-THC (19%).^26^ Importantly, the developed method could be tuned for the selective synthesis of both Δ^9^- and Δ^8^-THC, (Scheme 1, part B). Thus, reaction of olivetol (**18**) with terpene **19** in the presence of 0.6 equiv of FeCl_3_·6H_2_O afforded Δ^9^-THC (**2**) as the major product in 88% isolated yield after 75 min. Consistent with this observation, CBD (**1**) was completely converted into Δ^9^-THC (**2**) after 2.5 h under the same loading of the iron salt (Scheme 1, part B). Similarly, treatment of Δ^9^-THC (**2**) with 0.6 equiv of FeCl_3_·6H_2_O for 24 h led to formation of Δ^8^-THC (**8**) in 84% yield. Alternatively, Δ^8^-THC (**8**) could be obtained directly from olivetol (**18**) and terpene **19** in 64% yield upon treatment with a stoichiometric amount of FeCl_3_·6H_2_O for 24 h.^26^

Collectively, these findings, and in particular the detection of only trace amounts of abn-CBD (**20**) after 15 min under the standard conditions, suggested that both Fe^3+^ and its associated ligands may play important roles in the reaction mechanism. To elucidate the course of the FeCl_3_·6H_2_O-catalyzed reaction, we first carried out a series of mechanistic experiments. Specifically, we considered whether the reaction might proceed through initial formation of the kinetic abn-CBD isomer (**20**), followed by equilibration to the thermodynamically favored normal CBD isomer (**1**), in a similar manner to the mechanism proposed by Porco Jr. et al.^27^ for the synthesis of 8,9-dihydrocannabidiol (H_2_CBD, **25**), via an intramolecular retro-Friedel–Crafts/Friedel–Crafts sequence (Scheme 2A). To test this hypothesis, we subjected pure synthetic abn-CBD (**20**) to the action of FeCl_3_·6H_2_O (10 mol %) in CH_2_Cl_2_ at 23 °C for 15 min, thereby reproducing the optimized catalytic protocol. Under these conditions, however, no equilibration of abn-CBD (**20**) to the normal isomer was observed. Instead, ^1^H NMR analysis of the crude reaction mixture after 15 min showed formation of the cyclized product abn-Δ^9^-THC (**26**), together with unreacted abn-CBD (**20**). These results indicate that, under the FeCl_3_·6H_2_O-catalyzed conditions, CBD (**1**) is formed directly rather than through interconversion of the abnormal isomer. We next corroborated this conclusion by a kinetic experiment monitored by GC-MS. Specifically, the reaction of olivetol (**18**) and (+)-*p*-mentha-2,8-dien-1-ol (**19**) in the presence of FeCl_3_·6H_2_O (10 mol %) was monitored over time (see the SI for experimental details), and only trace amounts of abn-CBD (**20**) were detected at any stage of the reaction, with the normal isomer observed as the predominant product at all time points.

**Scheme 2:**
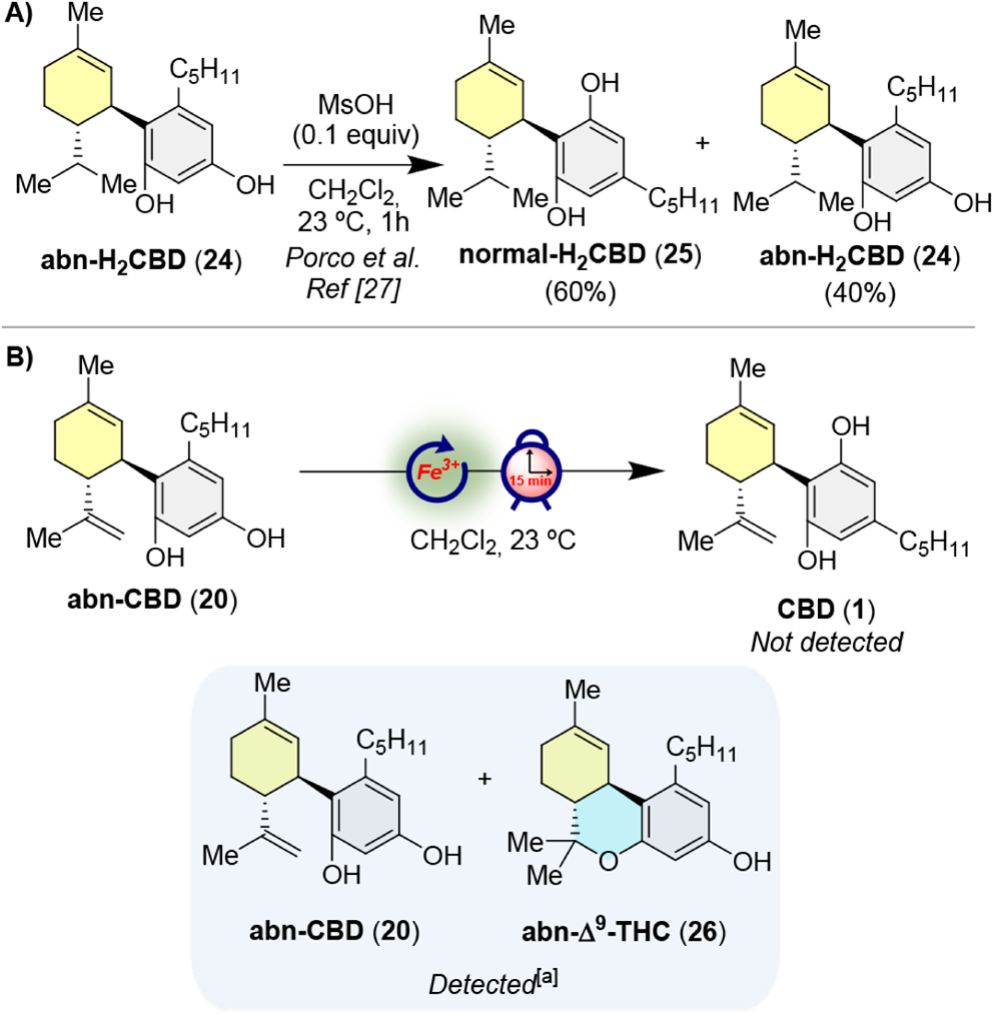
A) Equilibration of Abnormal to Normal H_2_CBD by MsOH; B) Equilibration of Abnormal to Normal CBD by FeCl_3_·6H_2_O^a^

a Determined by ^1^H NMR analysis of the crude mixture.

Having established that abn-CBD (**20**) is not generated in significant amounts during the reaction course, we next turned our attention to the role of the iron catalyst. As shown in our initial screening studies, alternative iron salts, including Fe­(acac)_3_ and Fe­(NO_3_)_3_, were ineffective for CBD formation, while anhydrous FeCl_3_ afforded poor yield and selectivity. These observations collectively indicate that FeCl_3_·6H_2_O plays a distinctive role in controlling the course of the reaction and does not merely function as a classical Lewis acid for activation of the terpenic alcohol. To probe whether iron forms a complex with olivetol (**18**), and whether such a species could be catalytically relevant, we recorded the UV–vis spectrum in CH_2_Cl_2_ of olivetol (**18**) and compared it with the spectra obtained in the presence of FeCl_3_·6H_2_O using different olivetol (**18**) and iron stoichiometries (Figure 2). Analysis of the UV–vis data revealed that mixtures of olivetol (**18**) (1.0, 2.0, or 3.0 equiv) and FeCl_3_·6H_2_O (1.0 equiv) displayed two new absorption bands in the 300–400 nm region relative to FeCl_3_·6H_2_O or olivetol (**18**) alone, consistent with formation of an iron-olivetol complex. Notably, the same bands were also observed under catalyst-loading conditions using olivetol (**18**) (1.0 equiv) and FeCl_3_·6H_2_O (0.1 equiv), indicating that the same iron-olivetol chromophore is generated under both stoichiometric and catalytic conditions and suggesting a predominant complex containing one olivetol ligand per iron center. These observations are consistent with our experimental protocol, in which olivetol (**18**) (1.0 equiv) and FeCl_3_·6H_2_O (10 mol %) are first dissolved in CH_2_Cl_2_, giving a light yellow solution prior to addition of the terpenic alcohol. Moreover, literature precedent also supports the formation of a complex between Fe^3+^ and olivetol (**18**), but in a 1:3 ratio, in 50%MeOH-water.^28^ To further probe the preferred stoichiometry of the complex in CH_2_Cl_2_, UV–vis studies were carried out using the method of continuous variation (Job plot).^29^ Analysis of the more intense and better-resolved absorption band at λ = 360 nm showed a maximum at an olivetol mole fraction of 0.495, consistent with an 1:1 stoichiometry for the iron-olivetol complex (see Supporting Information). With evidence suggesting that FeCl_3_·6H_2_O may act as a precatalyst, and that the catalytically active species is generated *in situ* through complexation with olivetol (**18**), we next performed a series of control experiments with modified arene partners to probe the role of the phenolic hydroxy groups of olivetol in the reaction (Figure 3). Thus, olivetol dimethyl ether was prepared and subjected to the standard reaction conditions with (+)-*p*-mentha-2,8-dien-1-ol (**19**). Under these reaction conditions, the expected product **27** was obtained in only 17% yield, with substantial recovery of the starting materials. This marked loss of reactivity is consistent with involvement of the phenolic OH groups of olivetol in Fe^3+^ coordination and supports the hypothesis of an iron-olivetol complex as the catalytically relevant species.

**Figure 2:**
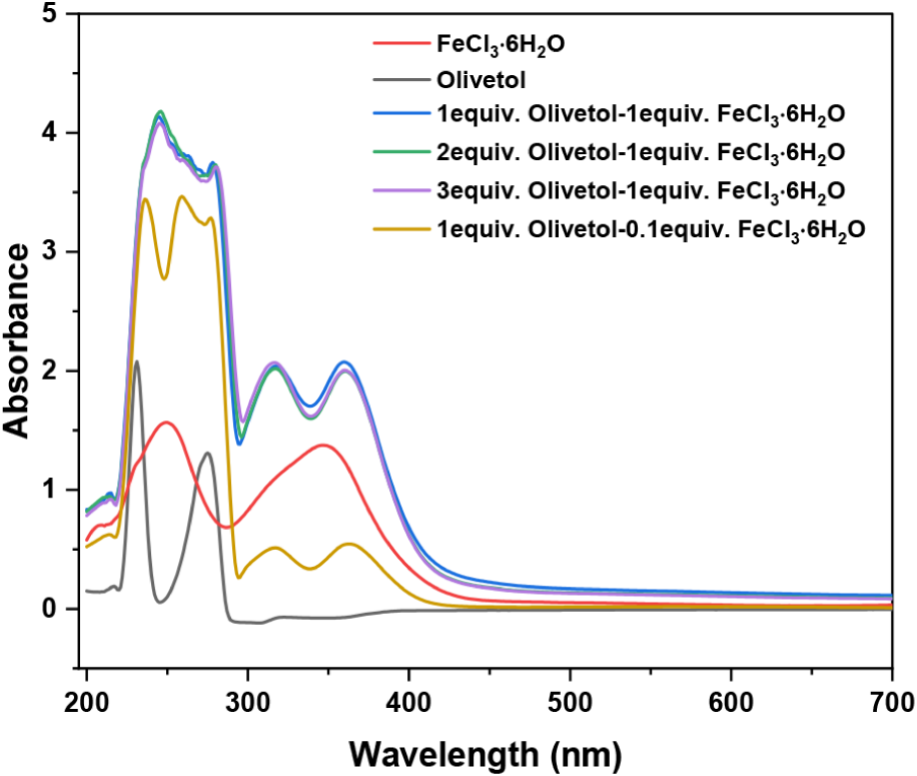
UV–vis spectra of FeCl_3_·6H_2_O, olivetol (**18**), and their mixtures at different stoichiometries, in CH_2_Cl_2_.

**Figure 3:**
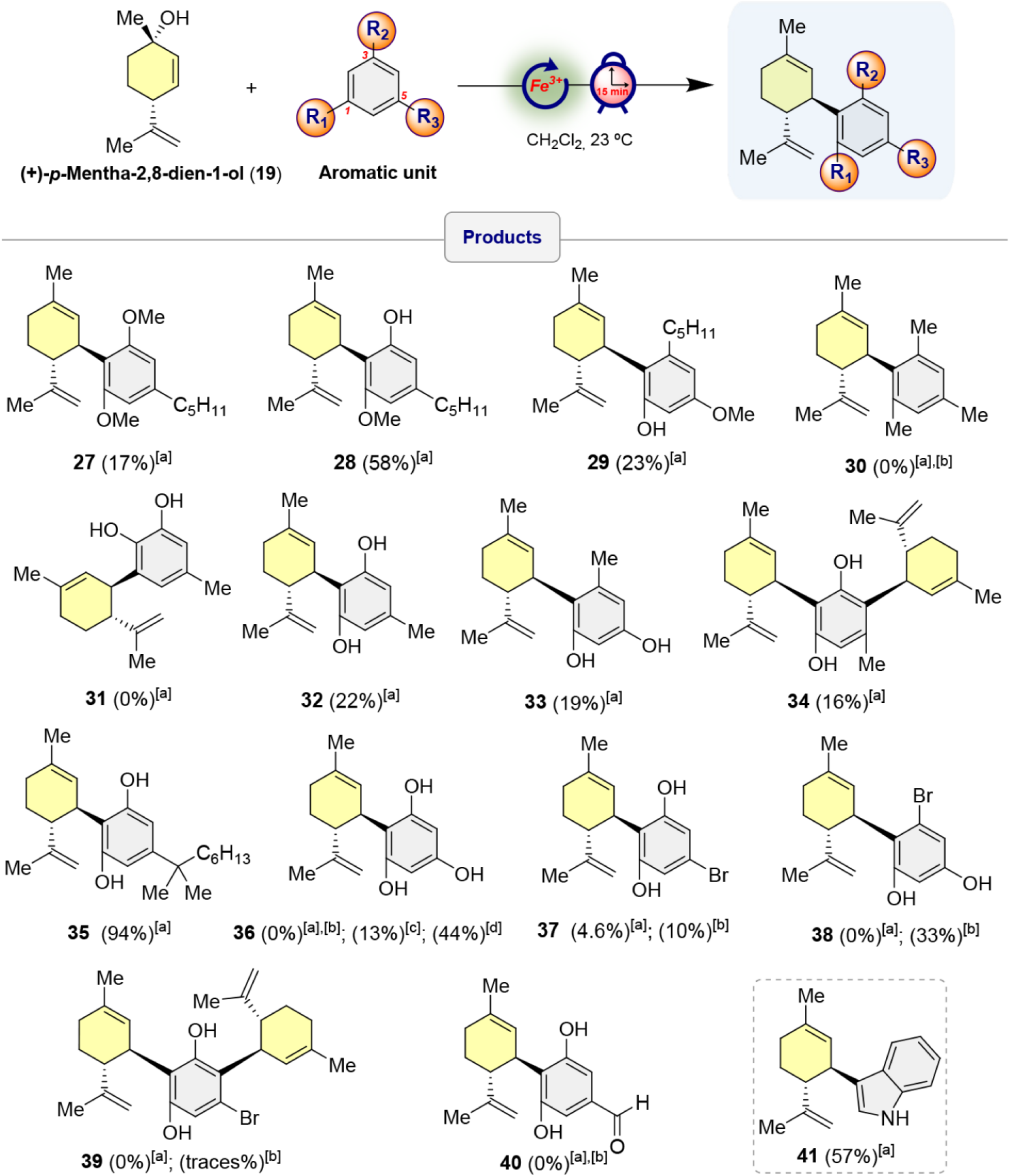
Substrate scope of the aromatic component in the FeCl_3_·6H_2_O-catalyzed Friedel–Crafts alkylation with with (+)-*p*-mentha-2,8-dien-1-ol (**19**) for mechanistic studies. Reaction conditions: (+)-*p*-mentha-2,8-dien-1-ol (**19**) (1.0 equiv), aromatic unit (1.0 equiv), FeCl_3_·6H_2_O (10 mol %), CH_2_Cl_2_, 23 °C. Isolated yields after column chromatography. ^[a]^Reaction time: 15 min. ^[b]^Reaction time: 24 h. ^[c]^Reaction time: 72 h, and THF was used as solvent. ^[d]^Reaction time: 15 min, and CH_3_CN was used as solvent.

We next examined olivetol monomethyl ether, which afforded the desired product **28** in 58% yield, together with the abnormal isomer (**29**, 23%) and recovered starting material. The improved outcome relative to the dimethyl ether **27**, yet diminished efficiency compared with olivetol itself (89%), further underscore the importance of a free phenolic OH group for productive Fe^3+^ mediated catalysis. Additional support was obtained from a control experiment using mesitylene as the arene component. Because mesitylene lacks a hydroxy group capable of coordinating iron, product **30** was not detected under the reaction conditions, as expected, even with longer reaction times. Finally, 4-methyl catechol was evaluated to probe the importance of the 1,3-dihydroxy substitution pattern of olivetol (**18**) in catalyst binding. In contrast to olivetol (**18**), the expected product **31** was not detected. The 1,2-dihydroxy motif of catechol likely chelates Fe^3+^ too strongly, thereby sequestering the metal and preventing productive catalysis required for formation of **31** (Figure 3).

Further insight into the reaction mechanism was obtained by examining the effect of steric bulk in the C5 alkyl substituent of the resorcinol partner. Thus, the reaction of orcinol, which bears a methyl group at the 5-position, afforded the desired product **32** in only 22% yield. In addition, the abnormal isomer (**33**) and the dialkylated product, **34**, were isolated in 19 and 16% yield, respectively, after column chromatography. The formation of substantial amounts of the abnormal and bis-alkylated products in this case may be attributed to the reduced steric demand at the 4- and 6-positions of orcinol. These results suggest that steric bulk at the 5-position of the aromatic unit is important for controlling the regioselectivity of the reaction.

Consistent with this interpretation, reaction of 5-(1,1-dimethylheptyl)­resorcinol, which bears a substantially bulkier substituent at C5, furnished the CBD-dimethylheptyl (DMH–CBD) derivative **35** as the sole product in 94% yield, confirming the importance of steric effects at this position. We were also interested in examining how the presence of additional heteroatom substituents at the C5 position of the resorcinol partner affected the reaction outcome. We first evaluated phloroglucinol, a highly electron-rich arene, but found it to be unreactive under the standard reaction conditions. This lack of reactivity was attributed primarily to its poor solubility in CH_2_Cl_2_. We therefore developed a modified protocol in which CH_2_Cl_2_ was replaced with THF and the reaction time was extended to 72 h. Under these conditions, the desired product **36** was obtained in a poor 13% yield, with recovery of the starting material. Further improvement was achieved by replacing THF with CH_3_CN, which raised the yield to 44%, suggesting that CH_3_CN may improve solubilization of the highly polar phloroglucinol substrate. This low reactivity suggests that, despite its electron-rich nature, phloroglucinol is not a particularly effective substrate under the present conditions, and the results are consistent with other examples in literature, in particular using CH_2_Cl_2_ or THF as solvent.^30^

Other heteroatom-substituted arene partners were also examined as potentially useful substrates for the preparation of CBD analogues. In particular, 5-bromo-1,3-benzenediol was investigated because the resulting products could serve as versatile intermediates for further derivatization, including the preparation of recently disclosed CBD-based PROTACs.^31^ Unfortunately, the normal isomer **37** was obtained in a poor 10% yield. By contrast, the abnormal isomer, compound **38**, was obtained in a 33% yield and traces of the bis-alkylated product were also detected. Notably, the preferential formation of the abnormal brominated isomer **38** may provide a useful entry to abnormal CBD analogues through subsequent functionalization at the bromine substituent, while also providing access to alternative linker attachment vectors for CBD-based PROTAC development.^31^ However, the FeCl_3_·6H_2_O-catalyzed reaction did not tolerate the less electron-rich arene 3,5-dihydroxybenzaldehyde, and the expected product **40** could not be detected even with prolonged reaction times. Finally, we examined the compatibility of the method with nonresorcinol arene equivalents, such as indole. Under the standard conditions, the reaction proceeded to furnish the desired coupling product **41** in a 57% yield. Importantly, compounds of this type are known intermediates in the synthesis of (+)-hapalindole H and (−)-12-epi-hapalindole U,^32^ a class of bioactive cyanobacterial alkaloids with antimicrobial, antifungal, and anticancer properties. Thus, this transformation provides an alternative synthetic entry to these natural products.

Different allylic alcohols as terpenoid fragment equivalents were evaluated next (Figure 4). These substrates were reacted with olivetol (**18**) under the action of FeCl_3_·6H_2_O in CH_2_Cl_2_ at 23 °C for either 15 min or 24 h. Linear allylic alcohol **42** and geraniol **43** proved unreactive under these conditions, and only starting materials were detected in the crude reaction mixtures by TLC and ^1^H NMR analysis. In the case of geraniol (**43**), which contains a methyl substituted alkene, only traces of cannabigerol (**CBG**, **3**) could be detected by ^1^H NMR of the crude mixture. Reaction of olivetol (**18**) with (1*R*)-(−)-myrtenol (**44**) was likewise unsuccessful, and only starting material was recovered. By contrast, when 2-cyclohexen-1-ol (**45**) was used as the allylic alcohol component, the desired product **50** was obtained in 19% yield after column chromatography, together with traces of the corresponding bis-alkylated product, indicating that secondary allylic alcohols are better tolerated under the reaction conditions. Reaction of 1-methylcyclohex-2-en-1-ol (**46**) afforded an inseparable mixture of **51** and bicyclic product **52** in a 2:1 ratio and 50% combined yield after column chromatography. Notably, the corresponding bicyclic product was not detected in the reaction with 2-cyclohexen-1-ol (**45**). This difference may be attributed to the greater electrophilicity of the alkene in 1-methylcyclohex-2-en-1-ol (**46**), as well as to the increased stability of the intermediate carbocation conferred by the methyl substituent. Finally, the more complex secondary allylic alcohol (*S*)-verbenol (**47**) was evaluated in reactions with both olivetol (**18**) and 5-(1,1-dimethylheptyl) resorcinol (DMH resorcinol). In these cases, inseparable mixtures of **53** and Δ^8^-THC (**8**), and of **54** and 1′,1′-DMH-Δ^8^-THC (**55**), in 1:3 and 1:2 ratios, respectively, were obtained after column chromatography. Formation of Δ^8^-THC (**8**) and 1′,1′-DMH-Δ^8^-THC (**55**) from (*S*)-verbenol (**47**) is consistent with literature precedent and has been attributed to a cascade process from the initially formed coupling products.^33^ Attempts to extend the reaction time did not improve the yield of the Δ^8^-isomer. Accordingly, under Fe^3+^ catalysis, access to this compound is more efficient from olivetol (**18**) or CBD (**1**) than from (*S*)-verbenol (**47**) (see Scheme 1B). Overall, these results indicate that primary allylic alcohols are not suitable substrates for this transformation, whereas secondary and tertiary allylic alcohols, particularly those bearing more electrophilic alkenes and capable of stabilizing the developing carbocation, are better tolerated.

**Figure 4:**
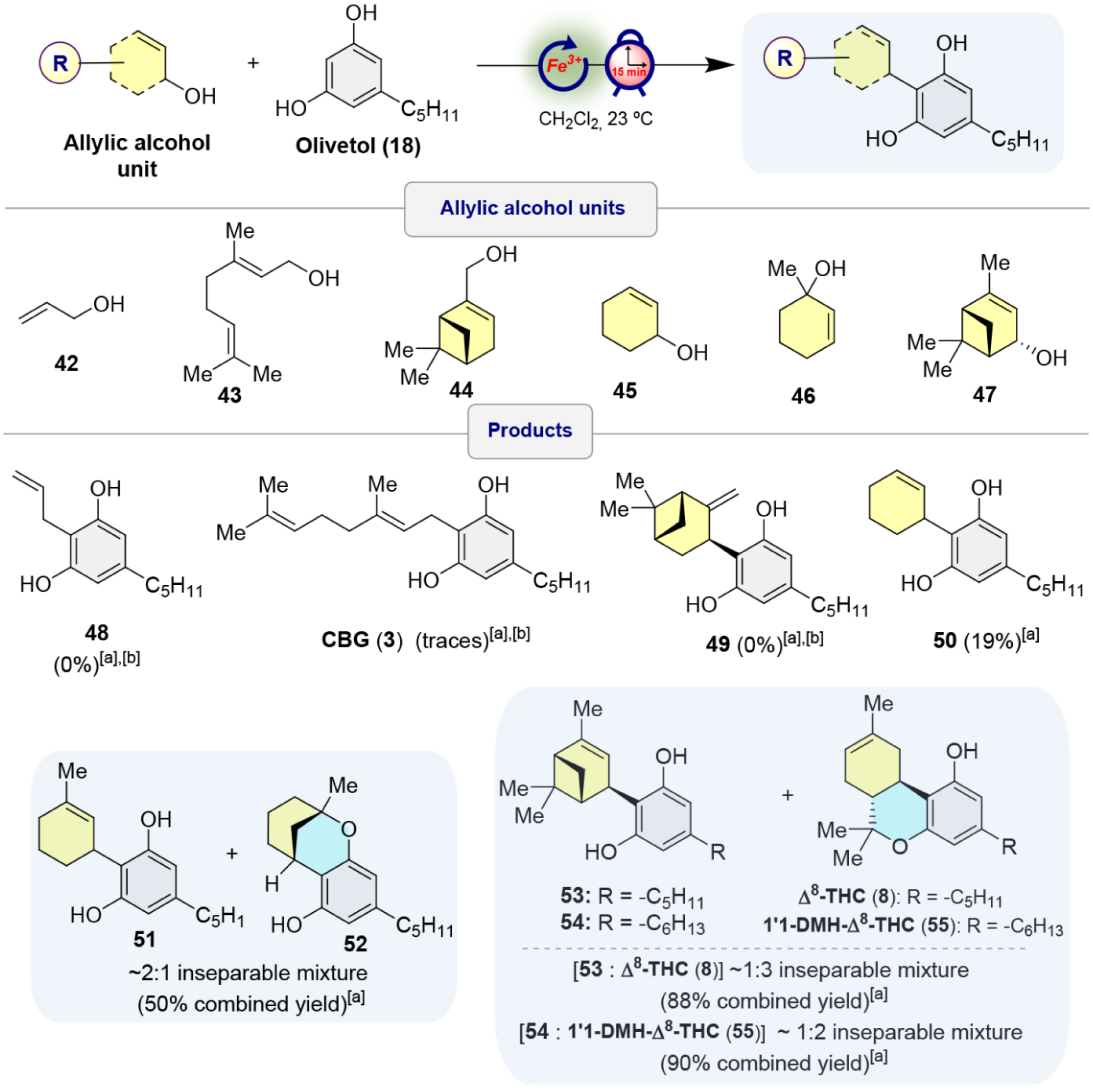
Substrate scope of the allylic alcohol component in the FeCl_3_·6H_2_O-catalyzed Friedel–Crafts alkylation of olivetol (**18**) for mechanistic studies. Reaction conditions: allylic alcohol unit (1.0 equiv), aromatic unit (1.0 equiv), FeCl_3_·6H_2_O (10 mol %), CH_2_Cl_2_, 23 °C, 15 min. ^[a]^Reaction time: 24 h. ^[b]^Reaction time: 72 h.

Based on the experimental studies described above, a plausible mechanism for the FeCl_3_·6H_2_O-catalyzed synthesis of CBD is proposed (Scheme 3). The catalytic cycle is initiated by formation of an iron-olivetol complex, occurring via ligand exchange with the loss of HCl and subsequent coordination of the phenolic oxygen atom of olivetol (**18**). This process leads to the generation of the triaquadichloroiron­(III)-olivetol species **I**. Intermediate **I** then promotes activation of the terpenic alcohol (or methoxy group), followed by loss of water in the case of terpene **19** and **23**, or methanol in the case of terpene **22**, to form stabilized tertiary carbocation **III**. Subsequent approach of the iron-olivetol complex **I** to carbocation **III** may direct the terpenic electrophile to the C2 position of olivetol (**18**), thereby affording intermediate **IV** regioselectively. This outcome is likely reinforced by the steric effect of the C5 alkyl chain, which appears to be a key factor in controlling the observed regioselectivity. Following C–C bond formation, triaquadichloroiron-CBD complex **V** is generated, together with release of HCl. The HCl formed in this step is sufficiently acidic to shift the equilibrium toward decoordination of the iron­(III)-CBD complex, thereby releasing CBD and generating hexaaquairon species **VI**. Subsequent complexation of **VI** with olivetol (**18**) regenerates complex **I**, thus completing the catalytic cycle (Scheme 3).

**Scheme 3:**
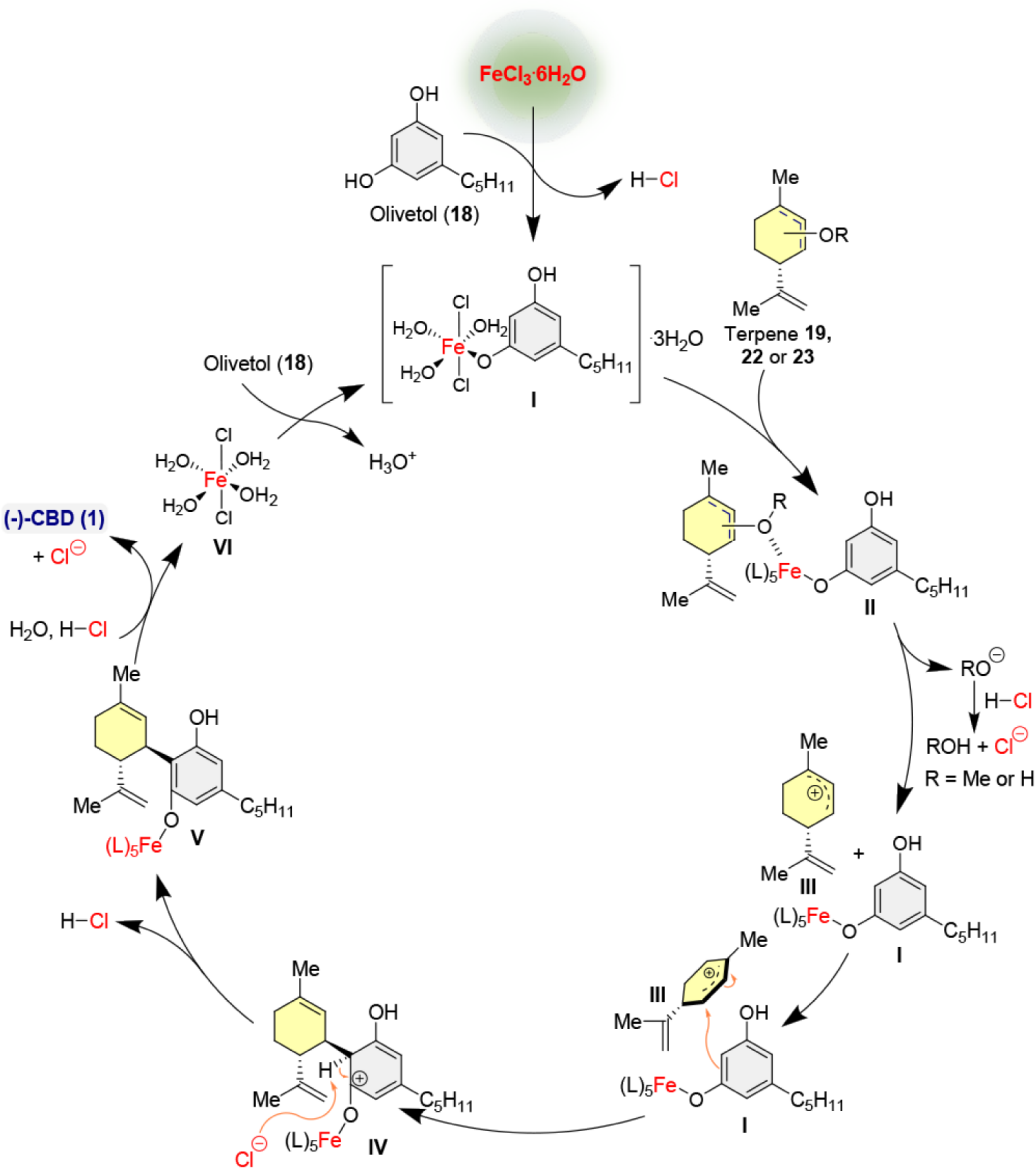
Proposed Mechanism for the FeCl_3_·6H_2_O Catalyzed Synthesis of (−)-CBD (**1**)

The mechanism of the addition of the iron complex **I** to the terpene cation **III** was also investigated using theoretical calculations with density functional theory. Initial exploration of the spin-state landscape confirmed that the high-spin sextet state (S = 5/2) was the exclusive reactive surface. Relaxed potential energy scans demonstrated that the quartet and doublet manifolds imposed severe thermodynamic penalties (+14.3 and +23.7 kcal/mol, respectively) without any accessible minimum energy crossing points (MECPs). Consequently, the entire mechanistic analysis was conducted on the ground-state sextet surface (see Supporting Information for full electronic structure discussion). To test the hypothesis that steric hindrance from the resorcinol C5 substituent acted as the primary stereocontrolling element in the regioselective synthesis of CBD (**1**), we evaluated the reaction trajectories which confirmed this premise, revealing that the C5 alkyl chain dictated the regioselectivity by imposing a steric penalty on the “abnormal” pathway. Comparing the competing transition states yielded an activation energy difference (ΔΔE^‡^) of 1.25 kcal/mol, favoring the formation of the normal CBD (**1**) (a detailed thermodynamic discussion justifying the use of electronic energies, ΔE^‡^, to avoid artificial entropic noise is provided in the Supporting Information). Structural analysis of these transition states explains this kinetic preference. In the “abnormal” pathway, the bulky pentyl chain is forced against the incoming cation, pushing their filled electron orbitals into direct spatial overlap. This caused the severe steric clashes (Pauli repulsion) that penalized the reaction (ΔE^‡^ = 1.87 kcal/mol). Conversely, in the “normal” pathway, the pentyl chain directed its bulk away from the reactive site effectively steering the reactants into a highly organized geometry. This forced alignment unlocked a stabilizing interaction: a noncovalent hydrogen bond between an iron-coordinated water ligand and the incoming isopropenyl group. Because the steric confinement of the pentyl chain preorganized the system to utilize this “OH anchor”, the normal trajectory proceeded through a more accessible barrier (ΔE^‡^ = 1.03 kcal/mol).

This kinetic divergence was directly reflected in the transition state geometries. The “abnormal” trajectory exhibited a larger imaginary frequency (−197 cm^–1^) than the “normal” trajectory (−142 cm^–1^), indicative of a more sterically congested transition state. As shown in Scheme 4A, the pentyl chain run parallel to the isopropenyl group in the abnormal structure, physically blocking the approach of the water ligand and preventing the formation of the stabilizing OH anchor, yielding the abn-CBD (**20**) product. By contrast, the normal trajectory navigated a broader, more relaxed saddle point stabilized by secondary noncovalent interactions, affording CBD (**1**) (Scheme 4B). To definitively confirm that the macroscopic bulk of the C5 substituent was the primary stereocontrolling element, the reaction was modeled using a sterically minimal methyl derivative complex **VII** (Scheme 4B). Truncating this aliphatic tail eliminated the steric bottleneck, resulting in a dramatic collapse of the activation energy difference (ΔΔE^‡^ = 0.17 kcal/mol). Because this negligible gap falls within the standard error margin for DFT calculations,^34,35^ the theoretical model predicted a complete loss of regioselectivity for the unhindered methyl derivative. This perfectly mirrored the experimental outcome, which yielded a nearly equimolar mixture of regioisomers (compound **32**, 22% normal and compound **33**, 19% abnormal).

**Scheme 4:**
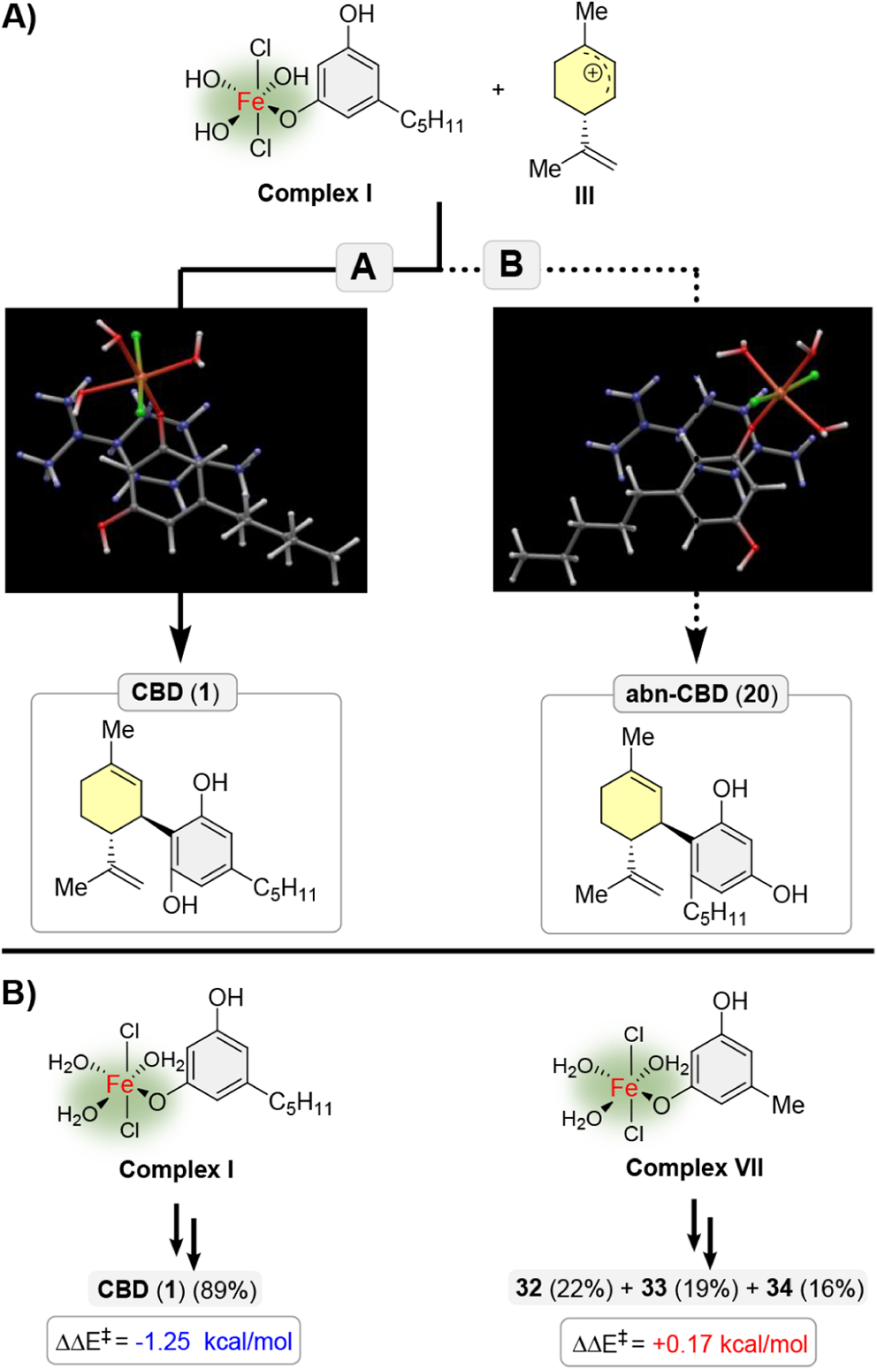
A) Structures of Transition State for Normal-CBD (**1**) (Pathway A) and abn-CBD (**20**) (Pathway B); B) Model Complex **VII** and Activation Energy Differences: ΔΔE^‡^ = ΔE^‡^Normal – ΔE^‡^Abnormal

To demonstrate the synthetic utility of the FeCl_3_·6H_2_O-catalyzed reaction, we set out to apply this protocol to the formal synthesis of the cannabinoid metabolites HU-210 (**15**)^9^ and ajulemic acid (CT-3, **17**)^9,10^ (Scheme 5). Thus, 5-(1,1-dimethylheptyl)­resorcinol (**56**) was coupled with (+)-*p*-mentha-2,8-dien-1-ol (**19**) under the standard reaction conditions to afford 1′,1′-DMH–CBD (**35**) in excellent yield (94%). From this intermediate, 1′,1′-DMH-Δ^9^-THC (**57**) was obtained in 90% yield by treatment with 0.6 equiv of FeCl_3_·6H_2_O for 2.5 h. Subsequent treatment of **57** with 0.6 equiv of FeCl_3_·6H_2_O for 24 h afforded 1′,1′-DMH-Δ^8^-THC (**55**) in 83% yield. The versatility of the method, through simple adjustment of iron salt amount and reaction time, was further demonstrated by the direct preparation of 1′,1′-DMH-Δ^8^-THC (**55**) from 5-(1,1-dimethylheptyl)­resorcinol (**56**) and (+)-*p*-mentha-2,8-dien-1-ol (**19**) upon conducting the reaction for 24 h in the presence of 1.2 equiv of FeCl_3_·6H_2_O. The conversion of compound **55** to HU-210 (**15**) and ajulemic acid (CT-3, **17**) is a procedure known in the literature,^9^ and thus, the present route constitutes a formal synthesis of both cannabinoids.

**Scheme 5:**
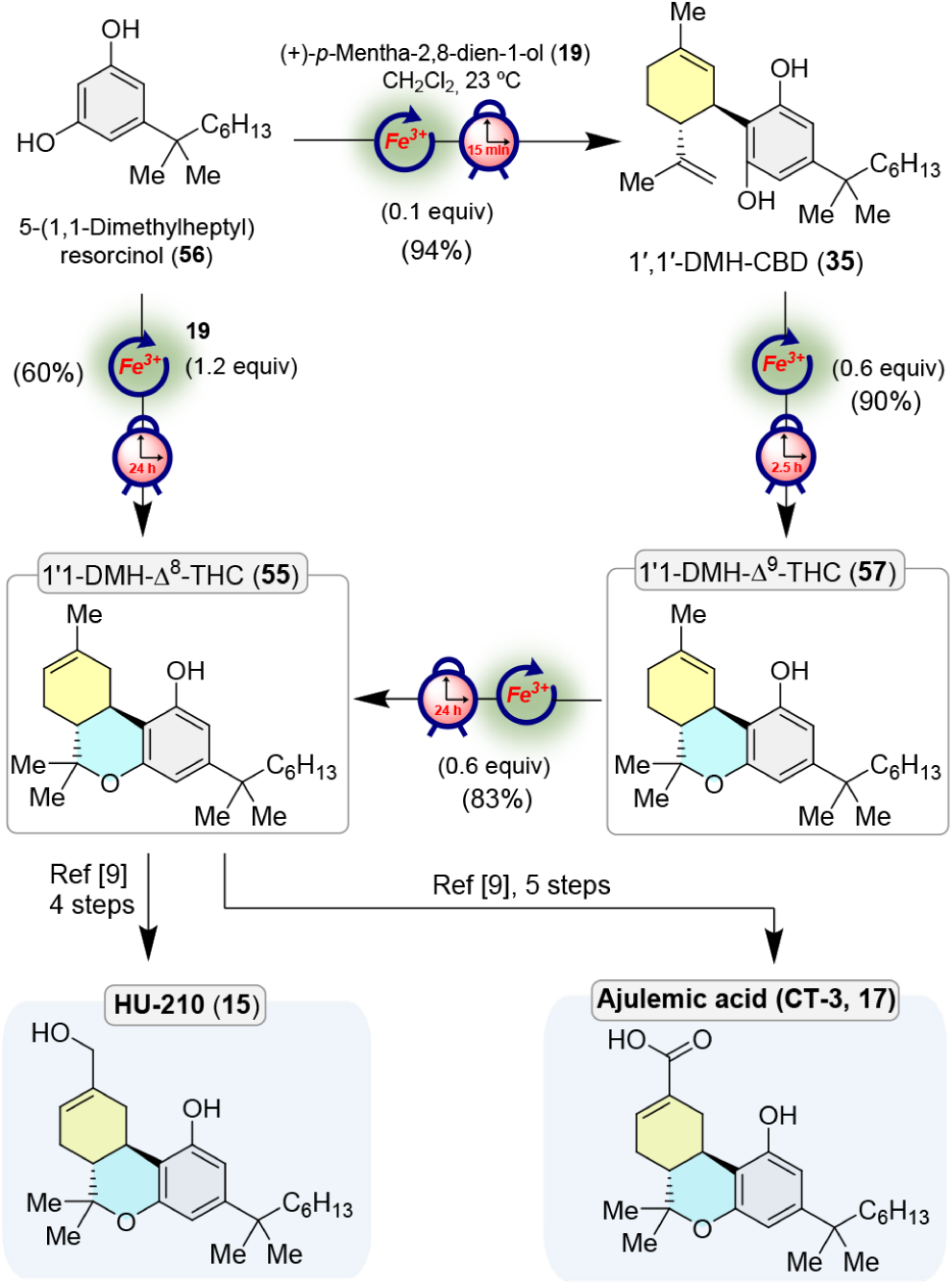
Synthetic Applications of the FeCl_3_·6H_2_O-Catalyzed Reaction to HU-210 (**15**) and Ajulemic Acid (CT3, **17**) Cannabinoids

## Conclusions

In conclusion, we have developed an efficient, scalable, and highly regioselective asymmetric synthesis of (−)-CBD (**1**) through an iron­(III)-promoted Friedel–Crafts reaction of olivetol (**18**) with terpenes **19**, **22** or **23**. Under the optimized conditions, (−)-CBD was formed as the major product, with only trace amounts of abn-CBD (**20**) and Δ^9^-THC (**2**) detected, thus overcoming the poor regioselectivity that has traditionally limited Friedel–Crafts alkylation of olivetol approaches. In addition, the course of the reaction could be tuned to provide either Δ^8^-THC (**8**) or Δ^9^-THC (**2**) by simple adjustment of the FeCl_3_·6H_2_O loading and reaction time, highlighting the versatility of the method. Mechanistic experiments established that normal CBD is generated directly under the reaction conditions rather than through interconversion of the abnormal isomer, and they support the involvement of an iron-olivetol complex as the catalytically relevant species. These studies further suggest that this intermediate, together with the steric effect imposed by the C5 substituent of olivetol, are responsible for the high regioselectivity observed. This mechanistic picture was further reinforced by DFT calculations, which supported the intermediacy of the iron-olivetol complex and showed that the C5 bulky chain was the primary stereocontrolling element under FeCl_3_·6H_2_O catalysis. Substrate scope studies revealed that the transformation is best suited to allylic alcohols capable of generating secondary or tertiary carbocations, whereas primary systems were not compatible under the optimized conditions. Finally, the synthetic utility of the protocol was demonstrated through the preparation of 1′,1′-DMH-Δ^8^-THC (**55**) and 1′,1′-DMH-Δ^9^-THC (**57**), key intermediates in the formal synthesis of HU-210 (**15**) and ajulemic acid (CT-3, **17**). In summary, these results positioned FeCl_3_·6H_2_O as a uniquely effective promoter for the selective preparation of (−)-CBD and provide new mechanistic insight into the origin of its regioselectivity.

## Supplementary Material



## Data Availability

The data underlying this study are available in the published article and its Supporting Information.

## Notes

Materials and general techniques; equilibration, kinetic and UV–vis experiments; computational studies; synthetic procedures and characterization data; and NMR spectra of compounds (PDF)
