# Supplementary material for: Unveiling the Origin of Regioselectivity in Iron(III)-Promoted Friedel–Crafts Synthesis of Cannabinoids: Theoretical Studies and Synthetic Applications
Source: J Org Chem. 2026 Jul 23;91(31):10679–89. doi: 10.1021/acs.joc.6c01142 (PMC13459411; doi:10.1021/acs.joc.6c01142)
Supplement: Supplementary file 1 [file jo6c01142_si_001.pdf]

Unveiling the Origin of Regioselectivity in Iron(III)-Promoted Friedel–Crafts Synthesis of Cannabinoids: Theoretical Studies and Synthetic Applications

Mohamed El Hllafi,[a],[b] Iván Cheng-Sánchez,*[a] Irama Fuentes-Pino,[a] Antonio Sánchez-Ruiz,[a] Juan Manuel López-Romero[a], Juan Suárez,[c],[d] Mohamed Amin El Amrani[b] and Francisco Sarabia*[a]

[a] Department of Organic Chemistry, Faculty of Sciences, University of Málaga, Campus de Teatinos s/n 29071-Málaga, Spain

[b] Laboratory of Applied Chemistry, Microbiology, and Biotechnology, Faculty of Sciences, Abdelmalek Essaadi University, Tetouan 93000, Morocco.

[c] Biomedical Research Institute of Málaga – IBIMA Plataforma BIONAND, 29590 Málaga, Spain

[d] Department of Human Anatomy, Legal Medicine and History of Science, University of Málaga, 29071 Málaga, Spain

Supporting Information

Table of Contents

1. Materials and General Techniques S2
2. General Synthetic Procedures S2
3. Equilibration Experiment of Abnormal to Normal CBD S3
4. Kinetic Experiment S4
5. UV-vis Experiment and Job Plot Analysis S20
6. Computational Studies S21
7. Experimental Procedures and Compound Characterization S57
8. ^{1}H, $^{13}C\{^{1}H\}$ NMR Spectra S68
9. References S90

1. Material and General Techniques

Characterization of compounds

^1H and $^{13}C\{^1H\}$NMR spectra were recorded on a Bruker DPX-400 or 500 MHz instrument calibrated using residual undeuterated solvent as an internal reference. Chemical shifts are reported in ppm with the resonance resulting from incomplete deuteration of the solvent as the internal standard ($^{13}CDCl_3$: 7.26 ppm, s and 77.0 ppm, t; $^{13}CD_3OD$: 4.87 ppm,s, 3.31 ppm, quin and 49.1 ppm, sep; 13C_2D_6OS: 2.49 ppm, quin and 39.52 ppm, sep). Data are reported as follows: chemical shift δ/ppm (multiplicity, coupling constants *J* (Hz) and integration (^1H only)).The following abbreviations were used to explain the multiplicities: s = singlet; d = doublet; t = triplet; q = quartet; quin = quintet; b =broad; m = multiplet or combination thereof. ^{13}C signals are singles, unless otherwise stated. High resolution mass spectrometry (HRMS) was performed on a H-ESI and APCI mass spectrometer in positive mode and using an ion trap (Orbitrap) as the mass analyzer type. HRMS signals are reported to 4 decimal places and are within ± 5 ppm of theoretical values. Specific optical rotations were corded on a Perkin-Elmer 241 polarimeter with a sodium halogen lamp (λ = 589 nm) and a cell path length of 100 mm (c given in g/100 mL). CG-MS analysis was performed on a PerkinElmer CG-MS (Claurus SQ8C) system with the Clarus 690 GC, with Electron Impact (EI) and Chemical Impact (CI) ionization and equipped with a 255 L/sec turbomolecular pump and Clarifi™ detector and quadrupole analyzer. The UV-vis experiment was recorder on a Biochrom Libra S22 UV/Visible spectrophotometer, 190–1100 nm, a bandwidth < 3 nm., with a xenon lamp.

Synthetic and purification methods

Reactions were carried out under air, unless otherwise stated. All solutions used in workup procedures were saturated unless otherwise noted. All reagents were purchased at highest commercial quality and used without further purification unless otherwise stated. Solvents were purchased in the best quality available. All reactions were monitored by thin-layer chromatography (TLC) using 0.25 mm silica gel plates (60F-254) using UV light (254 nm) as visualizing agent and acidic ceric ammonium molybdate/phosphomolybdic acid or potassium permanganate solutions and heat as developing agents. Flash column chromatography (FCC) was performed using silica gel (60 Å, particle size 230–400 mesh) under air pressure. All solvents used for chromatographic purifications were purchased in the best quality available.

2. General Synthetic Procedures

General procedure for the synthesis of CBD and derivatives

Olivetol (**18**) or the corresponding olivetol unit (1.0 equiv) and $FeCl_3{\cdot}6H_2O$ (0.1 equiv) were dissolved in CH_2Cl_2 (0.01 M) under ultrasound at 23 ºC for 5 min. After this time, the ultrasound bath was removed, and a solution of (+)-*p*-mentha-2,8-dien-1-ol (**19**) or the corresponding terpenic unit (1.0 equiv) in CH_2Cl_2 (0.02 M) was then added dropwise via a dropping funnel, under magnetic stirring. After complete addition, the reaction mixture was stirred at 23 ºC for 15 min. After this time, the crude reaction mixture was subsequently quenched with a saturated aqueous $NaHCO_3$ solution, extracted with Et_2O three times, and the combined organic layers were washed with brine, dried over anhydrous $MgSO_4$, filtered and concentrated under reduced pressure. The crude mixture was purified by column chromatography (silica gel, hexanes → 10% Et_2O in hexanes) to obtain the corresponding CBD (**1**) or CBD derivative.

Gram scale synthesis

The scalability of the general procedure was previously demonstrated on gram scale for the synthesis of (−)-CBD.[1]

3. Equilibration Experiment of Abnormal to Normal CBD

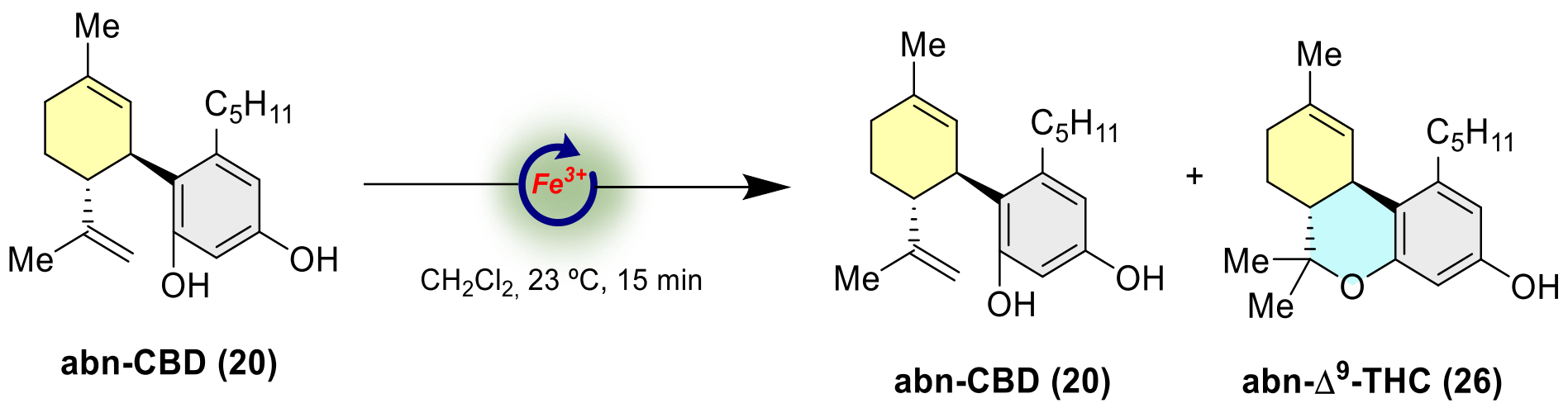

Figure S1. Equilibration experiment of abnormal to normal CBD determined by ^{1}H NMR analysis of the crude mixture.

Abn-CBD (**20**)[1] (7.2 mg, 0.022 mmol, 1.0 equiv) and $FeCl_3 \cdot 6H_2O$ (0.61 mg, 0.002 mmol, 0.1 equiv) were dissolved in CH_2Cl_2 (6 mL) and the resulting mixture was stirred at 23 ºC for 15 min. After this time, the reaction mixture was quenched with a saturated aqueous $NaHCO_3$ solution, extracted with CH_2Cl_2 three times, and the combined organic layers were washed with brine, dried over anhydrous $MgSO_4$, filtered and concentrated under reduced pressure to afford 6.3 mg of a pale yellow crude mixture which was analysed by ^{1}H NMR and was assigned as a mixture of **abn-CBD** (**20**) and **abn-Δ⁹-THC** (**26**) (Figure S2), based on comparison with the ^{1}H NMR spectrum of previously synthesized **abn-CBD** (**20**)[1] (Figure S3) and reported data for **abn-Δ⁹-THC** (**26**).[2]

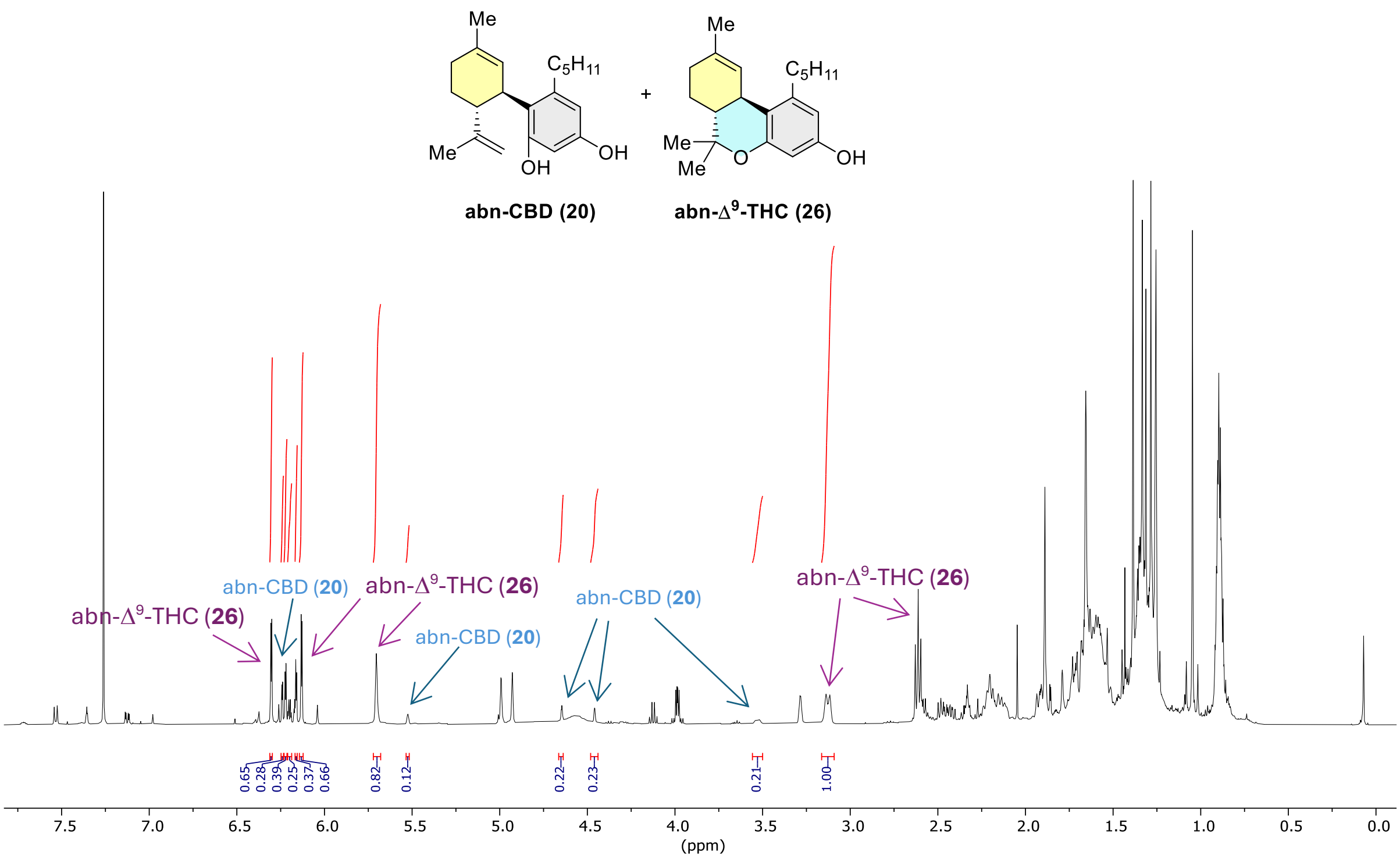

Figure S2. ^{1}H NMR spectra of the crude mixture obtained from the equilibration experiment.

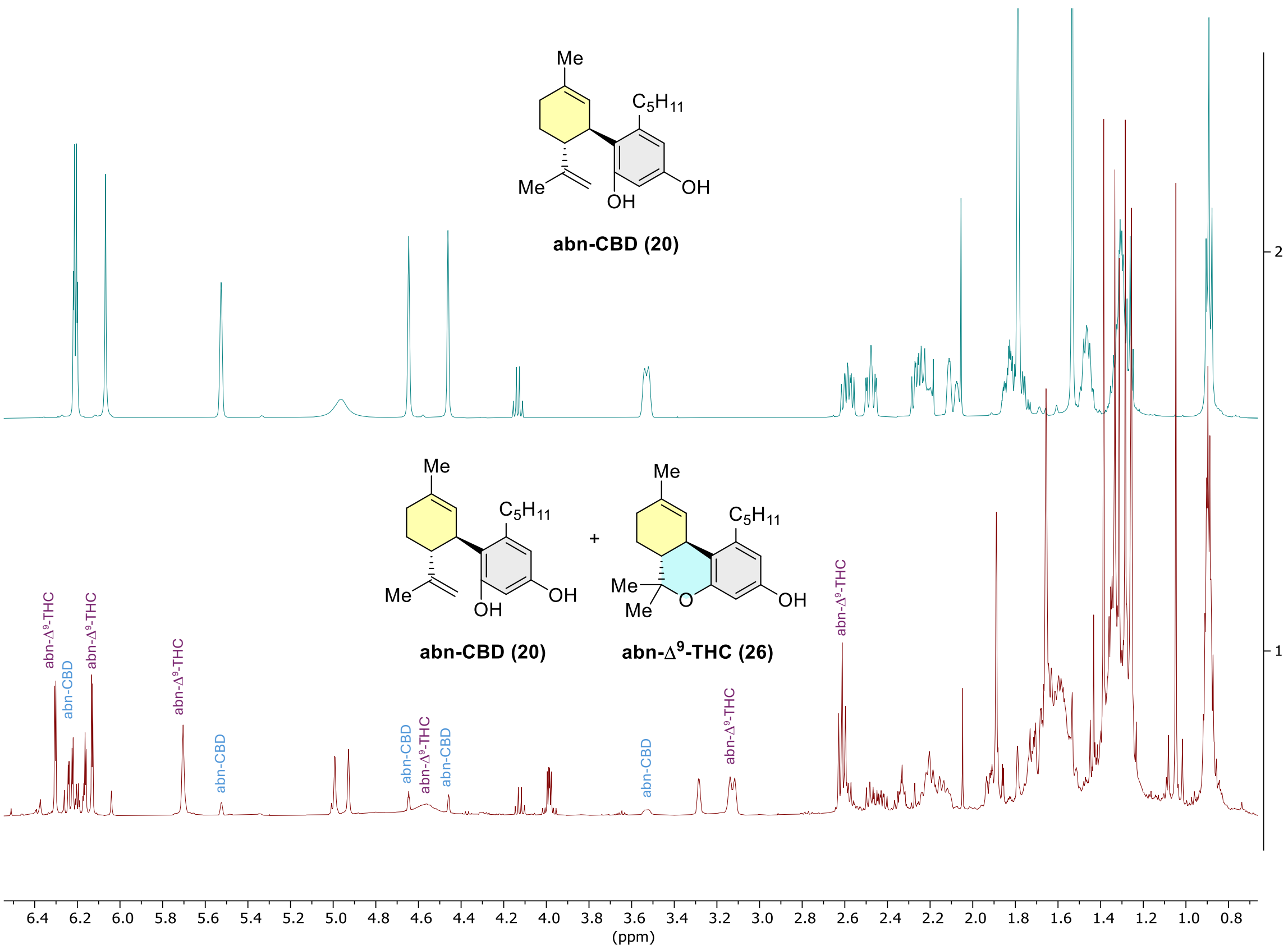

Figure S3. ^{1}H NMR stacked spectra of abn-CBD (**20**) (top) and the crude mixture obtained from the equilibration experiment (bottom).

4. Kinetic Experiment

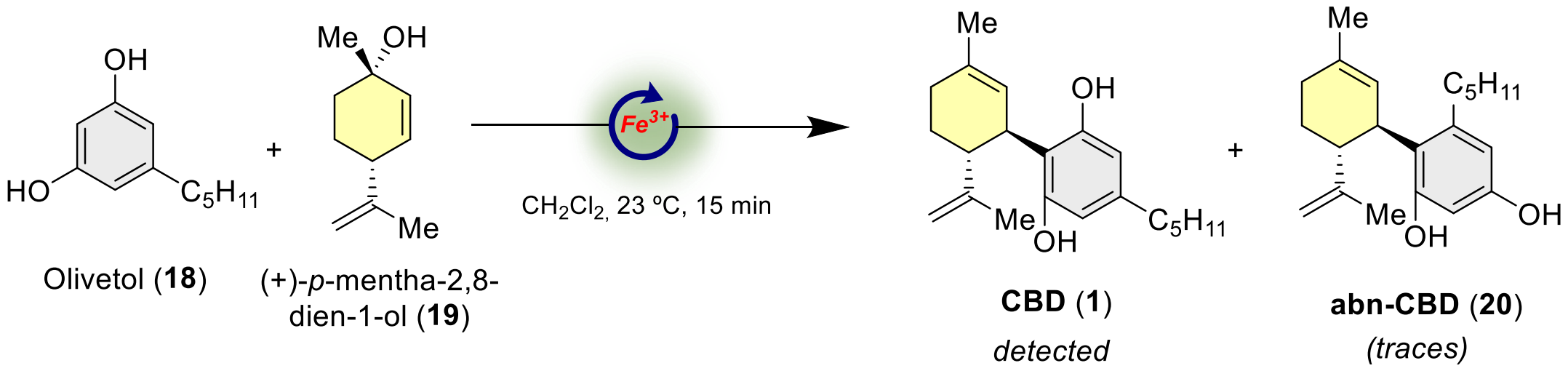

Figure S4. Kinetic experiment. The formation of CBD was monitored by CG-MS analysis.

Olivetol (**18**) (55.6 mg, 0.309 mmol, 1.0 equiv) and (+)-*p*-mentha-2,8-dien-1-ol (**19**) (45.0 mg, 0.309 mmol, 1.0 equiv) were reacted according to the general procedure for the synthesis of CBD. Aliquots of the reaction mixture were analyzed by GC-MS to monitor the reaction progress. During the first 5 minutes of the reaction, 200 μL aliquot of the reaction mixture were taken every 30 s. The iron catalyst from each aliquot was immediately quenched with 50 μL of an aqueous EDTA solution (0.5 M) to ensure the reaction was stopped. Subsequently, 800 μL of EtOAc was added and the mixture was shaken. Then, 800 μL of the organic phase was transferred into a GC-MS vial for analysis. After the first 5 minutes of the reaction, 200 μL aliquot of the reaction mixture were taken every 3 min and treated using the same quenching and extraction procedure prior to GC-MS analysis. CG-MS analysis was performed on a PerkinElmer CG-MS (Clarus SQ8C) system with the Clarus 690 GC, with Electron Impact (EI) and Chemical Impact (CI) ionization

and equipped with a 255 L/sec turbomolecular pump and ClarifiTM detector and quadrupole analyzer. The CG Method was set-up as follows: experiment time of 29.93 min; delay time 0.00 min; run time 29.93 min; injection volume 1 μL; Sampling rate of 1.56250 pts/s; for the oven temperature programme, the initial temperature was 80ºC for 5.00 min with a ramp of 10.0 ºC/min to 250 ºC, hold for 7.93 min. The MS Method was set-up including a solvent delay of 2 min, and MS scan time of 1.00 to 25.00 min, and a Mass from 50.00 to 800.00 EI+. For reference and to facilitate analysis of the GC-MS kinetic experiment, samples of pure (−)-CBD (**1**) and abn-CBD (**20**) were analyzed previously, giving retention times of 23.46 and 23.26 min, respectively. It should be noted that the peak at ca. 24.62 min arises from conversion of CBD to Δ^9-THC during GC-MS analysis, as previously reported in the literature.[3]

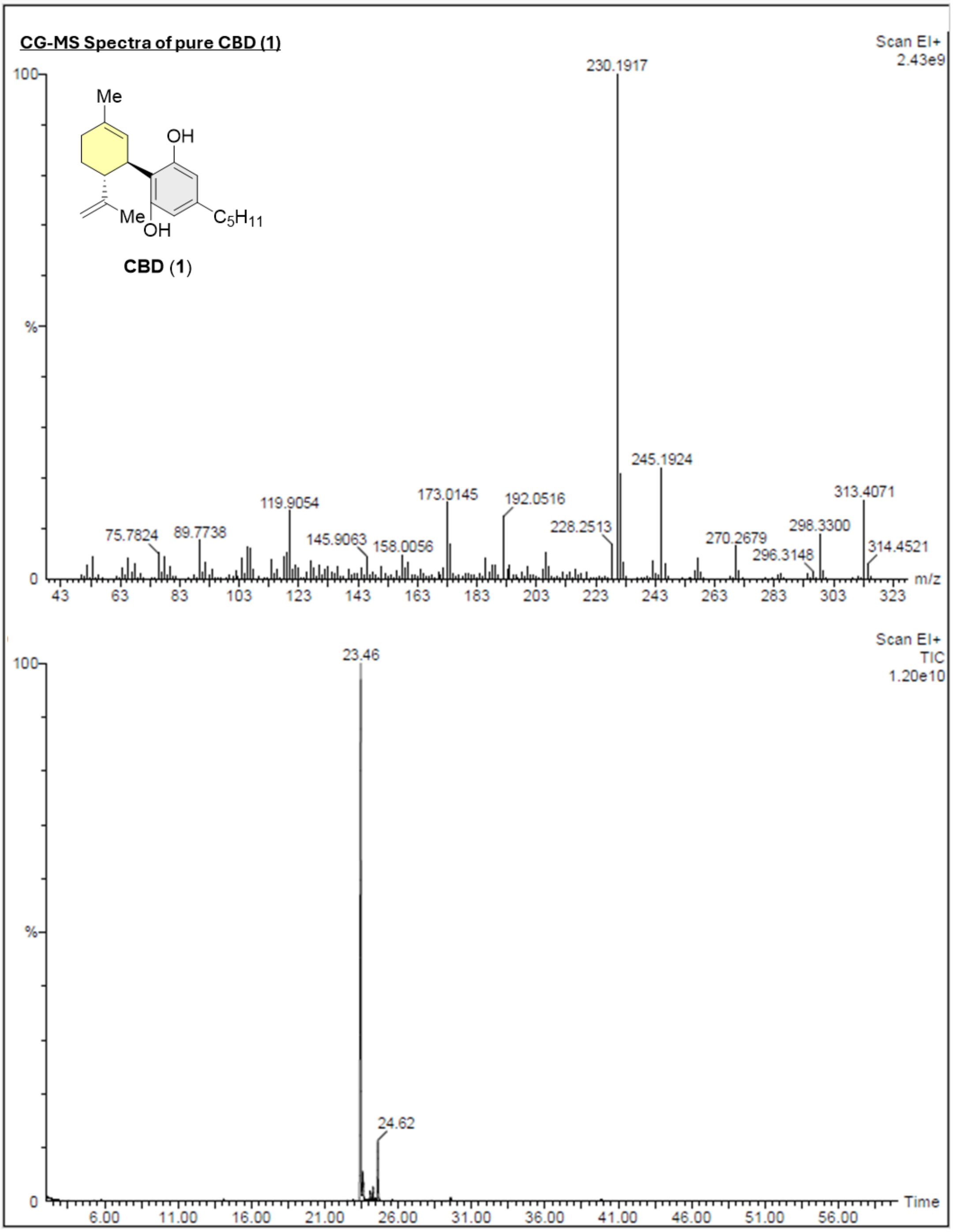

Figure S5. CG-MS of CBD (**1**).

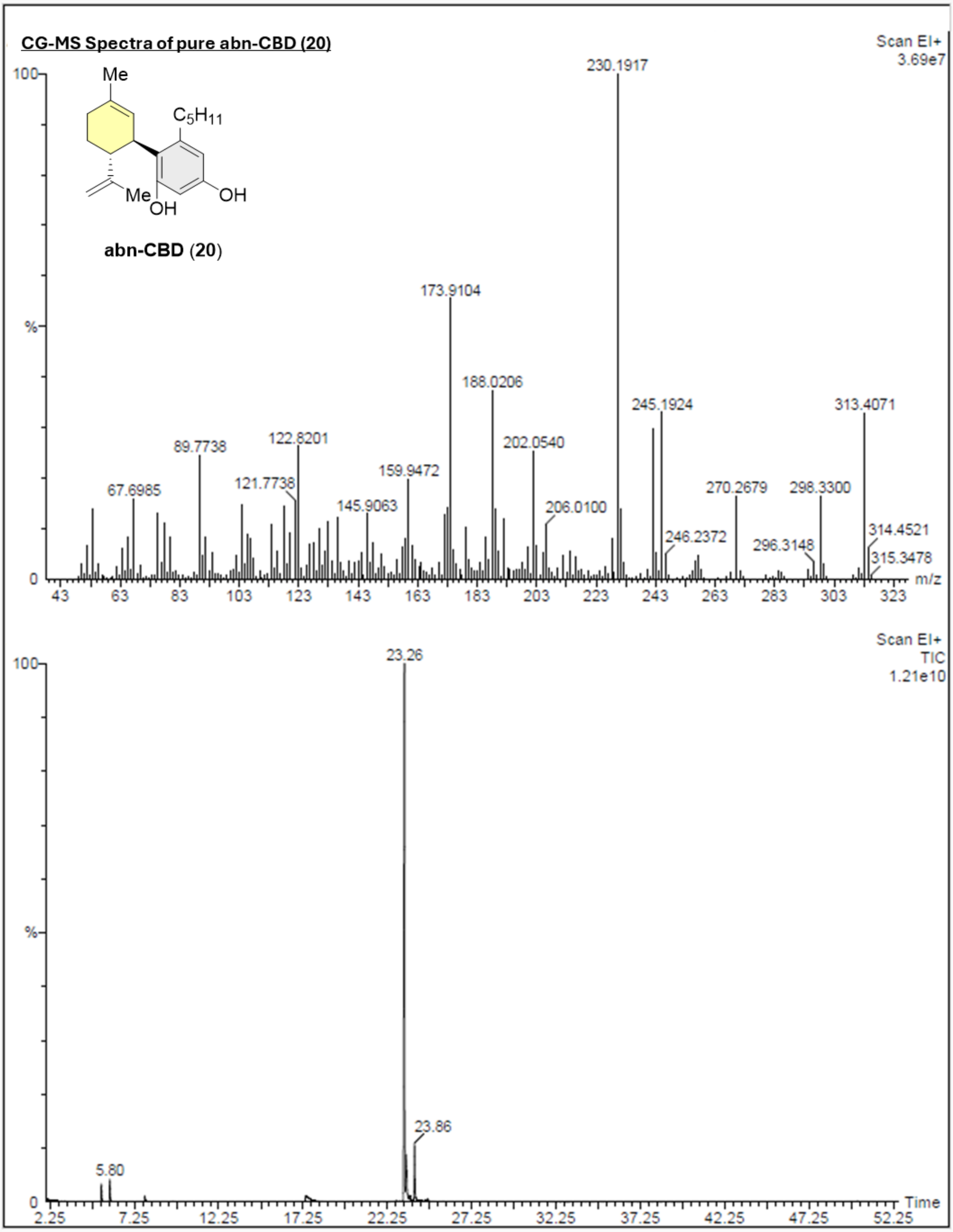

Figure S6. CG-MS of abn-CBD (**20**).

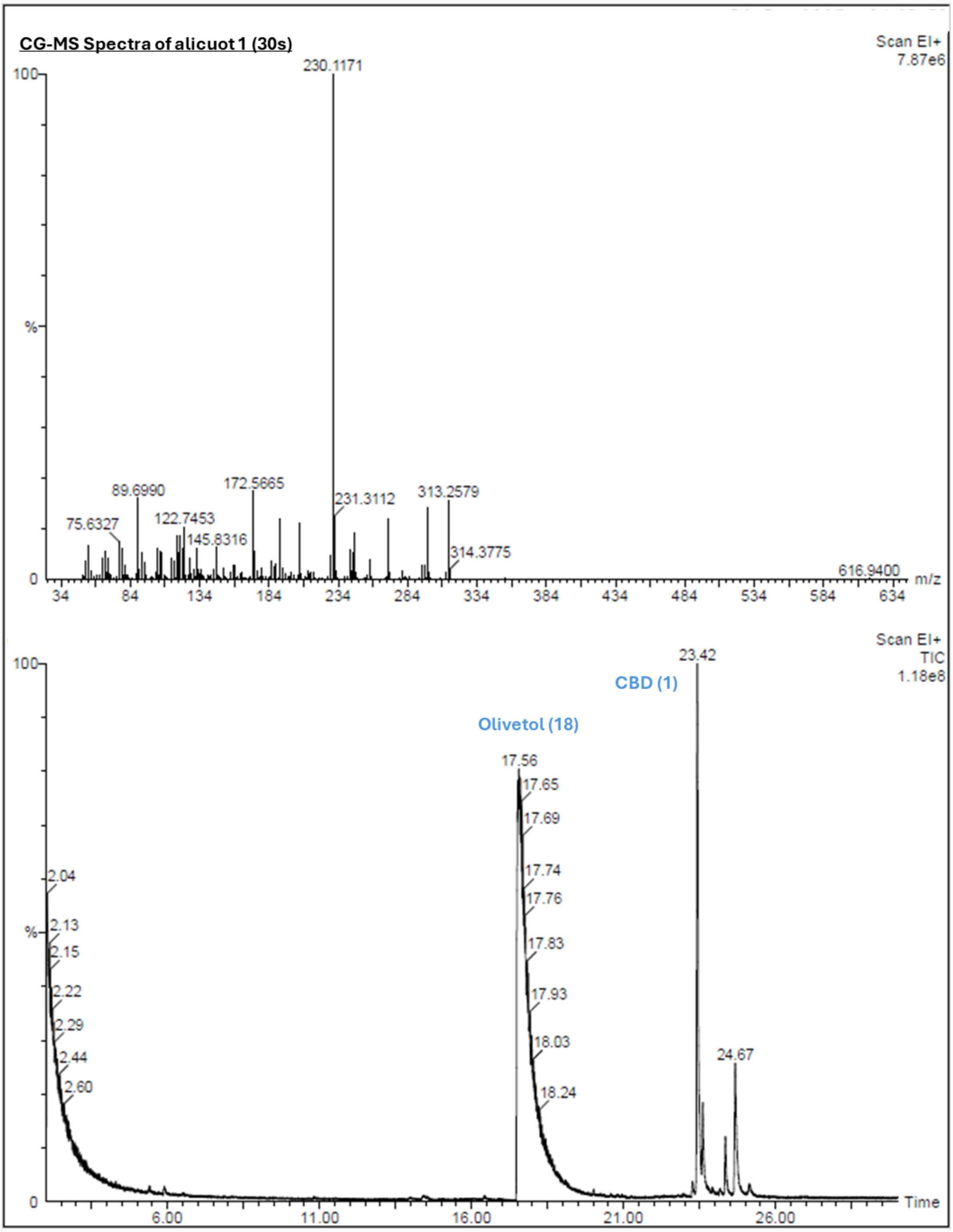

Figure S7. CG-MS of the crude mixture. Reaction time: 30 seconds.

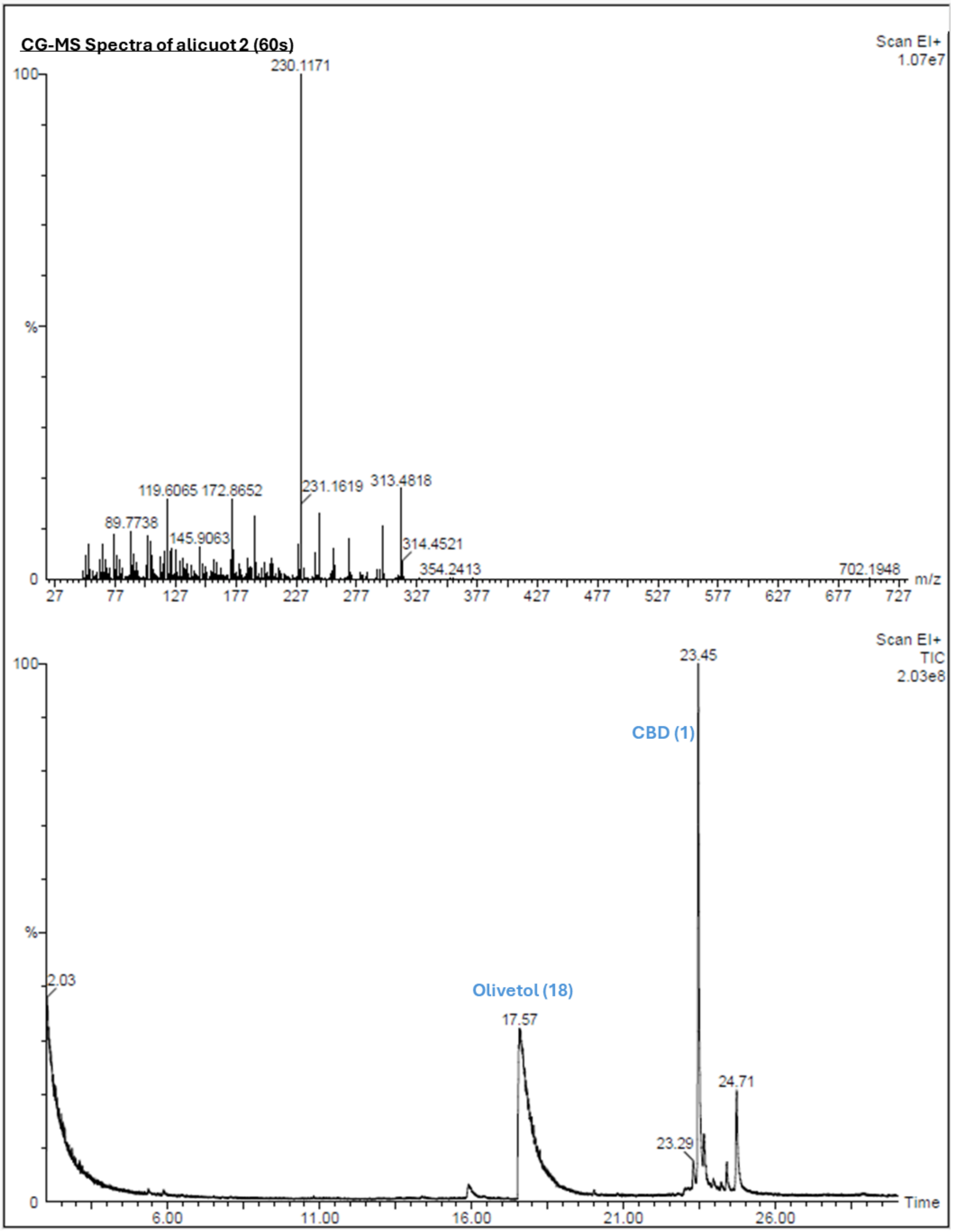

Figure S8. CG-MS of the crude mixture. Reaction time: 60 seconds.

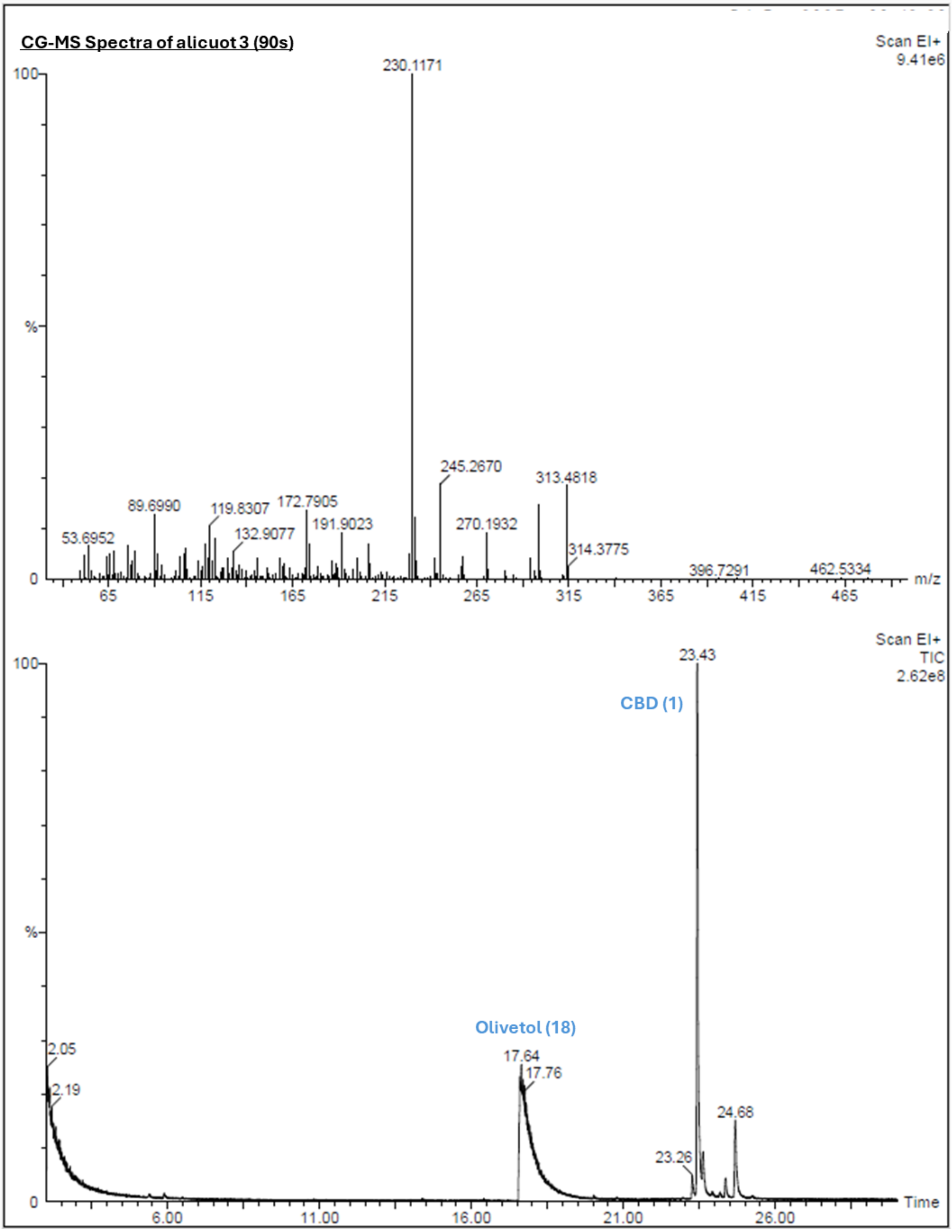

Figure S9. CG-MS of the crude mixture. Reaction time: 90 seconds.

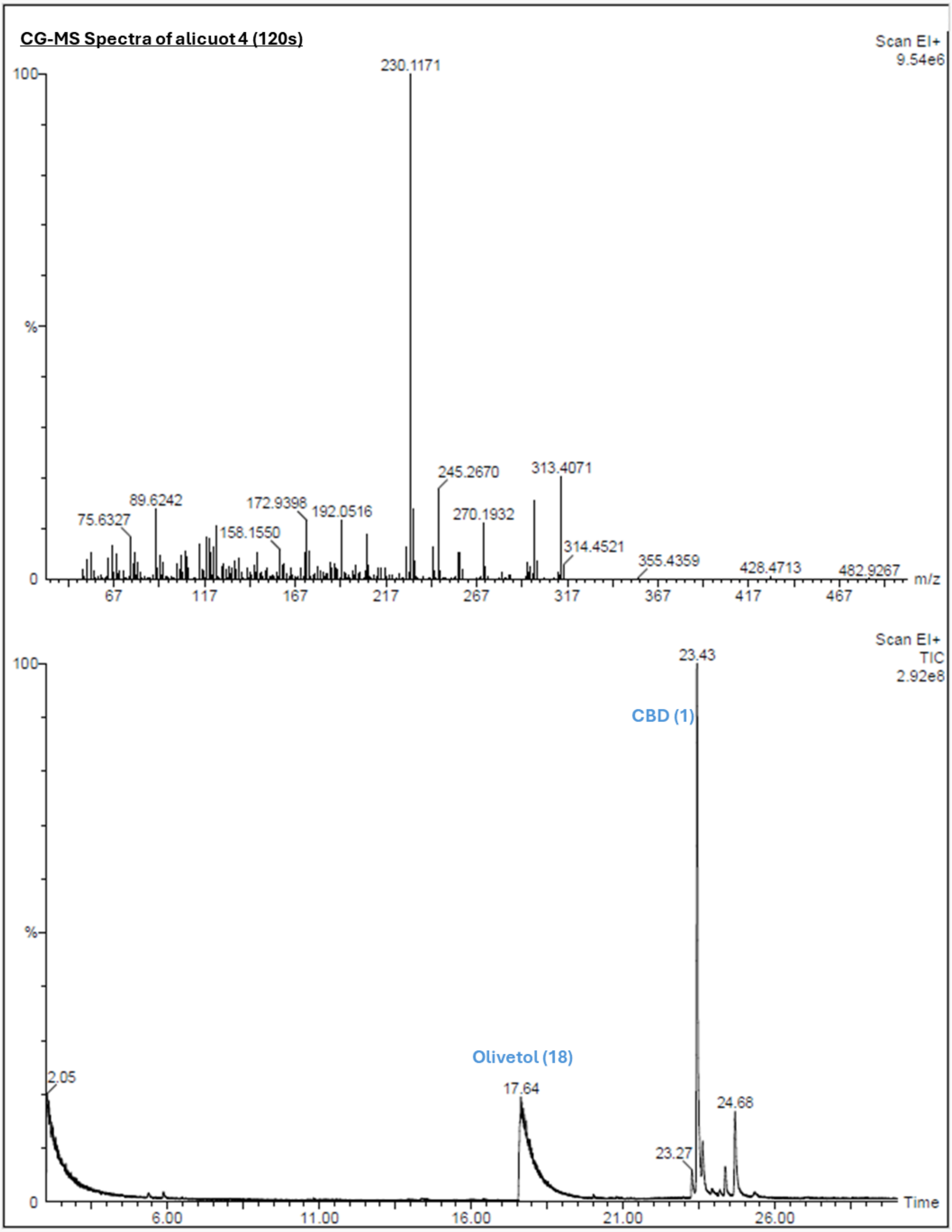

Figure S10. CG-MS of the crude mixture. Reaction time: 120 seconds.

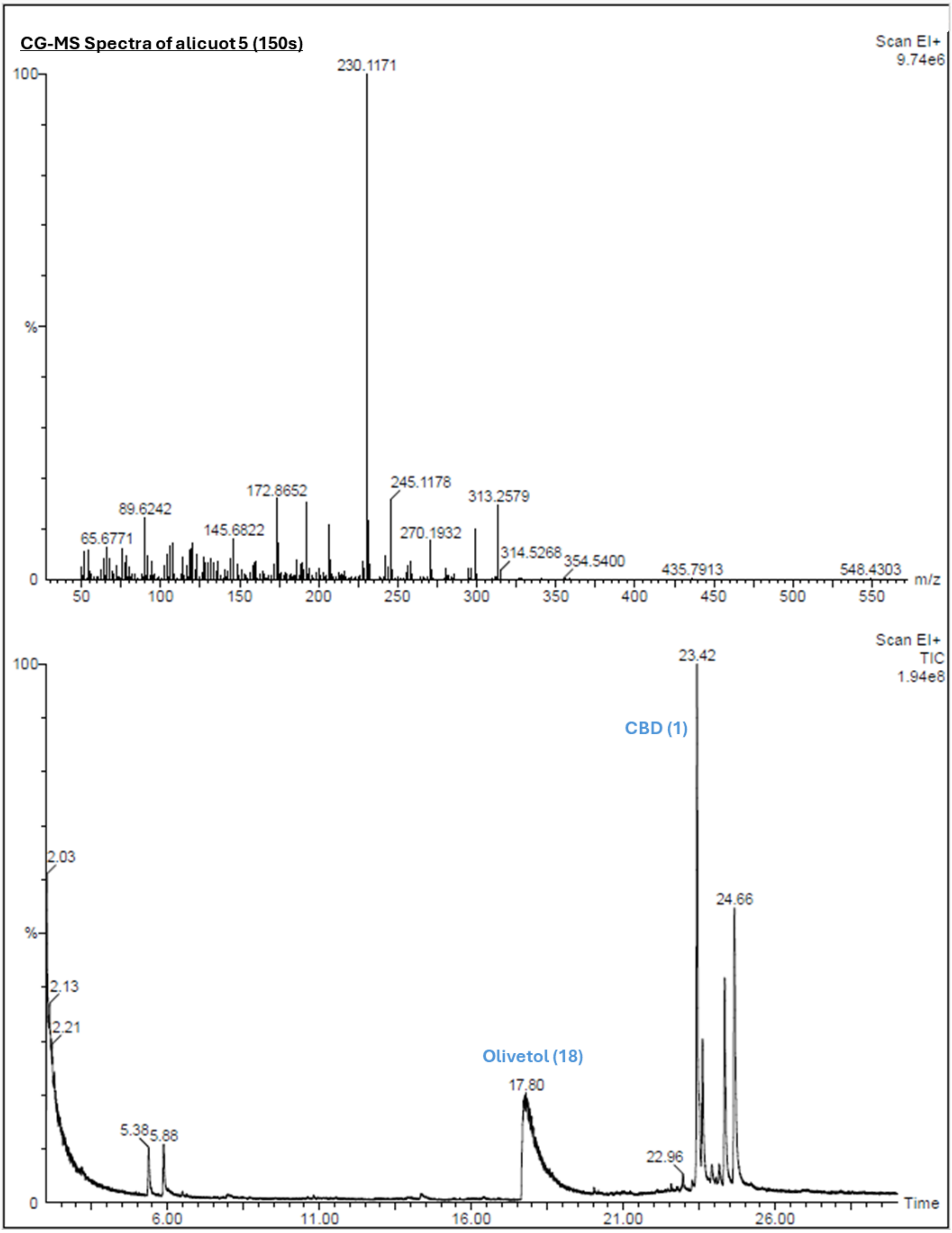

Figure S11. CG-MS of the crude mixture. Reaction time: 150 seconds.

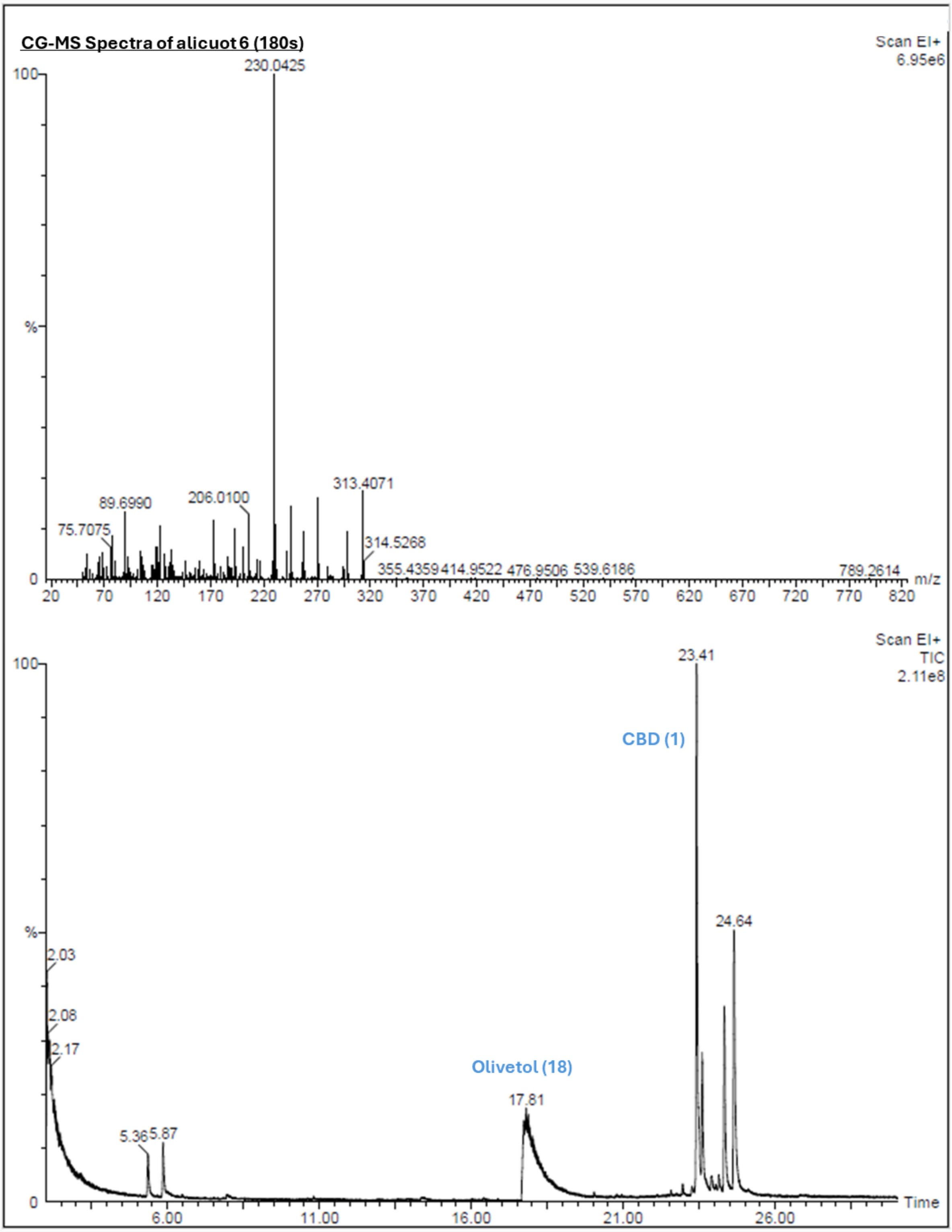

Figure S12. CG-MS of the crude mixture. Reaction time: 180 seconds.

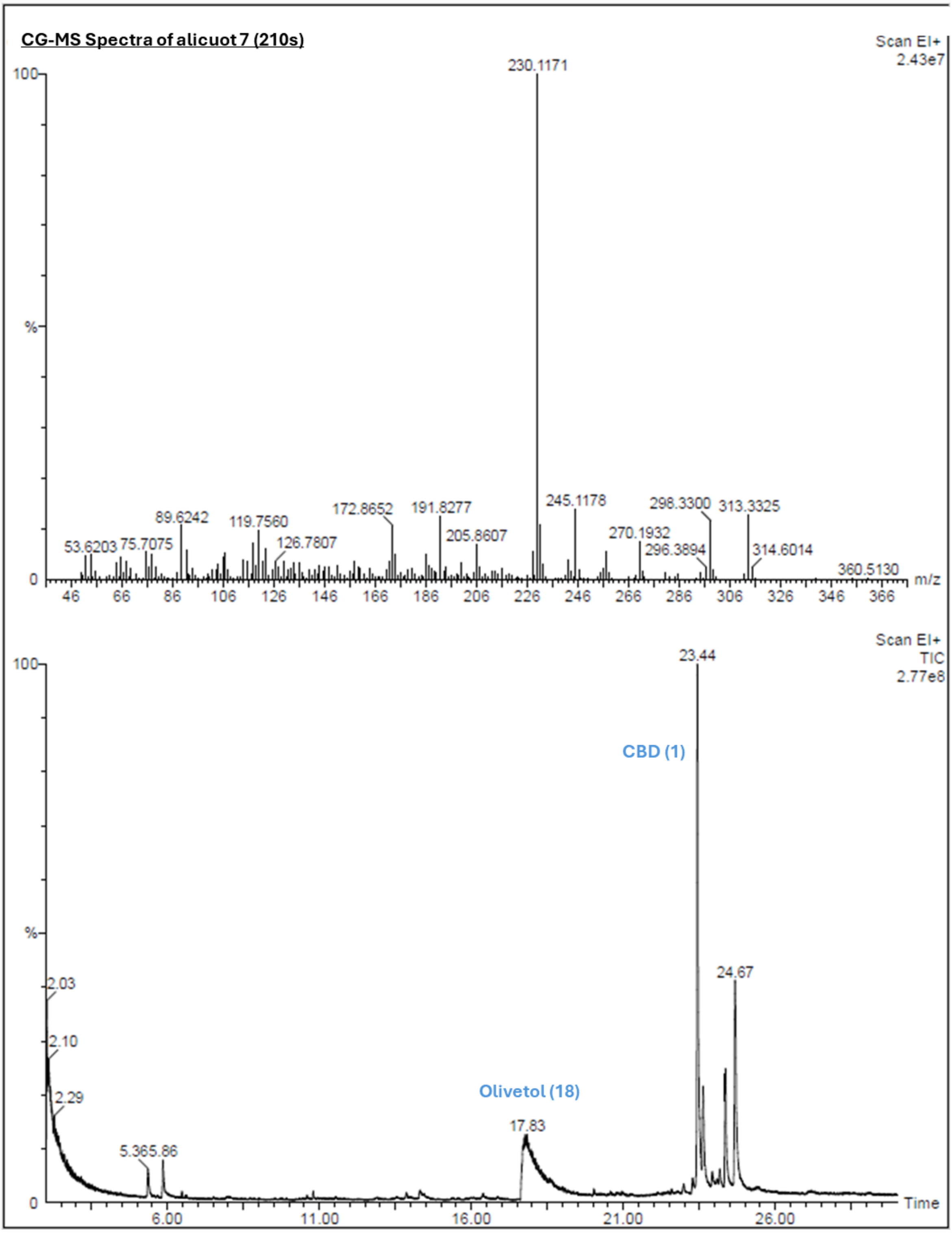

Figure S13. CG-MS of the crude mixture. Reaction time: 210 seconds.

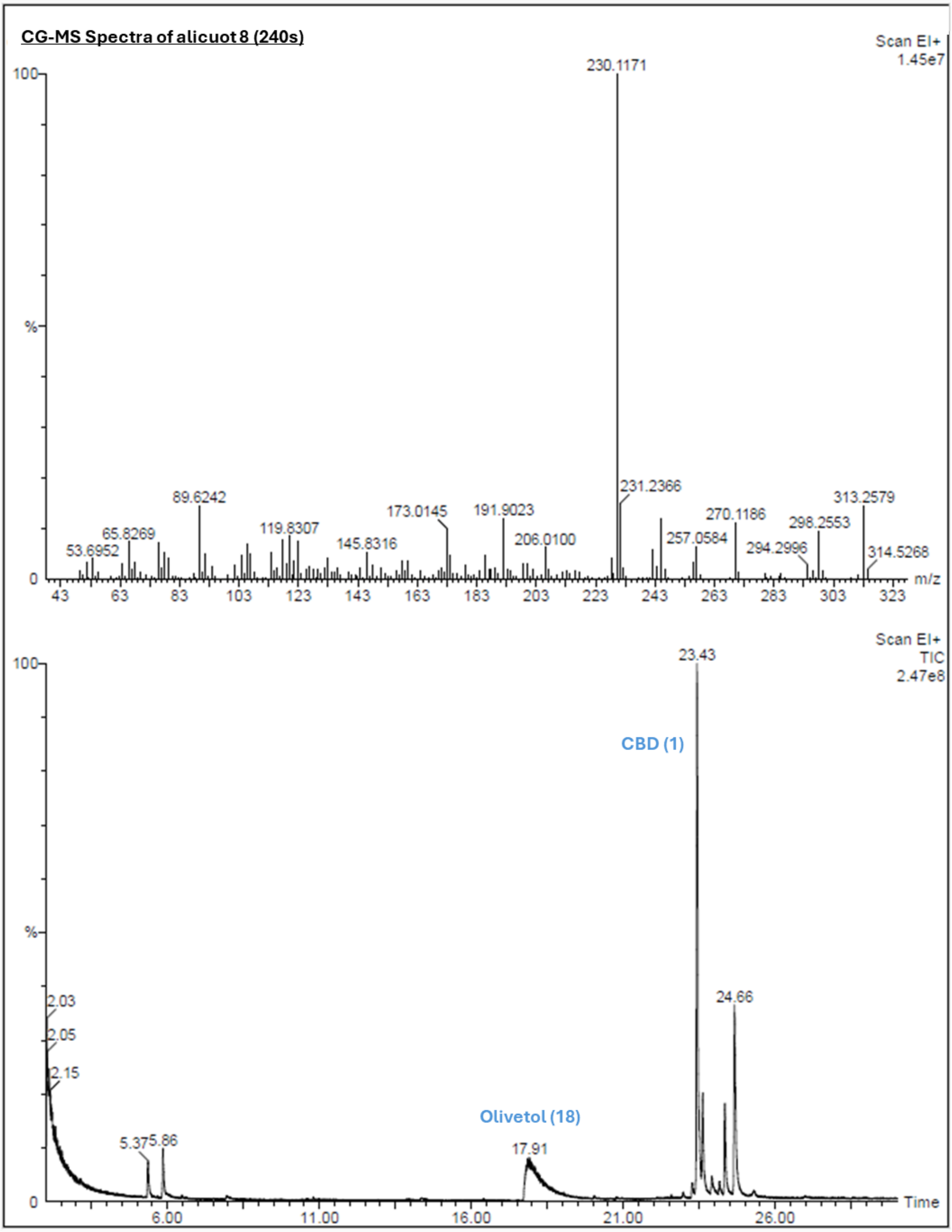

Figure S14. CG-MS of the crude mixture. Reaction time: 240 seconds.

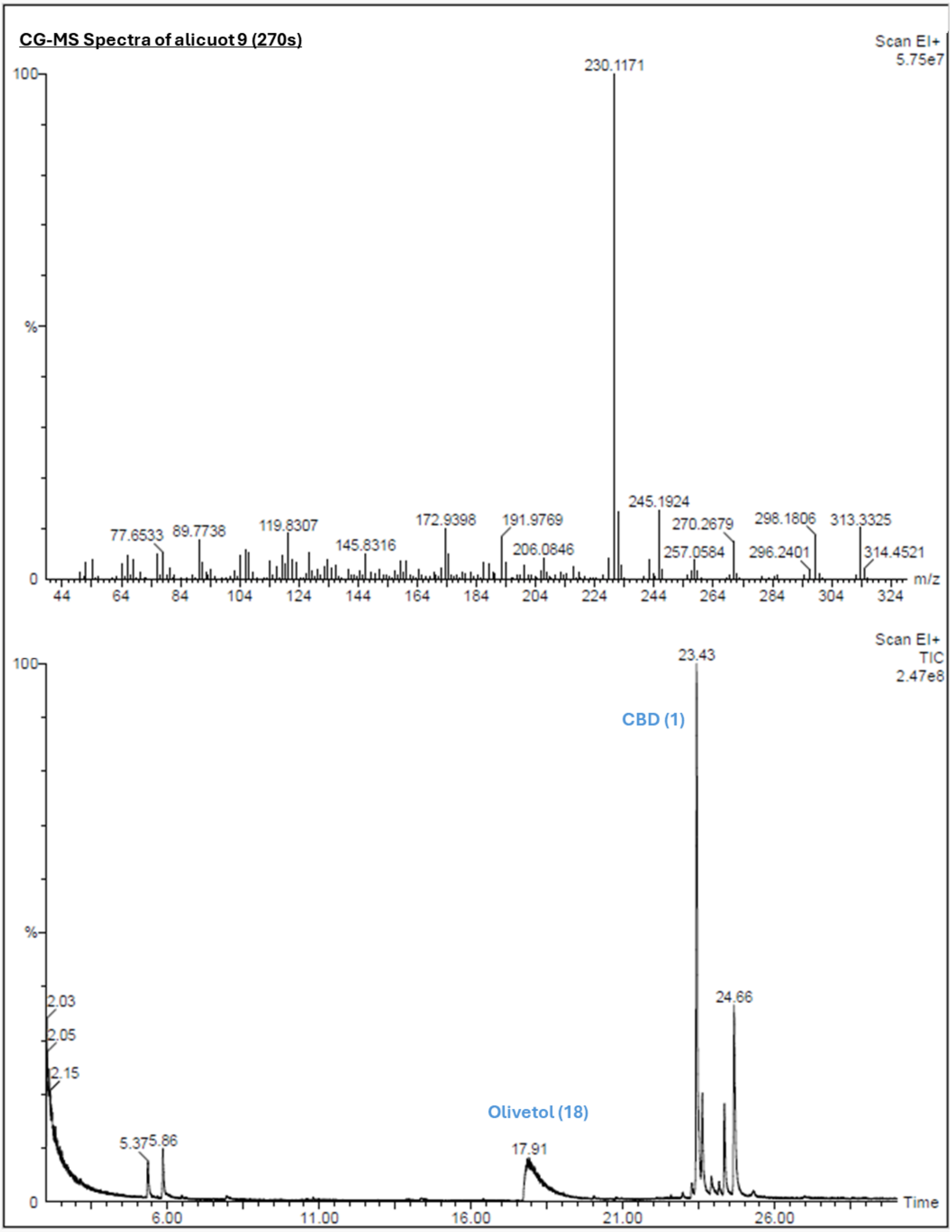

Figure S15. CG-MS of the crude mixture. Reaction time: 270 seconds.

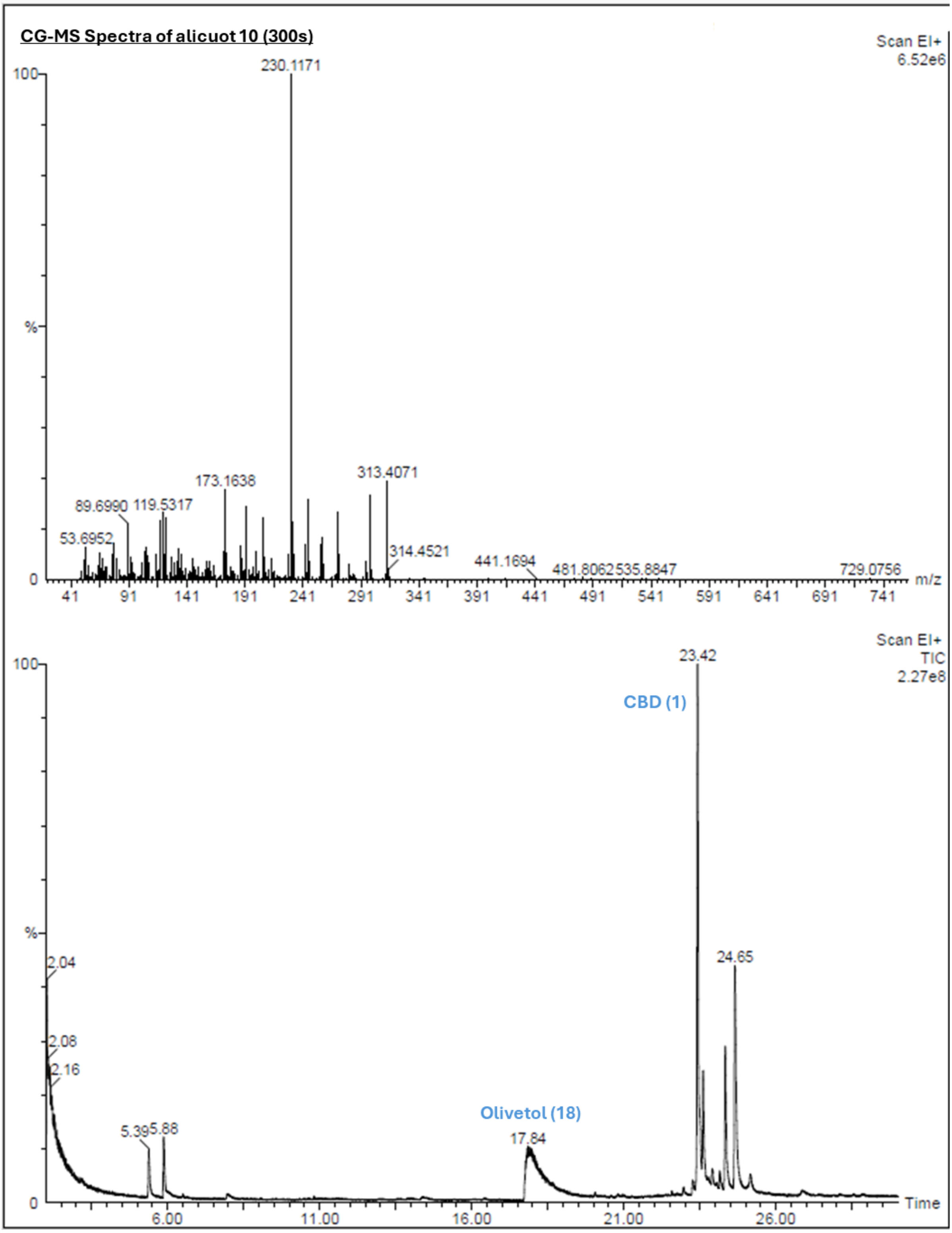

Figure S16. CG-MS of the crude mixture. Reaction time: 300 seconds.

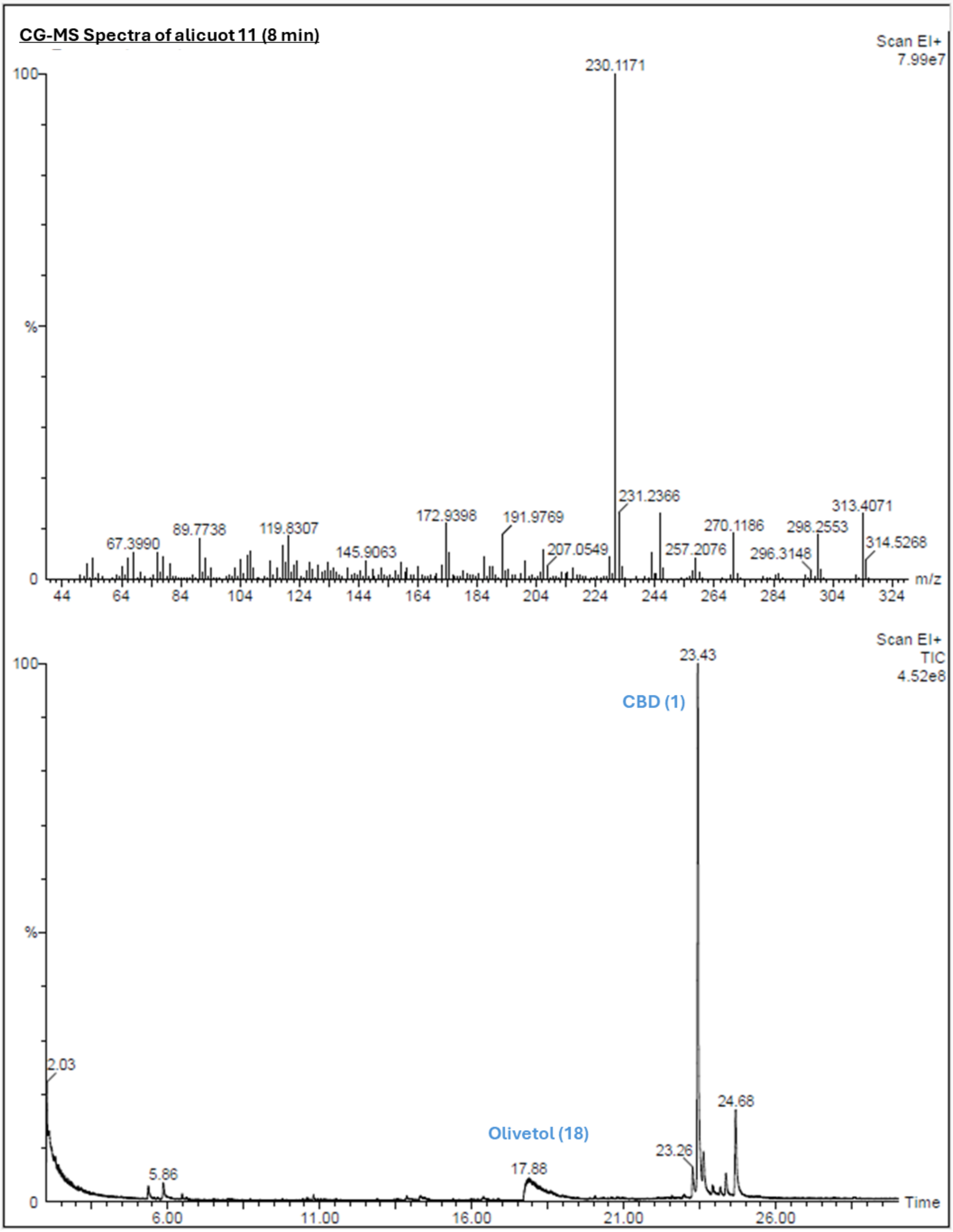

Figure S17. CG-MS of the crude mixture. Reaction time: 8 min.

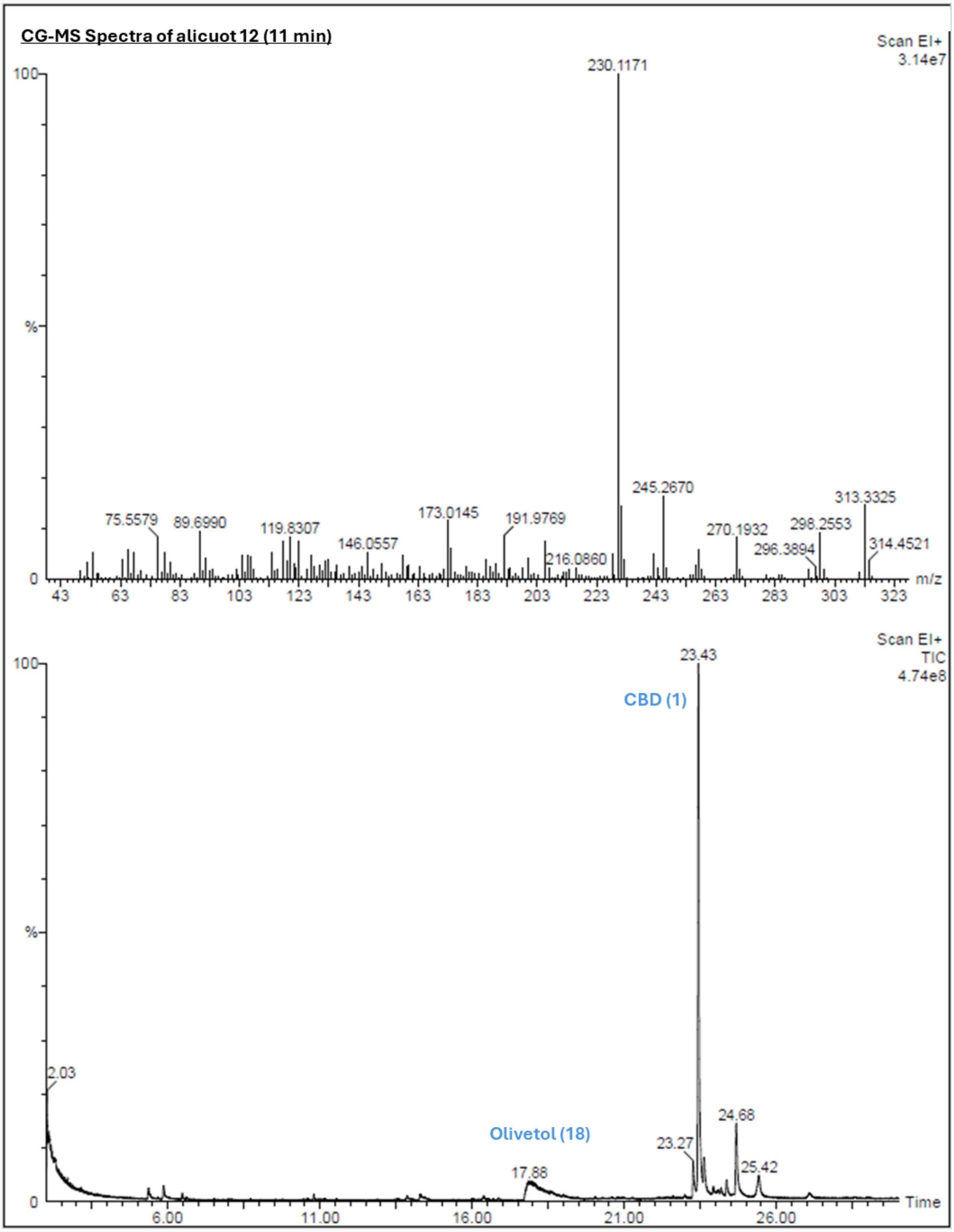

Figure S18. CG-MS of the crude mixture. Reaction time: 11 min.

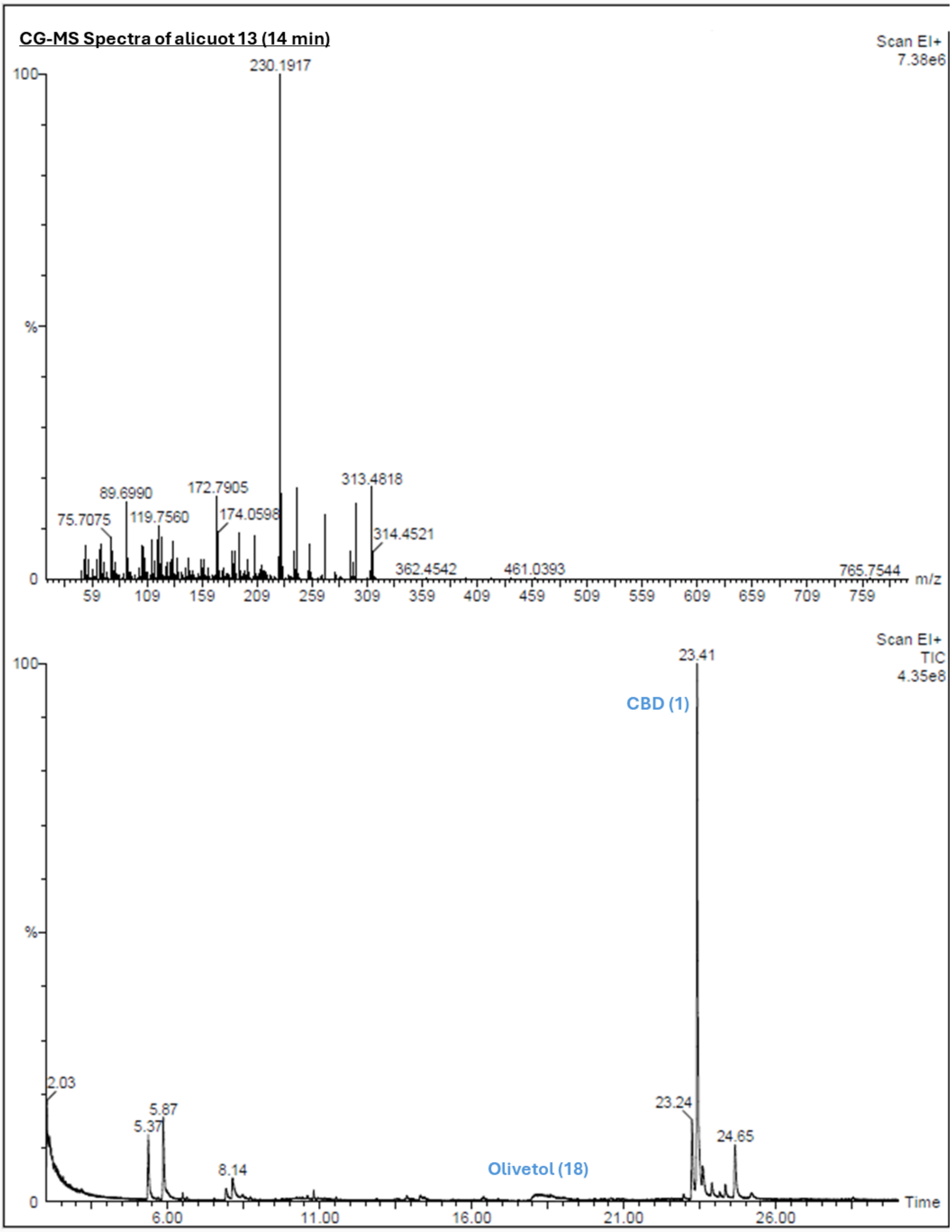

Figure S19. CG-MS of the crude mixture. Reaction time: 14 min.

5. UV-vis Experiment and Job Plot Analysis

UV-vis experiment. Six solutions were prepared in CH_2Cl_2 at a final concentration of $1.0 \cdot 10^{-3}$ M. Vial 1 contained $FeCl_3 \cdot 6H_2O$ (1.0 equiv) and olivetol (1.0 equiv); vial 2, $FeCl_3 \cdot 6H_2O$ (1.0 equiv) and olivetol (2.0 equiv); vial 3, $FeCl_3 \cdot 6H_2O$ (1.0 equiv) and olivetol (3.0 equiv); vial 4, $FeCl_3 \cdot 6H_2O$ (0.1 equiv) and olivetol (1.0 equiv); vial 5, olivetol alone (1.0 equiv); and vial 6, $FeCl_3 \cdot 6H_2O$ alone (1.0 equiv). The resulting solutions were mixed at 23 °C and transferred to quartz cuvettes. UV-vis spectra were recorded over the range 200-700 nm using CH_2Cl_2 as the blank. Relative to the spectra of the individual components, the mixtures displayed new absorption bands consistent with complex formation (see Figure 2 in the main text).

Job plot analysis.[4] To evaluate the stoichiometry of the complex formed between olivetol and $FeCl_3 \cdot 6H_2O$ in CH_2Cl_2, solutions of olivetol and $FeCl_3 \cdot 6H_2O$ of identical concentration ($1 \cdot 10^{-3}$ M) were prepared, and mixtures were made at different molar ratios while keeping the total concentration constant (Table S1). The mole fraction of olivetol ($X_{olivetol}$) was varied from 0.1 to 0.9, and the resulting solutions were analyzed by UV-vis spectroscopy at 360 nm, corresponding to an absorption maximum of the complex. For each mixture, the absorbance of the $FeCl_3 \cdot 6H_2O$ blank at the same concentration was subtracted. A plot of the corrected absorbance versus $X_{olivetol}$ gave a maximum at $X_{olivetol} = 0.495$, consistent with a 1:1 stoichiometry for the olivetol/Fe(III) complex (Figure S20).

$X_{olivetol}$	Abs_{sample} (nm)	$Abs_{FeCl3.6H2O\ blank}$ (nm)	Ideal Abs $_{FeCl3.6H2O}$ ($Abs_{FeCl3.6H2O\ blank}$*$X_{olivetol}$) (nm)	Abs_{sample} – Ideal $Abs_{FeCl3.6H2O}$ (nm)
0	2.788	2.788	2.788	0
0.1	2.648	2.788	2.5092	0.1388
0.2	2.89	2.788	2.2304	0.6596
0.3	2.712	2.788	1.9516	0.7604
0.4	2.629	2.788	1.6728	0.9562
0.5	2.668	2.788	1.394	1.274
0.6	2.256	2.788	1.1152	1.1408
0.7	1.858	2.788	0.8364	1.0216
0.8	1.589	2.788	0.5576	1.0314
0.9	1.213	2.788	0.2788	0.9342
1	0.776	2.788	0	0.776

Table S1. Data obtained from the UV-vis experiment at λ = 360 nm of different $X_{olivetol}$.
$X_{olivetol} = n_{olivetol}/(n_{olivetol} + n_{FeCl3.6H2O})$.

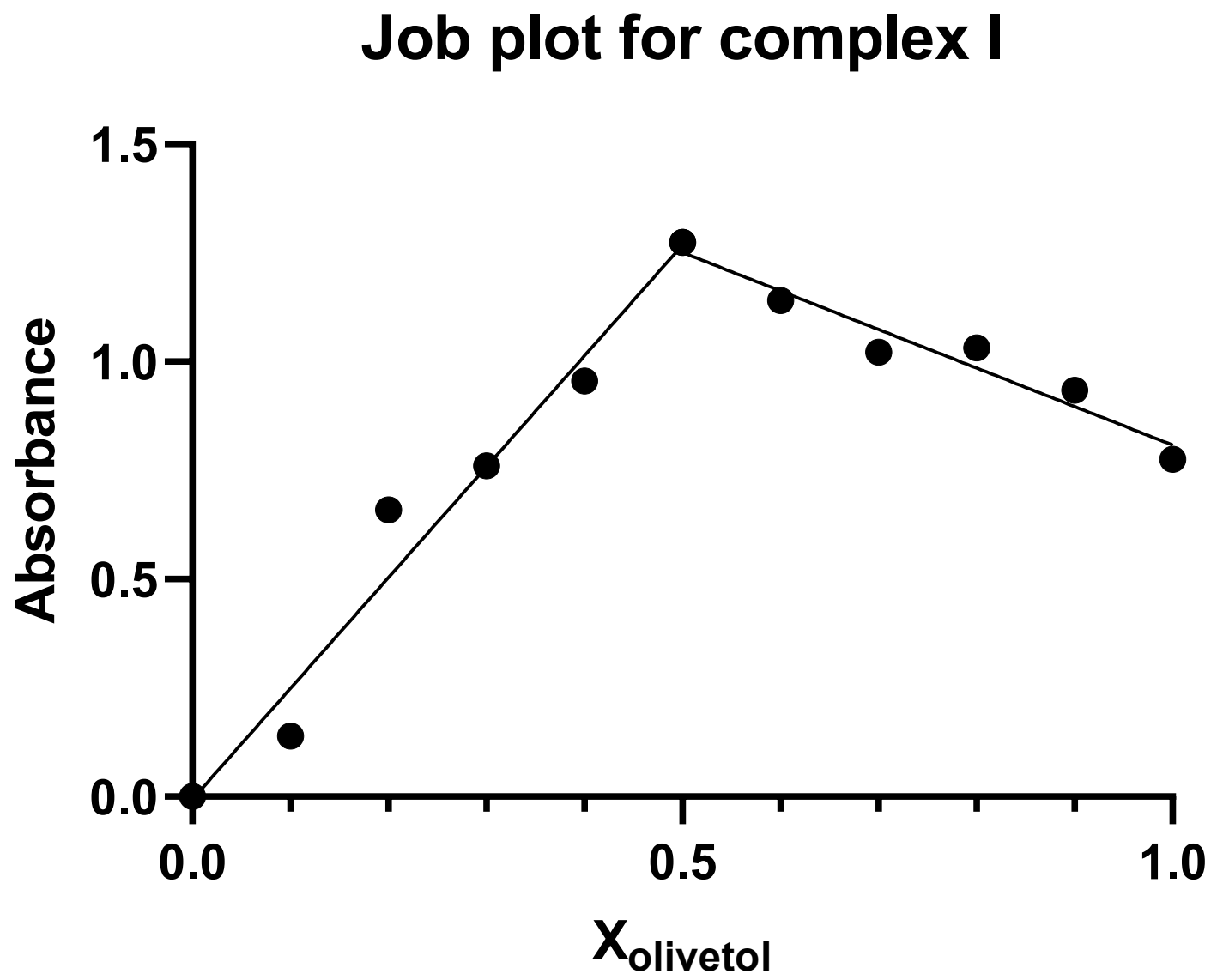

Figure S20. Job plot for complex I. Absorbance in nm.

6. Computational Studies

All density functional theory (DFT) calculations were performed using the ORCA 6.1.1 program package.[5,6] Potential energy surface exploration, including relaxed surface scans and the full geometry optimization of all stationary points (pre-reaction π-complexes and transition states), was carried out using the B3LYP functional in conjunction with the def2-SVP basis set. To accurately account for non-covalent interactions, Grimme's D4 dispersion correction was applied.[7] Bulk solvent effects for dichloromethane were implicitly incorporated throughout all calculations using the Conductor-like Polarizable Continuum Model (CPCM). To refine the energetic values, single-point energy calculations were subsequently performed on the def2-SVP optimized geometries using the extended def2-TZVPP basis set.

Vibrational frequency calculations performed directly at the B3LYP-D4/def2-SVP level on the optimized structures confirmed true minima (zero imaginary frequencies) for the pre-reaction π-complexes and first-order saddle points (exactly one imaginary frequency) for the transition states. The zero-point vibrational energies (ZPVE) and thermal corrections to the Gibbs free energy at 298.15 K were added to the def2-TZVPP electronic energies to obtain the final absolute thermodynamic values. As noted by Harding *et al.*,[8] transition states and loosely bound complexes located on flat potential energy plateaus represent highly fluxional regions characterized by large-amplitude, anharmonic motions. Because standard rigid-rotor harmonic-oscillator (RRHO) treatments fail to accurately model these configurations and heavily over-penalize low-frequency modes, thermochemical corrections were computed utilizing the quasi-RRHO scheme.[9] This correction interpolates low-frequency vibrational modes into free-rotor partition functions, applying a frequency cutoff value (ω_0) of 100 cm^{-1}, ensuring physically meaningful absolute Gibbs free energies for all evaluated trajectories.

6.1. Computational Justification of the Ground and Reactive Spin State

The reactivity of Fe(III) complexes in intermediate ligand-field environments is governed by an intricate Potential Energy Surface (PES) where the sextet (S = 5/2), quartet (S = 3/2), and doublet (S = 1/2) states might thermodynamically compete.[10,11] To establish the spin landscape in the initial formation of the π-complex, relaxed PES scans were performed for the three spin states. As shown in Figure S21, the sextet state remained the global minimum throughout the approach. The intermediate quartet state exhibited a severe and abrupt thermodynamic barrier (+14.3 kcal/mol), while the doublet state suffered an even higher destabilization (+23.7 kcal/mol), rendering both states kinetically inaccessible under the reaction conditions.

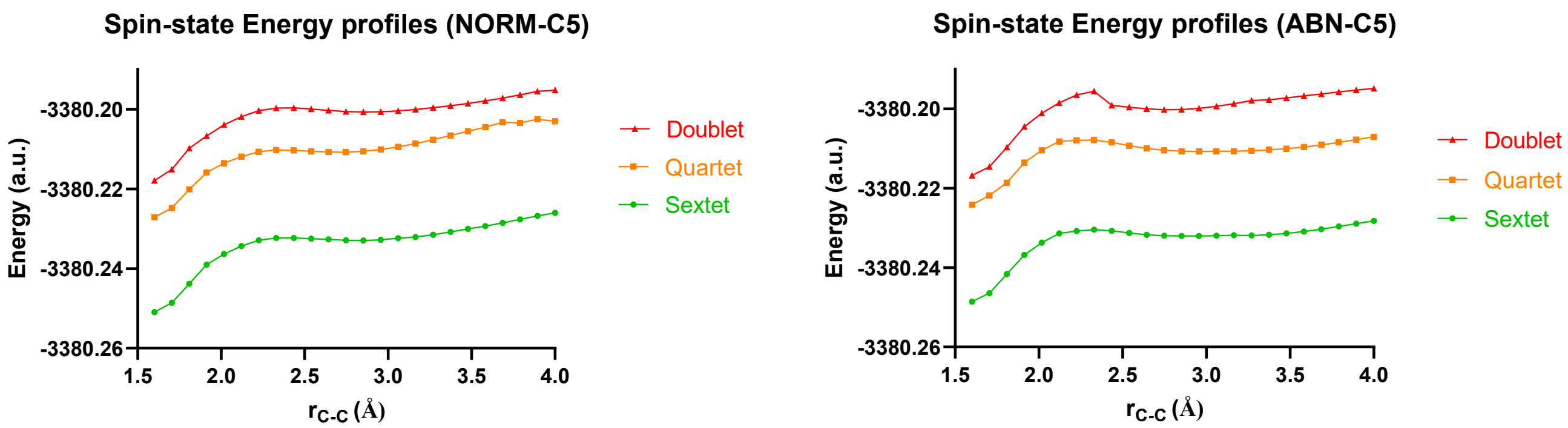

Figure S21. Relaxed potential energy surface (PES) scans for the C–C bond formation along the normal (NORM-C5, left) and abnormal (ABN-C5, right) trajectories. Total electronic energies are plotted in atomic units (a.u.) against the forming C–C bond distance ($r_{C\text{–}C}$) in angstroms (Å). The profiles evaluate the sextet (green), quartet (orange), and doublet (red) spin states. The ground-state sextet surface remained the global minimum across the entire reaction coordinate. The massive energetic penalties associated with the low-spin configurations were rigidly maintained throughout the bimolecular approach, demonstrating the absence of any accessible minimum energy crossing points (MECPs) and establishing that the entire regioselectivity-determining step proceeds exclusively on the high-spin sextet manifold.

From these results, the explicit optimization of transition state geometries for the quartet and doublet manifolds was considered redundant, since the relaxed surface scans demonstrated that the massive energetic penalties of the low-spin pre-complexes (+14.3 and +23.7 kcal/mol) were strictly maintained along the entire C–C coupling coordinate. The absolute absence of Minimum Energy Crossing Points (MECPs) dictates that these spin states cannot kinetically compete with the ground sextet manifold, rendering further geometric characterization of their saddle points unnecessary.

6.1.1. Unrestricted Wavefunction Analysis Methodology

Standard unrestricted Kohn-Sham (UKS) calculations often produce highly delocalized orbitals that obscure the true chemical bonding picture in systems with high spin polarization. To extract a chemically rigorous representation of the electronic structure, two standard topological transformations were applied to the wavefunction:

- *Quasi-Restricted Orbitals (QROs):* This transformation filtered out the artificial "noise" of spin polarization to provide a clean spatial representation of the valence electrons. It allowed the accurate determination of the true energy levels of the iron d-band and pinpoint the exact spatial location of the unpaired metal radicals (occupancy ≈ 1.0).[12]
- *Unrestricted Corresponding Orbitals (UCOs):* UCOs were used to visualize and directly quantify the spatial pairing of and electrons. The transformation mathematically forced maximum spatial overlap (S) between spin-up and spin-down channels. A near-perfect overlap ($S_{\alpha\beta} \approx 1.000$)) indicated an intact, strain-free covalent framework. A significant drop ($S_{\alpha\beta} < 1.000$) directly quantified spin polarization, revealing the exact locations where formally doubly occupied bonds are physically contorting to mitigate intra-orbital Pauli repulsion. Pure, unpaired magnetic electrons were cleanly isolated with $S_{\alpha\beta}$ = 0.000.[13]

6.1.2. Electronic structure of the Sextet State (S=5/2).

The sextet state was confirmed as the thermodynamic ground state of the system. QRO analysis demonstrated that the iron d-band, containing the five magnetic electrons, was strongly stabilized by the contribution of exchange energy. Topologically, these electrons were buried deep within the potential well (distributed from the absolute HOMO down to the HOMO-7 QRO level). Thus, the sextet state minimized the overall energy of the system and avoided populating more energetic frontier orbitals. This electronic configuration prevented intra-orbital Pauli repulsion, allowing the iron center to act as a stable, non-interfering coordinative template, accommodating the approaching substrate to form the pre-reactive π-complex and establishing the necessary steric and electronic architecture for the subsequent substrate-centered C-C coupling without the severe thermodynamic penalties that accompany forced electron pairing in low-spin states.

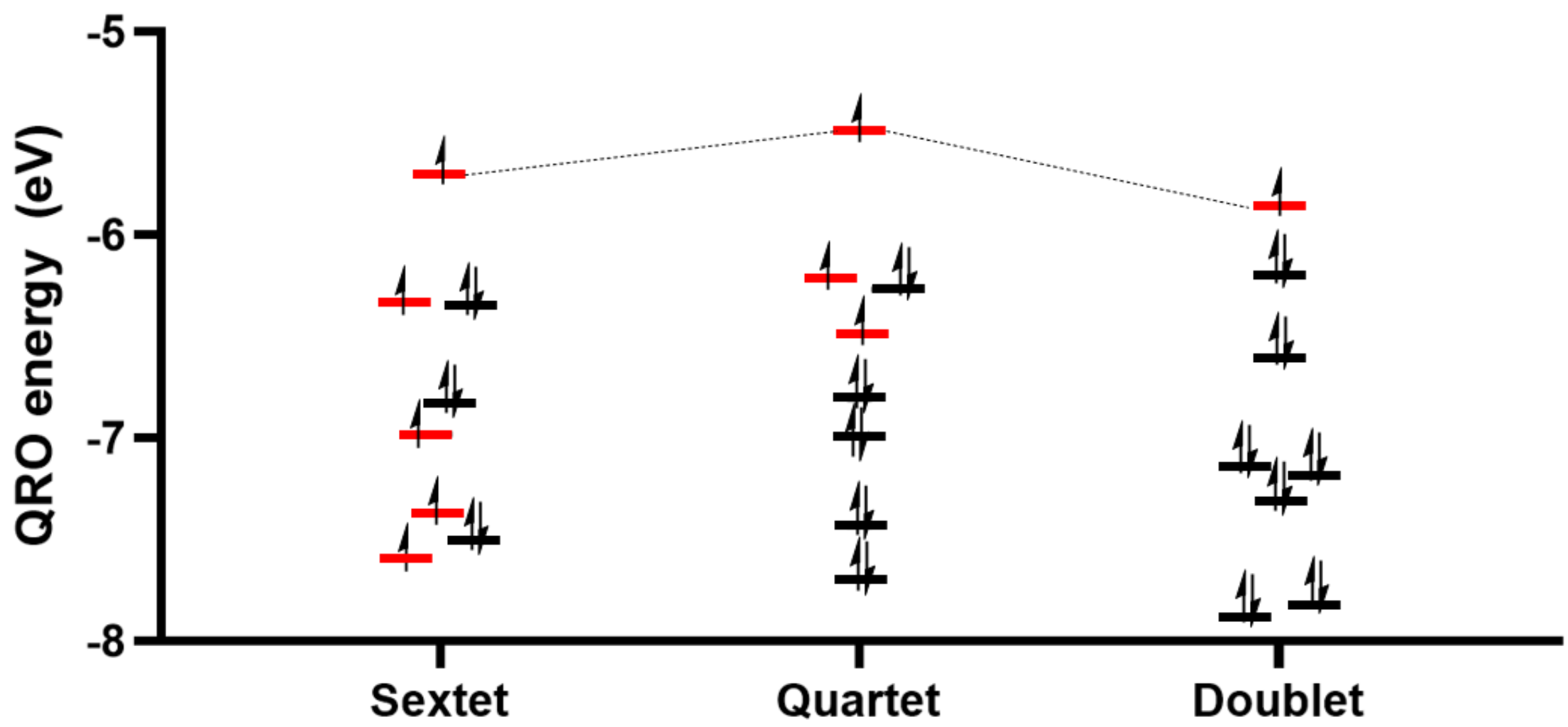

Figure S22. Frontier Quasi-Restricted Orbitals (QRO) energy diagram for the optimized pre-transition state π-complexes. Singly occupied orbitals (red) highlight the relative electronic destabilization across different multiplicity states. In the ground sextet state, the highest-energy metal radical resided at a relatively stable frontier (~ -5.7 eV), while the remaining magnetic orbitals are deeply buried (down to ~ -7.6 eV) due to maximal exchange stabilization. Forcing the system into the quartet state (S=3/2) induced severe intra-orbital Pauli repulsion, pushing the metal radical upwards to a more unstable level (~ -5.5 eV) and

compressing the entire d-band to higher energies. The doublet state (S=1/2) electronically relaxed this frontier but was rendered inaccessible due to a structural reorganization energetic penalty.

6.1.3. Electronic structure of the Quartet State (S=3/2).

Constraining the system to the quartet state required the pairing of two metal electrons, resulting in a thermodynamic penalty of +14.3 kcal/mol. The geometry and UCO analyses revealed that this barrier originated from severe, localized electronic strain: the complex attempts to accommodate the forced S=3/2 configuration while maintaining the broad architectural framework of the ground sextet, specifically retaining a comparable olivetol orientation ($\tau_{(Fe\text{-}O1\text{-}C1\text{-}C2)} \approx -105^{\circ}$, see figure S24 for atomic labels) and equatorial Fe-OH_2 distances (≈2.19 Å). This topological rigidity provided no geometric relief, trapping the Pauli repulsion within the coordination framework. The system mitigated this strain through localized spin polarization, directly observed in the spatial decoupling of the formally doubly occupied metal-ligand pairs ($S_{\alpha\beta}$=0.9120). As corroborated by the QRO levels, this internal electronic fracturing destroyed the exchange stabilization and pushed the entire d-band toward higher energies than the sextet, resulting in the net magnetic density emerging at the absolute frontier. Consequently, while the coordination framework remained intact and did not collapse, the extreme energetic penalty of forcing the system into these destabilized electronic levels imposed an insurmountable energetic barrier, rendering this spin-state pathway entirely inaccessible.

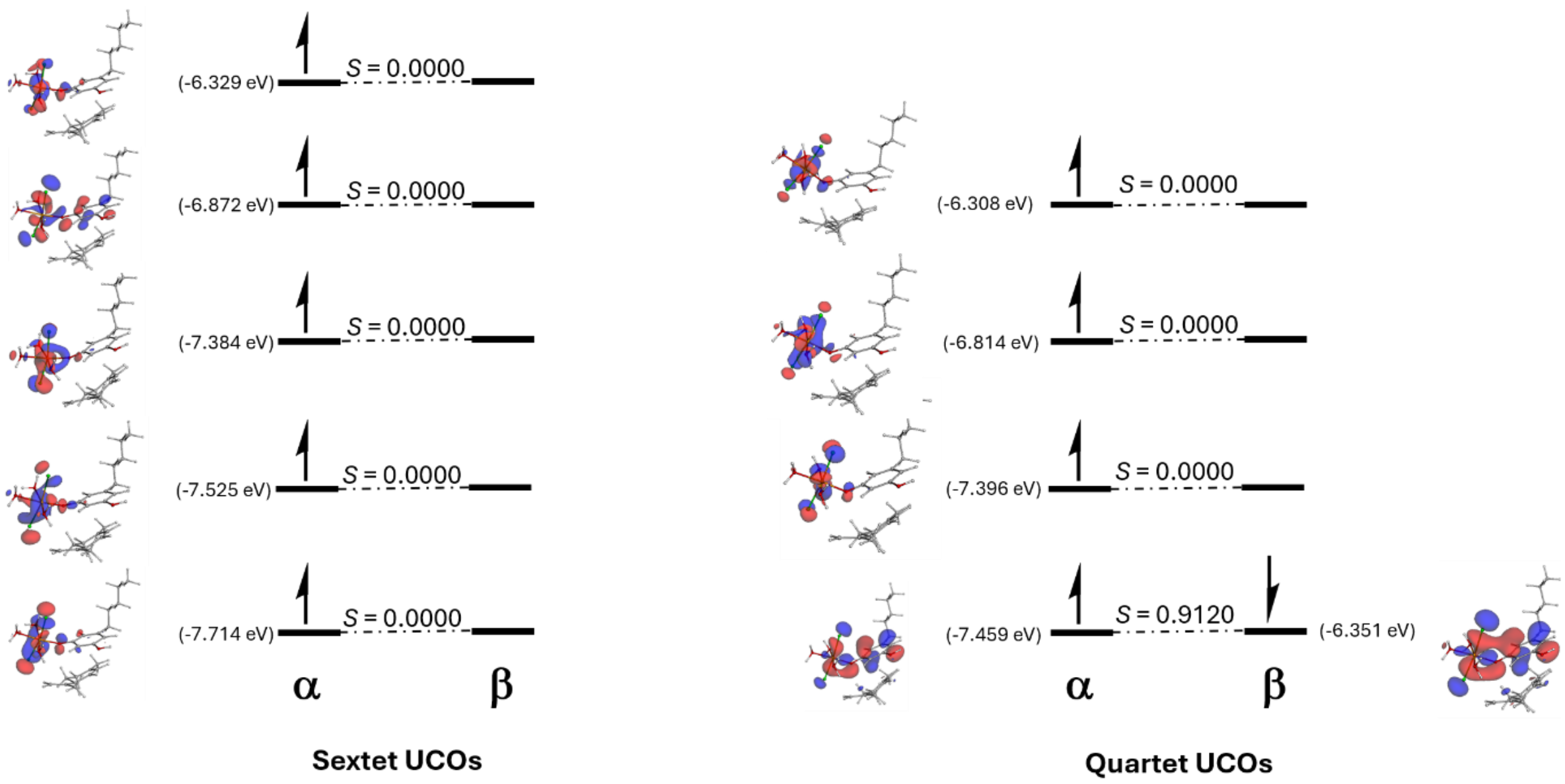

Figure S23. Unrestricted Corresponding Orbital (UCO) diagrams for the optimized pre-reactive π-complexes in the ground sextet and quartet states. Energy levels (in eV) and spatial overlap integrals (*S*) between the α and β channels are provided for the frontier metal *d*-band. In the sextet state (left), the five unpaired electrons represent pure, fully stabilized magnetic density (*S* = 0.0000). In the quartet state (right), the forced pairing of two electrons within the rigid structural framework induces severe intra-orbital Pauli repulsion. This localized electronic strain is mathematically and visually evidenced by the spin polarization of the formally doubly occupied pair, which drops the spatial overlap to *S* = 0.9120 and spatially decouples the α and β electron densities.

6.1.4. Macroscopic Structural Collapse of the Doublet State (S=1/2).

The fundamental mechanism of mitigating Pauli repulsion diverged completely in the doublet state. The higher intrinsic repulsion of forcing an S=1/2 multiplicity could not be accommodated through localized orbital polarization, triggering instead structural modifications.

UCO analysis revealed that the core coordination framework of the doublet state was remarkably stable, with spatial overlaps of the formally doubly occupied pairs fully restored ($S_{\alpha\beta}$=0.9808 and 0.9979). This lack of severe spin polarization confirmed that the thermodynamic penalty did not originate from any internal topological fracturing. Instead, the system achieved this internal electronic pairing at the cost of substantial geometric contortion, with the equatorial waters contracting to distances < 2.00 Å, and the olivetol substrate undergoing a severe torsional twist ($\Delta\tau_{(Fe\text{-}O1\text{-}C1\text{-}C2)}$ ≈120º).

Table S2. Selected geometric parameters (distances in Å, angles in degrees) for the optimized pre-transition state complexes in the sextet, quartet, and doublet electronic states.

Geometric parameters	Sextet	Quartet	Doublet
r(Fe-O1)	1.859	1.849	1.828
r(Fe-O4)	2.204	2.03	2.025
r(Fe-Cl1)	2.389	2.288	2.281
r(Fe-Cl2)	2.316	2.322	2.304
r(Fe-O2)	2.144	2.185	1.985
r(Fe-O3)	2.134	2.194	1.987
τ(Fe-O1-C1-C2)	-130.933	-105.497	-10.461

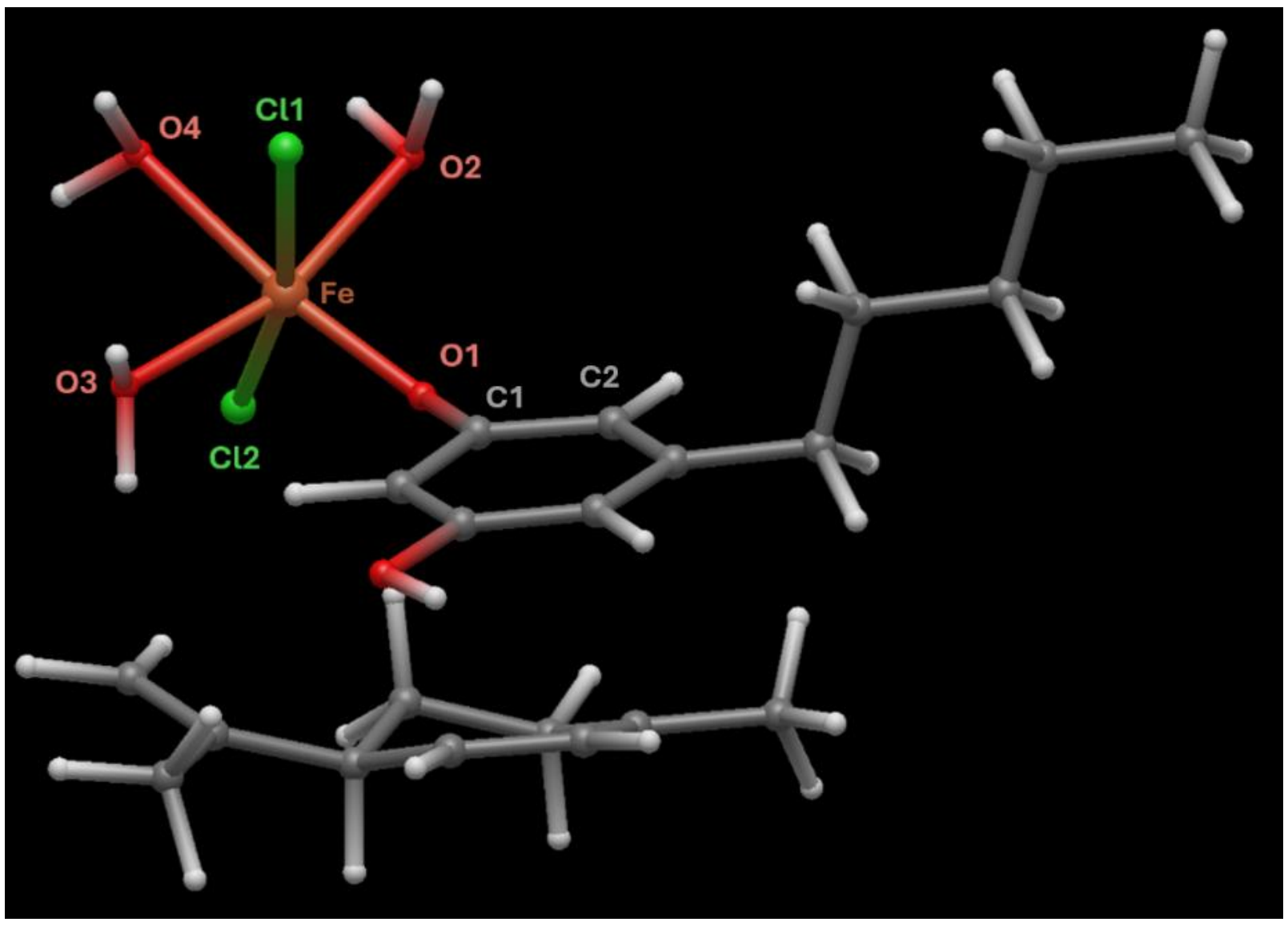

Figure S24. Key geometric parameters and atom-labeling scheme for the optimized pre-reactive π-complexes across the sextet, quartet, and doublet spin states. Bond distances (r) are provided in angstroms (Å) and the torsion angle (τ) in degrees. The 3D model illustrates the structural framework. Consistent with the topological analysis, the quartet state largely retains the spatial architecture of the ground sextet, specifically maintaining comparable equatorial distances and a wide olivetol orientation (τ ≈ -105º). Conversely, the doublet state exhibits extreme geometric distortion, evidenced by the sharp contraction of the equatorial water ligands ($r_{(Fe\text{–}O2)}$ and $r_{(Fe\text{–}O3)}$ < 2.00 Å) and the forced rotation of the

substrate into a constrained dihedral alignment (τ ≈ -10º) dictated by the asymmetric d_π electron demand of the low-spin metal center.

This distortion was a strict stereoelectronic consequence of its t_{2g}^5 configuration. The substrate was mechanically forced to rotate into a specific dihedral alignment (τ ≈ −10º) to maximize p→d_π orbital overlap with the single half-vacant t_{2g} orbital (asymmetric π-locking), an effect demonstrated in structural studies of low-spin iron(III) complexes with planar axial ligands.[14] While the complex maintained its geometrical integrity and could accommodate the C-C coupling trajectory, the nuclear reorganization energy required to force the substrate into this specific alignment imposed a steep thermodynamic penalty. Thus, the energetic cost of satisfying this d_π vacancy entirely destabilized the doublet surface, rendering it inaccessible during the bimolecular approach.

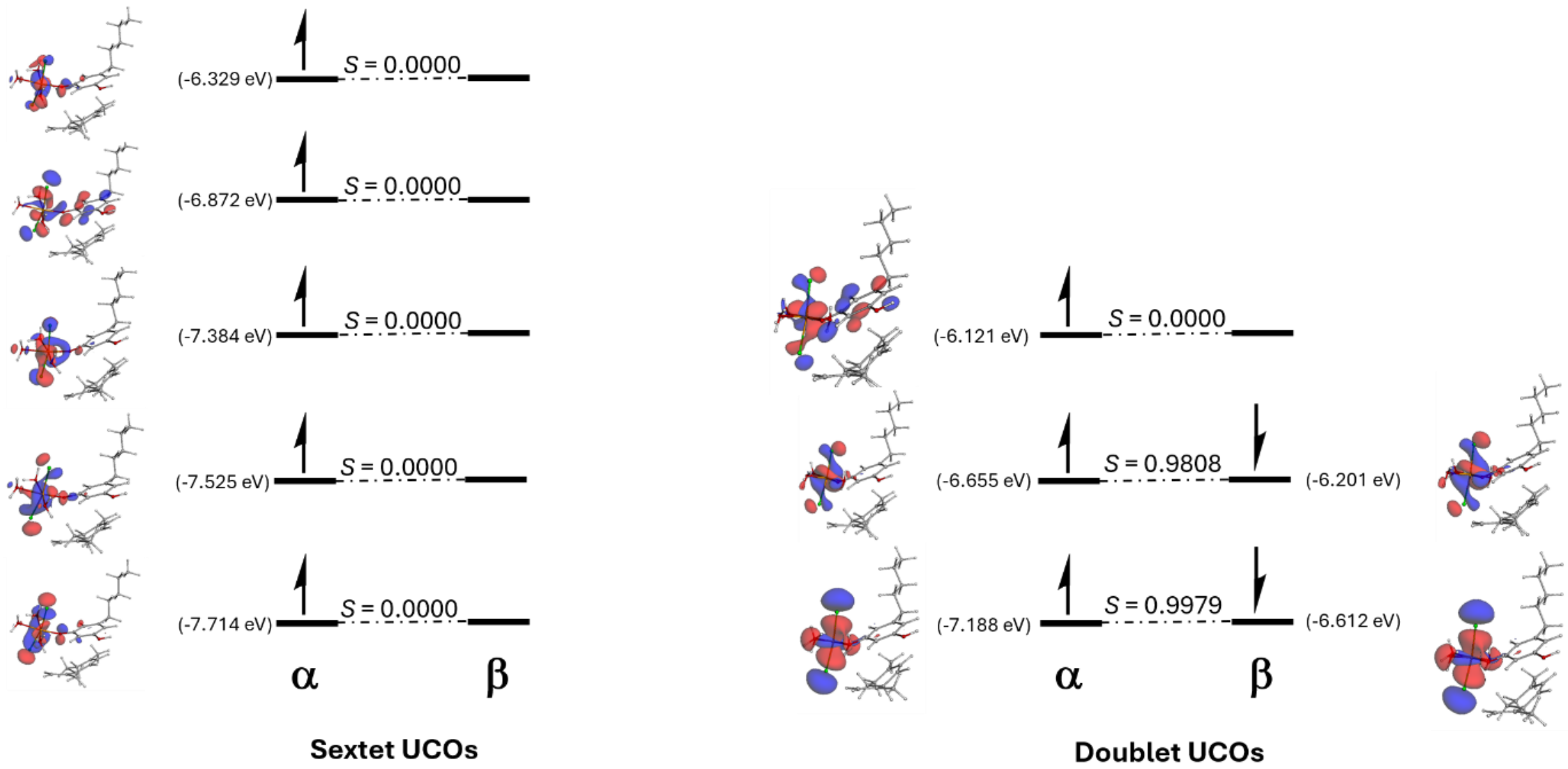

Figure S25. Unrestricted Corresponding Orbital (UCO) diagrams for the optimized pre-reactive π-complexes in the ground sextet and doublet states. Energy levels (in eV) and spatial overlap integrals (*S*) between the α and β channels are provided for the frontier metal *d*-band. In the sextet state (left), the five unpaired electrons represent pure, fully stabilized magnetic density (*S* = 0.0000). In the doublet state (right), the forced pairing of four electrons results in one isolated radical (*S* = 0.0000) and two spatial orbitals with high spatial overlap (*S* = 0.9808 and *S* = 0.9979). In contrast to the quartet state, these near-unity overlap values demonstrate that the doublet minimizes intra-orbital Pauli repulsion; however, this stable electronic pairing is only achieved through a severe structural distortion (asymmetric π-locking) that imposes the nuclear reorganization penalty discussed in the text.

6.1.5. Regioselectivity Analysis.

Proceeding exclusively on the kinetically accessible sextet manifold, computational analysis of the independent pathways towards each of the expected compounds revealed the formation of π-complexes during the approaching step. Shape comparison of both energy profiles exhibited that while the NORM-C5 (**1**) channel exhibited a shallow minimum located at 2.85 Å, the ABN-C5 (**20**) channel presented a plateau between 3.27 and 2.74 Å.

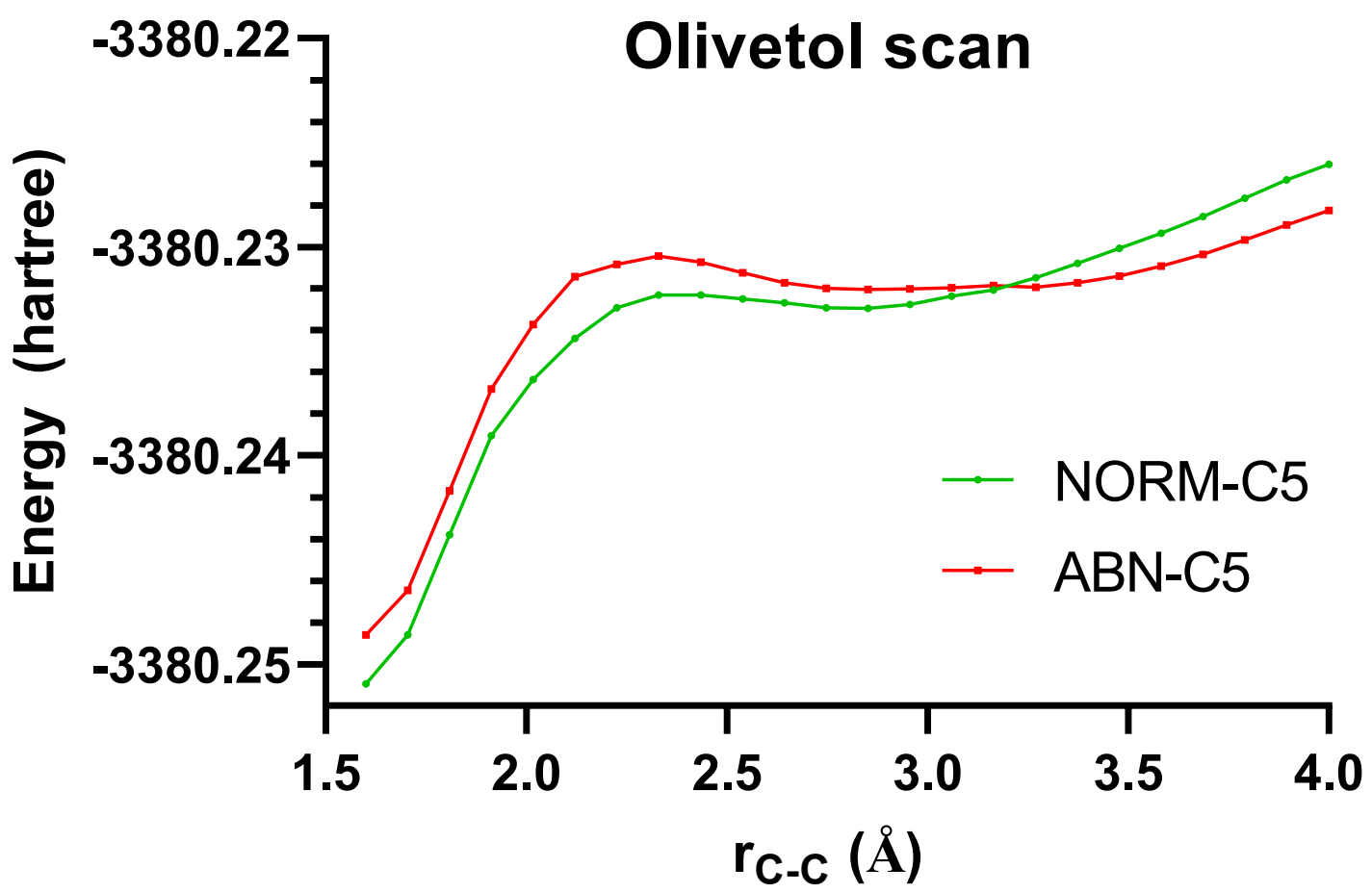

Figure S26. Energy vs C-C distance scans for the approach of the olivetol-iron complex to the cation in the normal (green) and abnormal (orange) trajectories. The normal trajectory shows a single and shallow minimum at 2.85 Å, while the abnormal trajectory presents a plateau.

Analysis of the pre-transition state π-complexes for the reaction involving olivetol. In accordance with the established unified continuum of electrophilic aromatic substitution, pre-reaction encounter complexes can exhibit a wide range of topological profiles ranging from distinct intermediates to shallow minima.[15] This topological difference suggests that in the NORM-C5 (**1** ***π-complex***) trajectory, reactants are easily pre-organized in the reactive orientation due to the lack of steric hindrance. In contrast, the steric hindrance presented by the interaction between the pentyl chain and the isopropylene group in the ABN-C5 (**20**) trajectory prevents this structural pre-organization.

According to the Activation Strain Model developed by Bickelhaupt and Houk,[16] this steric bottleneck is a physical manifestation of Pauli repulsion. In the abnormal pathway, the geometric constraints force the occupied, closed-shell electron clouds of the fragments into severe spatial overlap. Consequently, the electron density must be extensively rearranged in this interfacial region to prevent the high-energy Pauli clashes. This physical reorganization was visually confirmed by the emergence of multiple, heavily mixed frontier orbitals (HOMO through HOMO-2). As a result, the reactants must "roam" across the potential energy surface until they find the orientation that balances the attractive cation-donation interaction against this intense four-electron destabilization[8,17]; visually, this effect resulted in the observed plateau in the potential energy scan.

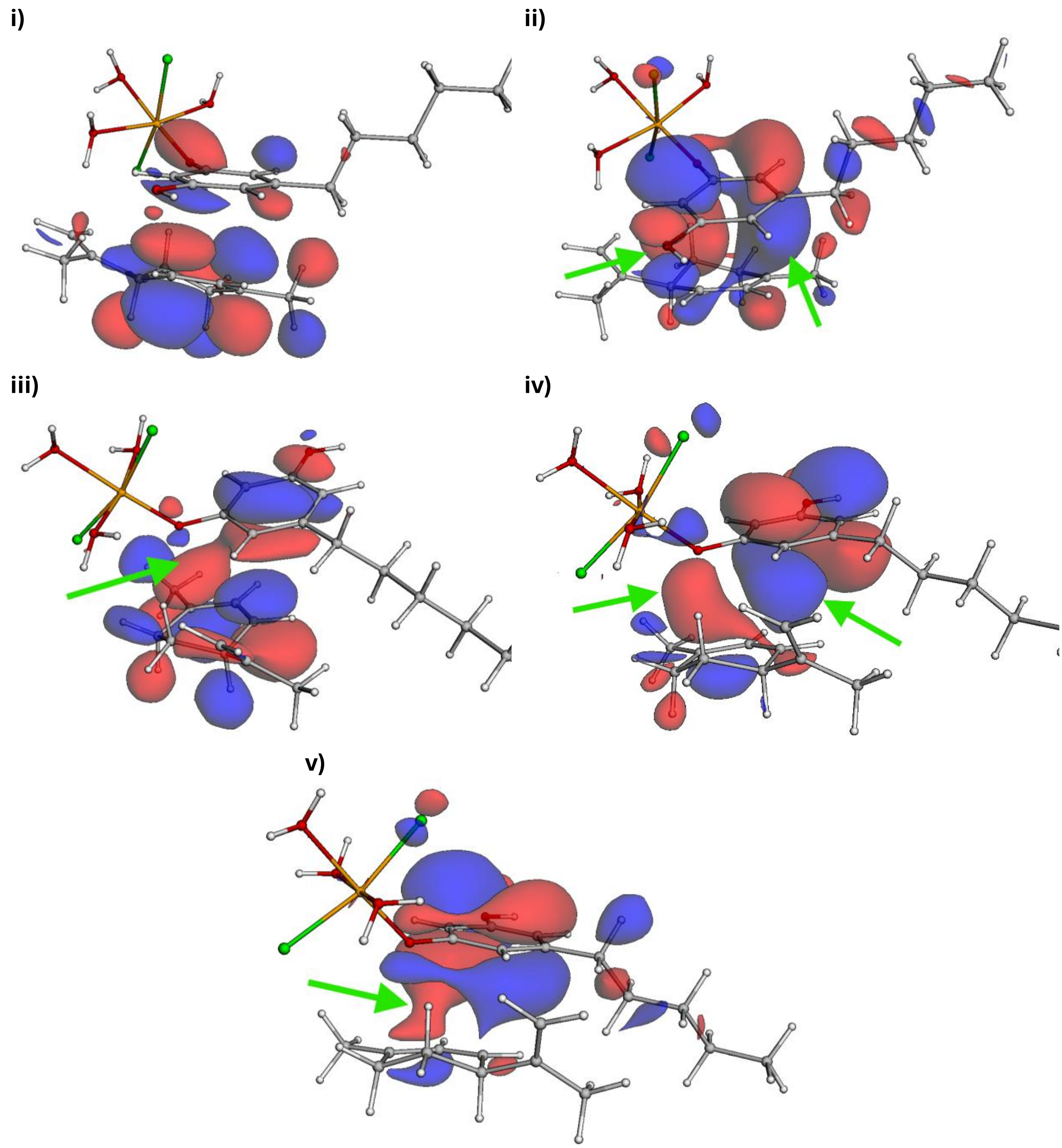

Figure S27. Orbitals for the π-complex located at 2.85 Å for the NORM-C5 (**1**) trajectory that show stabilizing interactions. i) HOMO, ii) HOMO-2. Green arrows mark the attractive interaction between the resorcinol π-system and the terpene cation. The next set of images are the orbitals of the π-complex located at 2.85 Å for the ABN-C5 (**20**) trajectory. iii) HOMO, iv) HOMO-1, v) HOMO-2. The green arrows indicate interfacial regions of high-energy occupied-occupied overlap between the resorcinol π-system and the terpene cation framework. Because these are closed-shell interactions, they do not represent stabilizing bonds; rather, they highlight the primary coordinates of Pauli repulsion where electron density must be extensively rearranged to satisfy the Pauli exclusion principle.

In order to evaluate the energetics of these interactions, standard thermochemical calculations were conducted together with quasi-RRHO corrections applied. However, the highly fluxional nature of the transition metal complex, specifically the low-frequency rotations of the coordinated water ligands and flexible aliphatic chains, introduced severe artificial conformational noise into the entropic calculations. As previously documented in computational modeling of bulky and transition-metal systems[9], the standard harmonic oscillator

approximation poorly describes large-amplitude, low-frequency vibrations. Crucially, while Grimme's quasi-RRHO scheme effectively mitigated the mathematical divergence of these low frequencies, it could not resolve the underlying physical limitation of single-structure static thermodynamics: the quasi-RRHO model still calculates the partition function based on a single geometric snapshot. Because the water ligands and flexible chains possess multiple accessible rotameric states at 298.15 K, the transition state optimizations inevitably locked the spectator groups into slightly different orientations for the normal and abnormal transition states. The quasi-RRHO correction mathematically translated these rotameric profiles into artificial free energy deviations (often 1-2 kcal/mol). This persistent noise easily masked the sub-kcal/mol intrinsic chemical differences, which could only be resolved via computationally expensive conformational averaging.[9] Therefore, to rigorously isolate the intrinsic steric penalty imposed by the pentyl chain without interference from the artificial entropic noise of rotating spectator ligands, the relative electronic energies ($\Delta E^{\ddagger}$) evaluated at the definitive B3LYP-D4/def2-TZVPP level were employed as the primary metric for the energy analyses.

Analysis of the transition states for both trajectories in the reaction involving olivetol. While macroscopic steric bulk is classically expected to impose kinetic penalties, recent studies in transition-metal catalysis demonstrate that severe steric confinement can paradoxically accelerate reactions by forcing the engagement of cooperative, secondary-sphere noncovalent interactions[18,19]. This precise dynamic governs the native olivetol system. Orbital analysis of the unhindered NORM-C5 transition state (**1 TS**) revealed that the pentyl chain directs its steric bulk away from the interfacial gap, successfully avoiding spatial overlap between the frontier orbitals (HOMO through HOMO-3) to minimize Pauli repulsion. However, the macroscopic bulk of the chain simultaneously acts as a steric wall that severely confines the overall conformational landscape. Having already paid the entropic penalty of this spatial restriction, the reactants are effectively funneled into a highly specific, pre-organized geometry. This forced alignment unlocks a secondary stabilizing interaction: as observed in the HOMO-4 orbital, one of the iron-coordinated water ligands acts as an electronic anchor, forming a non-covalent O-H hydrogen bond with the isopropenyl group. Because the steric confinement of the pentyl chain pre-organizes the system to utilize this attractive noncovalent interaction, the normal trajectory proceeds through a highly accessible intrinsic barrier (1.03 kcal/mol)

i)

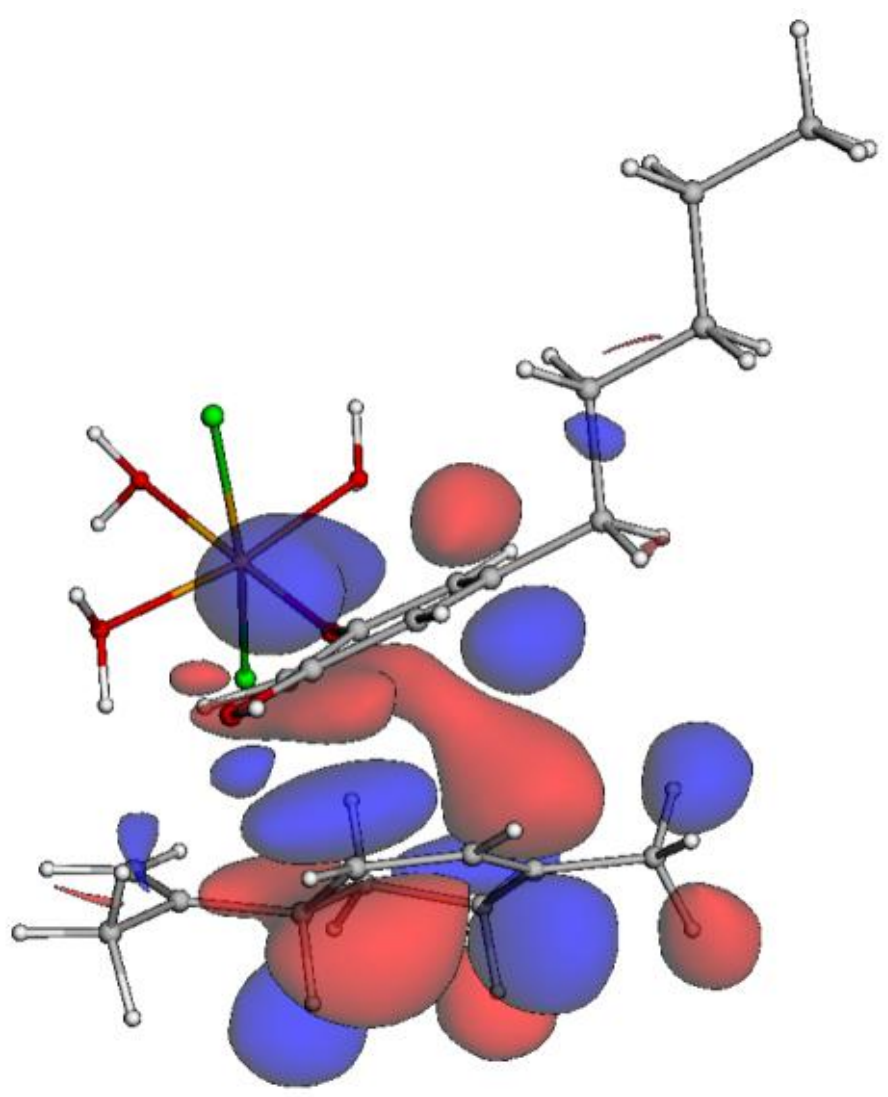

ii)

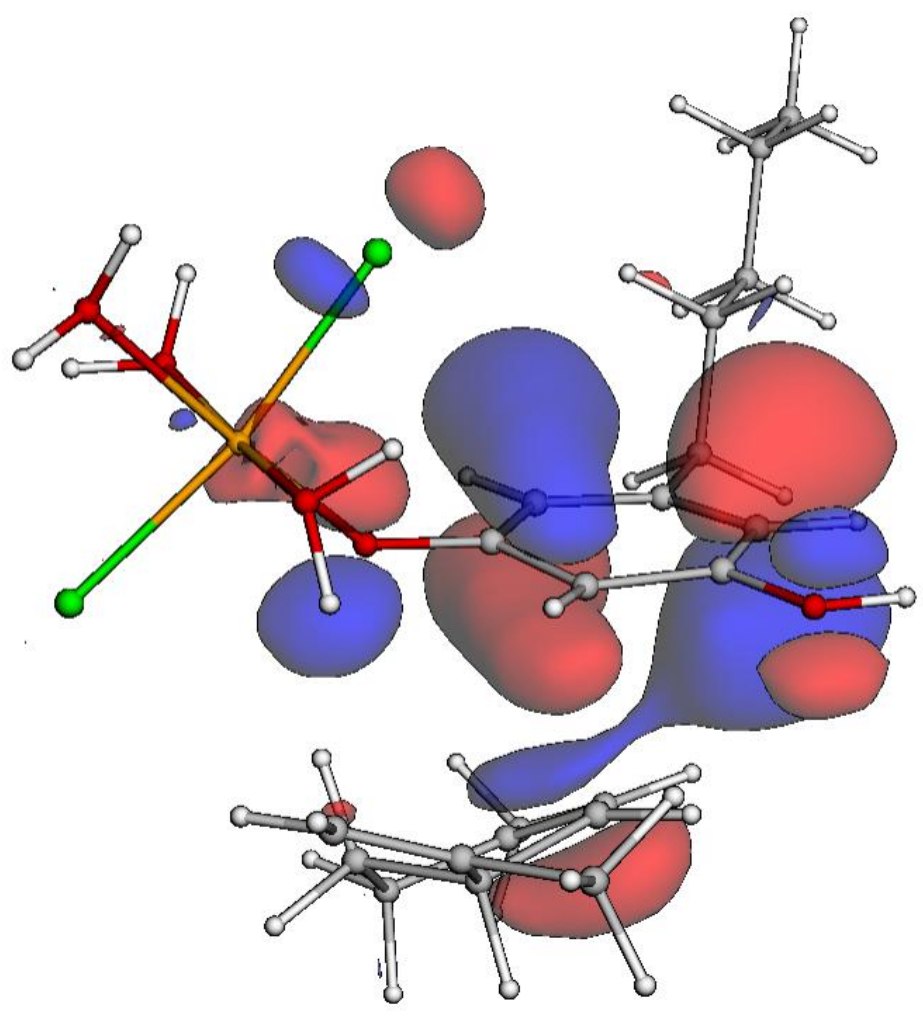

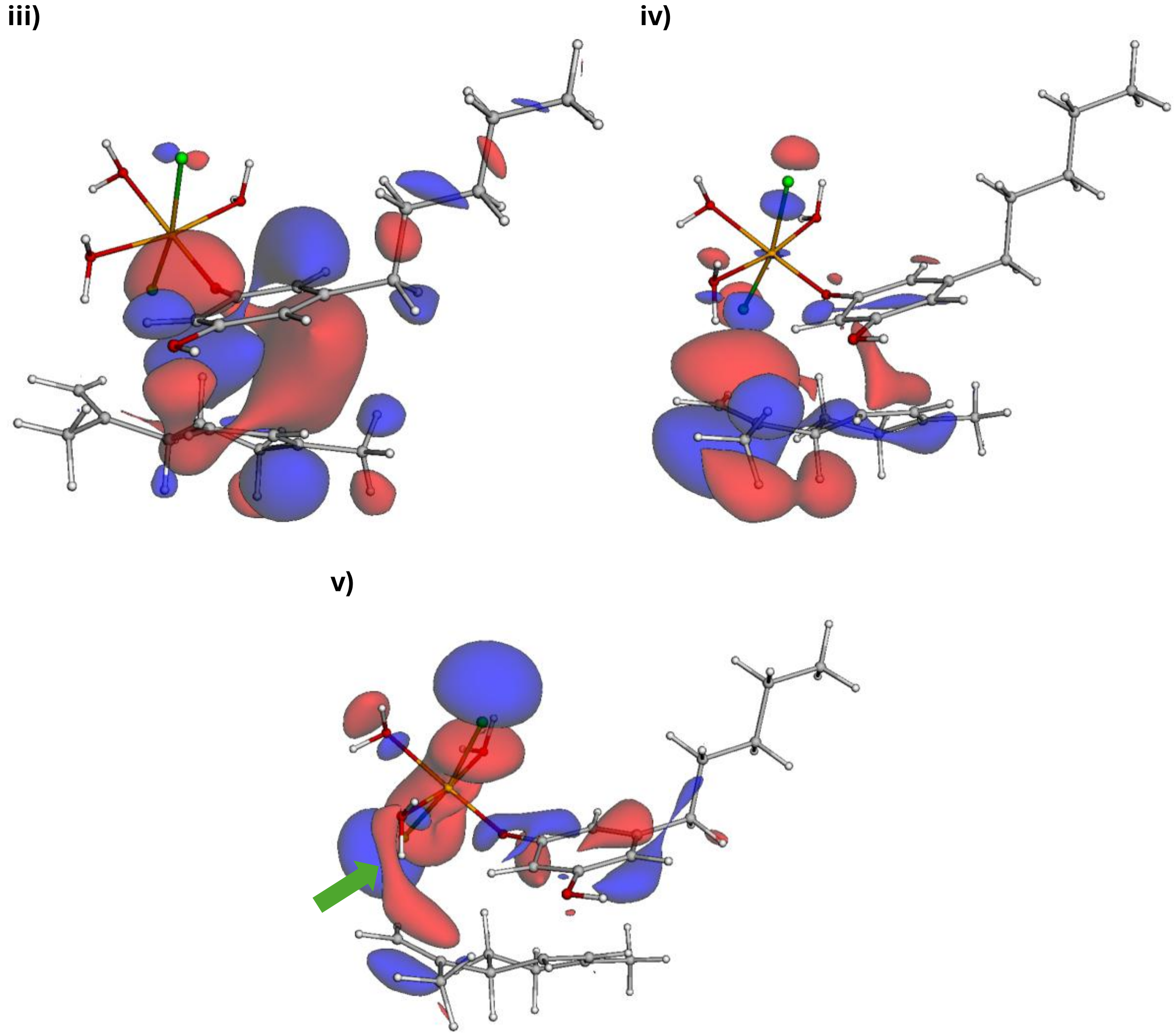

Figure S28. Orbitals for the transition state of the normal system. i) HOMO, ii) HOMO-1, iii) HOMO-2, iv) HOMO-3, v) HOMO-4. All orbitals show interactions between the resorcinol and the terpene cation orbitals. HOMO to HOMO-3 reveal that no occupied-occupied overlapping occurs between them, thus keeping the Pauli clashes to a minimum. HOMO-4 reveals the formation of an H-bond between a water molecule of the iron complex and the alkene present in the isopropenyl group, which has stabilizing character (green arrow).

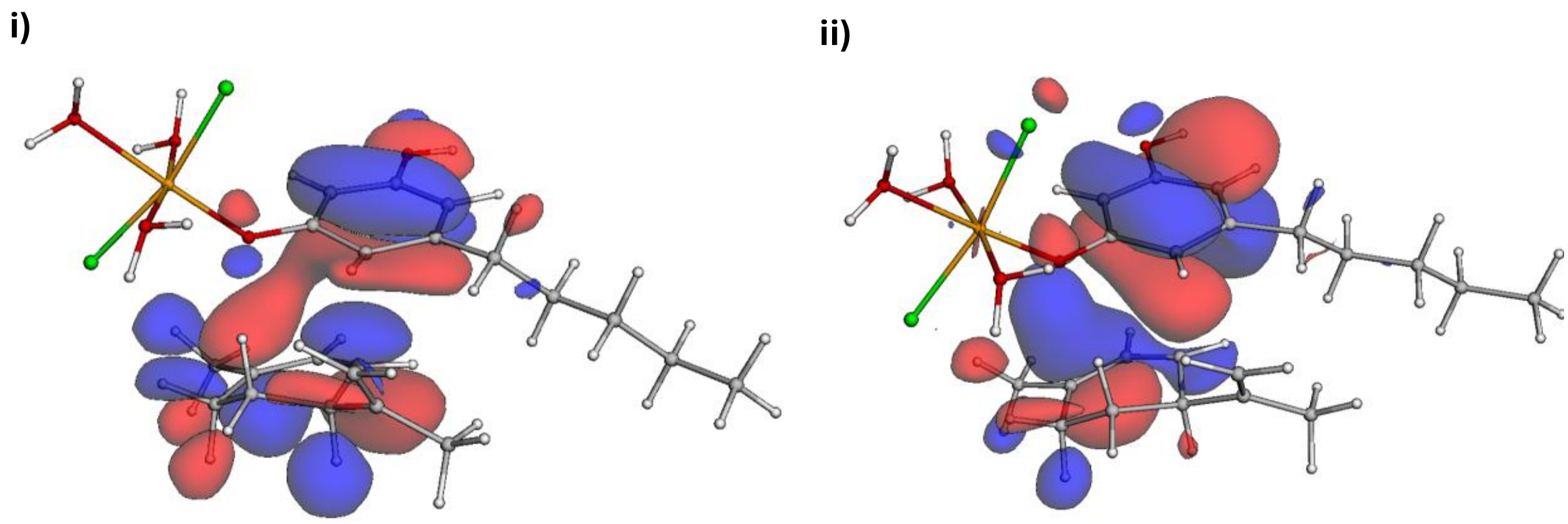

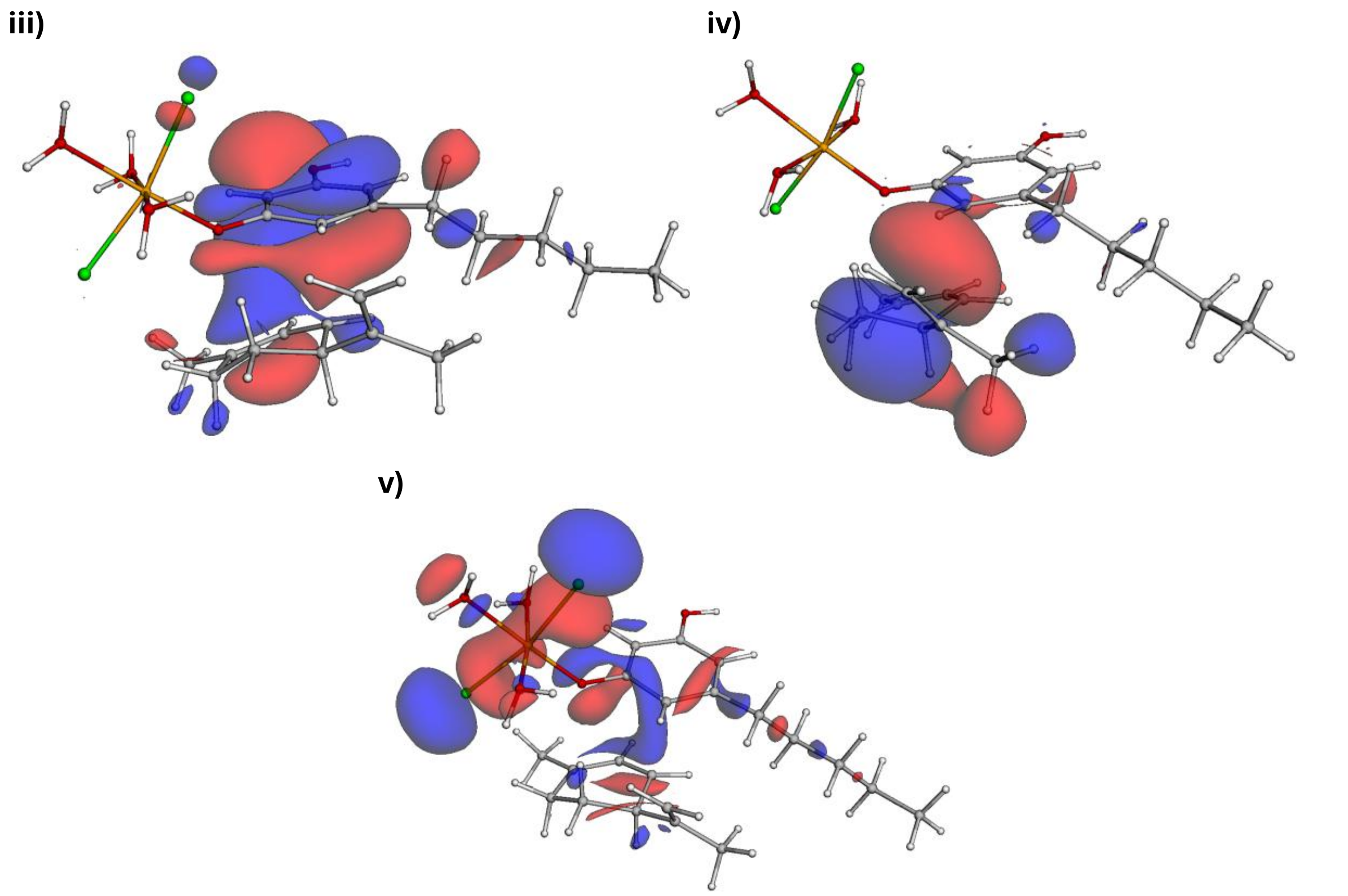

Figure S29. Orbitals for the transition state of the abnormal system. i) HOMO, ii) HOMO-2, iii) HOMO-3, iv) HOMO-4. HOMO to HOMO-3 reveal the electronic congestion in the intermolecular gap between the resorcinol ring and the terpene cation, causing Pauli clashes between the electron densities of each orbital. HOMO-4 shows the funneling of electronic density from the phenolic oxygen bound to the iron towards the reactive carbon in the terpene cation.

On the other hand, analysis of the frontier orbitals for the abnormal transition state further confirmed the steric congestion via the Pauli repulsions previously observed in the π-complex. In the transition state, the pentyl chain was directed inward, preventing the orbital localization found in the normal transition state and causing severe Pauli repulsions. As visually evidenced by the HOMO through HOMO-3 orbitals, the electron density had to be distributed into a high-energy configuration that scrambled across the interfacial gap to resolve the closed-shell clashes. Furthermore, this steric occlusion physically prevented the approach of the water ligand, permanently blocking the highly stabilizing O-H anchor enjoyed by the normal pathway, which was observed by the intact π-system from the isopropylene group in HOMO-3 orbital. This strained transition state resorted to a secondary compensatory delocalization channel, funneling electron density directly through the coordinated phenoxide oxygen to stabilize the incipient carbon-carbon bond (HOMO-4 orbital). This density funneling is characteristic of secondary orbital interactions and ancillary ligand π-donation frequently documented in transition-metal catalysis[20]. In the context of the Activation Strain Model,[11] this secondary delocalization represents the system's attempt to maximize favorable orbital interaction energies to offset the massive thermodynamic penalty imposed by the persistent Pauli clashes. Overall, these interactions explained the larger intrinsic barrier for the abnormal trajectory ($\Delta E_{int}^{\ddagger}$ = 1.87 kcal/mol).

Analysis of the imaginary frequencies for both transition states (reaction involving olivetol). The stark electronic divergence of the two pathways is directly manifested in the topographic curvature of their respective saddle points, as quantified by their imaginary frequencies. For the sterically congested ABN-C5 (**20**) trajectory, the calculated frequency was larger (-197 cm^{-1}) than for the NORM-C5 (**1** TS) (-142 cm^{-1}), which characterizes a more rigid, and 'tight' transition state. The sharper curvature in the ABN-C5 (**20 TS**) reflected the severe geometric penalty of the pathway: the system must transition from a flat, disorganized pre-reaction plateau, exhibiting fluxional character reminiscent of 'roaming' dynamics, where intermediates lack a directed reaction vector[17], into a highly compressed bottleneck, as evidenced from the Pauli repulsions analyzed previously. To achieve bond formation, the reacting fragments must be forced against the repulsive steric wall imposed by the different orientation of the pentyl chain, resulting in a highly restricted, higher energy saddle point.

Conversely, the unhindered NORM-C5 (**1**) trajectory exhibited a significantly smaller imaginary frequency (-142 cm^{-1}), indicative of a broader, 'loose' transition state. Because the non-covalent O-H interaction acted as a persistent conformational anchor from the π-complex onward, the NORM-C5 (**1** TS) system is highly pre-organized: it does not compress itself to escape a fluxional roaming state; instead, it seamlessly funnels through a conformationally relaxed, shallow reaction pathway towards the Wheland intermediate through a transition state free of forced closed-shell interpenetration. In summary, this vibrational analysis bridges the electronic and thermodynamic data: the abnormal trajectory pays a massive kinetic penalty to force a fluxional intermediate through a sterically compressed wall, whereas the normal pathway smoothly navigates a relaxed, pre-organized pass.

Analysis of the pre-transition state π-complexes for the reaction involving olivetol. When analyzing the normal and abnormal pathways for the C5-methyl derivatives, the potential energy surfaces were found to be extremely close in energy and shape, with both exhibiting a shallow π-complex at 2.74 Å, with no plateaus that show a roaming mechanism and similar energies for the transition states, albeit with a slight preference for the normal pathway.

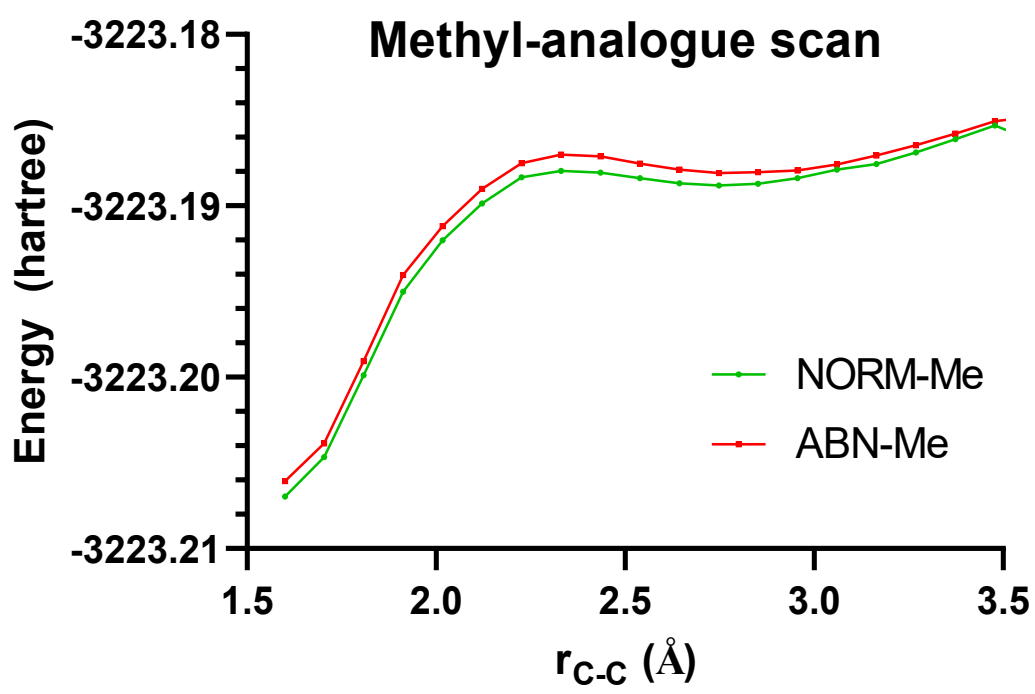

Figure S30. Energy vs C-C distance scans for the approach of the methyl analogue-iron complex to the cation in the normal (green) and abnormal (orange) trajectories. Both trajectories exhibit the same shape with a single and shallow minimum at 2.74 Å.

Analysis of the pre-transition state π-complexes for the reaction involving orcinol.

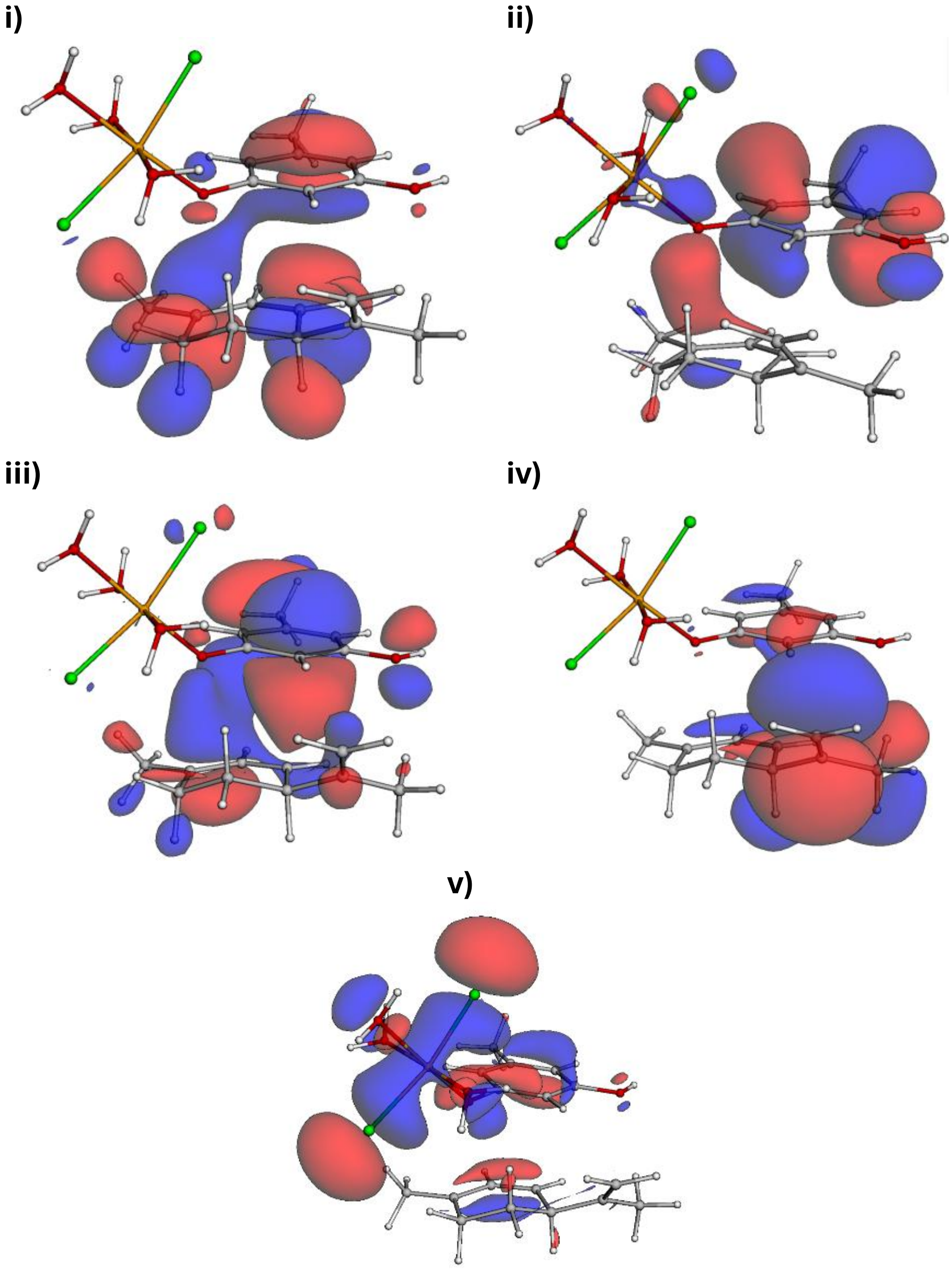

Figure S31. Orbitals for the π-complex located at 2.85 Å for the NORM-Methyl π-complex (**32** π-complex) trajectory that show stabilizing interactions. i) HOMO, ii) HOMO-1, iii) HOMO-2, iv) HOMO-3, v) HOMO-4.

Orbital analysis of the NORM-Me π-complex (**32** π-complex) provided a critical baseline for the inherent electronic state of the system in the absence of long-range steric effects. As shown in the frontier orbitals (HOMO through HOMO-2), the electron density remained perfectly localized and discrete upon their respective fragments. The coordinated resorcinol nucleophile maintained symmetric, unperturbed π-clouds, while the terpene cation framework exhibited no evidence of the density scrambling or forced scenarios where the electronic density rearranged to avoid steric overlap, as was observed in the hindered olivetol trajectories. Crucially, the HOMO-3 revealed that at a separation of 2.8 Å, the system has not yet entered the covalent regime; the resorcinol ring and the cation core remained electronically autonomous, held together in a van der Waals well primarily through baseline dispersion forces. Furthermore, the HOMO-4 orbital confirmed that the iron-coordinated water ligands were not yet engaged in secondary interactions with the substrate. This established the NORM-Me (**32**) π-complex as a

starting point where the lack of spatial interpenetration of the orbitals ensured a minimal Pauli repulsion baseline prior to the climb toward the transition state.

i) **ii)** **iii)** **iv)** **v)**

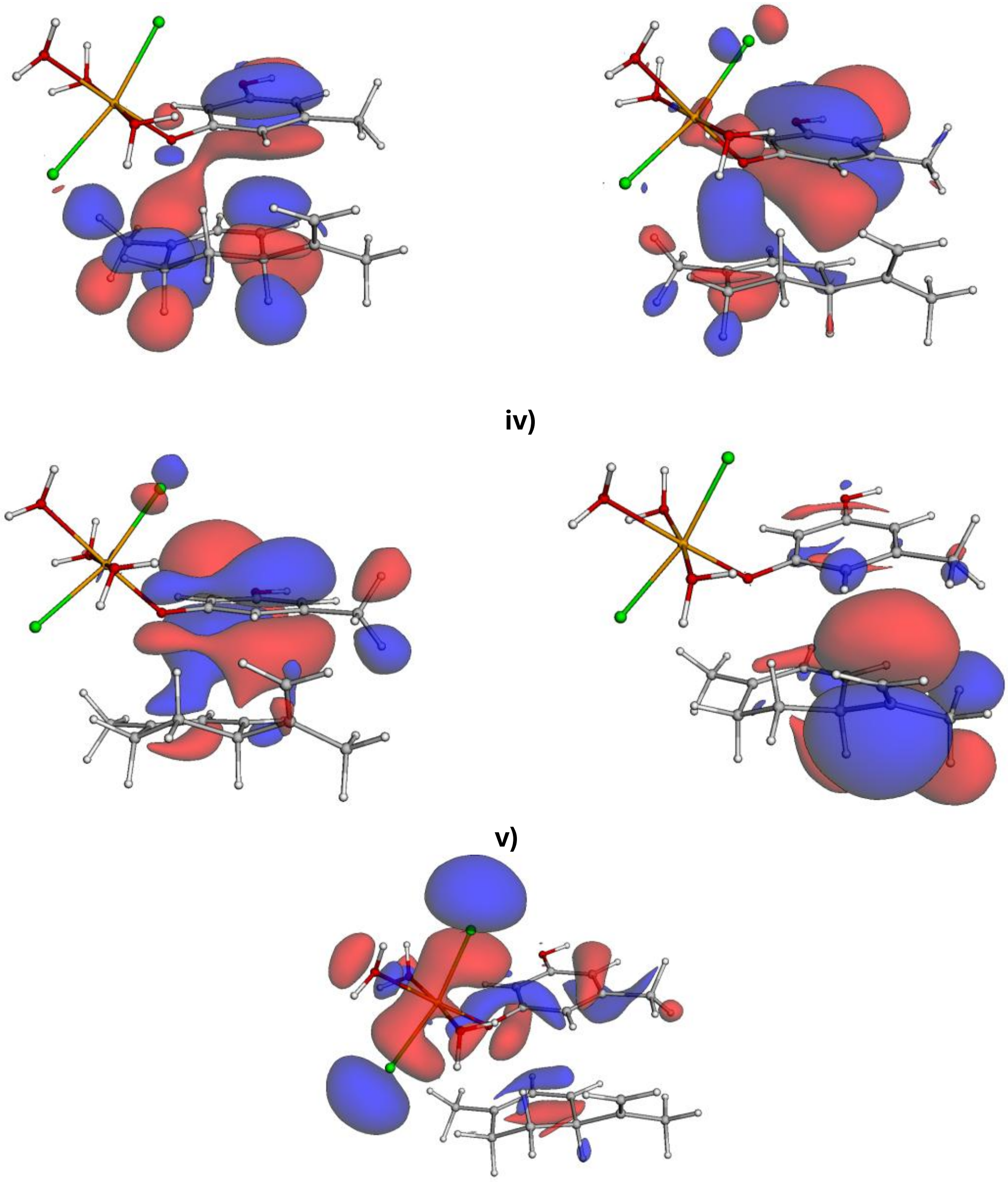

Figure S32. Orbitals for the π-complex located at 2.85 Å for the ABN-Methyl (**33**) trajectory that show stabilizing interactions. i) HOMO, ii) HOMO-1, iii) HOMO-2, iv) HOMO-3, v) HOMO-4.

Analysis of the transition states for the reaction involving orcinol.

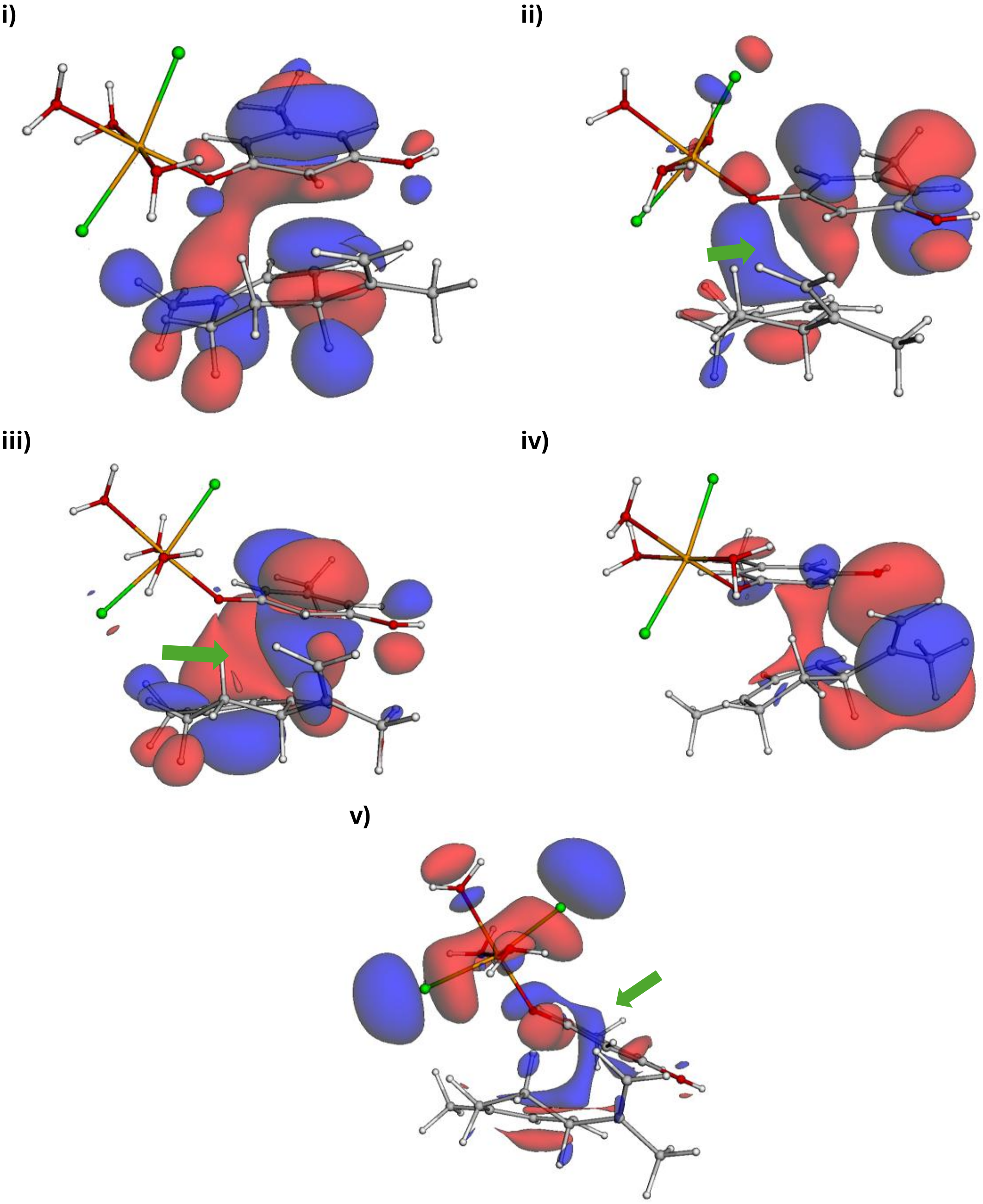

Figure S33. Orbitals for the transition state at 2.33 Å for the NORM-Methyl (**32**) trajectory. i) HOMO, ii) HOMO-1, iii) HOMO-2, iv) HOMO-3, v) HOMO-4. HOMO-1 and HOMO-2 reveal the stabilization by the direct overlapping of the electron density from the iron-phenolic oxygen and the tertiary carbon atom on the terpene cation. HOMO-4 showed a more pronounced lobe over the reactive carbons, due to the electron density funneling from the phenol-iron oxygen towards the terpene cation.

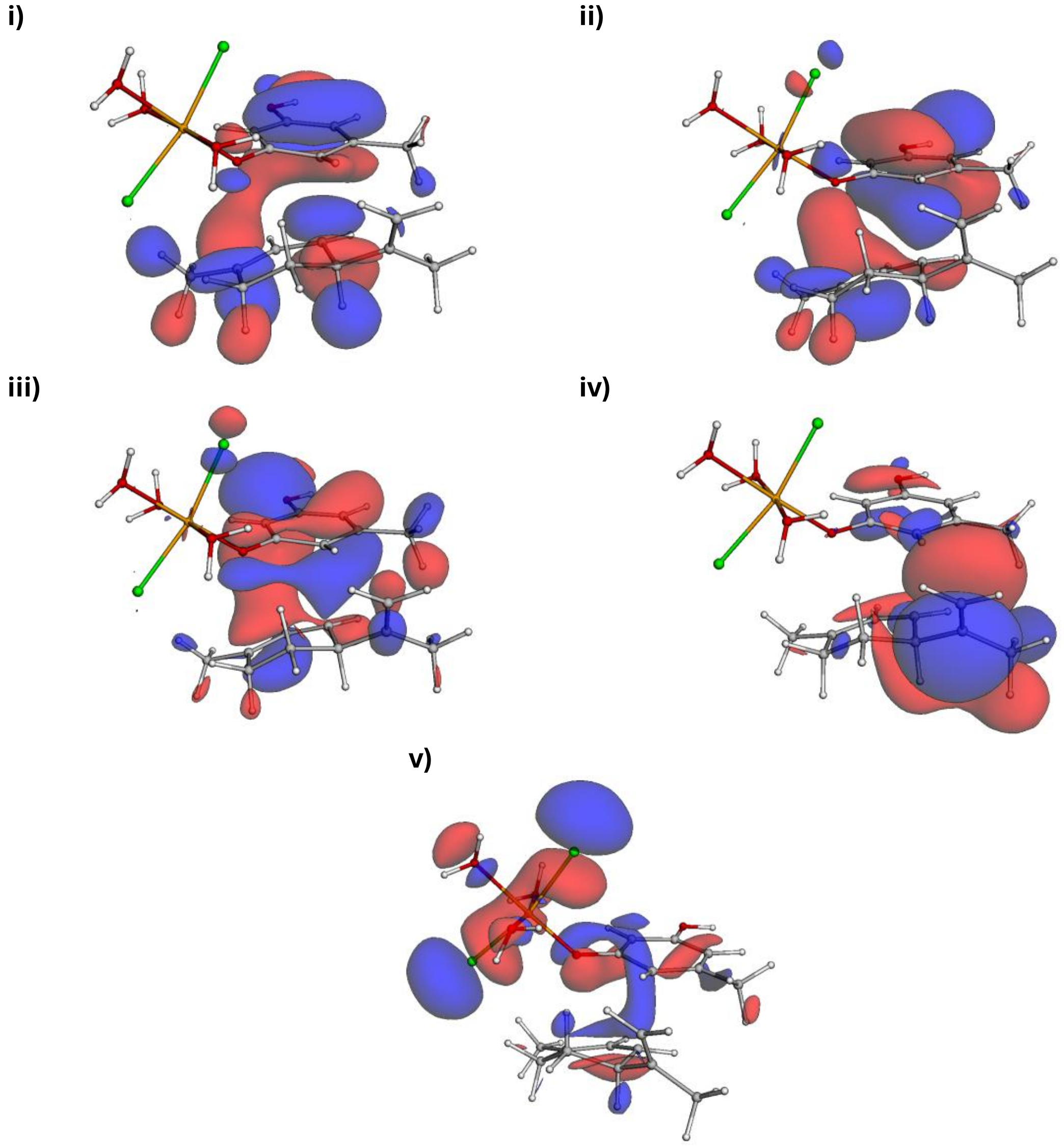

Figure S34. Orbitals for the transition state at 2.33 Å for the ABN-Methyl (**33**) trajectory. Comparison of these orbitals with those from the NORM-Methyl (**32**) transition state reveals the similarity in the interacting orbitals, thus pointing towards a similar chemical reactivity.

Evaluation of the truncated NORM-Me (**32**) and ABN-Me (**33**) transition states revealed a state of electronic isomorphism. In the absence of the elongated aliphatic pentyl chain, the spatial symmetry of the resorcinol core dominated the bimolecular interaction. The incoming terpene cation approached both the normal and abnormal positions via geometrically identical trajectories, governed solely by the primary C–C bond formation and stabilizing non-covalent cation-π interactions. Notably, the orbital maps for both transition states displayed unperturbed, highly delocalized electron densities in the inter-fragment region. Neither attack exhibited the orbital spatial confinement, density depletion, or forced geometric distortion characteristic of steric hindrance. This unhindered, symmetric baseline stood in contrast to the pronounced asymmetry observed in the native olivetol system. In the ABN-C5 transition state (**20 TS**), the pentyl chain forced closed-shell electron clouds into direct spatial overlap, inducing a localized spike in Pauli repulsion that exacted a severe kinetic penalty ($\Delta\Delta E_{CH}^{\ddagger}$ = 1.25 kcal/mol). However, truncating the aliphatic tail to a simple methyl group abolished this Pauli clash. Without the steric

bulk of the pentyl chain, the competing regioisomeric attacks became kinetically degenerate: the NORM-Me (**32**) trajectory proceeded through an accessible intrinsic barrier ($\Delta E_{int}^{‡}$ = 1.35 kcal/mol), while the ABN-Me (**33**) trajectory navigated a nearly identical intrinsic barrier ($\Delta E_{int}^{‡}$ = 1.56 kcal/mol). This kinetic gap between the two truncated pathways ($\Delta\Delta E_{CH}^{‡}$ = 0.17 kcal/mol) fell entirely within the sub-kcal/mol error margin (typically ~0.5 kcal/mol) inherent to error-canceling isodesmic comparisons in modern DFT,[21] confirming true kinetic degeneracy rather than any inherent electronic bias. This energetic convergence physically confirmed that the metal-coordinated resorcinol core possessed no intrinsic electronic bias, proving that the observed regioselectivity was unequivocally dictated by the steric hindrance of the pentyl tail.

Analysis of the imaginary frequencies for the transition states from the reaction involving orcinol. The curvature of the saddle points for the methyl analogues was quantified, as in the case of the olivetol derivatives, by their imaginary frequencies. The normal transition state had an imaginary frequency of -164 cm^{-1}, while for the abnormal saddle point the found frequency was -179 cm^{-1}. However, all geometry optimizations and frequency calculations were performed using the RIJCOSX approximation and the default integration grid (DEFGRID2) to manage the computational cost of the transition metal complex. Because of this, the observed variance fell entirely within the established numerical noise floor for DFT hessian evaluations. As noted in the ORCA 6.1.1 documentation, the calculation of analytical second derivatives, especially when coupled with the auxiliary grids required for the RIJCOSX approximation, routinely introduces frequency fluctuations in the 10–50 cm^{-1} interval. As this 15 cm^{-1} difference fell within the fluctuation range, it was attributed to integration grid artifacts, confirming that the two pathways traversed kinetically and topologically equivalent saddle points.

System	Trajectory	Intrinsic barrier, $\Delta E_{int}^{‡}$ (kcal/mol)	Curtin-Hammett gap, $\Delta\Delta E_{CH}^{‡}$ (kcal/mol)	Imaginary frequency (cm^{-1})
**Olivetol (C5)**	NORM-C5 (**1**)	1.03	0.00	-142
	ABN-C5 (**20**)	1.87	+1.25	-197
**Truncated (Methyl)**	NORM-Me (**32**)	1.35	+0.17	-164
	ABN-Me (**33**)	1.56	0.00	-179

Table S3. Topological and electronic comparison of the normal and abnormal transition states for both the olivetol (C5) and truncated (Me) systems. Intrinsic electronic barriers ($\Delta E_{int}^{‡}$) are calculated relative to their respective π-complex minima. The Curtin-Hammett kinetic gap ($\Delta\Delta E_{CH}^{‡}$) represents the absolute energy difference between competing transition states. Geometries and imaginary frequencies were obtained at the RIJCOSX-B3LYP-D4/def2-SVP level, with final single-point electronic energies evaluated at the B3LYP-D4/def2-TZVPP level.

Because the pre-reaction π-complexes are non-covalently bound and exist in rapid pre-equilibrium, the ultimate regioselectivity of the reaction is governed by Curtin-Hammett kinetics. As summarized in Table 1, comparing the absolute electronic energies of the competing saddle points ($\Delta\Delta E_{CH}^{‡}$) revealed a 1.25 kcal/mol preference for the unhindered NORM-C5 trajectory. Conversely, truncation of the pentyl chain completely suppressed this difference, resulting in a negligible -0.17 kcal/mol gap between the two methyl transition states. This confirmed that the observed kinetic bias was driven entirely by the macroscopic steric bulk of the pentyl tail, rather than any intrinsic electronic asymmetry in the resorcinol core.

Data of the structures of interest (optimized at B3LYP-D4/def2-SVP CPCM(dichloromethane)):

Scan data for spin-states trajectories:

r_{C-C}	NORM-C5 Sextet Energy (a.u.)	NORM-C5 Quartet Energy (a.u.)	NORM-C5 Doublet Energy (a.u.)
1.6	-3380.25095724	-3380.2271569	-3380.21793625
1.704348	-3380.24860377	-3380.2247997	-3380.21512459
1.808696	-3380.24378880	-3380.2201471	-3380.20982647
1.913043	-3380.23904642	-3380.2158432	-3380.20671753
2.017391	-3380.23635376	-3380.2135571	-3380.20391134
2.121739	-3380.23437654	-3380.2119075	-3380.20187691
2.226087	-3380.23291299	-3380.2106648	-3380.20035908
2.330435	-3380.23231766	-3380.2102295	-3380.19970601
2.434783	-3380.23229547	-3380.2103217	-3380.19965235
2.53913	-3380.23249015	-3380.2105789	-3380.19993572
2.643478	-3380.23268860	-3380.2107566	-3380.20029337
2.747826	-3380.23291360	-3380.2107785	-3380.20058687
2.852174	-3380.23295389	-3380.2105683	-3380.20072680
2.956522	-3380.23276248	-3380.2101179	-3380.20062132
3.06087	-3380.23236858	-3380.2094589	-3380.20043390
3.165217	-3380.23206047	-3380.2086257	-3380.20005864
3.269565	-3380.23147759	-3380.2076606	-3380.19959936
3.373913	-3380.23078767	-3380.2065989	-3380.19911587
3.478261	-3380.23007292	-3380.205564	-3380.19856441
3.582609	-3380.22933435	-3380.204471	-3380.19793257
3.686957	-3380.22853743	-3380.203281	-3380.19722552
3.791304	-3380.22766017	-3380.203452	-3380.19641359
3.895652	-3380.22678609	-3380.202535	-3380.19554142
4	-3380.22604519	-3380.203024	-3380.19519373

r_{C-C}	ABN-C5 Sextet Energy (a.u.)	ABN-C5 Quartet Energy (a.u.)	ABN-C5 Doublet Energy (a.u.)
1.6	-3380.24859383	-3380.22416278	-3380.21678809
1.704348	-3380.24645455	-3380.22184689	-3380.21455869
1.808696	-3380.24168150	-3380.21859994	-3380.20968508
1.913043	-3380.23681197	-3380.21356115	-3380.20450625
2.017391	-3380.23371483	-3380.21043442	-3380.20110452
2.121739	-3380.23141542	-3380.20823960	-3380.19846040
2.226087	-3380.23083307	-3380.20792479	-3380.19647692
2.330435	-3380.23045187	-3380.20787515	-3380.19555694
2.434783	-3380.23072936	-3380.20844456	-3380.19915469
2.53913	-3380.23125257	-3380.20925229	-3380.19960631
2.643478	-3380.23173141	-3380.20996841	-3380.20001749
2.747826	-3380.23198776	-3380.21045212	-3380.20023406

2.852174	-3380.23204195	-3380.21065879	-3380.20018964
2.956522	-3380.23201418	-3380.21071209	-3380.19987754
3.06087	-3380.23196387	-3380.21070693	-3380.19935621
3.165217	-3380.23184053	-3380.21066278	-3380.19869443
3.269565	-3380.23193146	-3380.21055307	-3380.19792892
3.373913	-3380.23173127	-3380.2103234	-3380.19772678
3.478261	-3380.23139186	-3380.2100456	-3380.19723815
3.582609	-3380.23093286	-3380.2096485	-3380.19673969
3.686957	-3380.23034778	-3380.2091127	-3380.19624403
3.791304	-3380.22966314	-3380.2084477	-3380.19573324
3.895652	-3380.22895075	-3380.2077370	-3380.19526850
4	-3380.22825469	-3380.2070699	-3380.19487242

Scan data for reaction trajectories:

r_{C-C} (Å)	Energy (a.u.)			
	**NORM-C5 (1)**	**ABN-C5 (20)**	**NORM-Methyl (32)**	**ABN-Methyl (33)**
1.6	-3380.25095724	-3380.24859383	-3223.20697	-3223.206073
1.704348	-3380.24860377	-3380.24645455	-3223.204661	-3223.20387
1.808696	-3380.24378880	-3380.24168150	-3223.199885	-3223.199055
1.913043	-3380.23904642	-3380.23681197	-3223.195019	-3223.194034
2.017391	-3380.23635376	-3380.23371483	-3223.192023	-3223.191183
2.121739	-3380.23437654	-3380.23141542	-3223.189873	-3223.189004
2.226087	-3380.23291299	-3380.23083307	-3223.188347	-3223.187503
2.330435	-3380.23231766	-3380.23045187	-3223.18797	-3223.187005
2.434783	-3380.23229547	-3380.23072936	-3223.188057	-3223.187122
2.53913	-3380.23249015	-3380.23125257	-3223.188398	-3223.187525
2.643478	-3380.23268860	-3380.23173141	-3223.18868	-3223.187899
2.747826	-3380.23291360	-3380.23198776	-3223.188803	-3223.188099
2.852174	-3380.23295389	-3380.23204195	-3223.188706	-3223.188034
2.956522	-3380.23276248	-3380.23201418	-3223.188381	-3223.187943
3.06087	-3380.23236858	-3380.23196387	-3223.187888	-3223.187578
3.165217	-3380.23206047	-3380.23184053	-3223.187568	-3223.187064
3.269565	-3380.23147759	-3380.23193146	-3223.186899	-3223.186473
3.373913	-3380.23078767	-3380.23173127	-3223.186124	-3223.185777
3.478261	-3380.23007292	-3380.23139186	-3223.185325	-3223.185058
3.582609	-3380.22933435	-3380.23093286	-3223.186249	-3223.184782
3.686957	-3380.22853743	-3380.23034778	-3223.186145	-3223.184281
3.791304	-3380.22766017	-3380.22966314	-3223.185903	-3223.183786
3.895652	-3380.22678609	-3380.22895075	-3223.189909	-3223.18329
4	-3380.22604519	-3380.22825469	-3223.189357	-3223.182823

NORM-C5 π-complex Quartet:

Charge: +1 | Multiplicity: 4 | Imaginary frequency: ---

E_{elec}(def2-TZVPP): -3381.877619 a.u.

Thermal correction to Gibbs Free Energy (quasi-RRHO) (def2-SVP): 0.472702 a.u.

Absolute Gibbs Free Energy: -3381.404917 a.u.

H	-0.90369389905571	-4.72926043742168	0.08830607802718
C	-0.76520333642925	-3.73430115244789	-0.35674619082717
C	-1.44083496837348	-2.41019739869383	-2.42085099154900
C	-1.73135467082969	-3.56969698252527	-1.53037327230441
C	0.69483175310960	-3.55765759507318	-0.78609959376329
H	-1.68322585108933	-4.45413008326060	-2.20239743569377
H	0.95534217262366	-4.39568674320828	-1.48225588763335
H	-1.00403554329817	-2.99142400887804	0.41651587505160
C	-2.53663478126220	-1.87691030922876	-3.26380361633309
H	-2.77740120888700	-3.52810644386192	-1.18764046176151
C	1.73961785182587	-3.66690358682856	0.31904394883707
C	1.40711080756239	-4.00441633031792	1.57615089570622
H	2.19035455838742	-4.15402126965365	2.32655604445447
H	0.37626827784002	-4.16070525108650	1.89937261134258
C	3.17361224658013	-3.45118467330513	-0.09002370950505
H	3.86206937393287	-3.74336266879818	0.71532942171940
H	3.37231335809801	-2.39358662030312	-0.33372823541553
H	3.42090841230065	-4.03662639219620	-0.99240433323413
C	0.87058857002350	-2.39218129324684	-1.70088762523118
H	1.88669626460618	-2.05483758794303	-1.91212883414093
C	-0.15435595977302	-1.89903376331178	-2.50322399331806
H	0.06278465670643	-1.09774104200018	-3.21001412379690
Fe	-0.92067865436667	-0.69623830030651	3.06986925745739
O	-1.02477000551726	-0.47475018251127	5.08212588831285
H	-0.32359843599239	0.07669503554114	5.46951162570671
H	-1.01597100750859	-1.33266186851825	5.54189651355274
O	-2.96774643121581	0.07421901760514	3.05090809497733
H	-3.12557829508299	0.70418557527526	3.77374770318902
H	-3.54296059759696	-0.68507146945840	3.24953956169343
Cl	-0.04136238934923	1.41063590211184	2.97269024118528
Cl	-1.79505675727095	-2.82555279198914	3.41673434274067
O	1.10122749703486	-1.47681260056926	3.39312924684593
H	1.30502965809959	-2.20811178677022	2.77860588753197
H	1.77035967573458	-0.79269894675347	3.22364200706763
H	1.35172699982677	1.75055735098770	-2.59177102328154
C	0.87623810037112	1.19173142976258	-1.78066238945080
C	-0.33247931383977	-0.24984480654158	0.31190468874398
C	1.65432165755725	0.36572904298365	-0.95960665577776
C	-0.50771350470466	1.28994037523013	-1.56898324794303
C	-1.10389884378370	0.57687959038145	-0.51824982771946
C	1.05288852373919	-0.38114349404010	0.06814714870425
H	-2.17610989318606	0.65482234800945	-0.32661222101303

H	1.66394737424777	-0.98340920695960	0.73606975402891
C	-1.34015680736284	2.20958373568608	-2.42502953567705
H	-2.35387471184607	1.78946144159160	-2.53561407262548
H	-0.90407394975112	2.27307363842200	-3.43593006296569
O	-0.91752109098388	-0.98878570327926	1.24408027282275
O	2.98479688423902	0.20810833497450	-1.13388536290117
H	3.29568749095989	0.74414113773528	-1.88096681260720
C	-1.44427916599110	3.62494890060357	-1.83025631961148
H	-1.85783302828922	3.55681995517775	-0.80803166830482
H	-0.42811817213167	4.04458445341475	-1.72092741922963
C	-2.30397566019955	4.56807625140362	-2.67216143279113
H	-3.31810541031257	4.13925375062623	-2.77896434366527
C	-2.40911103666465	5.97977848571838	-2.09004973160262
H	-1.88993922868776	4.62360093506257	-3.69653256530348
H	-2.81788764755075	5.91831817715271	-1.06460841402989
H	-1.39361492929412	6.40399837036535	-1.98481851170750
C	-3.27328205066688	6.91938966737102	-2.93135117947940
H	-4.30430666425538	6.53702936089899	-3.02393636381225
H	-3.32902217031730	7.92624114992871	-2.48631972541415
H	-2.86847028592093	7.02695298241902	-3.95230058909017
H	-3.06378205273689	-2.70129628471437	-3.77481529592900
H	-3.28817166305970	-1.40830908763747	-2.60119772127582
H	-2.18943709097088	-1.13502723280112	-3.99467031198267

NORM-C5 π-complex Doublet:

Charge: +1 | Multiplicity: 2 | Imaginary frequency: ---

E_{elec}(def2-TZVPP): -3381.863087 a.u.

Thermal correction to Gibbs Free Energy (quasi-RRHO) (def2-SVP): 0.477877 a.u.

Absolute Gibbs Free Energy: -3381.385210 a.u.

H	-0.95295099171649	-4.61689025297170	0.12961826757438
C	-0.79382194989662	-3.62210284946075	-0.30846487810498
C	-1.46273386258391	-2.28239958991771	-2.37039173446627
C	-1.77566845816765	-3.42414065411044	-1.46446556223485
C	0.66353770736902	-3.47992039604300	-0.75955166661169
H	-1.77530185073963	-4.31287657548903	-2.13248608615620
H	0.89184398818393	-4.32046014638465	-1.46359726958613
H	-1.00323617963516	-2.88318035641028	0.47705635892701
C	-2.55287858182862	-1.73059269526050	-3.20930325073218
H	-2.81204896348750	-3.34194626432273	-1.10015697506072
C	1.72124577768839	-3.62592862263882	0.32790735008950
C	1.40389146042620	-4.02204571013202	1.57324496743687
H	2.19514893350115	-4.20116700948658	2.30856239704317
H	0.37788342379937	-4.20590016574777	1.89710838394869
C	3.14957692737341	-3.38018842181621	-0.08275487199513
H	3.84783706571187	-3.71243774552032	0.69813733785173
H	3.33729188930281	-2.30944730233470	-0.26988782220078
H	3.38981632650465	-3.91387134036906	-1.01841235540915

C	0.85299653090350	-2.31108361256223	-1.66739707870756
H	1.87226915449159	-1.98121236096344	-1.87423299127581
C	-0.16405624790528	-1.80741048088391	-2.47201285452803
H	0.06641633062738	-1.01810844233505	-3.18739524420567
Fe	-0.86377453443435	-0.82326213375787	3.13861752619426
O	-0.88509978868905	-0.75845525111474	5.16009532527978
H	-0.09657141168995	-0.34057910005360	5.54694890199919
H	-0.94644340845167	-1.66059690229254	5.52285357972354
O	-2.75115317494536	-0.20327386209751	3.27547892147427
H	-2.88948346327056	0.22359570958471	4.14009668621062
H	-3.27687244881607	-1.02613693826595	3.31001792601201
Cl	0.01665560323479	1.27467314735448	3.21705054832041
Cl	-1.70567877229717	-2.96780045051022	3.36807341036566
O	0.98717307007302	-1.47668890475270	3.22467421681102
H	1.22302444143361	-2.21940098731945	2.62762619278430
H	1.61845197934298	-0.74965970533022	3.07660678303365
H	1.37792812593822	1.75813521212421	-2.51024292684387
C	0.87310189372366	1.21396165452490	-1.70720838921571
C	-0.41221545792652	-0.19016826014157	0.38356653928179
C	1.61382657885656	0.39590767637510	-0.84869499924072
C	-0.51635147194548	1.31200388199456	-1.54308249997469
C	-1.14614553886351	0.61826052885432	-0.49863006117527
C	0.97573179541867	-0.33892493424949	0.16972948936401
H	-2.22476414285146	0.70112232918348	-0.34853503760677
H	1.58189823968747	-0.94139141776391	0.83904874748025
C	-1.32431768653643	2.20422171851230	-2.45050786624826
H	-2.33068235263552	1.77350903364023	-2.58585688837432
H	-0.85320984664409	2.24256383779928	-3.44700345487811
O	-1.07158290138064	-0.86550860760137	1.31873758932495
O	2.95060105542287	0.23042617334828	-0.97096461685381
H	3.28936180934969	0.75610703887720	-1.71320789793169
C	-1.46003494040770	3.63570640631800	-1.90354078880838
H	-1.91872446128573	3.59635633548387	-0.89924245918323
H	-0.45157612908074	4.06431327259223	-1.76251331538651
C	-2.28540059596562	4.54752623099242	-2.81190109378961
H	-3.29165828437474	4.10946077998639	-2.95096421691238
C	-2.42347700135833	5.97641455023936	-2.28100286411703
H	-1.82576652587390	4.57344345196018	-3.81788318718086
H	-2.88007009836414	5.94572433619404	-1.27454245224459
H	-1.41578676786236	6.40940431135534	-2.14191622338336
C	-3.25058386612674	6.88353332104120	-3.19243187280861
H	-4.27450024241513	6.49261287504322	-3.32113288458330
H	-3.33127753083998	7.90414088653597	-2.78411246856847
H	-2.79819212559948	6.95944941744825	-4.19612109019630
H	-3.10136128607600	-2.54744688890299	-3.70999922375959
H	-3.28905516452392	-1.23911726711081	-2.54654505632222
H	-2.19390660087103	-1.00378051093771	-3.94951696966775

ABN-C5 π-complex Quartet:

Charge: +1 | Multiplicity: 4 | Imaginary frequency: ---

E_{elec}(def2-TZVPP): -3381.878662 a.u.

Thermal correction to Gibbs Free Energy (quasi-RRHO) (def2-SVP): 0.470004 a.u.

Absolute Gibbs Free Energy: -3381.408658 a.u.

H	1.07342267271632	-4.63810784513242	0.38885746771906
C	0.58689241019993	-3.76496959204213	-0.06884042030133
C	-1.52924652396379	-3.04320624926493	-1.23447710007777
C	-0.86070037861337	-4.09512113568312	-0.42619428193904
C	1.38267283610611	-3.31232096846137	-1.29722386983393
H	-0.91233359628086	-5.01284542982212	-1.05232444400915
H	1.46366969389449	-4.18225931778249	-1.99120929253844
H	0.57117368044024	-2.96190351291933	0.68020827907314
C	-2.99976049681650	-2.96446020738626	-1.22185777607750
H	-1.46375938969476	-4.30127289841679	0.47026710660851
C	2.81548664913421	-2.84974268200980	-1.03892873954123
C	3.22865585142869	-2.43696800854291	0.16663241578946
H	4.26412981355505	-2.11995000355586	0.32146678165208
H	2.57017983362712	-2.40842786003666	1.03730636085415
C	3.73965373919464	-2.92492602826931	-2.22487274427356
H	4.71967282532542	-2.47893722766119	-2.00345868014319
H	3.32152828465952	-2.41029443219798	-3.10646825595399
H	3.89059054594974	-3.97746143832911	-2.52416323051383
C	0.60712116939375	-2.32041586866431	-2.10066653255524
H	1.15387011591481	-1.68640841189119	-2.80519406357244
C	-0.76162724147539	-2.21138892370407	-2.06875561843047
H	-1.26157724395082	-1.47063749127431	-2.69486058663683
Fe	-1.81725174235131	-1.14939768616841	2.53501869655377
O	-2.46127583781221	-1.16925877467899	4.48105718036557
H	-1.88698638664017	-0.63949265671048	5.06087136589487
H	-2.50960790770203	-2.06422586509573	4.85847066783689
O	-3.68135080150207	-0.13436540695474	2.04770998813672
H	-3.69449343078752	0.76770177361171	2.40974647944977
H	-4.46416708153832	-0.57717810468185	2.41504450494598
Cl	-0.81175131993966	0.85556986827079	3.05844122041141
Cl	-2.91087409628388	-3.22253250121701	2.36276549887417
O	-0.04454869575953	-2.20498735839569	3.27625742541175
H	-0.01508281363189	-3.14436152505262	3.03258804123146
H	0.80931912392403	-1.82819812523756	3.00860312808204
H	0.47111108599808	2.82922291519606	-2.13510552922284
C	0.11926690831443	1.99882428858478	-1.51509140142444
C	-0.82153221752542	-0.10390227101479	0.07333652862350
C	1.04288172387670	1.16645270971511	-0.86148686855556
C	-1.26436168893705	1.78926211135853	-1.37004065927236
C	-1.73930651642410	0.74251103831536	-0.57532813915369
C	0.56735401002694	0.11934076839971	-0.06913360940420
H	-2.81002802661364	0.58273320462525	-0.44632270985550

H	1.25893145593853	-0.52906007254036	0.46686513491980
O	-2.17745867081985	2.58216023862855	-1.97999876547244
H	-3.42452374368779	-3.95836532358772	-1.45387428281026
H	-3.31351201567321	-2.75430425653242	-0.18202414827207
O	-1.25182747424572	-1.16450679385815	0.74318498004804
C	2.52679718936413	1.38350118392628	-1.03597742564992
H	-3.39743313540440	-2.20863701780506	-1.91049571760505
C	3.05252360595377	0.91371531314223	-2.40078926613105
H	2.76098466714750	2.45526341787788	-0.91171581453263
H	3.06570178247519	0.84559109798184	-0.23915322070708
H	-1.72873770421516	3.26939449656111	-2.49617303184464
H	2.56906469657330	1.50001717729330	-3.20277754773049
C	4.57188656756345	1.01573635626311	-2.52827554998580
H	2.74534784089563	-0.13268089034853	-2.56096169408988
H	4.88806698483697	2.05822705674861	-2.33606767041542
H	5.04209775157321	0.40570973215566	-1.73404264292727
C	5.10343388326279	0.56702898269865	-3.89169191488300
H	4.63909185377362	1.18450364104269	-4.68248378208052
C	6.62592349318345	0.64806209432174	-4.00345019470161
H	4.77231966850353	-0.46959384633093	-4.08456638276827
H	6.98266725023644	1.68006557333525	-3.84401030376409
H	7.11499019119451	0.01004992681339	-3.24740391479408
H	6.97881832213316	0.32167904239223	-4.99533042802919

ABN-C5 π-complex Doublet:

Charge: +1 | Multiplicity: 2 | Imaginary frequency: ---

E_{elec}(def2-TZVPP): -3381.864361 a.u.

Thermal correction to Gibbs Free Energy (quasi-RRHO) (def2-SVP): 0.477866 a.u.

Absolute Gibbs Free Energy: -3381.386495 a.u.

H	0.99736818245309	-4.77112974365237	0.47018530819494
C	0.52257272439105	-3.87507351571024	0.04579676937767
C	-1.54136357576901	-3.12145306463203	-1.20587902283686
C	-0.90553179700600	-4.19432045574257	-0.39276558147710
C	1.37170087483035	-3.33665843578743	-1.10971133846512
H	-0.92666523903334	-5.09962152402608	-1.03727487348493
H	1.48075779828181	-4.15350329372674	-1.86379053273846
H	0.46812081090252	-3.11629577378611	0.83697129489967
C	-3.01463238724430	-3.05468874481854	-1.25909426005951
H	-1.54754716058582	-4.42195547883414	0.47125395524549
C	2.79761669354712	-2.92334123315634	-0.75818109729390
C	3.20387626718028	-2.76549878631896	0.50867585628011
H	4.23813550803542	-2.48649457541058	0.73003204765789
H	2.53829354642729	-2.90519186589355	1.36301430455051
C	3.73290996238694	-2.75063001327435	-1.92583753457375
H	4.73609910001453	-2.44705106378114	-1.59451386101650
H	3.36955398144411	-1.99094890364109	-2.63747491261065
H	3.82158119999268	-3.69373146861000	-2.49339889362199

C	0.62755892772753	-2.30068883911285	-1.88762682243177
H	1.19812548969347	-1.62791430984433	-2.53175152214197
C	-0.74991368778440	-2.25188596597691	-1.96017379973080
H	-1.22672633514235	-1.49972320515335	-2.59046594001737
Fe	-1.92464690058797	-1.10786434836926	2.57552364938240
O	-2.77006967515411	-1.01495366129060	4.41114112008821
H	-2.27835796227302	-0.37887280200392	4.96177667653384
H	-2.77243995266636	-1.86385077187883	4.88568584980757
O	-3.43374775646675	0.00519404843352	1.95743374748844
H	-3.25055136342068	0.92971412228947	2.21146494723936
H	-4.26711998619844	-0.24106501671318	2.39656990343434
Cl	-0.85377705572021	0.82579535285770	3.16028718927957
Cl	-3.10621127057702	-3.07046796105385	2.33249714327690
O	-0.47259815364259	-2.20891595576488	3.35589774214298
H	-0.60686008643780	-3.13891095611819	3.09703407229084
H	0.40255059607921	-1.94716091312758	3.02089164194263
H	0.63311685159784	2.54256021949394	-2.22600308742526
C	0.27693618210719	1.75790917286067	-1.55285549396082
C	-0.70576087257267	-0.21986386247776	0.18249182129043
C	1.18018731063132	0.90612337628534	-0.90561830737557
C	-1.10448129188416	1.62058931128906	-1.33491870340205
C	-1.60083696069318	0.63245775774488	-0.47489180100518
C	0.68263657230208	-0.08436721136941	-0.04395462655853
H	-2.67467086524952	0.53063089263593	-0.32504338967720
H	1.36642035191424	-0.73714370110309	0.49745985968611
O	-2.00818822520906	2.42375168747044	-1.94421092168497
H	-3.43409407428524	-4.05457136554280	-1.46938413198542
H	-3.36996701706850	-2.79886383791855	-0.24225336599208
O	-1.12799800261376	-1.24358244271640	0.92979090333235
C	2.67073021851147	1.04362994223446	-1.12132094820367
H	-3.38578614382419	-2.32231969066282	-1.98742494287711
C	3.10794063467303	1.03988460233718	-2.59224162101297
H	3.01772563984808	1.98280753344983	-0.65165989985149
H	3.18244097678696	0.22668057662139	-0.58792185063449
H	-1.55321370460187	3.06110771775068	-2.51623758819721
H	2.67645579624077	1.91074408232245	-3.11590113037099
C	4.62747561394231	1.05167318137402	-2.76203049066965
H	2.68969363018032	0.14986530249291	-3.09586804593283
H	5.04373820725370	1.95052809165085	-2.26960953818013
H	5.06045906072854	0.18668987466069	-2.22599780968560
C	5.07909499871245	1.01602375848339	-4.22354278492738
H	4.64308377238814	1.87941651210290	-4.75908019627483
C	6.59889186473471	1.02262566338509	-4.38907566817135
H	4.65727476384299	0.11627092091478	-4.70868419161759
H	7.04493863675515	1.92852270679971	-3.94397490503197
H	7.05783236118479	0.15099569764753	-3.89157480471952
H	6.89204639598787	0.99363265341302	-5.45122456549496

NORM-C5 transition state (1TS):

Charge: +1 | Multiplicity: 6 | Imaginary frequency: -142 cm^{-1}

E_{elec}(def2-TZVPP): -3381.89848224 a.u.

Thermal correction to Gibbs Free Energy (quasi-RRHO) (def2-SVP): 0.472908 a.u.

Absolute Gibbs Free Energy: -3381.425574 a.u.

H	-0.82745600000000	-4.74629800000000	0.15304000000000
C	-0.74016800000000	-3.75823200000000	-0.31978400000000
C	-1.42344600000000	-2.55551900000000	-2.46063500000000
C	-1.68133800000000	-3.69276200000000	-1.52312200000000
C	0.71435100000000	-3.50846900000000	-0.72851200000000
H	-1.58269500000000	-4.61070600000000	-2.13980800000000
H	1.00624400000000	-4.31168400000000	-1.44960900000000
H	-1.03931300000000	-3.01187400000000	0.42795500000000
C	-2.51096000000000	-2.17209800000000	-3.40117100000000
H	-2.73549900000000	-3.67727600000000	-1.20171700000000
C	1.75878300000000	-3.61970600000000	0.37279500000000
C	1.43673200000000	-3.98938500000000	1.62463800000000
H	2.22330900000000	-4.14344300000000	2.37054900000000
H	0.41026300000000	-4.18194500000000	1.94210800000000
C	3.18912900000000	-3.38058600000000	-0.03686900000000
H	3.88234200000000	-3.63680300000000	0.77661700000000
H	3.36703000000000	-2.32790200000000	-0.31338000000000
H	3.44795900000000	-3.98642600000000	-0.92255800000000
C	0.84346700000000	-2.30276200000000	-1.61303100000000
H	1.85419600000000	-1.99248000000000	-1.87986500000000
C	-0.19784900000000	-1.93562200000000	-2.49240000000000
H	-0.01114400000000	-1.14536600000000	-3.22097800000000
Fe	-1.01020600000000	-0.80600100000000	3.07037100000000
O	-1.01124800000000	-0.60384900000000	5.24773300000000
H	-0.38565100000000	0.07487600000000	5.55092700000000
H	-0.77733400000000	-1.42524400000000	5.71050100000000
O	-3.00162900000000	-0.04173900000000	3.15008100000000
H	-3.00463200000000	0.89108100000000	3.42485600000000
H	-3.58832800000000	-0.50799500000000	3.76900100000000
Cl	-0.22809500000000	1.39982800000000	3.14538000000000
Cl	-1.77513500000000	-2.94770100000000	3.46790400000000
O	1.02832600000000	-1.44110600000000	3.31879800000000
H	1.28527800000000	-2.17897200000000	2.72859600000000
H	1.64373500000000	-0.71282900000000	3.12906600000000
H	1.29797400000000	1.78143400000000	-2.67598800000000
C	0.82108800000000	1.20174200000000	-1.88114200000000
C	-0.40945500000000	-0.25814900000000	0.20408900000000
C	1.58426000000000	0.34396000000000	-1.09271400000000
C	-0.56147400000000	1.31638100000000	-1.64707900000000
C	-1.16684700000000	0.59607900000000	-0.60653300000000
C	0.96689700000000	-0.46205400000000	-0.10160000000000
H	-2.23300900000000	0.70974400000000	-0.40077700000000

H	1.59067600000000	-1.01908200000000	0.59394200000000
C	-1.38121200000000	2.27615600000000	-2.46851400000000
H	-2.40789500000000	1.88753700000000	-2.57084400000000
H	-0.95777100000000	2.35150700000000	-3.48371800000000
O	-0.96467200000000	-0.91474800000000	1.19873500000000
O	2.90876100000000	0.17065900000000	-1.25931900000000
H	3.23735500000000	0.71969600000000	-1.99014800000000
C	-1.43498000000000	3.68003600000000	-1.83845500000000
H	-1.83776400000000	3.59974300000000	-0.81298000000000
H	-0.40599400000000	4.06803700000000	-1.73376000000000
C	-2.27824900000000	4.66533200000000	-2.64815900000000
H	-3.30455100000000	4.26622900000000	-2.75301800000000
C	-2.33882300000000	6.06488300000000	-2.03148500000000
H	-1.87389800000000	4.73409200000000	-3.67549600000000
H	-2.73874000000000	5.99000500000000	-1.00345400000000
H	-1.31123600000000	6.45918800000000	-1.92738300000000
C	-3.18585200000000	7.04694100000000	-2.84118100000000
H	-4.22756900000000	6.69434400000000	-2.93202800000000
H	-3.21031600000000	8.04385900000000	-2.37167700000000
H	-2.78829700000000	7.16837000000000	-3.86340600000000
H	-2.90710800000000	-3.06306500000000	-3.91855900000000
H	-3.35580300000000	-1.75714900000000	-2.82155200000000
H	-2.18542300000000	-1.42764400000000	-4.14022800000000

NORM-C5 π-complex (1 π-complex):

Charge: +1 | Multiplicity: 6 | Imaginary frequency: ---

E_{elec}(def2-TZVPP): -3381.900118 a.u.

Thermal correction to Gibbs Free Energy (quasi-RRHO) (def2-SVP): 0. 471612 a.u.

Absolute Gibbs Free Energy: -3381.428506 a.u.

H	-0.99220694570166	-4.54524169466212	0.06510554380386
C	-0.79675018037844	-3.55069901959504	-0.35768480736054
C	-1.42543858236849	-2.16927130390160	-2.40748777356281
C	-1.77904088562995	-3.29890484818286	-1.50272322808975
C	0.66335722366578	-3.45436771839348	-0.81352052914033
H	-1.82236471305751	-4.18224968093180	-2.17665852109031
H	0.85699071280769	-4.29396887411531	-1.52956740613595
H	-0.97207080535407	-2.81965871256553	0.44304970483107
C	-2.49562687624645	-1.57860331922956	-3.24492741372833
H	-2.80788476367329	-3.17543430596066	-1.12906947522309
C	1.71865193082140	-3.65784023898992	0.26731086579128
C	1.39581696018945	-4.12127906690297	1.48715831133679
H	2.18367626590698	-4.34444478281768	2.21384734834128
H	0.36867899893225	-4.32317587517050	1.79623850230110
C	3.14996210704099	-3.40238572863457	-0.12634039647644
H	3.84197075529059	-3.75990473027742	0.64885757185786
H	3.34372754214001	-2.32794293068346	-0.28347015356209

H	3.39498937835330	-3.91069317970949	-1.07509267570034
C	0.88960510591319	-2.28581226392779	-1.71160563800926
H	1.91768000928438	-1.97566686737950	-1.90572040797226
C	-0.10746570856660	-1.74704425153308	-2.51528091238796
H	0.15004523558315	-0.96825735378499	-3.23243865671996
Fe	-0.94116214494135	-0.95890014700800	3.23307396654951
O	-0.92934412304235	-1.04975882879711	5.42981148575138
H	-0.25832388310335	-0.45232948015810	5.79973874239086
H	-0.72970212021903	-1.93759574855572	5.76902888787053
O	-2.88643513407752	-0.10223837142304	3.47571413137529
H	-2.80112531453980	0.81592526736149	3.78546333429374
H	-3.43147259133609	-0.55515257361729	4.14089616353108
Cl	-0.00607292057923	1.16171902396015	3.62254514873483
Cl	-1.80894847528229	-3.10336774467239	3.37215390312053
O	1.05579135396021	-1.73560770514829	3.38940393270741
H	1.29112338587426	-2.40929072983099	2.71884600129452
H	1.71356455706825	-1.02409494872323	3.31267733597727
H	1.38418712155877	1.76262177030402	-2.54461933589763
C	0.89172015479294	1.22672537096445	-1.72841663113806
C	-0.35815613524329	-0.15500187395114	0.38889594632407
C	1.65060159530883	0.42871599913404	-0.86431311818953
C	-0.49766806113027	1.30972491157620	-1.55899848722521
C	-1.11578847841342	0.62120368614357	-0.50303036797693
C	1.03056126531514	-0.29167359158893	0.17362389467086
H	-2.19499005377886	0.69111302119654	-0.35151037320906
H	1.63935937093057	-0.87545694994666	0.86020538939555
C	-1.32251836371777	2.17299853265337	-2.47934576251361
H	-2.32105316928617	1.72207183396417	-2.60623049457063
H	-0.85356131085853	2.20293694179473	-3.47710036366896
O	-0.95556681247092	-0.79621939339718	1.37963771782977
O	2.98751825126486	0.27769598001865	-0.99661789544951
H	3.31295069221457	0.79536739818196	-1.75036219689309
C	-1.48296334251509	3.61076186768134	-1.95633888984781
H	-1.93938286326302	3.58040237494223	-0.95068145742976
H	-0.48203671706876	4.05947630059055	-1.82455594702853
C	-2.32586914016557	4.49243245426751	-2.87826613255575
H	-3.32522441105696	4.03559543575363	-3.00624947736262
C	-2.48557401279437	5.92816281330701	-2.37243734927223
H	-1.86967099520347	4.50795607654521	-3.88600647262675
H	-2.93779199371967	5.90806132699703	-1.36373316891776
H	-1.48460458220465	6.37997406292493	-2.24523752770585
C	-3.33095869704644	6.80511216370749	-3.29652416058085
H	-4.34907320310727	6.39572398122651	-3.41321203144848
H	-3.42603675371274	7.83165760994093	-2.90663725825718
H	-2.88424132316162	6.86962365802189	-4.30353441762793
H	-3.06546315629501	-2.37698736530749	-3.75193794841815
H	-3.22017570624238	-1.07090290082204	-2.58210898874031
H	-2.11362952366327	-0.85926376286271	-3.98064358036838

ABN-C5 transition state (20) :

Charge: +1 | Multiplicity: 6 | Imaginary frequency: -197 cm^{-1}

E_{elec}(def2-TZVPP): -3381.89648499 a.u.

Thermal correction to Gibbs Free Energy (quasi-RRHO) (def2-SVP): 0.473064 a.u.

Absolute Gibbs Free Energy: -3381.423421 a.u.

H	1.04461291408412	-4.60287502100565	0.55841147628080
C	0.51994899390061	-3.73029081895865	0.14269186056268
C	-1.53343509662570	-3.13116265301101	-1.23459763057017
C	-0.87025510369400	-4.13797611634822	-0.34387194667570
C	1.35229994730306	-3.12844720833572	-0.99260688360282
H	-0.81377840469903	-5.07978506745832	-0.92753250469539
H	1.40357328605185	-3.89228539218515	-1.80250926557081
H	0.41443779960037	-2.99349481075384	0.95104136628093
C	-3.00121356099276	-3.27213944813638	-1.44397611230658
H	-1.53573274886474	-4.35563498088995	0.50627004984323
C	2.80202081164223	-2.80950367732093	-0.66436913105873
C	3.24362849635971	-2.68304510882520	0.59500856055417
H	4.29612891596505	-2.46541728138263	0.79914494227636
H	2.58569570601530	-2.78981817468988	1.46068876816525
C	3.72405905935318	-2.70117853601356	-1.85052514203075
H	4.73674163009314	-2.39537245965976	-1.55148597320951
H	3.35312984727438	-1.97844507534581	-2.59507663135565
H	3.79207834174137	-3.67319066674318	-2.37034132963284
C	0.59103038511372	-2.02571425881043	-1.68725650472912
H	1.13157758447308	-1.41980811934566	-2.41374084279810
C	-0.80470359752893	-2.16571258637284	-1.88175120204645
H	-1.30899970539664	-1.45927729667096	-2.54474253003060
Fe	-2.01634516212610	-1.12527918257775	2.65456591005700
O	-2.96221896326089	-1.10601547437973	4.62196537670310
H	-2.42218140771100	-0.63894702917003	5.28087654384965
H	-3.15150659488881	-1.98602132299017	4.98686313588263
O	-3.70834007188644	0.00589985356632	1.99432316853326
H	-3.79887061069505	0.86545075204572	2.43935267262688
H	-4.56064133857510	-0.44964775853307	2.09881375962844
Cl	-1.01924784400385	0.81875363074825	3.41691526461300
Cl	-3.24135724114065	-3.07891184075290	2.34512531473408
O	-0.47006674245802	-2.30792414033470	3.55289594190424
H	-0.45164513844763	-3.22999984449792	3.24683135537998
H	0.43000114191188	-1.96104169372687	3.43465740118865
H	0.79108077137122	2.51198008983476	-2.26024370408892
C	0.40580445092591	1.73099503084922	-1.60005363626485
C	-0.67239893472614	-0.17304091767306	0.17500285798834
C	1.25472454956700	0.78862468380603	-1.04062226734849
C	-0.97266851741211	1.73255741558379	-1.27725277184785
C	-1.51012474711365	0.79518025738431	-0.38942527545474
C	0.70764221539976	-0.23288283998522	-0.20914477363610

H	-2.56830026410032	0.82417178327056	-0.13195696624548
H	1.37918071040361	-0.83401568924365	0.40172921898668
O	-1.81077595073971	2.64260231649130	-1.79601293076195
H	-3.24664873927598	-4.29937842732590	-1.76697618621100
H	-3.51408651397237	-3.12719809448359	-0.47627187158570
O	-1.14416247374391	-1.08955071278304	0.98309331078003
C	2.74442772309832	0.84276950990500	-1.28377832465313
H	-3.39361985100778	-2.55529345674224	-2.17844912943462
C	3.16553855397553	0.96730917428896	-2.75430754209732
H	3.14720490841715	1.70950980937045	-0.72735388993546
H	3.21515007128325	-0.04463908726785	-0.83605559484623
H	-1.33504640302167	3.24864022525022	-2.38676274875544
H	2.75113643855589	1.89348049482447	-3.18850728753045
C	4.68419100601009	0.96540200940267	-2.93259517801966
H	2.72475933509934	0.13813589529245	-3.33686598784481
H	5.11879633212023	1.80631197812154	-2.36037449282256
H	5.10303709147660	0.04645011290185	-2.48296710560737
C	5.12793176436197	1.06014157009985	-4.39383741598100
H	4.70781839644774	1.97936304138701	-4.84204894296789
C	6.64699123510703	1.05245850137783	-4.56594525750185
H	4.68624532267532	0.21940167222861	-4.96006870762736
H	7.11266609102686	1.90163921003679	-4.03709365593256
H	7.08955801758993	0.12746990145376	-4.15804348496983
H	6.93570588231322	1.12091335120972	-5.62743949653353

ABN-C5 π-complex (20 π-complex):

Charge: +1 | Multiplicity: 6 | Imaginary frequency: ---

E_{elec}(def2-TZVPP): -3381.899468 a.u.

Thermal correction to Gibbs Free Energy (quasi-RRHO) (def2-SVP): 0.472276 a.u.

Absolute Gibbs Free Energy: -3381.427192 a.u.

H	1.02244838034033	-4.66856982320251	0.54021692312052
C	0.51796589547389	-3.79957078477730	0.09432724358718
C	-1.56407577796174	-3.15351303233051	-1.19051012665870
C	-0.89500885527092	-4.18088120895649	-0.34241754137372
C	1.34710469672004	-3.26685978589454	-1.07811555753721
H	-0.87556697378652	-5.10511336976112	-0.95892767222285
H	1.44021322659415	-4.09156484680435	-1.82548888971298
H	0.44366780642843	-3.02470263434714	0.87012182962263
C	-3.04051909408433	-3.17180024663475	-1.27847668981941
H	-1.53426531268933	-4.41138670380303	0.52318454885787
C	2.77982015797624	-2.85918460348118	-0.75762860919127
C	3.21131024443672	-2.69249707983045	0.49989978440983
H	4.25093208362309	-2.41715763423804	0.69956768891179
H	2.56083532491484	-2.81997616948912	1.36788398710025
C	3.69296652155478	-2.70620658363407	-1.94495126070796
H	4.69934840774749	-2.38694392500721	-1.63933214695429
H	3.30858087986586	-1.96779598495606	-2.66742326699569

H	3.77904189199580	-3.66207462565672	-2.49115261877119
C	0.57806012268410	-2.24440172566474	-1.85467140190325
H	1.12608462977691	-1.57002041354785	-2.51519453360244
C	-0.80458634547817	-2.26090270122880	-1.94233292131494
H	-1.30619900411718	-1.53535549323904	-2.58494365185196
Fe	-1.96307306042830	-1.15366487214560	2.56706456621924
O	-2.80571668989839	-1.09995023784937	4.59616101038509
H	-2.23619287051886	-0.59228062929422	5.19762096878670
H	-2.92943752985861	-1.97404764989979	5.00070914948798
O	-3.72546099183616	-0.06841364231330	2.00522080412014
H	-3.72937379290075	0.83214280102617	2.37167469190177
H	-4.55658036076452	-0.48054255325546	2.29479614032594
Cl	-0.96234269171559	0.82613763380559	3.25433761262349
Cl	-3.14146254085938	-3.15640496859077	2.39846121896626
O	-0.33391171963340	-2.26115163866163	3.42147232156749
H	-0.29462024504629	-3.19832960749361	3.16951885687170
H	0.55041929680264	-1.89185740892957	3.26180091318529
H	0.75294109746104	2.60671410394923	-2.25077206991475
C	0.35642372080048	1.82183566237311	-1.60110947336299
C	-0.73042554870524	-0.14029359660304	0.08500406820684
C	1.21303327078666	0.90789698787481	-0.98193670487729
C	-1.03231780523141	1.76062271282341	-1.37696714588115
C	-1.57982075162677	0.78461548224343	-0.53711200042411
C	0.66490415014625	-0.08889683623374	-0.15267116930259
H	-2.65562354273821	0.74597662321176	-0.36473908987194
H	1.31645316468842	-0.77004826194892	0.39181274863504
O	-1.88548268216032	2.63542981157275	-1.95413634452274
H	-3.38668454097242	-4.18406947176983	-1.55540709514870
H	-3.44956929151668	-2.98928581785492	-0.26778423808447
O	-1.22868244500190	-1.10795769102997	0.84294831325394
C	2.70976666094387	0.98845164555822	-1.18362925050226
H	-3.43615703721431	-2.43349685722658	-1.98794100012033
C	3.15939590984211	1.02164229444644	-2.65056502729505
H	3.08876139103152	1.89576344164853	-0.67767642122142
H	3.18400226068419	0.13409460450642	-0.67568721691004
H	-1.39469192670053	3.26700555747074	-2.50272150689204
H	2.75005978421646	1.91672398166901	-3.15040655176539
C	4.68049492760338	1.01039328157634	-2.80671634850541
H	2.72866213537616	0.15595782386856	-3.18525445488896
H	5.10709029394992	1.89110267989637	-2.29097727084557
H	5.09456249965780	0.12703412920616	-2.28634518836613
C	5.14433715949572	0.99917728938825	-4.26482663731282
H	4.72705892225791	1.88094505155672	-4.78511540308837
C	6.66548020449081	0.98440532491391	-4.41707514187391
H	4.71238406534116	0.11715394143394	-4.77299965900458
H	7.12214301818081	1.87317643396183	-3.94885658868887
H	7.10566447385207	0.09497228061771	-3.93434652554892
H	6.96761475097329	0.97304953698691	-5.47703197730762

NORM-Methyl transition state (32 TS):

Charge: +1 | Multiplicity: 6 | Imaginary frequency: -164 cm^{-1}

E_{elec}(def2-TZVPP): -3224.680114 a.u.

Thermal correction to Gibbs Free Energy (quasi-RRHO) (def2-SVP): 0.363949 a.u.

Absolute Gibbs Free Energy: -3224.316165 a.u.

H	0.72738400000000	-4.81796300000000	0.86888400000000
C	0.31555400000000	-3.92144000000000	0.38353800000000
C	-1.60890300000000	-3.19396400000000	-1.10872800000000
C	-1.08436200000000	-4.21858900000000	-0.15131200000000
C	1.25920400000000	-3.46260500000000	-0.73023200000000
H	-1.09416800000000	-5.18441000000000	-0.69762200000000
H	1.30363800000000	-4.27949200000000	-1.48922500000000
H	0.24697800000000	-3.13433100000000	1.14735000000000
C	-3.07629100000000	-3.17430500000000	-1.35240500000000
H	-1.80211900000000	-4.33676800000000	0.67537300000000
C	2.70805300000000	-3.22897200000000	-0.32795000000000
C	3.09972100000000	-3.22972000000000	0.95457500000000
H	4.15166600000000	-3.08122100000000	1.21566800000000
H	2.40360000000000	-3.37391200000000	1.78375900000000
C	3.68400400000000	-3.03293200000000	-1.45885200000000
H	4.71895600000000	-3.00000100000000	-1.08856900000000
H	3.49174900000000	-2.08844700000000	-1.99261900000000
H	3.60141600000000	-3.85017300000000	-2.19627400000000
C	0.63523300000000	-2.35951900000000	-1.54312700000000
H	1.27238900000000	-1.85044800000000	-2.26689300000000
C	-0.75613700000000	-2.35107500000000	-1.78033800000000
H	-1.15720000000000	-1.62968200000000	-2.49553900000000
Fe	-1.98478300000000	-0.91545400000000	2.68685500000000
O	-3.02019900000000	-0.71917700000000	4.60137000000000
H	-2.47737900000000	-0.25721500000000	5.26161300000000
H	-3.29833800000000	-1.55809400000000	5.00391400000000
O	-3.55789300000000	0.30035300000000	1.89217000000000
H	-3.60145000000000	1.18394700000000	2.29529700000000
H	-4.44199000000000	-0.09157600000000	1.99117300000000
Cl	-0.88519100000000	0.99147000000000	3.40131100000000
Cl	-3.35613700000000	-2.78085200000000	2.40430800000000
O	-0.59431200000000	-2.17807100000000	3.71867300000000
H	-0.67694800000000	-3.11415000000000	3.47041000000000
H	0.34047500000000	-1.94333000000000	3.59387000000000
H	1.27076300000000	2.02190200000000	-2.48134100000000
C	0.78598900000000	1.38276900000000	-1.73797500000000
C	-0.45638800000000	-0.24204700000000	0.20480700000000
C	1.52507100000000	0.40179900000000	-1.08454300000000
C	-0.57538900000000	1.56883500000000	-1.41922300000000
C	-1.18541800000000	0.76584700000000	-0.45020900000000
C	0.90531900000000	-0.47435100000000	-0.15340900000000

H	-2.23478900000000	0.90602300000000	-0.18963300000000
H	1.53146500000000	-1.09630100000000	0.48498700000000
C	-1.35750900000000	2.63947100000000	-2.13015500000000
H	-2.39198800000000	2.69888500000000	-1.76404700000000
H	-1.38257700000000	2.44466100000000	-3.21563000000000
O	-1.02590200000000	-1.04151900000000	1.07218900000000
O	2.83545200000000	0.19831300000000	-1.31918100000000
H	3.16181400000000	0.82056900000000	-1.98918400000000
H	-0.87994800000000	3.62440500000000	-1.99618600000000
H	-3.43776000000000	-4.18565400000000	-1.60905100000000
H	-3.58398000000000	-2.90181300000000	-0.40928600000000
H	-3.36490200000000	-2.46821500000000	-2.14293600000000

NORM-Methyl π-complex (32 π-complex):

Charge: +1 | Multiplicity: 6 | Imaginary frequency: ----

E_{elec}(def2-TZVPP): -3224.682269 a.u.

Thermal correction to Gibbs Free Energy (quasi-RRHO) (def2-SVP): 0.361821 a.u.

Absolute Gibbs Free Energy: -3224.320448 a.u.

H	0.68781900000000	-4.86872200000000	0.87765200000000
C	0.30424400000000	-3.96706700000000	0.38007500000000
C	-1.63375300000000	-3.17654900000000	-1.04718800000000
C	-1.11810900000000	-4.21914100000000	-0.11592100000000
C	1.24624900000000	-3.56321600000000	-0.75750500000000
H	-1.16886900000000	-5.17121500000000	-0.68658800000000
H	1.32183800000000	-4.42924400000000	-1.46144200000000
H	0.27911300000000	-3.16084000000000	1.12630400000000
C	-3.09757200000000	-3.04321000000000	-1.20772300000000
H	-1.81952300000000	-4.33537900000000	0.72403800000000
C	2.68762700000000	-3.26627600000000	-0.35928800000000
C	3.10008400000000	-3.34331700000000	0.91374400000000
H	4.14728200000000	-3.15694400000000	1.16936700000000
H	2.42935800000000	-3.58616300000000	1.74023800000000
C	3.62947200000000	-2.91224900000000	-1.48156800000000
H	4.67603800000000	-2.96538100000000	-1.14857500000000
H	3.44908300000000	-1.88436000000000	-1.83717400000000
H	3.50079500000000	-3.58976600000000	-2.34302600000000
C	0.62097700000000	-2.53167700000000	-1.63941000000000
H	1.26811500000000	-1.96411700000000	-2.31122300000000
C	-0.74964900000000	-2.41203900000000	-1.80344900000000
H	-1.13878100000000	-1.68082000000000	-2.51380800000000
Fe	-1.98227100000000	-0.95116000000000	2.62876100000000
O	-2.94719400000000	-0.72292100000000	4.58885100000000
H	-2.37080900000000	-0.25221800000000	5.21340800000000
H	-3.19290100000000	-1.56094300000000	5.01359500000000
O	-3.58854600000000	0.26224700000000	1.89281800000000
H	-3.57570300000000	1.16101700000000	2.26306900000000
H	-4.46979400000000	-0.09928500000000	2.08603300000000

Cl	-0.84324400000000	0.95530000000000	3.29505900000000
Cl	-3.36300800000000	-2.82966600000000	2.45255300000000
O	-0.55574000000000	-2.19875100000000	3.63860500000000
H	-0.64585600000000	-3.14144900000000	3.42089300000000
H	0.37617100000000	-1.96868600000000	3.48765300000000
H	1.34704500000000	2.09015000000000	-2.36781900000000
C	0.82578500000000	1.44016700000000	-1.65875100000000
C	-0.49452200000000	-0.23592000000000	0.16711500000000
C	1.55479600000000	0.51781000000000	-0.90479400000000
C	-0.56878700000000	1.54345400000000	-1.49439500000000
C	-1.22111100000000	0.70911600000000	-0.58215100000000
C	0.90209600000000	-0.34302300000000	-0.00193900000000
H	-2.30118200000000	0.76950400000000	-0.44085900000000
H	1.48519600000000	-1.02362700000000	0.61701100000000
C	-1.33973300000000	2.55779700000000	-2.29930900000000
H	-2.42184200000000	2.47773900000000	-2.12162100000000
H	-1.15379300000000	2.42993000000000	-3.37860700000000
O	-1.12259300000000	-1.08204800000000	0.97069500000000
O	2.89959700000000	0.39292500000000	-1.01170200000000
H	3.24235000000000	1.02617100000000	-1.66177600000000
H	-1.02422000000000	3.58313200000000	-2.04136900000000
H	-3.53998200000000	-4.02800800000000	-1.44339300000000
H	-3.52680800000000	-2.75828400000000	-0.22919200000000
H	-3.37969400000000	-2.31131700000000	-1.97555600000000

ABN-Methyl transition state (33 TS):

Charge: +1 | Multiplicity: 6 | Imaginary frequency: -179 cm^{-1}

E_{elec}(def2-TZVPP): -3224.680387 a.u.

Thermal correction to Gibbs Free Energy (quasi-RRHO) (def2-SVP): 0.365690 a.u.

Absolute Gibbs Free Energy: -3224.314697 a.u.

H	0.81485400000000	-4.76775500000000	0.92215500000000
C	0.36473100000000	-3.90809100000000	0.40459900000000
C	-1.62140600000000	-3.30426800000000	-1.06528100000000
C	-1.04697500000000	-4.25923000000000	-0.06383700000000
C	1.25426800000000	-3.49758600000000	-0.77226600000000
H	-1.05488100000000	-5.25744900000000	-0.54830100000000
H	1.25698400000000	-4.34713300000000	-1.49376500000000
H	0.30827800000000	-3.08145000000000	1.12578900000000
C	-3.09243000000000	-3.35242700000000	-1.28698000000000
H	-1.73752300000000	-4.33896000000000	0.79046300000000
C	2.71939100000000	-3.24660000000000	-0.45162400000000
C	3.15303000000000	-3.01647300000000	0.79576800000000
H	4.21560200000000	-2.85119200000000	0.99682600000000
H	2.47925400000000	-2.98555000000000	1.65523400000000
C	3.66288400000000	-3.33386000000000	-1.62284600000000
H	4.68706900000000	-3.04990300000000	-1.34150200000000
H	3.34433100000000	-2.68930500000000	-2.45840200000000

H	3.68315100000000	-4.36497000000000	-2.01820200000000
C	0.58582200000000	-2.42213600000000	-1.59207700000000
H	1.18409600000000	-1.91976400000000	-2.35244100000000
C	-0.81156900000000	-2.46872100000000	-1.79525000000000
H	-1.25358100000000	-1.79448200000000	-2.53201400000000
Fe	-2.01307900000000	-0.90097500000000	2.58871700000000
O	-2.99992200000000	-0.63871200000000	4.52179100000000
H	-2.43364700000000	-0.18152000000000	5.16536700000000
H	-3.28795200000000	-1.46666000000000	4.93994500000000
O	-3.59164100000000	0.31237500000000	1.80419700000000
H	-3.63253800000000	1.19995300000000	2.19872000000000
H	-4.47458700000000	-0.07881100000000	1.91591500000000
Cl	-0.85972000000000	0.99951100000000	3.22592400000000
Cl	-3.40713300000000	-2.76121600000000	2.40870100000000
O	-0.61081200000000	-2.14743600000000	3.62185300000000
H	-0.69046400000000	-3.08997500000000	3.39907900000000
H	0.32444900000000	-1.91279000000000	3.50182600000000
H	1.19275600000000	2.02419200000000	-2.56439900000000
C	0.72150900000000	1.35273600000000	-1.84105800000000
C	-0.55182000000000	-0.28237700000000	0.06630200000000
C	1.47076400000000	0.38873200000000	-1.18324300000000
C	-0.65668200000000	1.50518100000000	-1.55555900000000
C	-1.29030200000000	0.70070400000000	-0.60316100000000
C	0.82462400000000	-0.49190200000000	-0.26879500000000
H	-2.34560100000000	0.84571400000000	-0.37386300000000
H	1.42830600000000	-1.09556100000000	0.40622800000000
O	-1.40121300000000	2.43513400000000	-2.17384600000000
H	-3.41559200000000	-4.38756900000000	-1.49609400000000
H	-3.60131500000000	-3.05871700000000	-0.35129000000000
O	-1.11928800000000	-1.07797000000000	0.94019500000000
C	2.94265600000000	0.24765600000000	-1.45932100000000
H	-3.41821300000000	-2.69513100000000	-2.10484900000000
H	3.38857500000000	1.21543800000000	-1.73183100000000
H	3.47825000000000	-0.16483800000000	-0.59247800000000
H	3.11553800000000	-0.43814200000000	-2.30680300000000
H	-0.86221100000000	2.93986000000000	-2.80404200000000

ABN-Methyl π-complex (33 π-complex):

Charge: +1 | Multiplicity: 6 | Imaginary frequency: ----

E_{elec}(def2-TZVPP): -3224.682878 a.u.

Thermal correction to Gibbs Free Energy (quasi-RRHO) (def2-SVP): 0.362331 a.u.

Absolute Gibbs Free Energy: -3224.320547 a.u.

H	0.77502100000000	-4.84383600000000	0.92116600000000
C	0.34836500000000	-3.98348000000000	0.38641700000000
C	-1.65971900000000	-3.31206400000000	-1.00361100000000
C	-1.08515100000000	-4.29388600000000	-0.03999000000000
C	1.23109600000000	-3.63344900000000	-0.81573600000000

H	-1.13122400000000	-5.27582600000000	-0.55797600000000
H	1.26285900000000	-4.53061800000000	-1.48095200000000
H	0.33208200000000	-3.13367100000000	1.08314000000000
C	-3.13147100000000	-3.23715100000000	-1.13159600000000
H	-1.75374500000000	-4.38253100000000	0.82975900000000
C	2.68990100000000	-3.31731500000000	-0.51161500000000
C	3.14345400000000	-3.17557400000000	0.74142200000000
H	4.20155800000000	-2.97208800000000	0.92985700000000
H	2.49493500000000	-3.25341900000000	1.61651800000000
C	3.60153900000000	-3.22169800000000	-1.70671000000000
H	4.64126900000000	-3.03651300000000	-1.40156100000000
H	3.30240400000000	-2.40723200000000	-2.38736900000000
H	3.57002300000000	-4.15410800000000	-2.29729900000000
C	0.55565600000000	-2.62992600000000	-1.69677000000000
H	1.16410400000000	-2.06080900000000	-2.40238000000000
C	-0.82282700000000	-2.55245400000000	-1.81640700000000
H	-1.25738100000000	-1.85659700000000	-2.53579000000000
Fe	-2.01607900000000	-0.90238700000000	2.52419500000000
O	-2.93214800000000	-0.59949600000000	4.49680100000000
H	-2.35883600000000	-0.07912600000000	5.08350300000000
H	-3.13935800000000	-1.42315000000000	4.96777200000000
O	-3.68602800000000	0.20222200000000	1.76366000000000
H	-3.71812900000000	1.11447700000000	2.09789100000000
H	-4.54764400000000	-0.19497700000000	1.97463300000000
Cl	-0.93345800000000	1.07952300000000	3.04414300000000
Cl	-3.31677700000000	-2.84366600000000	2.51064000000000
O	-0.49941100000000	-2.01925200000000	3.56105300000000
H	-0.54022200000000	-2.97516100000000	3.39197700000000
H	0.41209400000000	-1.74427400000000	3.36718400000000
H	1.27343200000000	1.99638500000000	-2.55041100000000
C	0.76725600000000	1.34685600000000	-1.82977100000000
C	-0.58043800000000	-0.28161700000000	0.01583900000000
C	1.50248900000000	0.45135900000000	-1.04809100000000
C	-0.62976100000000	1.43797000000000	-1.68308200000000
C	-1.30651300000000	0.62976000000000	-0.76201700000000
C	0.82460200000000	-0.38216000000000	-0.13928300000000
H	-2.38841700000000	0.70635100000000	-0.65084000000000
H	1.38077200000000	-1.04696100000000	0.51995300000000
O	-1.36853000000000	2.30134000000000	-2.41428700000000
H	-3.54033400000000	-4.24482600000000	-1.32882600000000
H	-3.55114300000000	-2.93785800000000	-0.15368800000000
O	-1.20502000000000	-1.10381200000000	0.84746300000000
C	3.00333800000000	0.39083200000000	-1.17543700000000
H	-3.45795300000000	-2.54161300000000	-1.91550100000000
H	3.45507400000000	1.36222800000000	-0.91368300000000
H	3.43279200000000	-0.37652500000000	-0.51742900000000
H	3.30521100000000	0.16760700000000	-2.21198500000000
H	-0.79453900000000	2.81377000000000	-3.00471700000000

7. Experimental Procedures and Compound Characterization

Compound S1 (1,3-Dimethoxy-5-pentylbenzene)

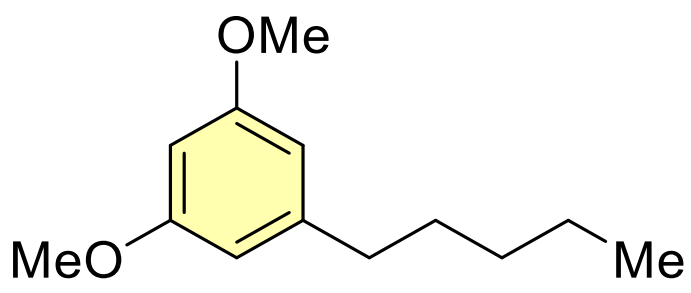

Olivetol (**18**) (1.0 g, 5.550 mmol, 1.0 equiv), methyl iodide (6.13 g, 44.40 mmol, 8.0 equiv) and potassium carbonate (6.3 mg, 44.40 mmol, 8.0 equiv) in 50 ml of anhydrous DMF were stirred for 15 h at 23 ºC under an argon atmosphere. The reaction mixture was quenched with 100 ml of water and extracted with diethyl ether. The organic phase was washed with water, brine, dried over anh. $MgSO_4$, and concentrated in vacuo using rotary evaporator. The residue was purified with flash column chromatography (100% hexanes to 6% Et_2O in hexanes) to afford the product **S1** (0.97 g, 85%) as a colorless oil. ^{1}H NMR (500 MHz, $CDCl_3$) δ 6.35 (d, *J* = 2.3 Hz, 2H), 6.30 (t, *J* = 2.3 Hz, 1H), 3.78 (s, 6H), 2.57 – 2.51 (m, 2H), 1.65 – 1.55 (m, 2H), 1.37 – 1.30 (m, 4H), 0.89 (t, *J* = 6.9 Hz, 3H). ^{13}C{^{1}H} NMR (125 MHz, $CDCl_3$) δ 160.8, 145.5, 106.6, 97.7, 55.3, 36.4, 31.7, 31.1, 22.7, 14.2. HRMS (ESI): m/z calcd for $C_{13}H_{21}O_2$ [M + H]$^+$: 209.1542, found 209.1538.

Compound 27

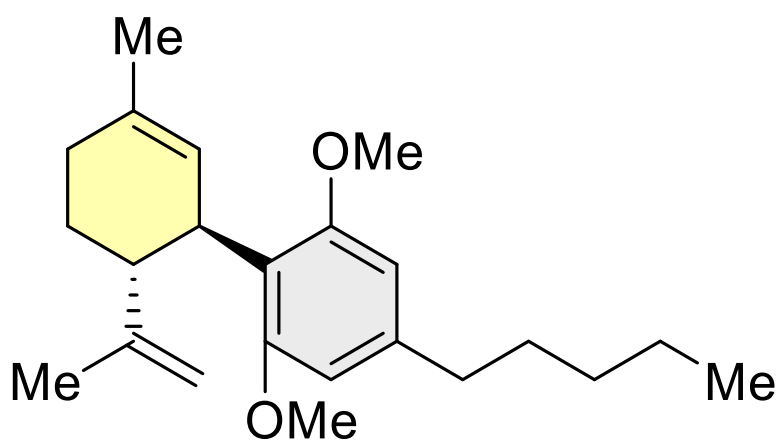

Compound **27** (17.7 mg, 17%, pale yellow oil) was prepared from olivetol dimethyl ether (**S1**) (64.3 mg, 0.309 mmol, 1.0 equiv) and (+)-*p*-mentha-2,8-dien-1-ol (**19**) (47.0 mg, 0.309 mmol, 1.0 equiv) according to general procedure and purified with flash column chromatography (100% hexanes to 6.5% Et_2O in hexanes). ^{1}H NMR (500 MHz, $CDCl_3$) δ 6.33 (s, 2H), 5.20 (tt, *J* = 2.4, 1.2 Hz, 1H), 4.44 (dq, *J* = 2.9, 1.1 Hz, 1H), 4.42 (dt, *J* = 2.6, 0.7 Hz, 1H), 3.98 (ddp, *J* = 10.8, 4.4, 2.4 Hz, 1H), 3.73 (s, 6H), 2.89 (td, *J* = 10.9, 3.8 Hz, 1H), 2.56 – 2.49 (m, 2H), 2.24 – 2.12 (m, 1H), 1.98 (ddt, *J* = 17.3, 5.0, 2.2 Hz, 1H), 1.78 – 1.70 (m, 2H), 1.66 (s, 3H), 1.64 – 1.61 (m, 1H), 1.60 (s, 3H), 1.56 (d, *J* = 8.2 Hz, 1H), 1.35 – 1.30 (m, 4H), 0.90 (t, *J* = 7.0 Hz, 3H). ^{13}C{^{1}H} NMR (125 MHz, $CDCl_3$) δ 158.6, 149.6, 141.9, 131.2, 125.9, 119.0, 109.6, 105.0, 56.0, 45.2, 36.4, 36.1, 31.7, 31.0, 30.8, 29.7, 23.5, 22.6, 19.1, 14.1. $[\alpha]^{25}_D$ = -155 (*c* 0.09, chloroform). HRMS (ESI): m/z calcd for $C_{23}H_{35}O_2$ [M + H]$^+$: 343.2637, found 343.2633.

Compound S2 (3-methoxy-5-pentylphenol)

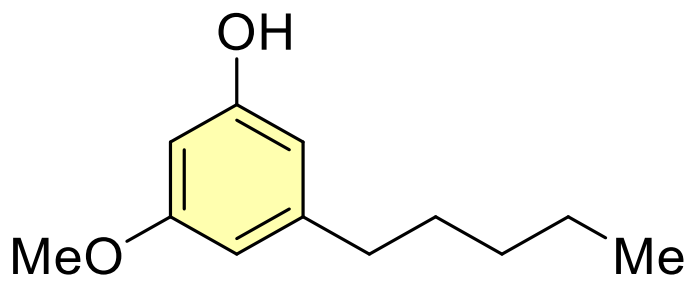

Olivetol (**18**) (1.0 g, 5.55 mmol, 1.0 equiv), methyl iodide (0.86 g, 6.060 mmol, 1.1 equiv) and potassium hydroxide (340 mg, 6.100 mmol, 1.1 equiv) in 50 ml of anhydrous DMF were stirred for 15 h at 23 ºC under an argon atmosphere. The reaction mixture was quenched with 100 ml of water and extracted with diethyl ether. The organic phase was washed with water, brine, dried over anh. $MgSO_4$, and concentrated in vacuo using rotary evaporator. The residue was purified with flash column chromatography (5% to 7.5% ethyl acetate in hexanes) to afford the product **S2** (500 mg, 46.4%) of as a pale brown oil. ^1H NMR (500 MHz, $CDCl_3$) δ 6.33 (dd, *J* = 2.3, 1.3 Hz, 1H), 6.27 (dd, *J* = 2.2, 1.3 Hz, 1H), 6.24 (t, *J* = 2.3 Hz, 1H), 3.77 (s, 3H), 2.54 – 2.47 (m, 2H), 1.64 – 1.53 (m, 2H), 1.40 – 1.25 (m, 4H), 0.89 (t, *J* = 6.9 Hz, 3H). $^{13}C\{^1H\}$ NMR (125 MHz, $CDCl_3$) δ 160.9, 156.6, 145.9, 108.0, 107.0, 98.8, 55.4, 36.2, 31.6, 31.0, 22.7, 14.2. HRMS (ESI): m/z calcd for $C_{12}H_{19}O_2$ [M + H]$^+$: 195.1385, found 195.1380.

Compound 28

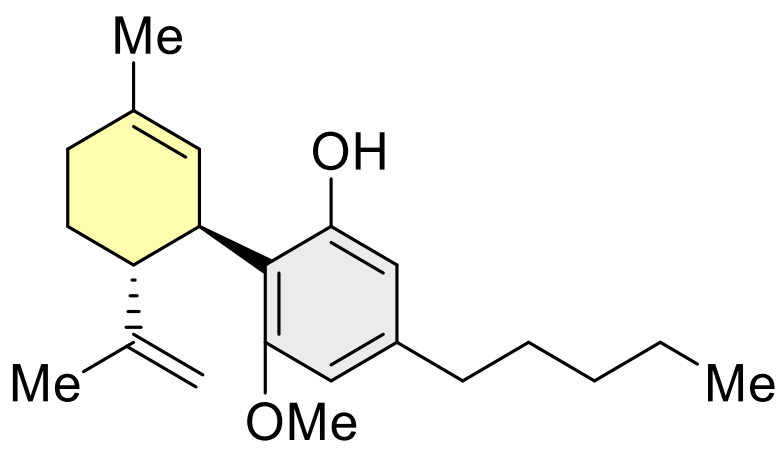

Compound **28** (57.9 mg, 58%, pale yellow oil) was prepared from olivetol monomethyl ether (**S2**) (59.9 mg, 0.309 mmol, 1.0 equiv) and (+)-*p*-mentha-2,8-dien-1-ol (**19**) (47.0 mg, 0.309 mmol, 1.0 equiv) according to general procedure and purified with flash column chromatography (100% hexanes to 2% Et_2O in hexanes). ^1H NMR (500 MHz, $CDCl_3$) δ 6.30 (d, *J* = 1.6 Hz, 1H), 6.22 (d, *J* = 1.6 Hz, 1H), 6.00 (s, 1H), 5.57 (s, 1H), 4.49 (dd, *J* = 2.4, 1.4 Hz, 1H), 4.32 (s, 1H), 3.99 (d, *J* = 10.4 Hz, 1H), 3.70 (s, 3H), 2.52 – 2.47 (m, 2H), 2.44 – 2.37 (m, 1H), 2.28 – 2.18 (m, 1H), 2.12 – 2.03 (m, 1H), 1.82 – 1.76 (m, 5H), 1.66 (s, 3H), 1.63 – 1.55 (m, 2H), 1.36 – 1.26 (m, 4H), 0.89 (t, *J* = 7.0 Hz, 3H). $^{13}C\{^1H\}$ NMR (125 MHz, $CDCl_3$) δ 158.3, 155.8, 147.4, 142.8, 139.7, 124.7, 115.2, 111.0, 109.7, 103.2, 55.7, 46.8, 36.2, 35.6, 31.7, 31.0, 29.9, 28.2, 23.9, 22.7, 18.9, 14.2. $[\alpha]^{25}_D$ =-57.8 (c 1.05, CH_2Cl_2). HRMS (ESI): m/z calcd for $C_{22}H_{33}O_2$ [M + H]$^+$: 329.2481, found 329.2478.

Compound 29

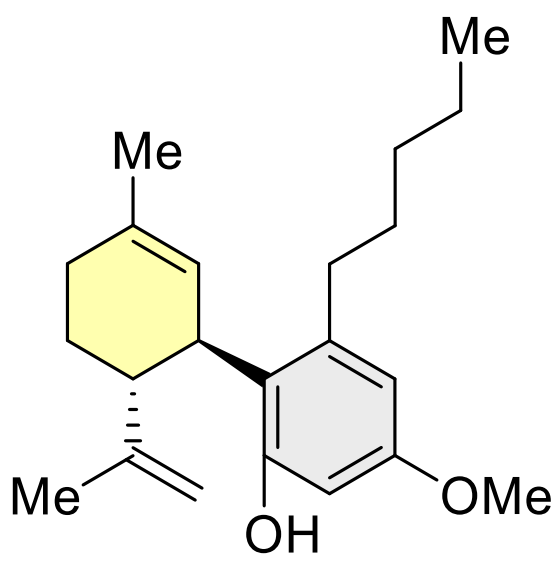

Compound **29** (23.2 mg, 23%, pale yellow oil) was prepared from olivetol monomethyl ether (**S2**) (59.93 mg, 0.3085 mmol, 1.0 equiv) and (+)-*p*-mentha-2,8-dien-1-ol (**19**) (46.98 mg, 0.308 mmol, 1.0 equiv) according to general procedure and purified with flash column chromatography (0% to 5% Et_2O in hexanes). ^{1}H NMR (500 MHz, $CDCl_3$) δ 6.27 (s, 2H), 6.03 (s, 1H), 5.55 – 5.51 (s, 1H), 4.65 (p, *J* = 1.6 Hz, 1H), 4.46 (d, J = 1.9 Hz, 1H), 3.74 (s, 3H), 3.54 (ddq, *J* = 9.1, 4.5, 2.4 Hz, 1H), 2.61 (ddd, *J* = 13.8, 8.9, 6.6 Hz, 1H), 2.49 (ddd, J = 12.8, 9.9, 3.2 Hz, 1H), 2.34 – 2.18 (m, 2H), 2.10 (ddt, J = 17.9, 4.9, 2.4 Hz, 1H), 1.88 – 1.79 (m, 1H), 1.79 (s, 3H), 1.53 (s, 3H), 1.53 – 1.43 (m, 2H), 1.36 – 1.26 (m, 5H), 0.89 (t, J = 7.0 Hz, 3H). ^{13}C{^{1}H} NMR (125 MHz, $CDCl_3$) δ 158.8, 156.6, 147.9, 143.8, 139.9, 124.9, 119.9, 111.6, 107.9, 100.4, 55.2, 45.1, 40.2, 34.4, 32.1, 31.4, 30.4, 28.3, 23.8, 22.7, 21.5, 14.2. $[\alpha]^{25}_D$ =-55.5 (c 0.09, CH_2Cl_2). HRMS (ESI): m/z calcd for $C_{22}H_{33}O_2$ $[M + H]^+$: 329.2481, found 329.2480.

Compound 32

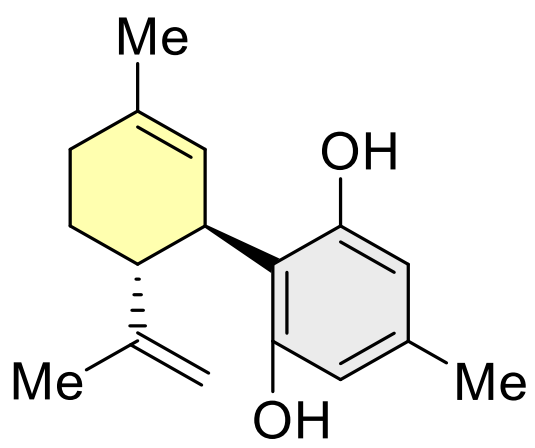

Compound **32** (17.4 mg, 22%, pale yellow oil) was prepared from orcinol (38.3 mg, 0.309 mmol, 1.0 equiv) and (+)-*p*-mentha-2,8-dien-1-ol (**19**) (46.98 mg, 0.309 mmol, 1.0 equiv) according to general procedure and purified with flash column chromatography (1% to 4% Et_2O in hexanes). ^{1}H NMR (500 MHz, $CDCl_3$) δ 6.27 (s, 1H), 6.16 (s, 1H), 5.96 (s, 1H), 5.55 (s, 1H), 4.66 (p, *J* = 1.7 Hz, 1H), 4.56 (s, 1H), 3.86 (ddp, *J* = 11.0, 4.6, 2.4 Hz, 1H), 2.40 (ddd, *J* = 13.6, 10.5, 3.4 Hz, 1H), 2.28 – 2.20 (m, 1H), 2.19 (s, 3H), 2.09 (ddt, *J* = 18.1, 5.2, 2.5 Hz, 1H), 1.86 – 1.80 (m, 1H), 1.79 (s, 3H), 1.66 (s, 3H), 1.59 (s, 1H), 1.40 – 1.28 (m, 1H). ^{13}C{^{1}H} NMR (125 MHz, $CDCl_3$) δ 156.2, 149.3, 140.1, 137.9, 124.1, 113.6, 110.9, 108.7, 46.2, 37.1, 30.4, 28.4, 23.7, 21.1, 20.4. $[\alpha]^{25}_D$ =-62.1 (c 1.0, CH_2Cl_2). HRMS (ESI): m/z calcd for $C_{17}H_{23}O_2$ $[M + H]^+$: 259.1698, found 259.1694.

Compound 33

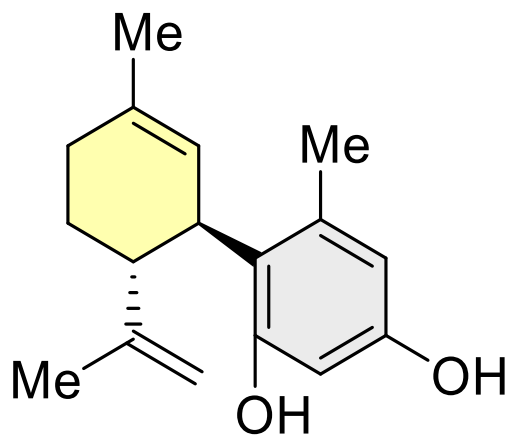

Compound **33** (15.2 mg, 19%, pale yellow oil) was prepared from orcinol (38.3 mg, 0.309 mmol, 1.0 equiv) and (+)-*p*-mentha-2,8-dien-1-ol (**19**) (47.0 mg, 0.309 mmol, 1.0 equiv) according to general procedure and purified with flash column chromatography (10% to 15% Et_2O in hexanes). ^{1}H NMR (500 MHz, $CDCl_3$) δ 6.21 (d, J = 2.7 Hz, 1H), 6.19 (d, *J* = 2.7 Hz, 1H), 6.09 (s, 1H), 5.54 (s, 1H), 4.73 (s, 1H), 4.65 (p, *J* = 1.6 Hz, 1H), 4.47 (d, *J* = 2.0 Hz, 1H), 3.55 (ddp, *J* = 8.7, 4.6, 2.3 Hz, 1H), 2.45 (ddd, *J* = 12.6, 9.9, 3.3 Hz, 1H), 2.28 – 2.17 (m, 1H), 2.15 (s, 3H), 2.09 (ddt, *J* = 17.9, 5.1, 2.5 Hz, 1H), 1.83 (ddt, *J* = 11.5, 5.7, 2.9 Hz, 1H), 1.79 (s, 3H), 1.78 – 1.71 (m, 1H), 1.57 (s, 3H). ^{13}C{^{1}H} NMR (100 MHz, $CDCl_3$) δ 156.5, 154.6, 147.7, 140.0, 139.1, 124.7, 120.6, 111.6, 109.6, 102.3, 45.2, 40.4, 30.3, 28.2, 23.8, 21.2, 21.0. $[\alpha]^{25}_D$ =-67.8 (c 1.05, CH_2Cl_2). HRMS (ESI): m/z calcd for $C_{17}H_{23}O_2$ $[M + H]^+$: 259.1698, found 259.1695.

Compound 34

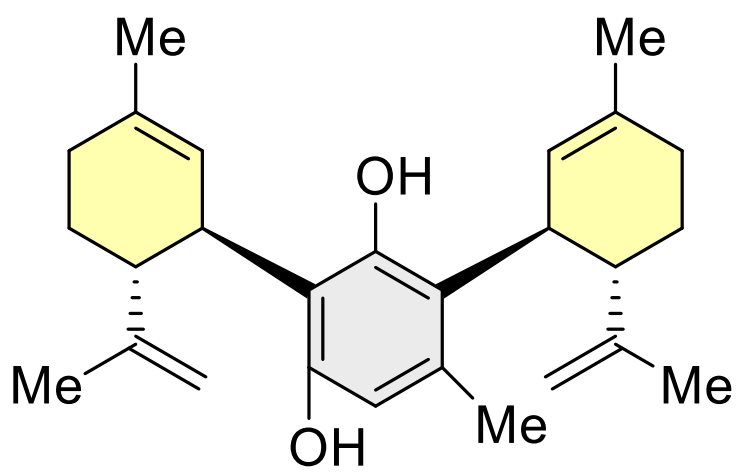

Compound **34** (19 mg, 16%, pale yellow oil) was prepared from orcinol (38.3 mg, 0.309 mmol, 1.0 equiv) and (+)-*p*-mentha-2,8-dien-1-ol (**19**) (47.0 mg, 0.309 mmol, 1.0 equiv) according to general procedure and purified with flash column chromatography (1% to 1.5% Et_2O in hexanes). ^{1}H NMR (500 MHz, $CDCl_3$) δ 6.21 (s, 1H), 5.95 (s, 1H), 5.77 (s, 1H), 5.55 (s, 1H), 5.51 (s, 1H), 4.61 (t, *J* = 1.8 Hz, 1H), 4.51 (t, *J* = 2.0 Hz, 1H), 4.45 (s, 1H), 4.43 (s, 1H), 4.00 (d, *J* = 8.5 Hz, 1H), 3.67 – 3.37 (m, 1H), 2.43 (dt, *J* = 20.1, 7.6 Hz, 2H), 2.29 – 2.17 (m, 2H), 2.11 (s, 3H), 2.06 (p, J = 3.7, 3.0 Hz, 2H), 1.80 (dd, J = 6.9, 4.9 Hz, 2H), 1.78 (s, 3H), 1.76 (s, 3H), 1.70 (s, 3H), 1.66 – 1.55 (m, 2H), 1.53 (s, 3H). ^{13}C{^{1}H} NMR (125 MHz, $CDCl_3$) δ 154.0, 153.7, 147.8, 147.7, 139.8, 139.2, 136.3, 124.8, 119.7, 115.4, 111.4, 111.3, 110.4, 46.6, 44.5, 40.8, 35.7, 30.5, 30.4, 29.7, 28.5, 28.3, 23.7, 23.7, 21.3, 20.8, 18.9. $[\alpha]^{25}_D$ =-61.7 (c 0.09, CH_2Cl_2). HRMS (ESI): m/z calcd for $C_{27}H_{37}O_2$ $[M + H]^+$: 393.2794, found 393.2790.

Compound 35

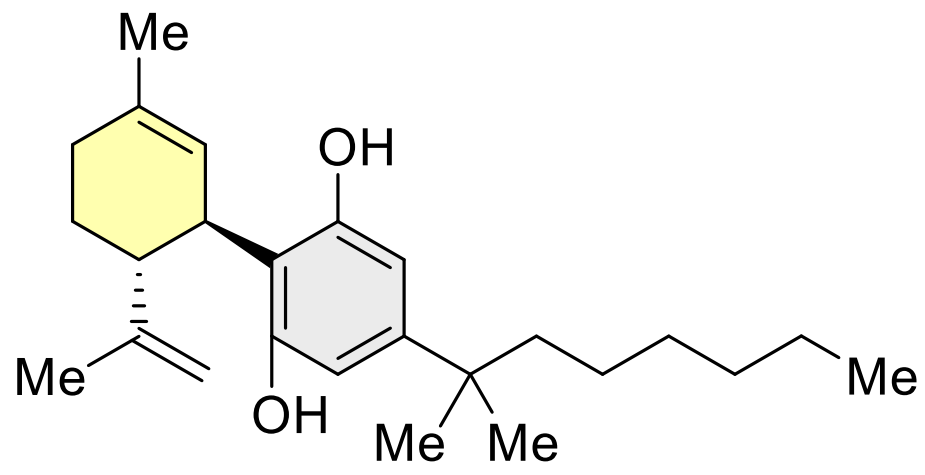

Compound **35** (107 mg, 94%, pale yellow oil) was prepared from 5-(2-methyloctan-2-yl)benzene-1,3-diol (72.9 mg, 0.309 mmol, 1.0 equiv) and (+)-*p*-mentha-2,8-dien-1-ol (**19**) (47.0 mg, 0.309 mmol, 1.0 equiv) according to general procedure and purified with flash column chromatography (0% to 10% Et_2O in hexanes). ^{1}H NMR (500 MHz, $CDCl_3$) δ 6.45 – 6.23 (m, 2H), 5.97 (s, 1H), 5.59 (s, 1H), 4.67 (t, *J* = 1.7 Hz, 1H), 4.56 (s, 1H), 3.82 (ddd, *J* = 10.0, 4.2, 2.2 Hz, 1H), 2.38 (ddd, J = 13.5, 10.5, 3.4 Hz, 1H), 2.31 – 2.18 (m, 1H), 2.10 (ddt, J = 18.0, 5.0, 2.4 Hz, 1H), 1.80 (s, 3H), 1.68 (s, 2H), 1.64 (s, 3H), 1.55 – 1.45 (m, 2H), 1.43 (d, *J* = 5.1 Hz, 1H), 1.20 (s, 3H), 1.20 (s, 3H), 1.18 – 1.13 (m, 4H), 1.09 (d, *J* = 8.8 Hz, 1H), 1.00 (dddd, *J* = 12.3, 7.4, 5.1, 2.4 Hz, 2H), 0.84 (t, *J* = 7.0 Hz, 3H). ^{13}C{^{1}H} NMR (125 MHz, $CDCl_3$) δ 155.9, 153.9, 150.4, 149.7, 140.2, 124.2, 113.6, 110.9, 107.8, 106.0, 46.2, 44.7, 37.7, 37.5, 32.0, 30.6, 28.9, 28.8, 28.5, 24.8, 23.8, 22.8, 20.9, 14.2. $[\alpha]^{25}_D$ =-54.8 (c 1.2, CH_2Cl_2). HRMS (ESI): m/z calcd for $C_{25}H_{39}O_2$ [M + H]$^+$: 371.2950, found 371.2947.

Compound 36

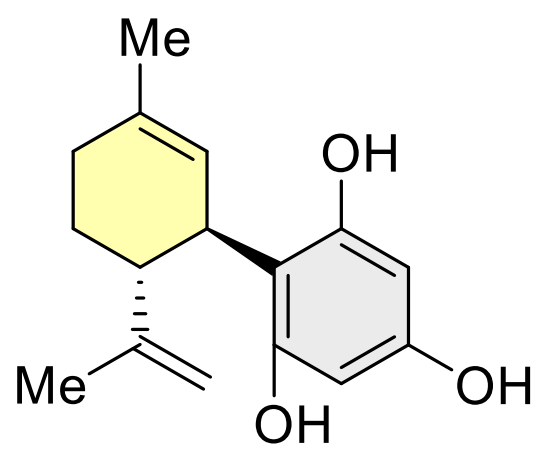

Phloroglucinol (38.9 mg, 0.309 mmol, 1.0 equiv) and $FeCl_3{\cdot}6H_2O$ (8.34 mg, 0.031 mmol, 0.1 equiv) were dissolved in THF (28 mL) under ultrasound at 23 ºC for 5 min. A solution of (+)-*p*-mentha-2,8-dien-1-ol (**19**) (46.9 mg, 0.309 mmol, 1.0 equiv) in THF (14 mL) was then added dropwise via a dropping funnel. After complete addition, the reaction mixture was stirred at 23 ºC for 72 h. After this time, the crude reaction mixture was subsequently quenched with a aqueous buffer (pH 7) solution, extracted with EtOAc (x3), and the combined organic layers were washed with brine, dried over anhydrous $MgSO_4$, filtered and concentrated under reduced pressure. The crude mixture was purified by column chromatography (1% to 8% Et_2O in hexanes) to obtain the corresponding product **36** (10.2 mg, 13%, pale yellow oil). Alternatively, the reaction was carried out using CH_3CN as solvent, affording **36** in higher yield (35 mg, 44%, pale yellow oil). ^{1}H NMR (500 MHz, $CDCl_3$) δ 6.09 (s, 1H), 5.96 (s, 1H), 5.89 (s, 1H), 5.54 (s, 1H), 4.85 (bs, 2H), 4.68 (s, 1H), 4.58 (s, 1H), 3.78 (ddt, *J* = 10.3, 4.0, 2.3 Hz, 1H), 2.35 (ddd, *J* = 11.6, 10.3, 3.4 Hz, 1H), 2.26 – 2.18 (m, 1H), 2.09 (ddt, J = 17.9, 4.9, 2.4 Hz, 1H), 1.85 – 1.74 (m, 5H), 1.66 (s, 3H). ^{13}C{^{1}H} NMR (125 MHz, $CDCl_3$) δ 155.3, 149.7, 140.5, 124.3, 111.0, 109.4, 97.4, 95.9, 46.5, 37.3, 30.6, 28.6, 23.8, 20.7. $[\alpha]^{25}_D$ =-62.6(c 1.0, CH_2Cl_2). HRMS (ESI): m/z calcd for $C_{16}H_{21}O_3$ [M + H]$^+$: 261.1491, found 261.1489.

Compound 37

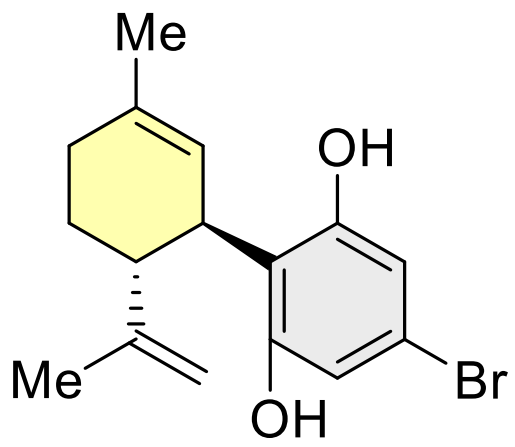

Compound **37** (4.6 mg, 4.6%, pale yellow oil) was prepared from 5-bromobenzene-1,3-diol (58.3 mg, 0.309 mmol, 1.0 equiv) and (+)-*p*-mentha-2,8-dien-1-ol (**19**) (47.0 mg, 0.309 mmol, 1.0 equiv) according to general procedure and purified with flash column chromatography (100% hexanes to 5% Et_2O in hexanes). Alternatively, compound **37** was also prepared with slight modification protocol (reaction time extended to 24 h), yielding 10.3 mg, 10.3%, pale yellow oil. ^{1}H NMR (500 MHz, $CDCl_3$) δ 6.61 (s, 1H), 6.50 (s, 1H), 6.13 (s, 1H), 5.52 (s, 1H), 4.99 (s, 1H), 4.65 (p, *J* = 1.6 Hz, 1H), 4.53 (dd, *J* = 1.8, 1.0 Hz, 1H), 3.85 (ddq, *J* = 10.7, 4.5, 2.3 Hz, 1H), 2.37 (td, *J* = 11.3, 10.7, 3.3 Hz, 1H), 2.22 (ddtd, *J* = 12.3, 5.8, 2.6, 1.3 Hz, 1H), 2.10 (ddt, *J* = 18.0, 4.9, 2.4 Hz, 1H), 1.87 – 1.75 (m, 5H), 1.66 (s, 3H). ^{13}C{^{1}H} NMR (125 MHz, $CDCl_3$) δ 157.1, 154.9, 148.8, 141.1, 123.4, 120.1, 116.0, 113.5, 111.5, 111.3, 46.2, 37.1, 30.5, 28.4, 23.8, 20.3. $[\alpha]^{25}_D$ =-58.9 (c 1.1, CH_2Cl_2). HRMS (ESI): m/z calcd for $C_{16}H_{20}BrO_2$ [M + H]$^+$: 323.0647, found 323.0645.

Compound 38

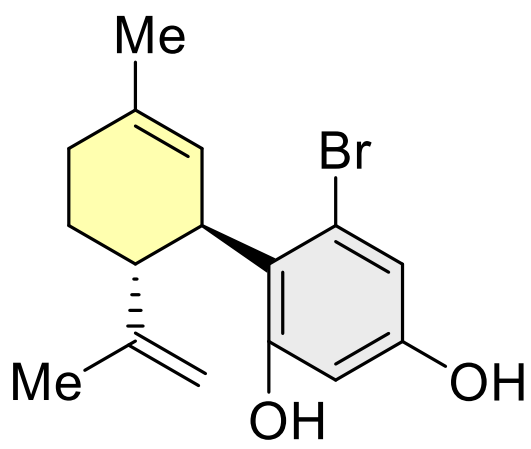

Compound **38** (33.4 mg, 33%, pale yellow oil) was prepared from 5-bromobenzene-1,3-diol (58.3 mg, 0.309 mmol, 1.0 equiv) and (+)-*p*-mentha-2,8-dien-1-ol (**19**) (47.0 mg, 0.309 mmol, 1.0 equiv) according to general procedure with slight modification (reaction time extended to 24 h), and purified with flash column chromatography (10% to 15% ethyl acetate in hexanes). ^{1}H NMR (500 MHz, $CDCl_3$) δ 6.62 (d, *J* = 2.6 Hz, 1H), 6.31 (d, *J* = 2.6 Hz, 1H), 6.27 (s, 1H), 5.59 – 5.49 (m, 1H), 5.00 (br s, 1H), 4.62 (p, *J* = 1.6 Hz, 1H), 4.37 (d, *J* = 2.1 Hz, 1H), 3.97 (dqt, *J* = 8.6, 4.6, 2.1 Hz, 1H), 2.49 (td, *J* = 10.5, 4.0 Hz, 1H), 2.29 – 2.16 (m, 1H), 2.10 (ddt, *J* = 18.1, 5.3, 2.6 Hz, 1H), 1.85 – 1.78 (m, 5H), 1.70 (s, 3H). ^{13}C{^{1}H} NMR (100 MHz, $CDCl_3$) δ 156.5, 155.2, 146.3, 140.7, 126.2, 123.4, 121.5, 112.5, 112.2, 104.1, 45.5, 43.4, 30.1, 27.5, 23.7, 20.0. $[\alpha]^{25}_D$ =-57.3 (c 0.09, CH_2Cl_2). HRMS (ESI): m/z calcd for $C_{16}H_{20}BrO_2$ [M + H]$^+$: 323.0647, found 323.0642.

Compound 39

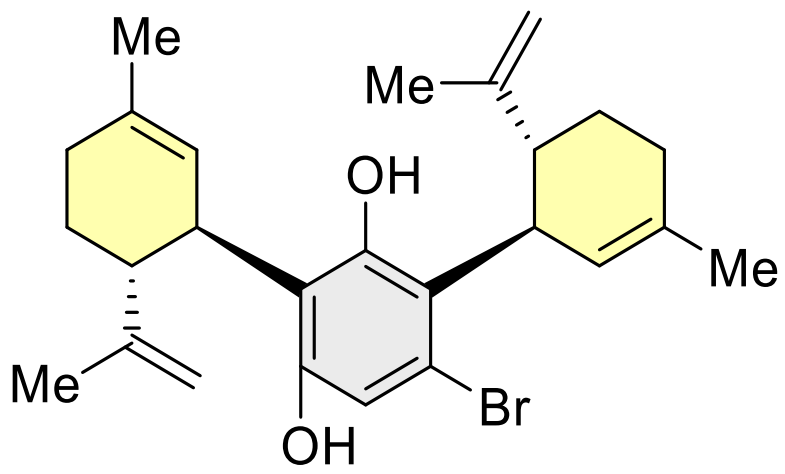

Compound **39** (2.0 mg, 1.4%, pale yellow oil) was prepared from 5-bromobenzene-1,3-diol (58.3 mg, 0.309 mmol, 1.0 equiv) and (+)-*p*-mentha-2,8-dien-1-ol (**19**) (47.0 mg, 0.300 mmol, 1.0 equiv) according to general procedure with slight modification (reaction time extended to 24 h), and purified with flash column chromatography (1% to 4% Et_2O in hexanes). ^{1}H NMR (500 MHz, $CDCl_3$) δ 6.63 (s, 1H), 6.16 (s, 1H), 5.90 (s, 1H), 5.51 (s, 2H), 4.58 (t, *J* = 2.0 Hz, 1H), 4.54 (s, 1H), 4.45 (s, 1H), 4.34 (s, 1H), 4.05 – 3.90 (m, 2H), 2.51 – 2.33 (m, 2H), 2.32 – 2.02 (m, 3H), 2.17 – 2.02 (m, 3H), 1.82 – 1.75 (m, 8H), 1.70 (s, 3H), 1.67 (s, 3H). ^{13}C{^{1}H} NMR (125 MHz, $CDCl_3$) δ 154.9, 154.0, 147.2, 146.5, 140.8, 140.2, 124.1, 123.9, 123.8, 120.6, 117.5, 113.3, 112.7, 111.8, 46.5, 45.2, 44.0, 35.9, 30.6, 30.4, 29.9, 28.5, 27.9, 23.9, 23.8, 20.1. HRMS (ESI): m/z calcd for $C_{26}H_{34}BrO_2$ $[M + H]^+$: 457.1742, found 457.1739.

Compound 41

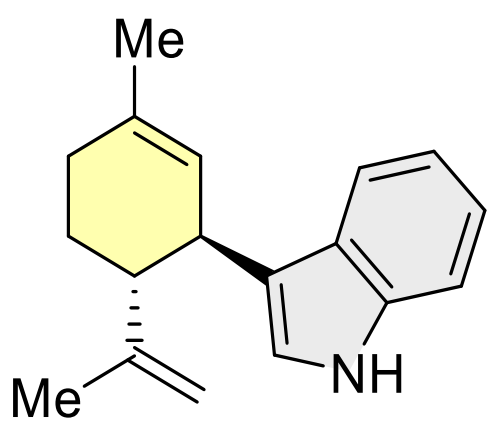

Compound **41** (44 mg, 57%, pale yellow oil) was prepared from (+)-*p*-mentha-2,8-dien-1-ol (**19**) (46.9 mg, 0.309 mmol, 1.0 equiv) and indole (36.2 mg, 0.309 mmol, 1.0 equiv) according to general procedure and purified with flash column chromatography (2% to 5% Et_2O in hexanes). ^{1}H NMR (500 MHz, $CDCl_3$) δ 7.76 (bs, 1H), 7.54 (d, *J* = 7.8 Hz, 1H), 7.24 (d, *J* = 8.1 Hz, 1H), 7.08 (ddd, *J* = 8.2, 7.0, 1.2 Hz, 1H), 7.00 (ddd, *J* = 7.9, 6.9, 1.1 Hz, 1H), 6.84 (d, *J* = 2.2 Hz, 1H), 5.43 (dh, *J* = 3.1, 1.4 Hz, 1H), 4.59 – 4.54 (m, 1H), 4.53 (s, 1H), 3.57 (dq, *J* = 8.2, 2.5 Hz, 1H), 2.46 (ddd, *J* = 10.4, 8.9, 3.3 Hz, 1H), 2.12 – 2.05 (m, 1H), 2.01 – 1.92 (m, 1H), 1.76 – 1.71 (m, 1H), 1.70 – 1.67 (m, 1H), 1.64 (s, 3H), 1.55 (t, *J* = 1.1 Hz, 3H). ^{13}C{^{1}H} NMR (125 MHz, $CDCl_3$) δ 148.8, 136.6, 133.2, 128.4, 126.8, 125.4, 121.7, 119.7, 118.9, 117.2, 111.1, 110.4, 47.3, 37.7, 30.0, 28.1, 23.5, 20.5. $[\alpha]^{25}_D$ =-66.1 (c 1.10, CH_2Cl_2). HRMS (ESI): m/z calcd for $C_{18}H_{22}N$ $[M + H]^+$: 252.1752, found 252.1749.

Compound 50

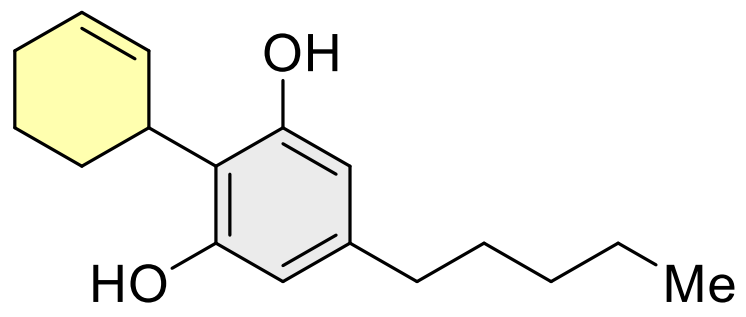

50

Compound **50** (15 mg, 19%, pale yellow oil) was prepared from olivetol (**18**) (55.6 mg, 0.309 mmol, 1.0 equiv) and 2-cyclohexen-1-ol (**45**) (30.3 mg, 0.309 mmol, 1.0 equiv) according to general procedure and purified with flash column chromatography (1% to 5% Et_2O in hexanes). ^{1}H NMR (500 MHz, $CDCl_3$) δ 6.23 (s, 2H), 6.20 – 6.12 (m, 1H), 5.97 – 5.90 (m, 1H), 5.42 (s, 2H), 4.04 – 3.83 (m, 1H), 2.54 – 2.39 (m, 2H), 2.16 (dt, *J* = 5.2, 2.7 Hz, 2H), 2.00 (dt, *J* = 12.4, 6.2 Hz, 1H), 1.88 (dq, *J* = 9.3, 5.1, 4.6 Hz, 1H), 1.72 – 1.65 (m, 2H), 1.60 – 1.52 (m, 2H), 1.34 – 1.30 (m, 4H), 0.89 (t, *J* = 6.9 Hz, 3H). ^{13}C{^{1}H} NMR (125 MHz, CDCl3) δ 155.2, 143.2, 133.0, 130.4, 114.2, 108.7, 35.6, 32.2, 31.7, 30.8, 28.8, 25.1, 22.7, 22.2, 14.2. HRMS (ESI): m/z calcd for $C_{17}H_{25}O_2$ $[M + H]^+$: 261.1855, found 261.1853

Compound 51 and 52

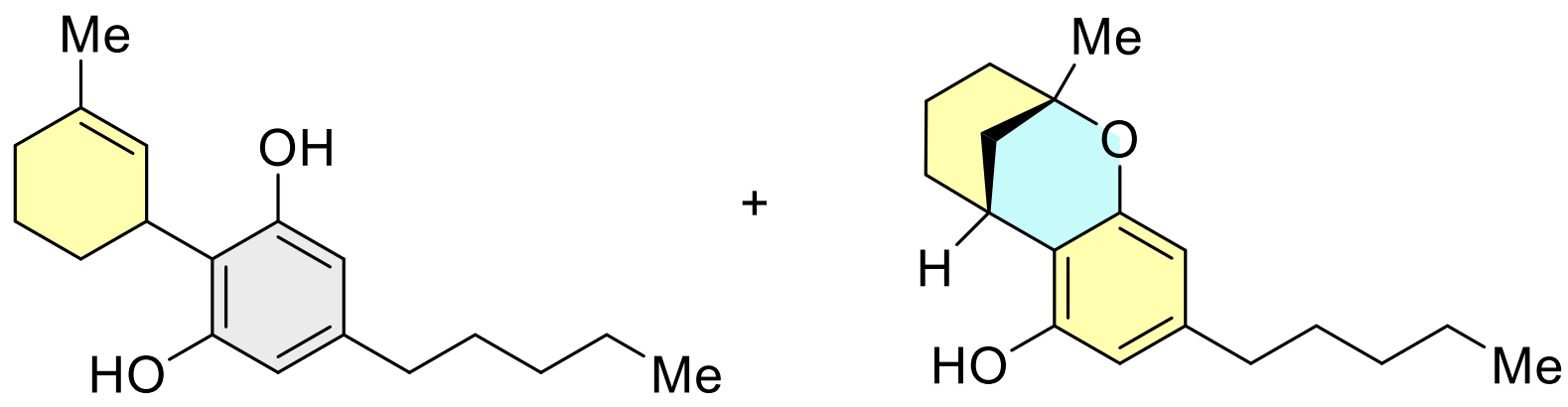

51 **52**

Compounds **51** and **52** (**51**:**52** ~2:1 inseparable mixture, 42 mg, 50% combined yield, pale yellow oil) were prepared from olivetol (**18**) (55.6 mg, 0.309 mmol, 1.0 equiv) and 1-methylcyclohex-2-en-1-ol (35 mg, 0.309 mmol, 1.0 equiv) according to general procedure and purified with flash column chromatography (3% to 6% Et_2O in hexanes). For the mixture of [**51**:**52**]: ^{1}H NMR (500 MHz, $CDCl_3$) δ 6.27 (d, *J* = 1.4 Hz, 1H), 6.22 (s, 4H), 6.13 (d, *J* = 1.5 Hz, 1H), 5.64 (tt, *J* = 2.5, 1.3 Hz, 2H), 3.89 (ddp, *J* = 11.6, 5.7, 2.8 Hz, 2H), 3.29 (t, *J* = 3.2 Hz, 1H), 2.45 (td, *J* = 7.9, 2.9 Hz, 5H), 2.20 – 2.08 (m, 2H), 2.07 – 1.97 (m, 2H), 1.99 – 1.82 (m, 5H), 1.82 – 1.72 (m, 8H), 1.72 – 1.63 (m, 2H), 1.64 – 1.52 (m, 10H), 1.51 – 1.43 (m, 2H), 1.34 (s, 3H), 1.30 (ddd, *J* = 11.4, 6.4, 2.7 Hz, 9H), 0.92 – 0.86 (m, 9H). ^{13}C{^{1}H} NMR (125 MHz, $CDCl_3$) δ 157.6, 152.4, 143.1, 142.7, 141.2, 123.9, 114.5, 110.04, 108.63, 107.99, 106.16, 74.62, 39.40, 36.19, 35.85, 35.62, 32.37, 31.73, 31.69, 30.92, 30.9, 30.3, 30.1, 29.6, 28.5, 26.5, 24.3, 22.7, 22.5, 18.9, 14.2. Formation of mixture of **51** and **52** has been reported previously from the reaction of olivetol with 1-methylcyclohex-2-en-1-ol under either Lewis or Brønsted acid catalysis.[22]

Compound 53 and Δ^8-THC (8)

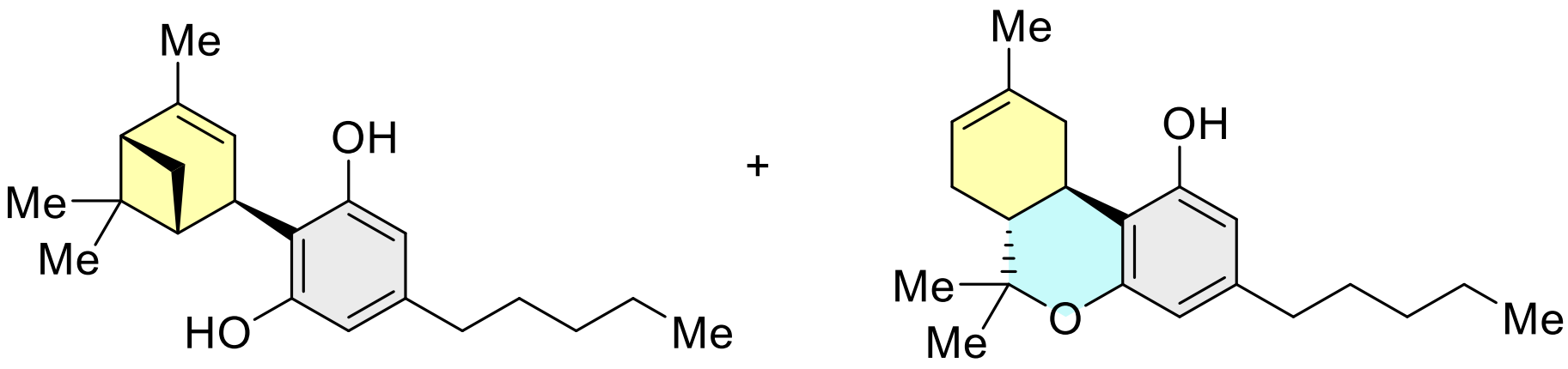

53 **Δ^8-THC (8)**

Compounds **53** and **Δ^8-THC (8)** (**53**:**8** ~1:3 inseparable mixture, 86 mg, 88% combined yield, pale yellow oil) were prepared from olivetol (18) (55.6 mg, 0.309 mmol, 1.0 equiv) and (*S*)-*cis*-verbenol (47.0 mg, 0.309 mmol, 1.0 equiv) according to general procedure and purified with flash column chromatography (7% Et_2O in hexanes). For the mixture of [**53**:**8**]: ^{1}H NMR (500 MHz, $CDCl_3$) δ 6.27 (d, *J* = 1.6 Hz, 1H), 6.22 – 6.15 (m, 0.75H), 6.11 (d, *J* = 1.6 Hz, 1H), 5.70 (dq, *J* = 3.1, 1.6 Hz, 0.32H), 5.43 (dd, *J* = 5.3, 3.1 Hz, 1H), 4.70 (s, 1H), 3.92 (q, *J* = 2.6 Hz, 0.34H), 3.24 – 3.14 (m, 1H), 2.70 (td, *J* = 10.9, 4.7 Hz, 1H), 2.44 (td, *J* = 7.6, 3.6 Hz, 3H), 2.36 – 2.25 (m, 1H), 2.20 – 2.11 (m, 2H), 1.88 – 1.75 (m, 4H), 1.70 (d, *J* = 2.0 Hz, 3H), 1.37 (s, 3H), 1.36 – 1.24 (m, 9H), 1.10 (s, 3H), 0.93 – 0.85 (m, 7H). ^{13}C{^{1}H} NMR (125 MHz, $CDCl_3$) δ 154.9, 154.8, 152.6, 143.1, 142.7, 134.8, 125.5, 123.7, 119.3, 116.5, 112.2, 110.5, 110.1, 107.6, 65.9, 47.9, 47.1, 44.9, 40.7, 37.8, 36.0, 35.5, 35.4, 31.6, 30.7, 30.6, 30.4, 30.3, 29.7, 28.9, 28.4, 27.9, 27.9, 27.6, 26.0, 23.8, 23.7, 23.5, 23.4, 23.0, 22.6, 22.4, 22.0, 18.5, 15.3, 14.1, 14.0. Data is in agreement with literature.[23,1]

Compound 54 and 1'1-DMH-Δ^8-THC (55)

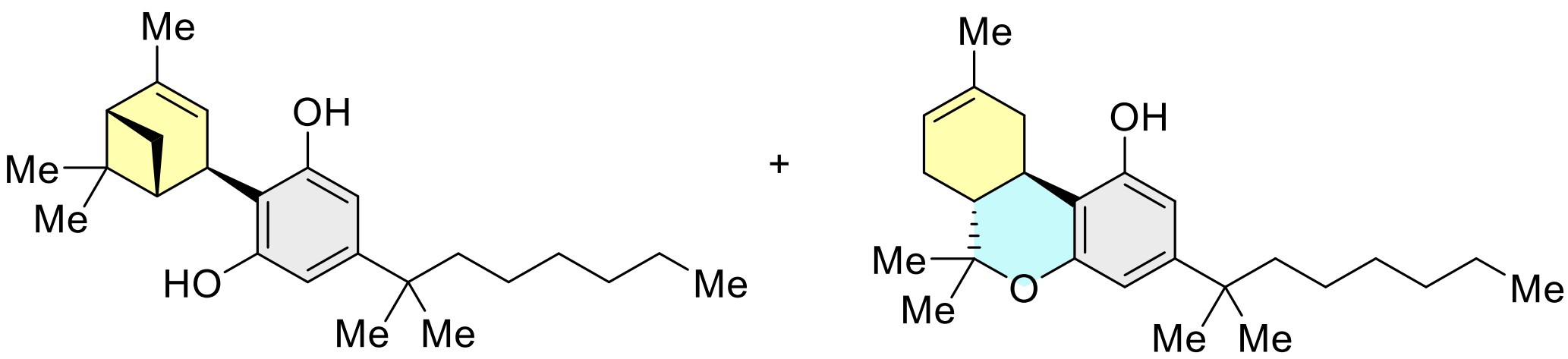

54 **1'1-DMH-Δ^8-THC (55)**

Compounds **54** and **1'1-DMH-Δ^8-THC** (**55**) (**54**:**55** ~1:2 inseparable mixture, 77.7 mg, 90% combined yield, pale yellow oil) were prepared from 5-(2-methyloctan-2-yl)benzene-1,3-diol (55 mg, 0.233 mmol, 1.0 equiv) and (*S*)-*cis*-verbenol (35 mg, 0.233 mmol, 1.0 equiv) according to general procedure and purified with flash column chromatography (1% to 8% Et_2O in hexanes). For the mixture of [**54:55**]: ^{1}H NMR (500 MHz, CD_2Cl_2) δ 6.39 (d, *J* = 1.8 Hz, 1H), 6.31 (s, 1H), 6.23 (d, *J* = 1.8 Hz, 1H), 5.71 (dt, *J* = 3.2, 1.5 Hz, 0.5H), 5.43 (dd, *J* = 5.0, 2.9 Hz, 1H), 5.15 – 5.08 (m, 1H), 4.63 (s, 1H), 3.91 (q, *J* = 2.6 Hz, 0.5H), 3.19 (dd, *J* = 16.7, 4.8 Hz, 1H), 2.69 (td, *J* = 11.1, 4.9 Hz, 1H), 2.32 (dt, *J* = 9.6, 5.6 Hz, 1H), 2.29 – 2.25 (m, 1H), 2.20 – 2.11 (m, 1H), 2.04 (h, *J* = 5.8 Hz, 3H), 1.89 – 1.76 (m, 3H), 1.70 (d, *J* = 1.8 Hz, 4H), 1.68 (s, 2H), 1.50 (ddd, *J* = 10.7, 6.3, 2.2 Hz, 4H), 1.39 (s, 3H), 1.32 (s, 2H), 1.23 – 1.15 (m, 19H), 0.89 – 0.81 (m, 7H). ^{13}C{^{1}H} NMR (125 MHz, CD_2Cl_2) δ 154.5, 154.4, 152.6, 150.4, 150.0, 135.2, 134.8, 125.0, 119.4, 116.6, 111.8, 110.2, 108.1, 105.4, 65.9, 48.4, 48.0, 47.0, 44.9, 44.5, 44.4, 40.7, 37.8, 37.4, 37.3, 37.3, 36.0, 32.2, 31.8, 31.8, 31.5, 30.3, 30.1, 30.0, 29.7, 28.8, 28.7, 28.7, 28.7, 28.0, 27.9, 27.6, 26.4, 26.0, 24.7, 24.6, 23.8, 23.5, 23.4, 22.7, 22.7, 20.5, 18.5, 14.1. Data is in agreement with literature.[24]

Compound 55 (1'1-DMH-Δ^8-THC)

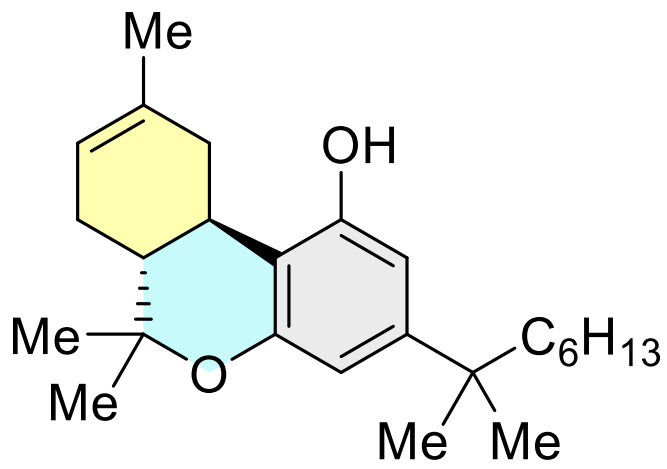

From 1'1-DMH-Δ^9-THC (**57**): 1'1-DMH-Δ^9-THC (**57**) (200 mg, 0.540 mmol, 1.0 equiv) and $FeCl_3·6H_2O$ (88 mg, 0.324 mmol, 0.6 equiv) were dissolved in CH_2Cl_2 (9 mL) and the resulting reaction mixture was stirred at 23 ºC for 24 h. After this time, the reaction was quenched with a saturated aqueous $NaHCO_3$ solution. After separation of both phases, the aqueous phase was extracted with CH_2Cl_2 (x3). The organic phases were collected and washed sequentially with water and brine and concentrated under reduced pressure. The crude mixture was purified by flash column chromatography (silica gel, 4 % Et_2O in hexanes) to obtain 1'1-DMH-Δ^8-THC (**55**) (166 mg, 83 % yield) as a pale yellow oil.

From 5-(1,1-DMH)resorcinol (**56**): A solution of (+)-*p*-mentha-2,8-dien-1-ol (**19**) (64 mg, 0.423, 1.0 equiv) in CH_2Cl_2 (20 mL) was added dropwise to a solution of 5-(1,1- DMH)resorcinol (**56**) (100 mg, 0.423 mmol, 1.0 equiv) and $FeCl_3·6H_2O$ (137 mg, 0.501 mmol, 1.2 eq.) in CH_2Cl_2 (4 mL) at 23 ºC for 24 h. After this time, the reaction was quenched with a saturated aqueous $NaHCO_3$ solution. After separation of both phases, the aqueous phase was extracted with CH_2Cl_2 (x3). The organic phases were collected and washed sequentially with water and brine and concentrated under reduced pressure. The crude mixture was purified by flash column chromatography (silica gel, 4 % Et_2O in hexanes) to obtain 1'1-DMH-Δ^8-THC (**55**) (94 mg, 60 % yield) as a as a pale yellow oil.

Compound 1'1-DMH-Δ^8-THC (**55**): ^{1}H NMR (500 MHz, $CDCl_3$) δ 6.39 (d, J = 1.8 Hz, 1H), 6.23 (d, J = 1.9 Hz, 1H), 5.47 – 5.38 (m, 1H), 4.76 (s, 1H), 3.20 (dd, *J* = 17.3, 5.2 Hz, 1H), 2.70 (td, *J* = 11.0, 4.9 Hz, 1H), 2.19 – 2.11 (m, 1H), 1.94 – 1.77 (m, 3H), 1.71 (s, 3H), 1.49 (ddd, *J* = 10.6, 5.7, 1.9 Hz, 2H), 1.39 (s, 3H), 1.30 – 1.20 (m, 6H), 1.20 (s, 6H), 1.12 (s, 3H), 1.10 – 1.04 (m, 2H), 0.85 (t, *J* = 7.0 Hz, 3H). ^{13}C{^{1}H} NMR (125 MHz, $CDCl_3$) δ 154.6, 154.6, 150.2, 134.9, 119.5, 110.3, 108.1, 105.6, 45.0, 44.6, 37.4, 36.1, 31.9, 31.6, 30.2, 28.9, 28.8, 28.0, 27.8, 27.1, 24.8, 23.7, 22.8, 18.7, 14.3. $[\alpha]^{25}_D$ =-64.2 (c 0.09, CH_2Cl_2). HRMS (ESI): m/z calcd for $C_{25}H_{39}O_2$ $[M + H]^+$: 371.2950, found 371.2948.

Compound 57 (1'1-DMH-Δ⁹-THC)

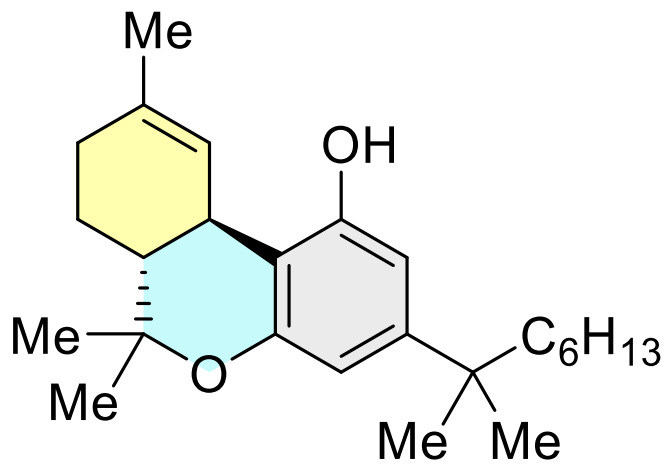

1',1'-DMH-CBD (**35**) (500 mg, 1.349 mmol, 1.0 eq.) was dissolved in CH_2Cl_2 (8 mL) and $FeCl_3 \cdot 6H_2O$ (219 mg, 0.809 mmol, 0.6 equiv) was added at 23 ºC for 2.5 h. After this time, the reaction was quenched with a saturated aqueous $NaHCO_3$ solution. After separation of both phases, the aqueous phase was extracted with CH_2Cl_2 (x3). The combined organic phases were sequentially washed with water and brine and concentrated under reduced pressure. The crude mixture was purified by flash column chromatography (silica gel, 4 % Et_2O in hexanes) to obtain 1'1-DMH-Δ⁹-THC (**57**) (450 mg, 90 %) as a pale yellow oil. ^{1}H NMR (500 MHz, $CDCl_3$) δ 6.39 (d, *J* = 1.9 Hz, 1H), 6.32 (p, *J* = 1.7 Hz, 1H), 6.26 (d, *J* = 1.8 Hz, 1H), 4.72 (s, 1H), 3.20 (dp, *J* = 10.6, 2.6 Hz, 1H), 2.20 – 2.14 (m, 2H), 1.96 – 1.87 (m, 2H), 1.74 – 1.70 (m, 1H), 1.68 (s, 3H), 1.57 (s, 3H), 1.52 – 1.46 (m, 2H), 1.42 (s, 3H), 1.27 – 1.21 (m, 9H), 1.10 (s, 3H), 1.10 – 1.02 (m, 2H), 0.84 (t, J = 7.0 Hz, 3H). ^{13}C{^{1}H} NMR (100 MHz, CDCl3) δ 154.5, 153.9, 150.1, 134.5, 123.7, 108.7, 108.0, 105.3, 45.8, 44.5, 37.5, 33.6, 31.8, 31.2, 29.8, 28.8, 27.6, 25.0, 24.6, 23.4, 22.7, 19.3, 14.1. $[\alpha]^{25}_D$ =-64.9 (c 0.09, CH_2Cl_2). HRMS (ESI): m/z calcd for $C_{25}H_{39}O_2$ $[M + H]^+$: 371.2950, found 371.2946.

8. ^{1}H, ^{13}C{^{1}H} NMR Spectra

Compound S1. ^{1}HNMR (500 MHz, $CDCl_3$) and ^{13}C{^{1}H} NMR (125 MHz, $CDCl_3$).

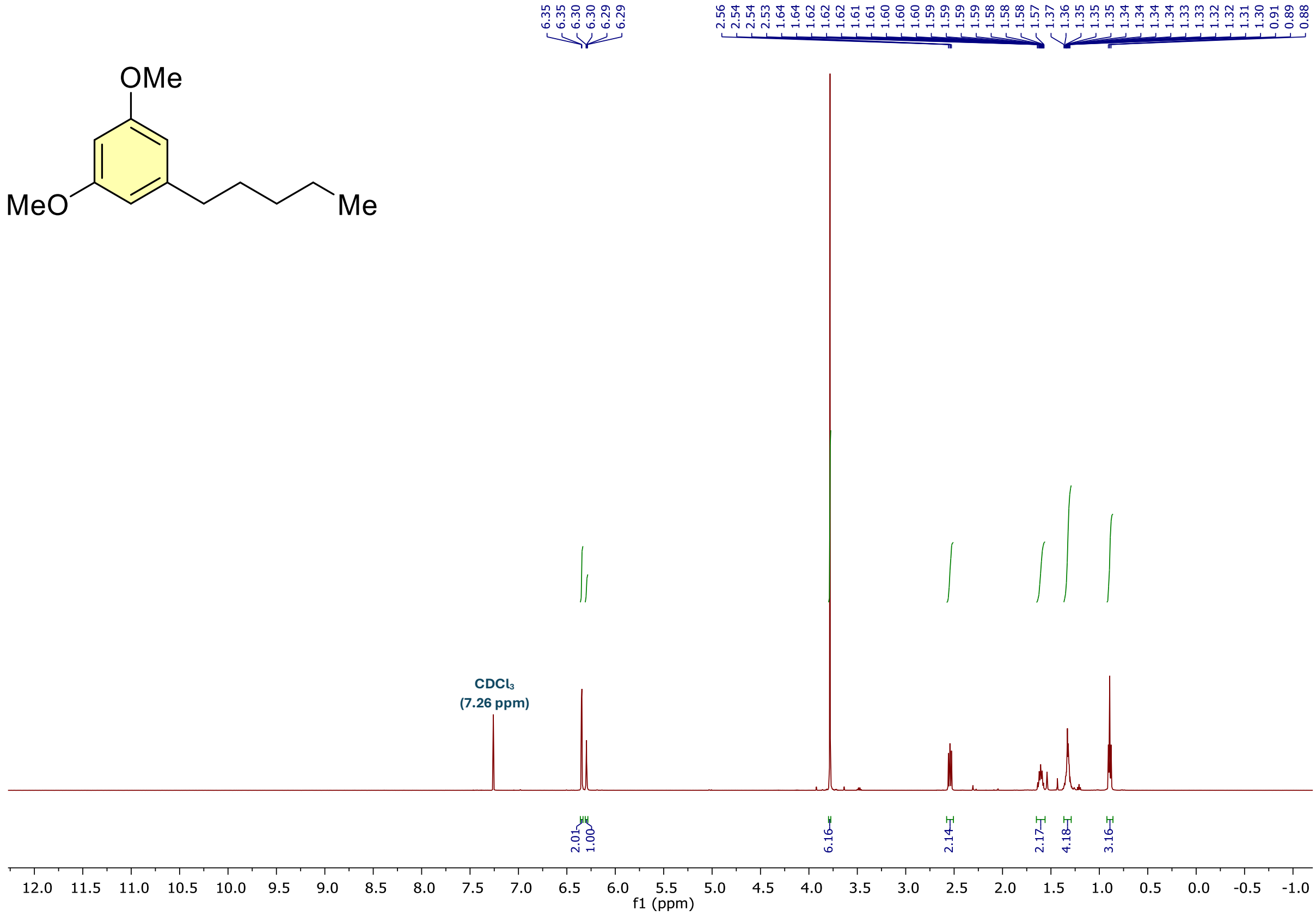

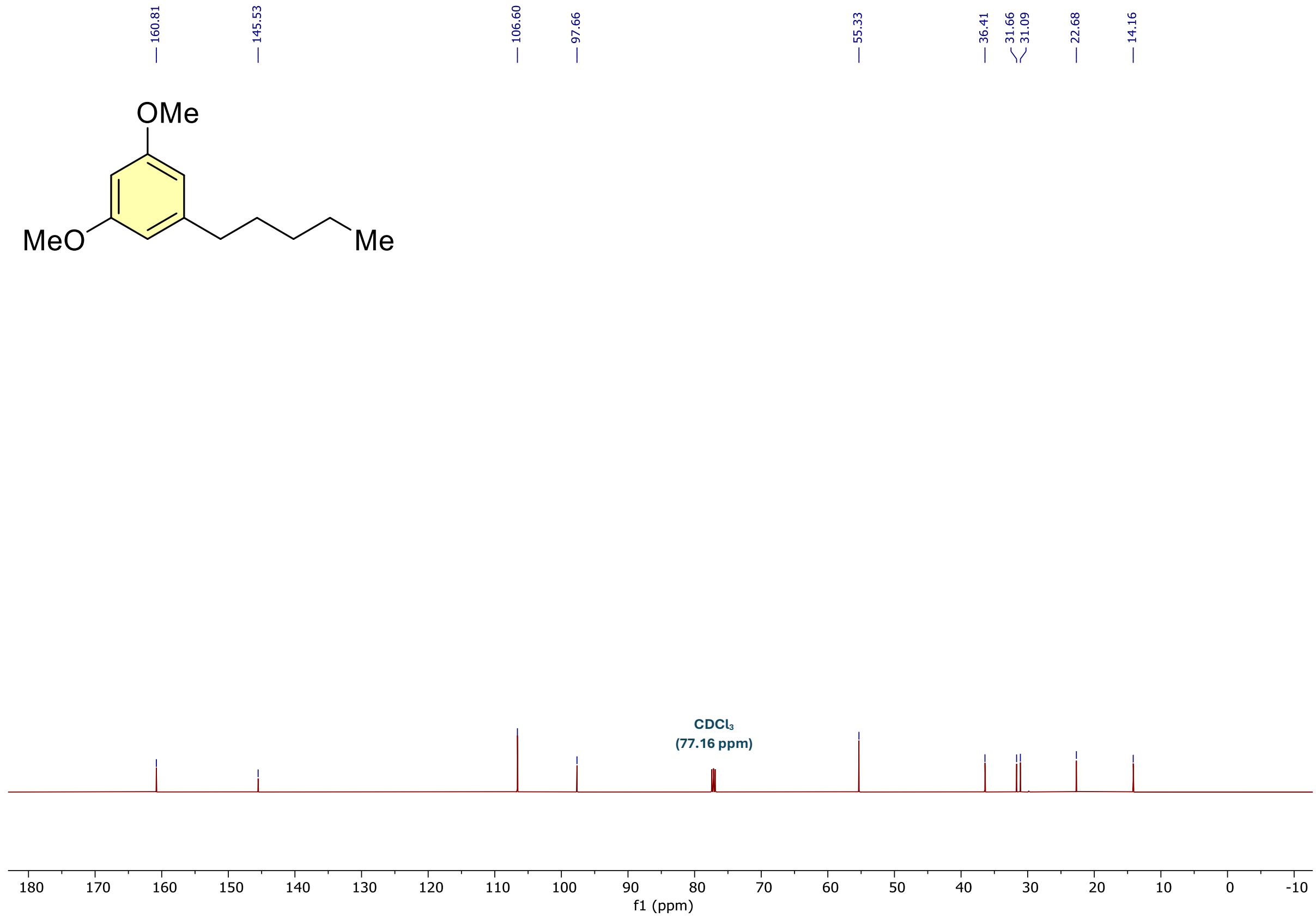

Compound 27. ^{1}HNMR (500 MHz, $CDCl_3$) and $^{13}C\{^{1}H\}$ NMR (125 MHz, $CDCl_3$).

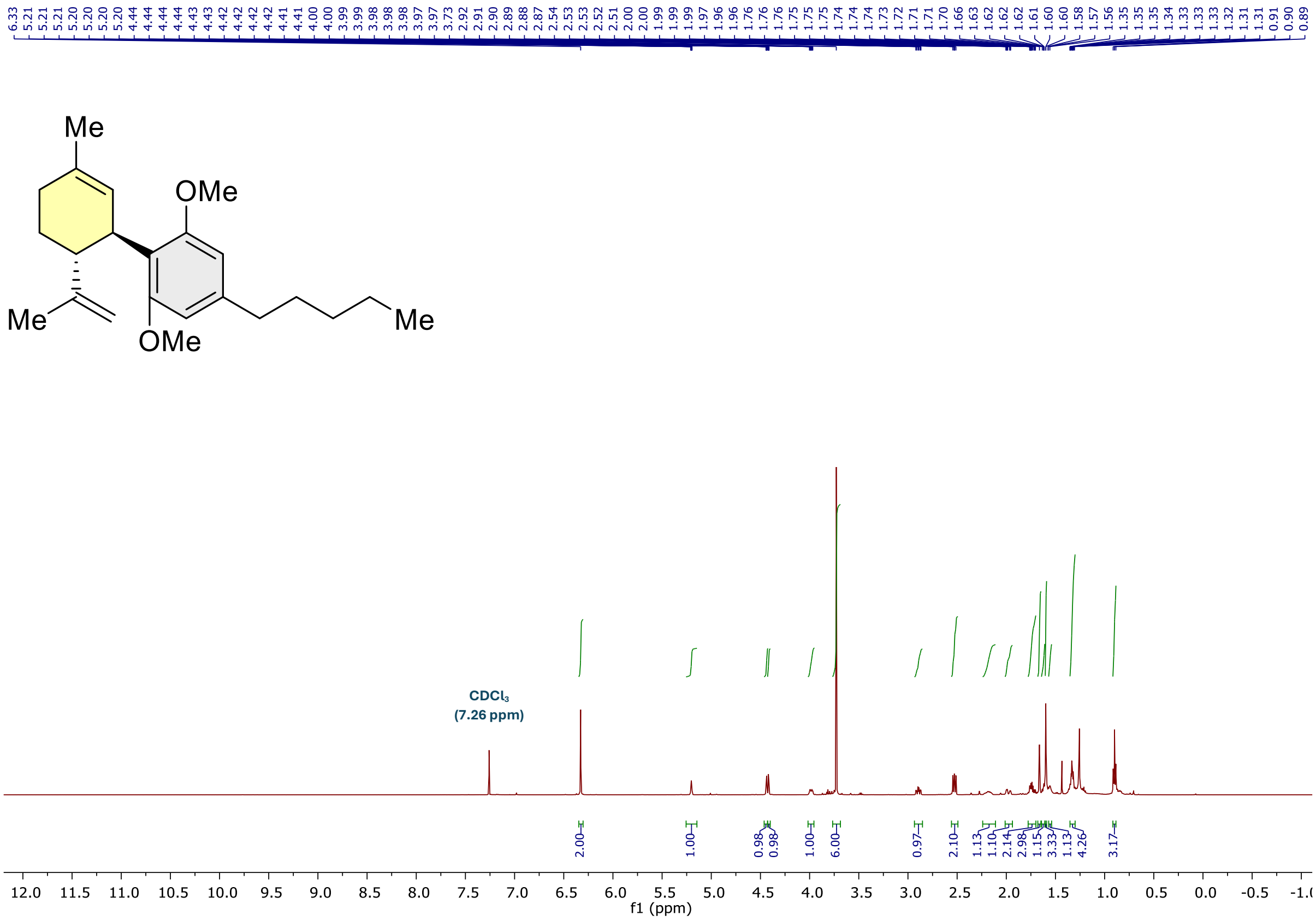

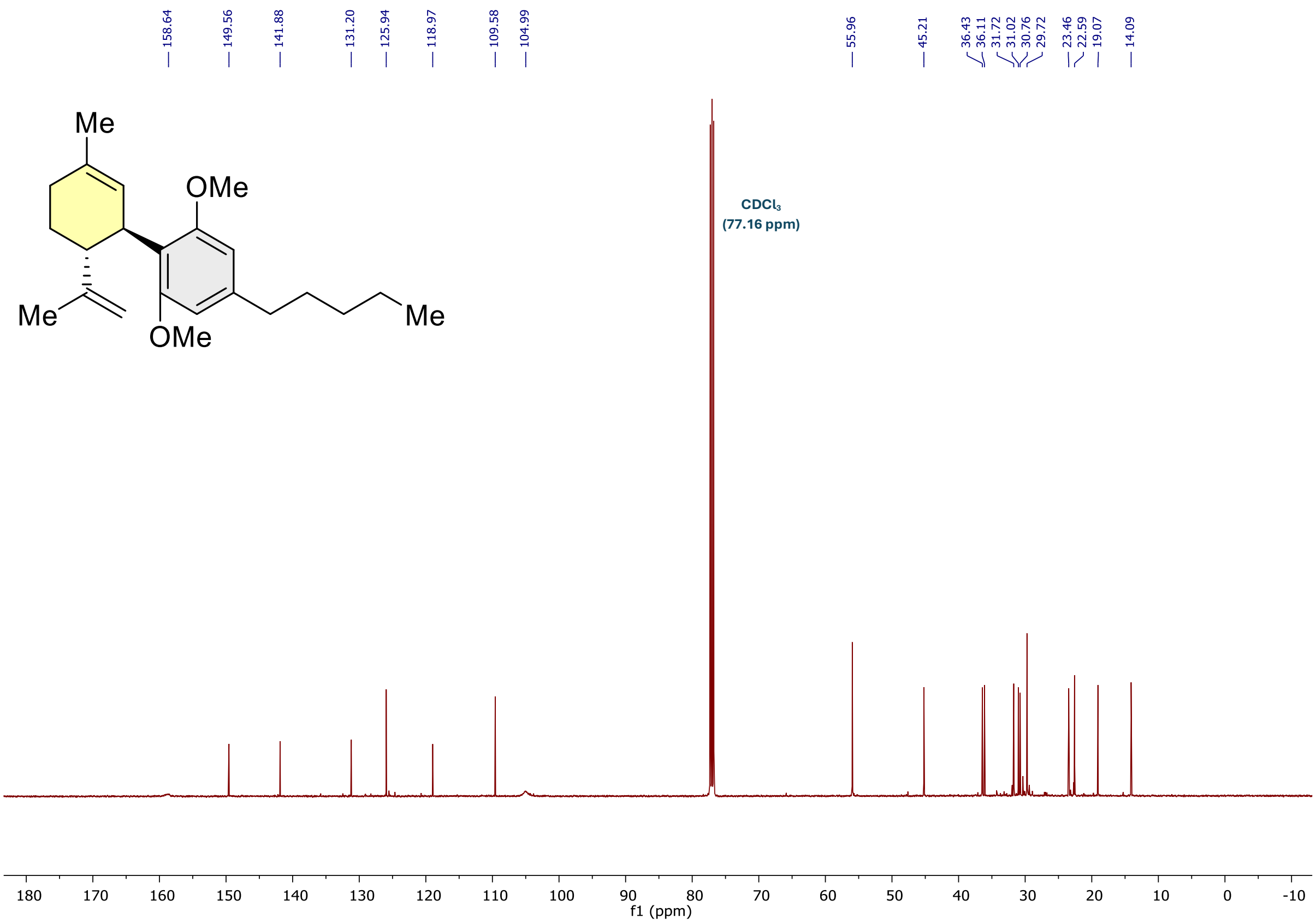

Compound S2. ^{1}HNMR (500 MHz, $CDCl_3$) and $^{13}C\{^1H\}$ NMR (125 MHz, $CDCl_3$).

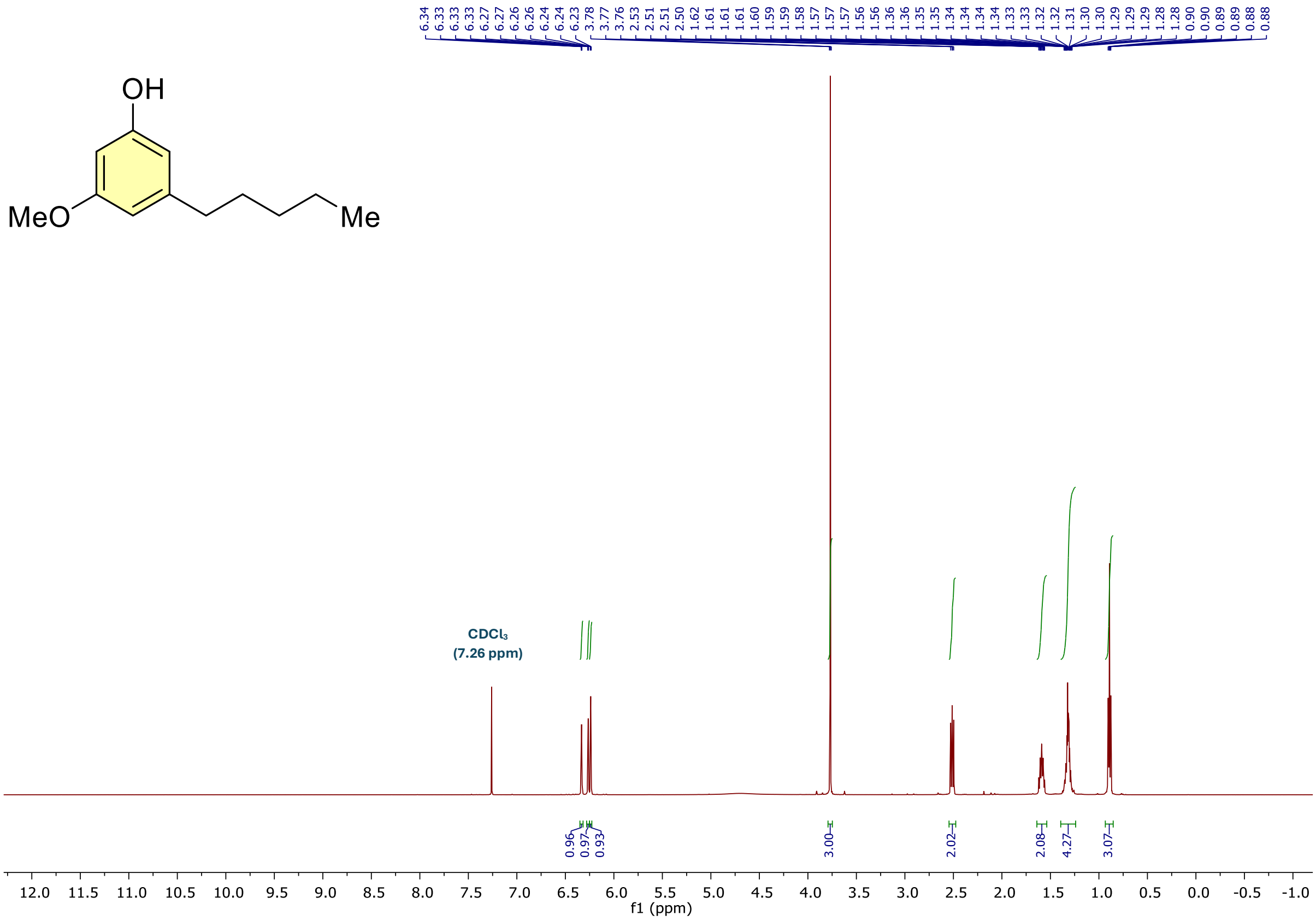

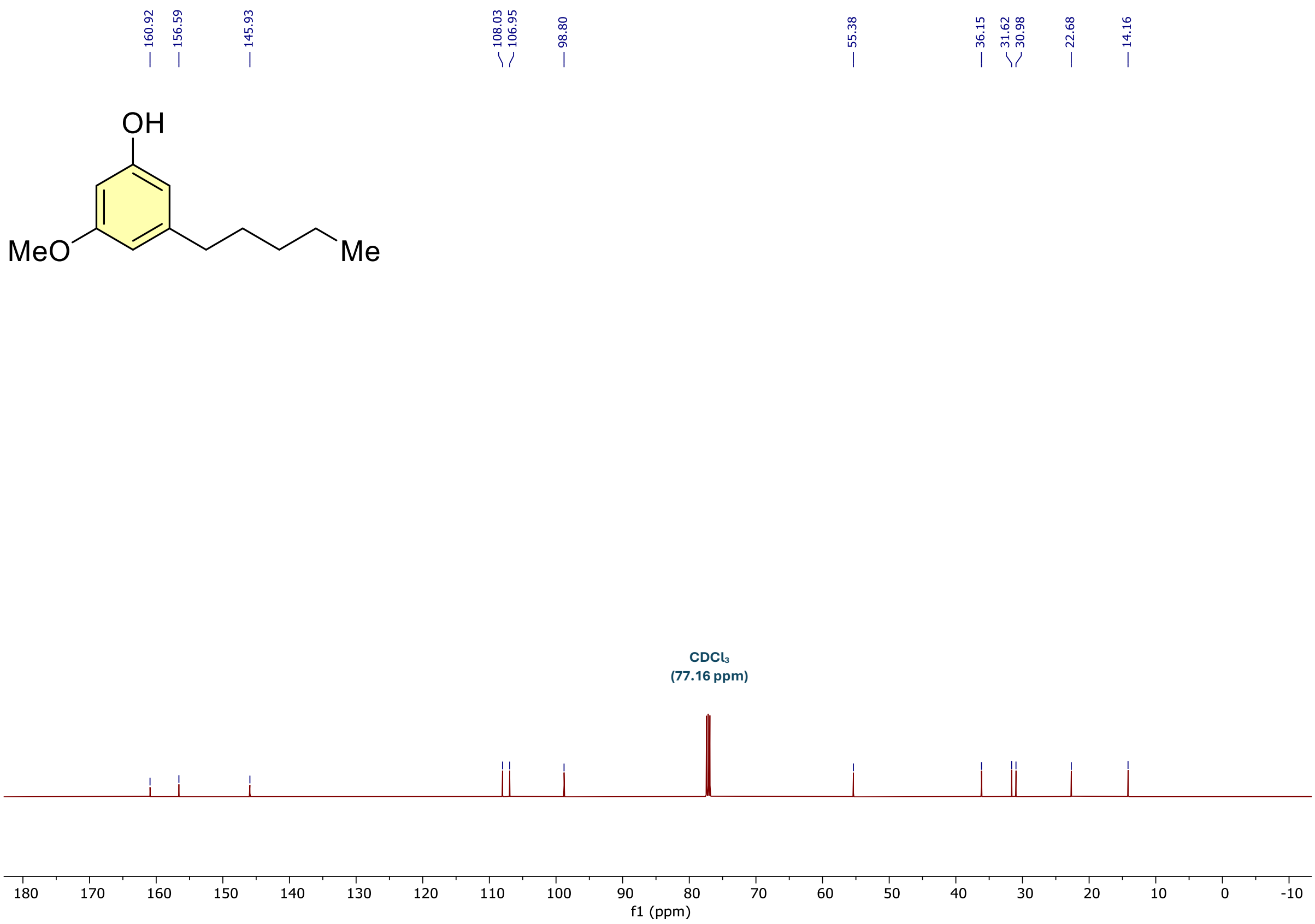

Compound 28. ^{1}HNMR (500 MHz, $CDCl_3$) and $^{13}C\{^{1}H\}$ NMR (125 MHz, $CDCl_3$).

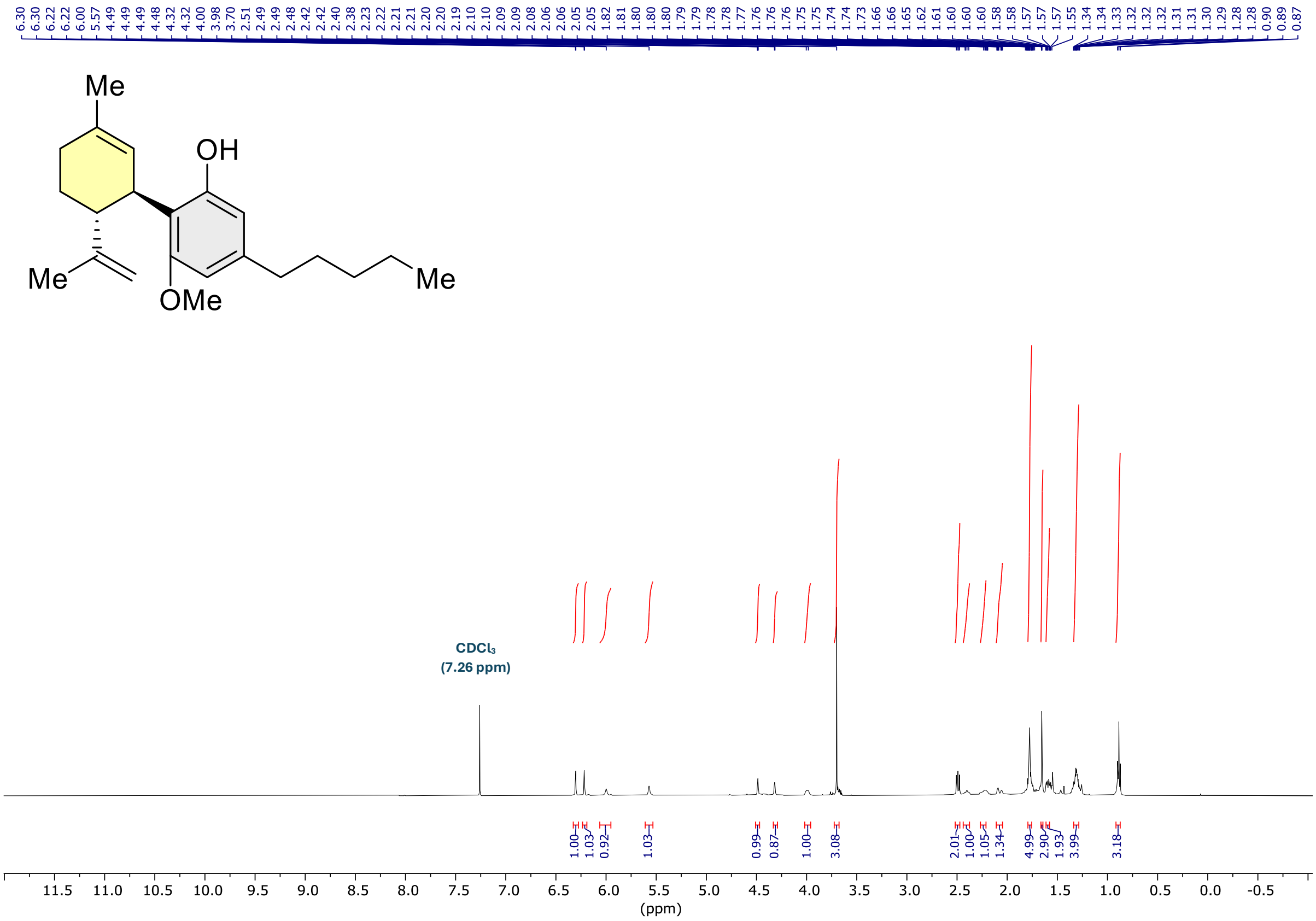

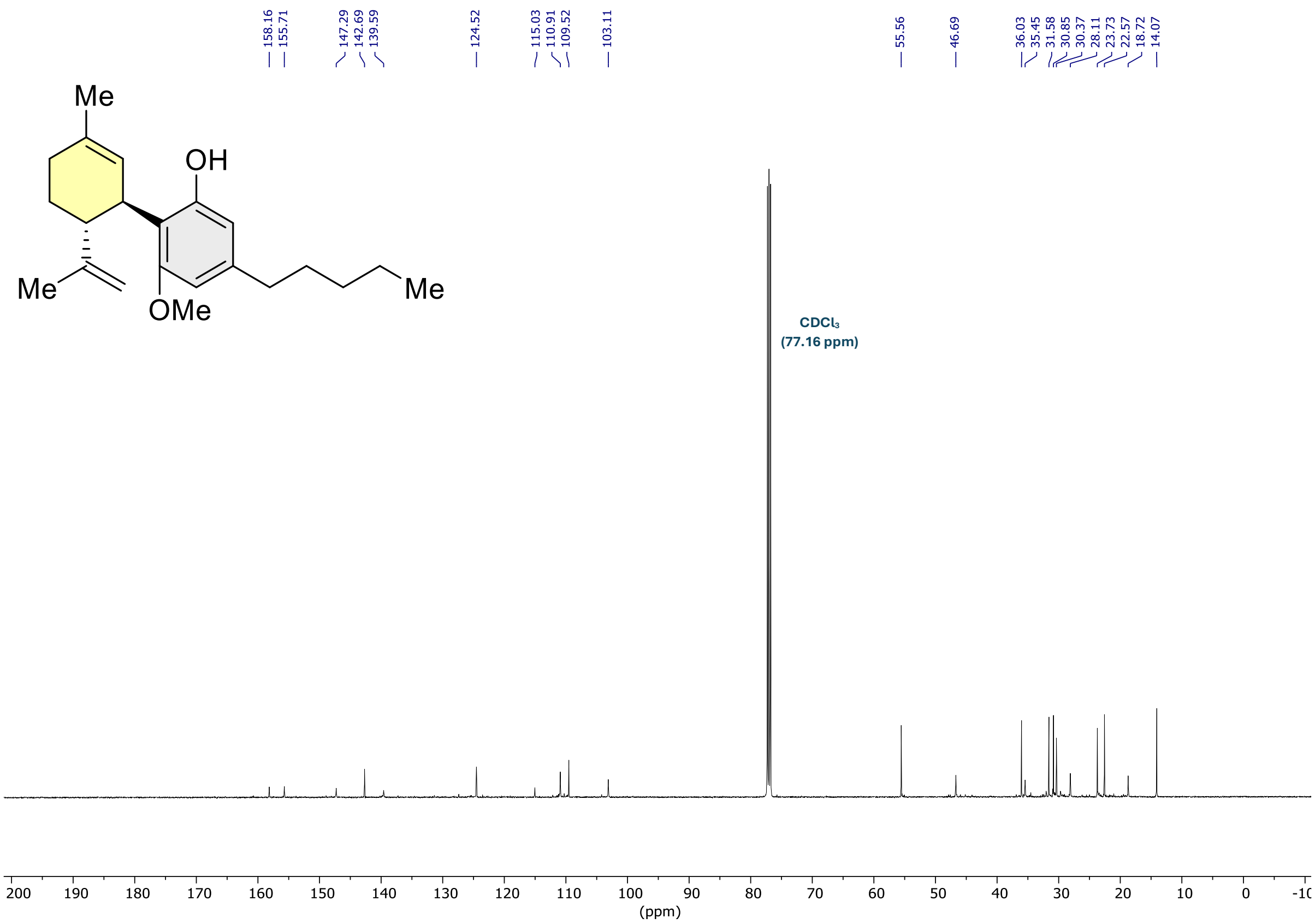

Compound 29. ^{1}HNMR (500 MHz, $CDCl_3$) $^{13}C\{^1H\}$ NMR (125 MHz, $CDCl_3$)

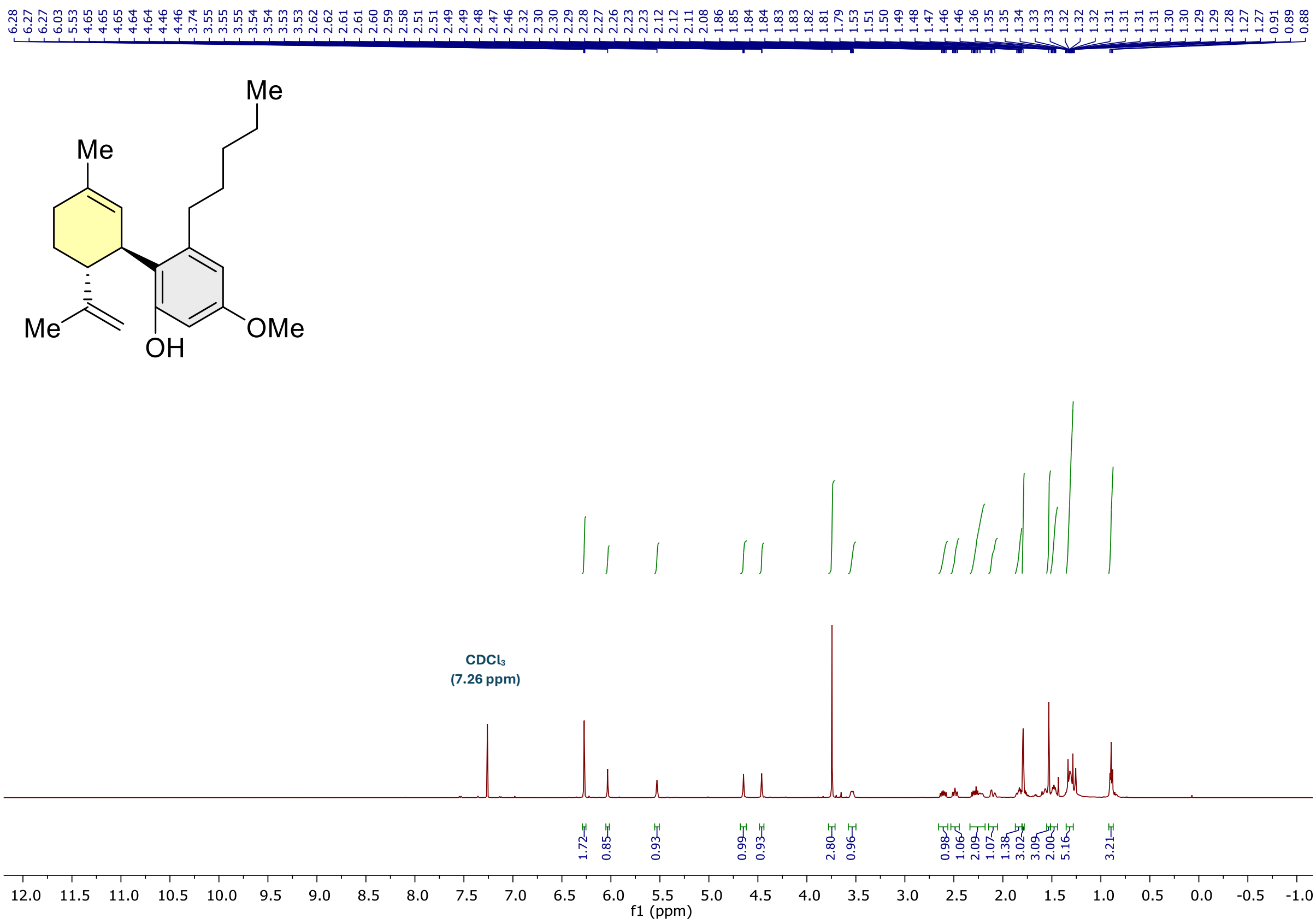

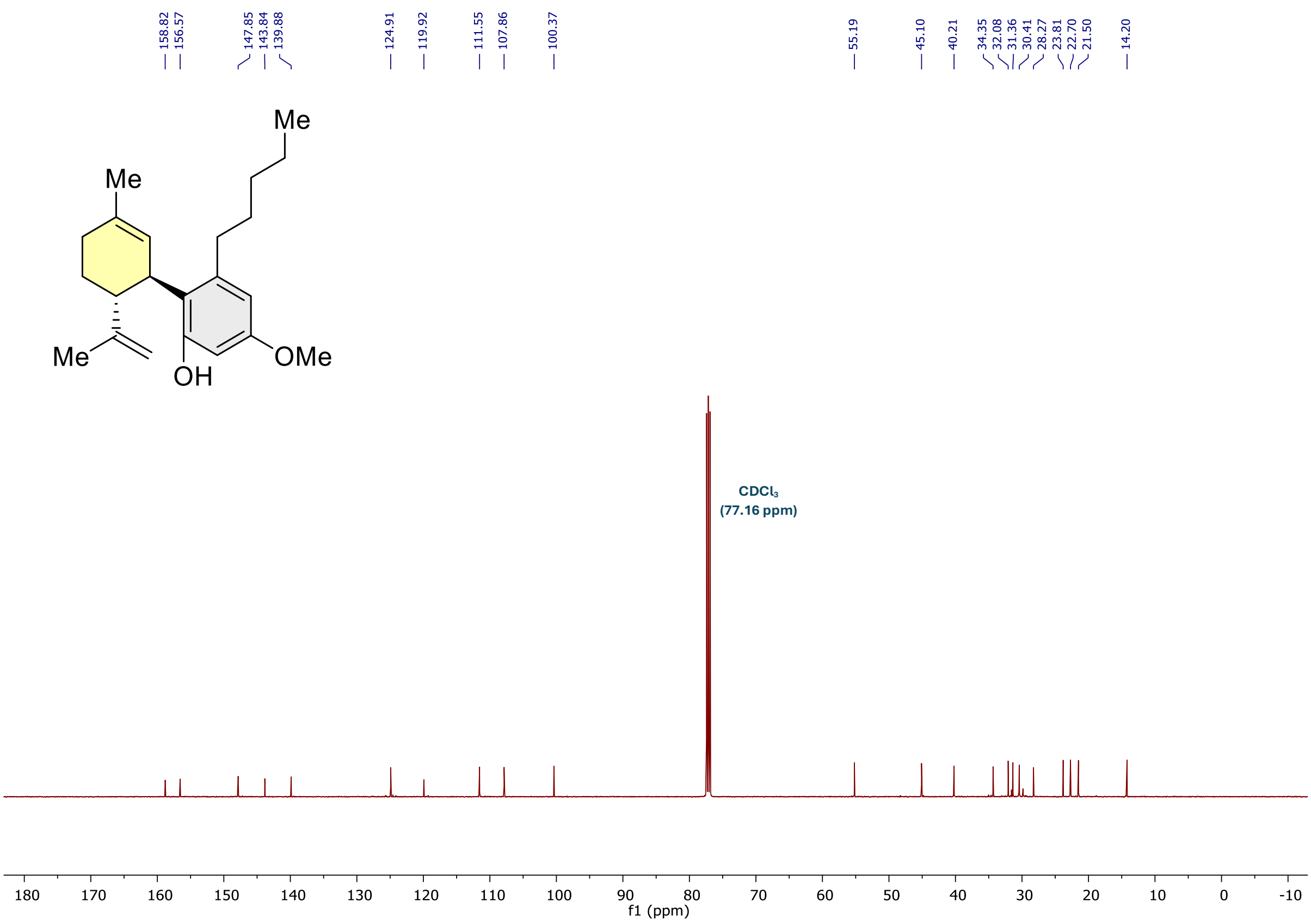

Compound 29. ^{1}H-^{13}C HSQC (500 MHz, $CDCl_3$) and ^{1}H-^{13}C HMBC (500 MHz, $CDCl_3$).

^{1}H-^{13}C HSQC (500 MHz, $CDCl_3$)

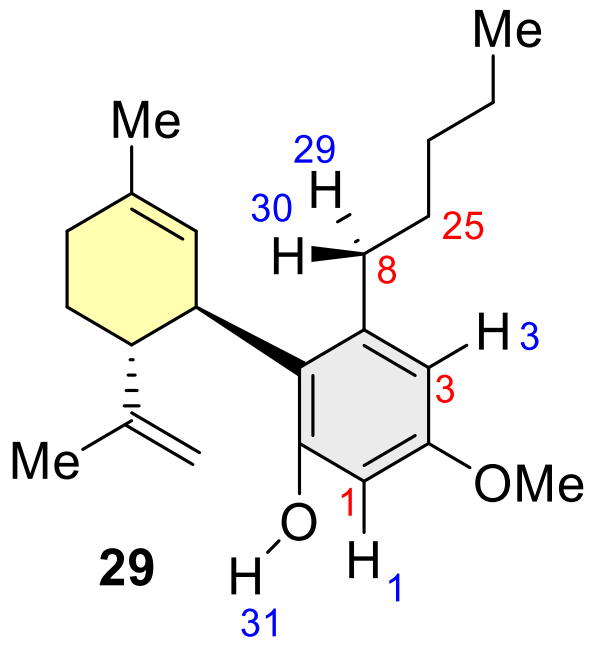

29

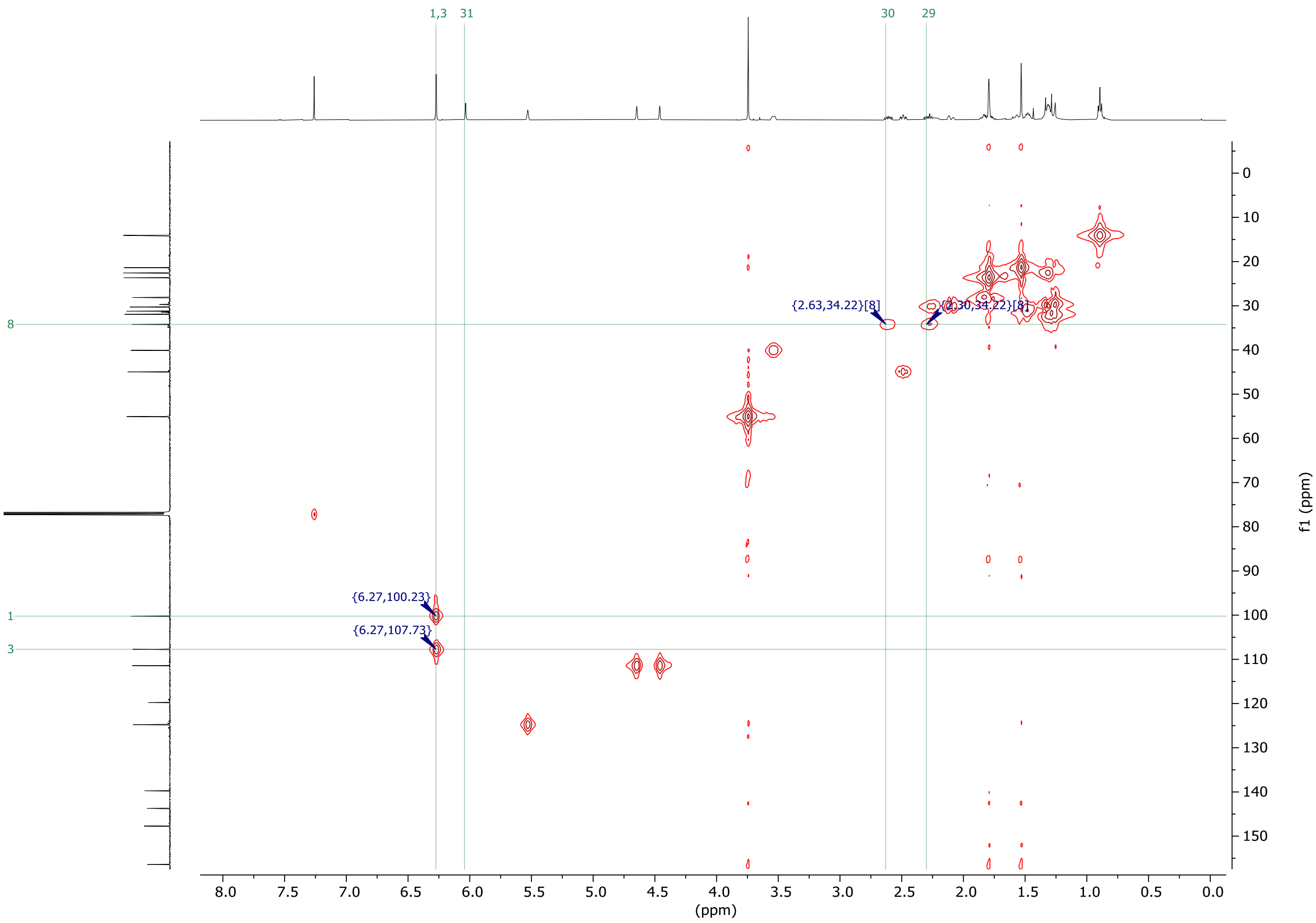

^1H-^{13}C HMBC (500 MHz, $CDCl_3$)

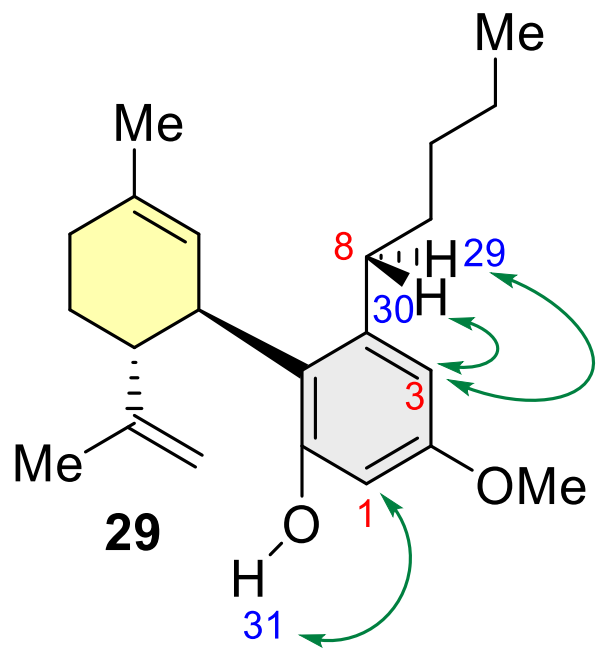

29

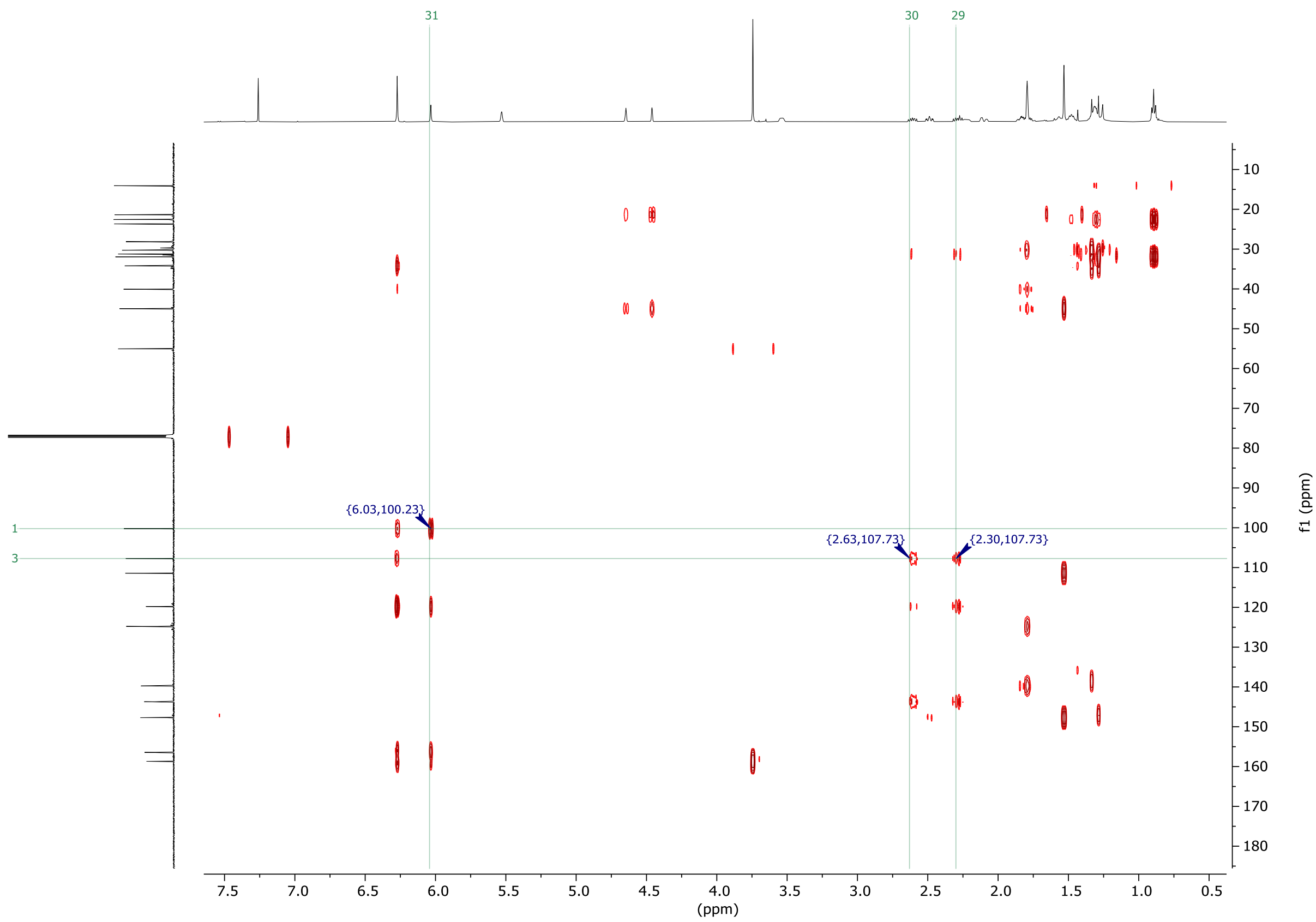

Compound 32. ^{1}HNMR (500 MHz, $CDCl_3$) and $^{13}C\{^1H\}$ NMR (125 MHz, $CDCl_3$).

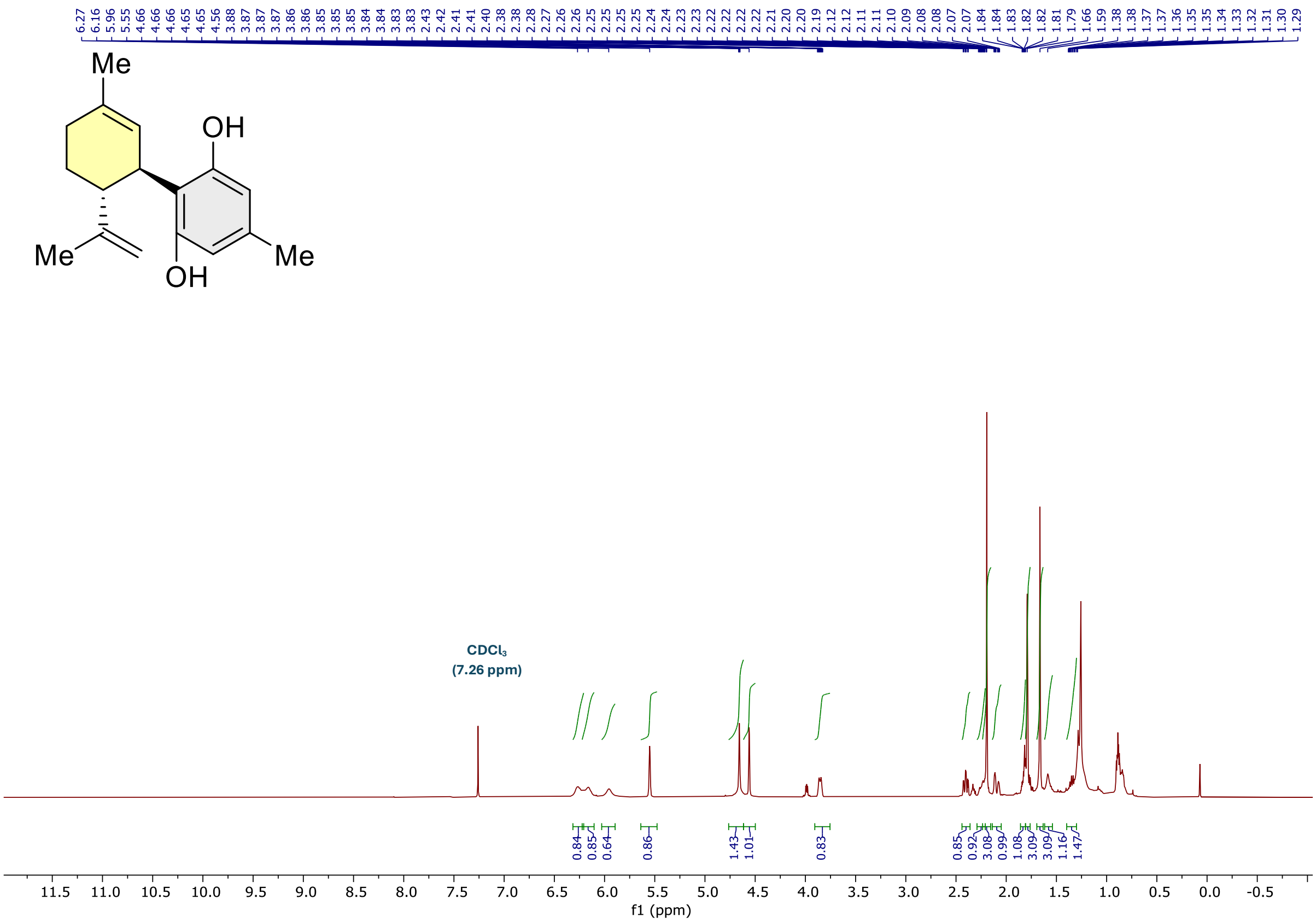

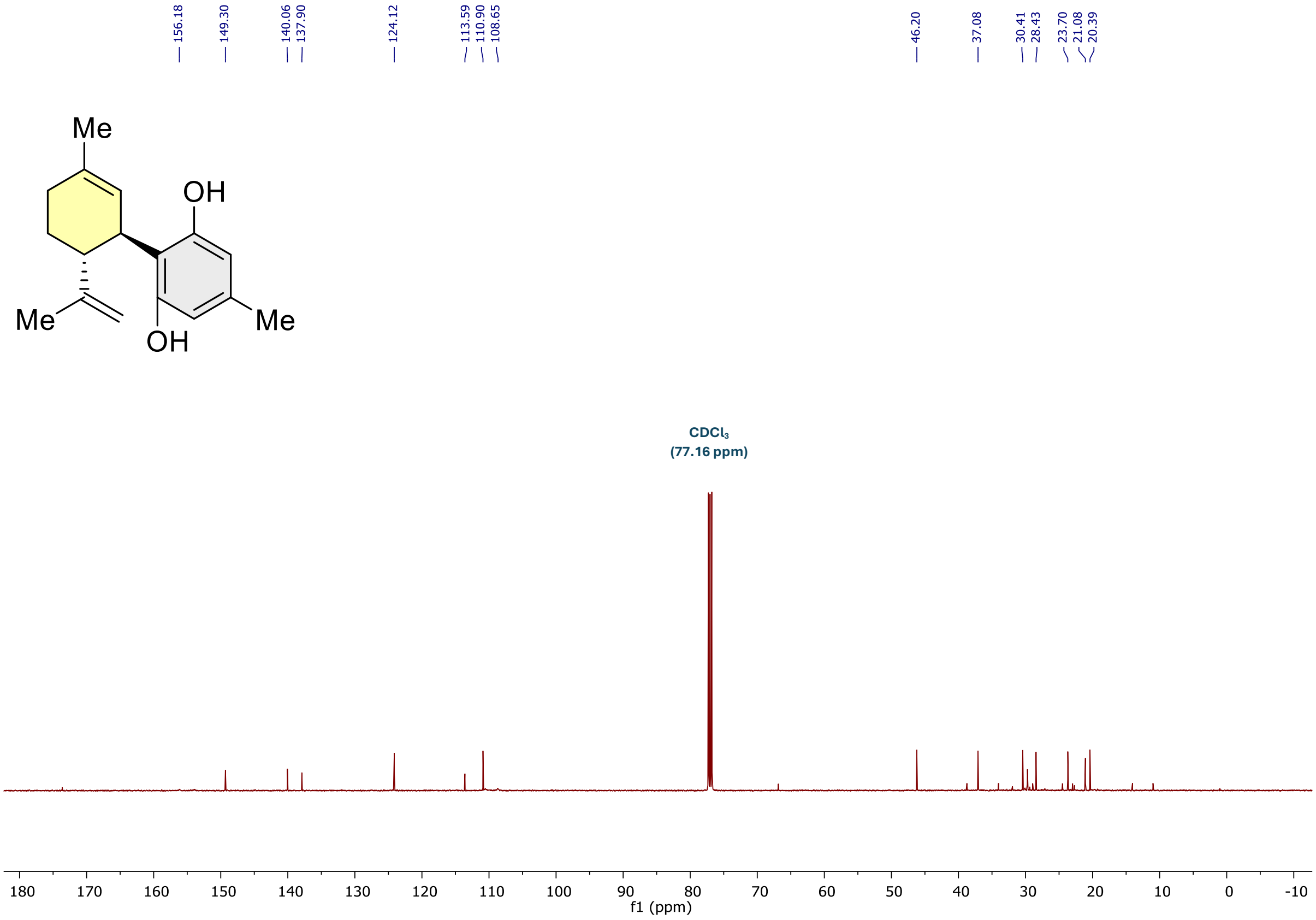

Compound 33. ^{1}HNMR (500 MHz, $CDCl_3$) and $^{13}C\{^1H\}$ NMR (100 MHz, $CDCl_3$).

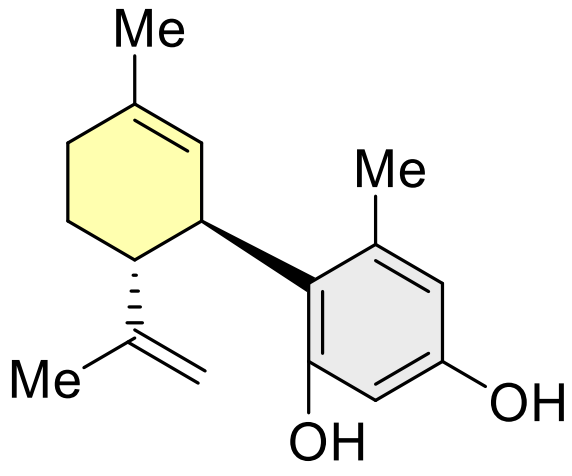

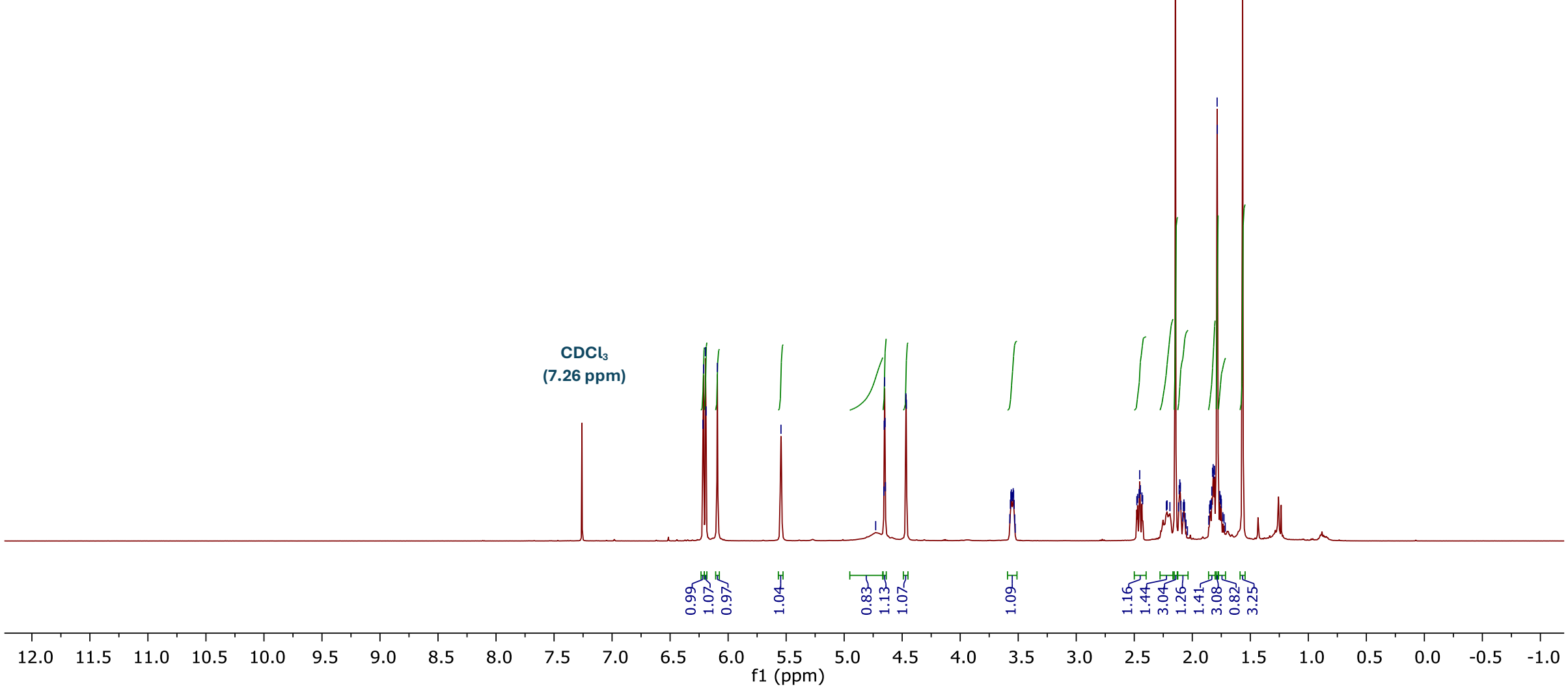

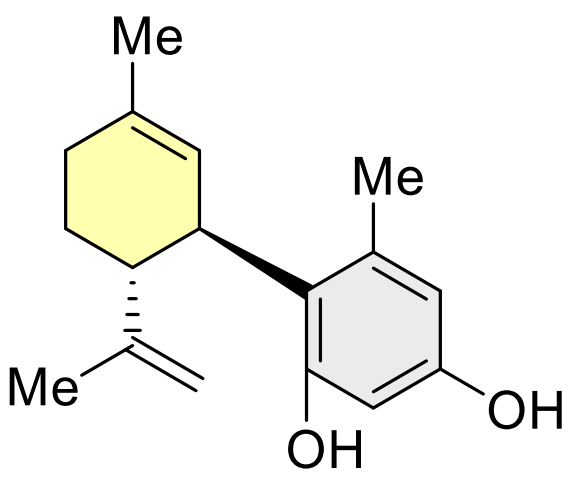

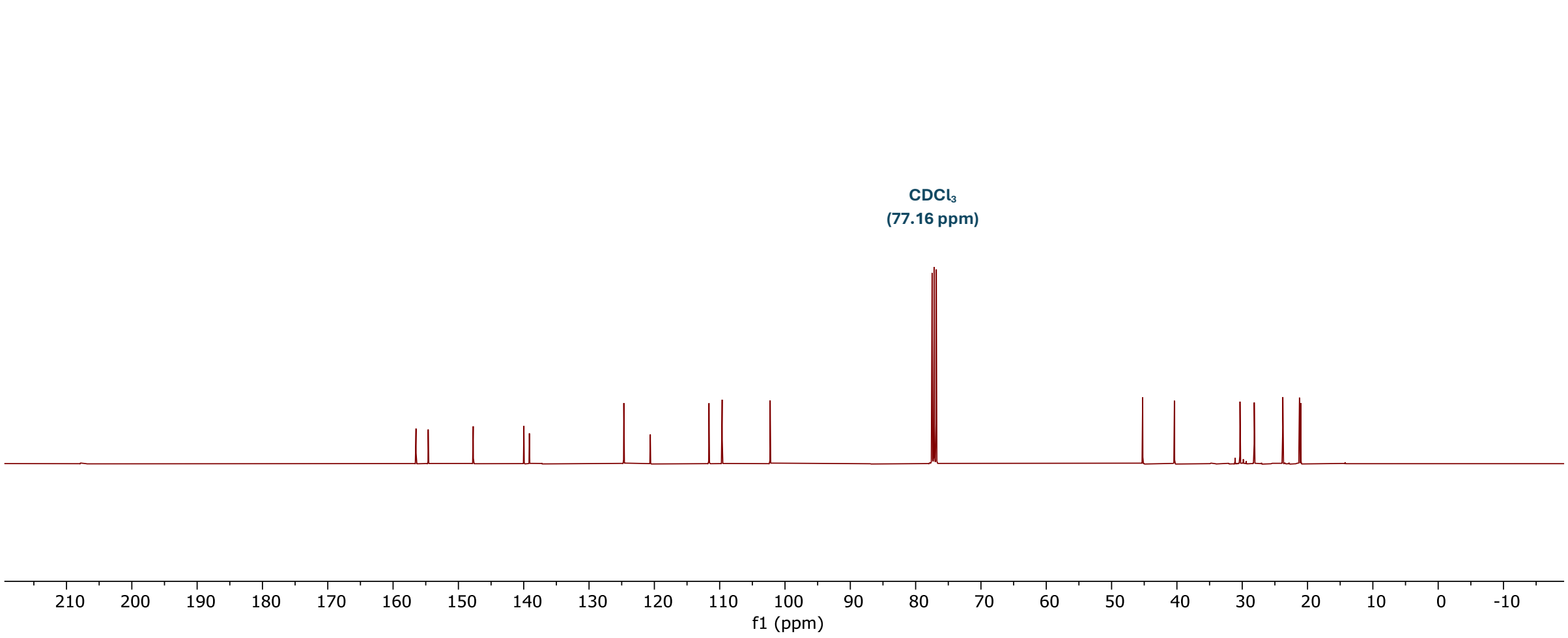

Compound 34. ^{1}HNMR (500 MHz, $CDCl_3$) and $^{13}C\{^{1}H\}$ NMR (125 MHz, $CDCl_3$).

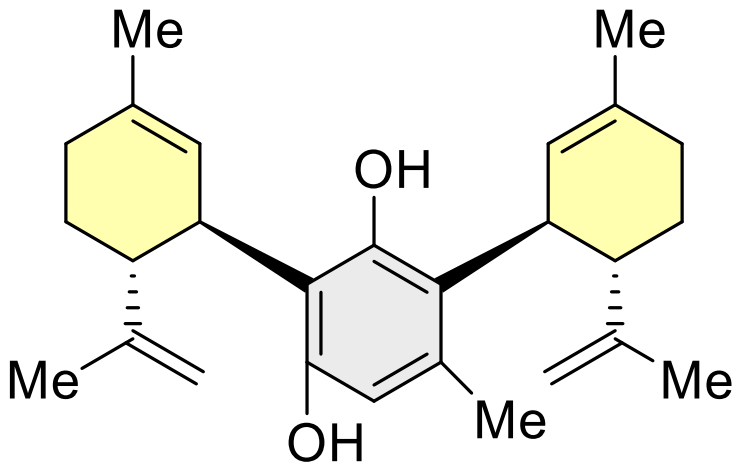

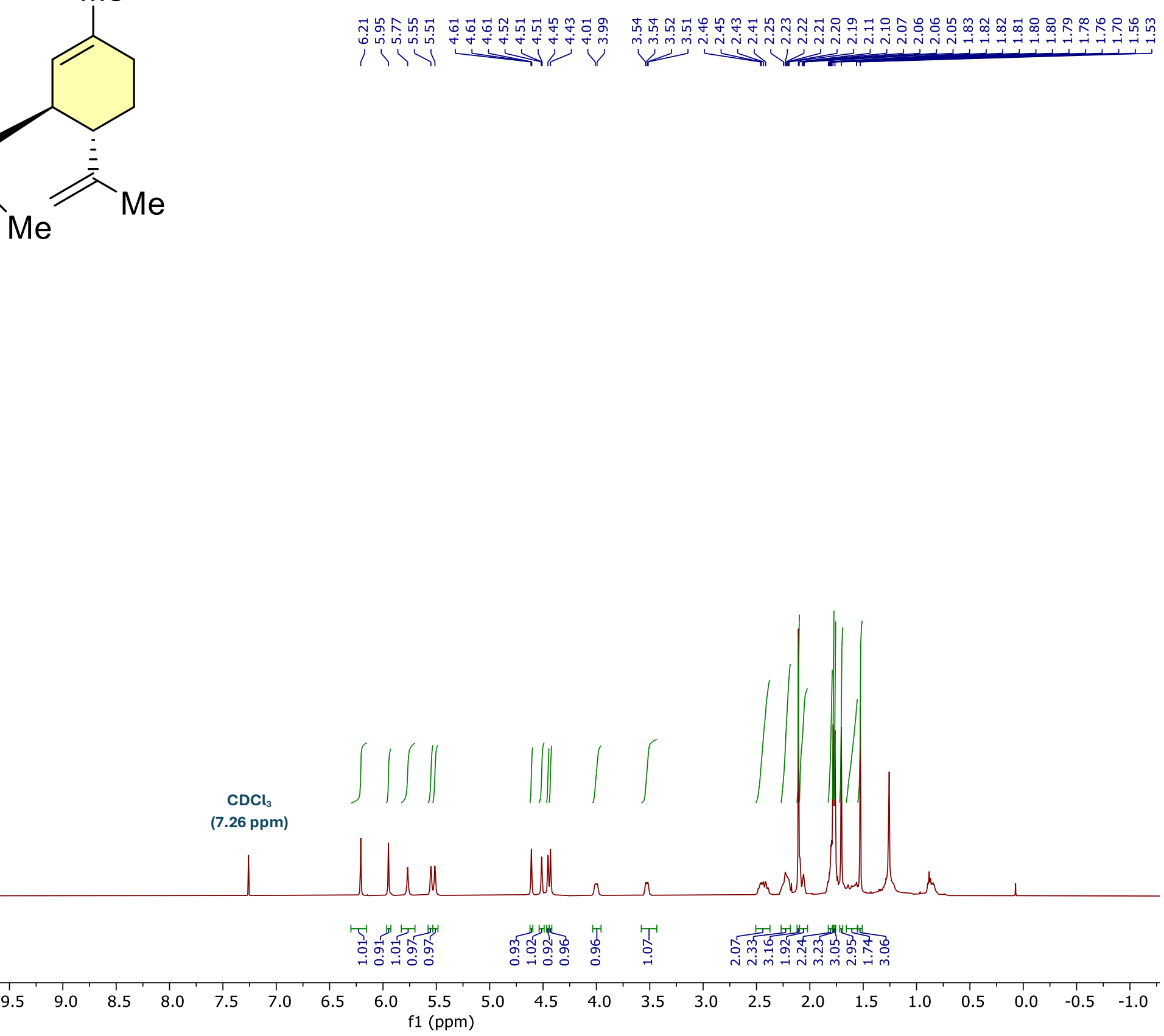

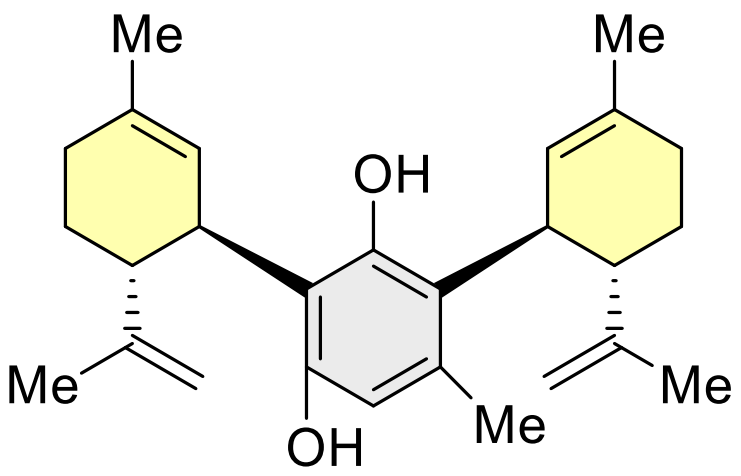

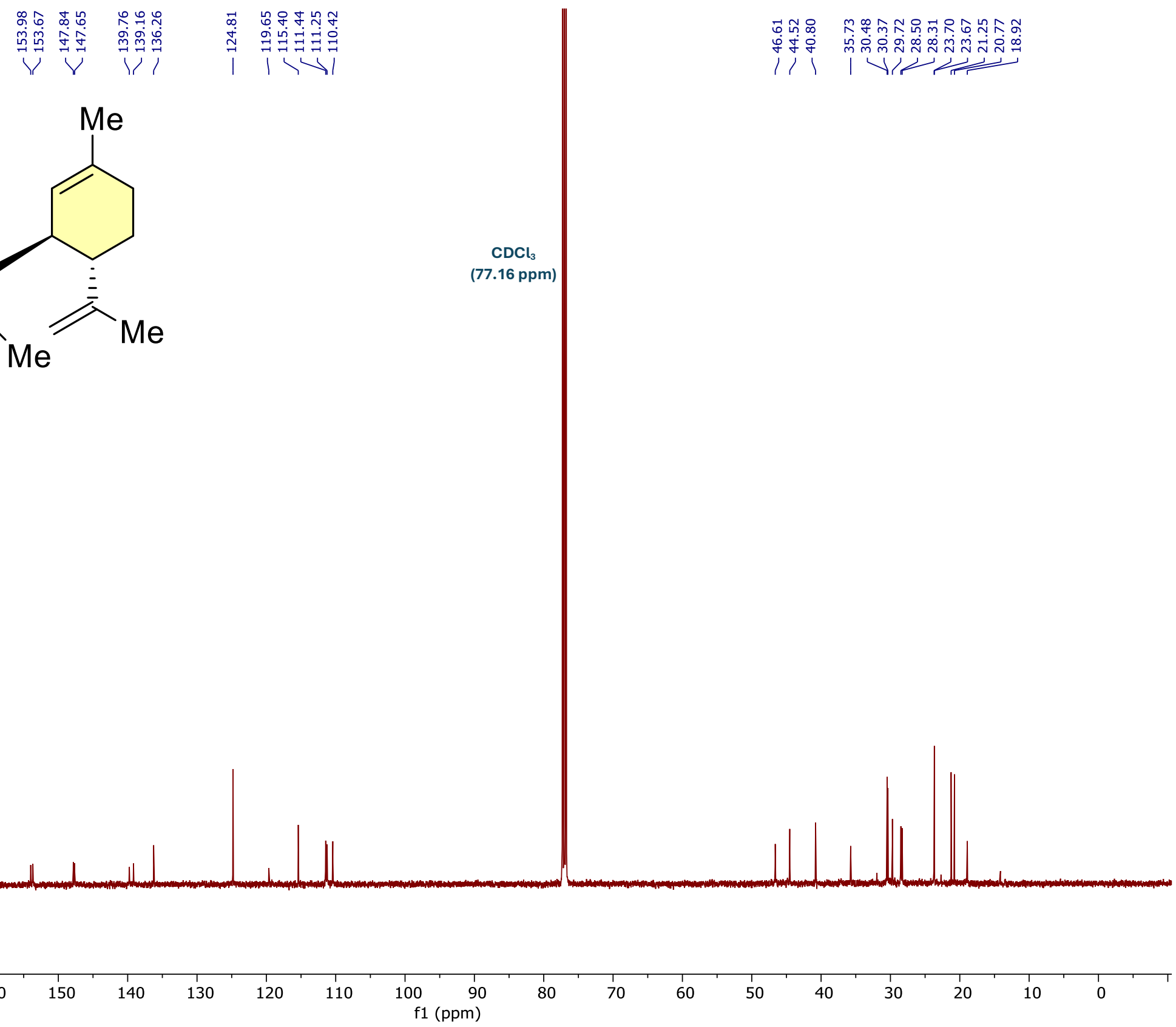

Compound 35. ^{1}HNMR (500 MHz, $CDCl_3$) and $^{13}C\{^{1}H\}$ NMR (125 MHz, $CDCl_3$).

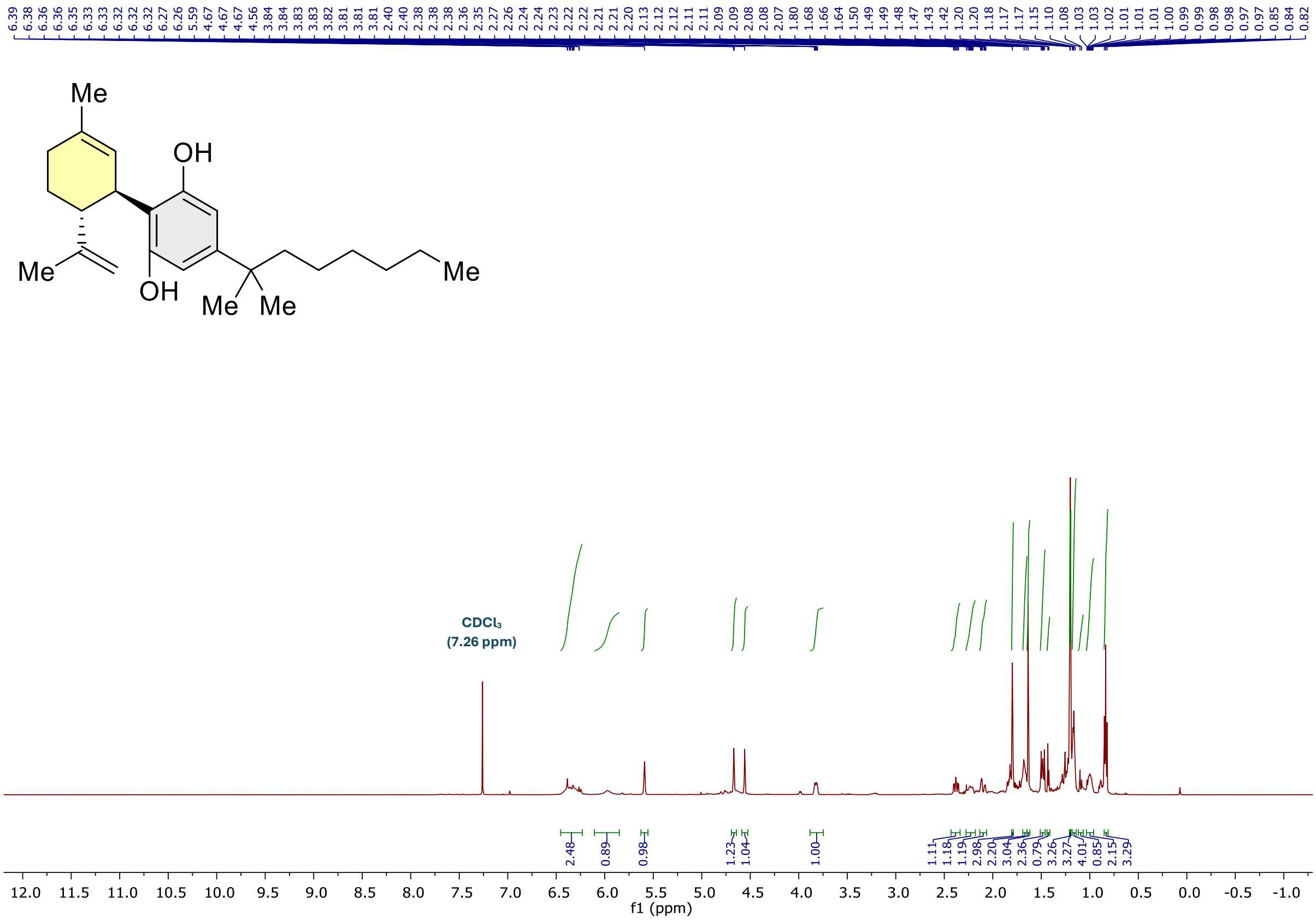

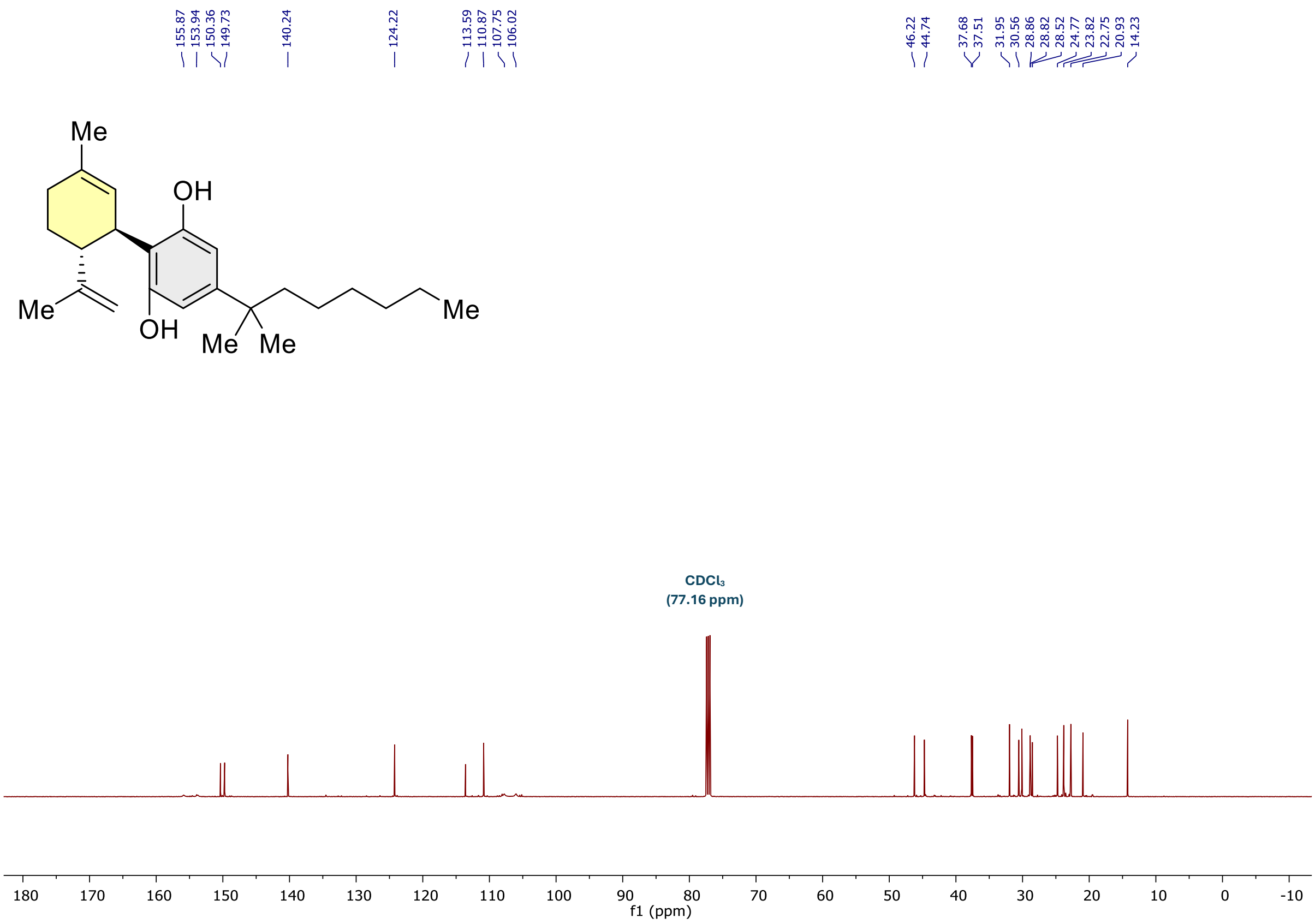

Compound 36. ^{1}HNMR (500 MHz, $CDCl_3$) and $^{13}C\{^{1}H\}$ NMR (125 MHz, $CDCl_3$).

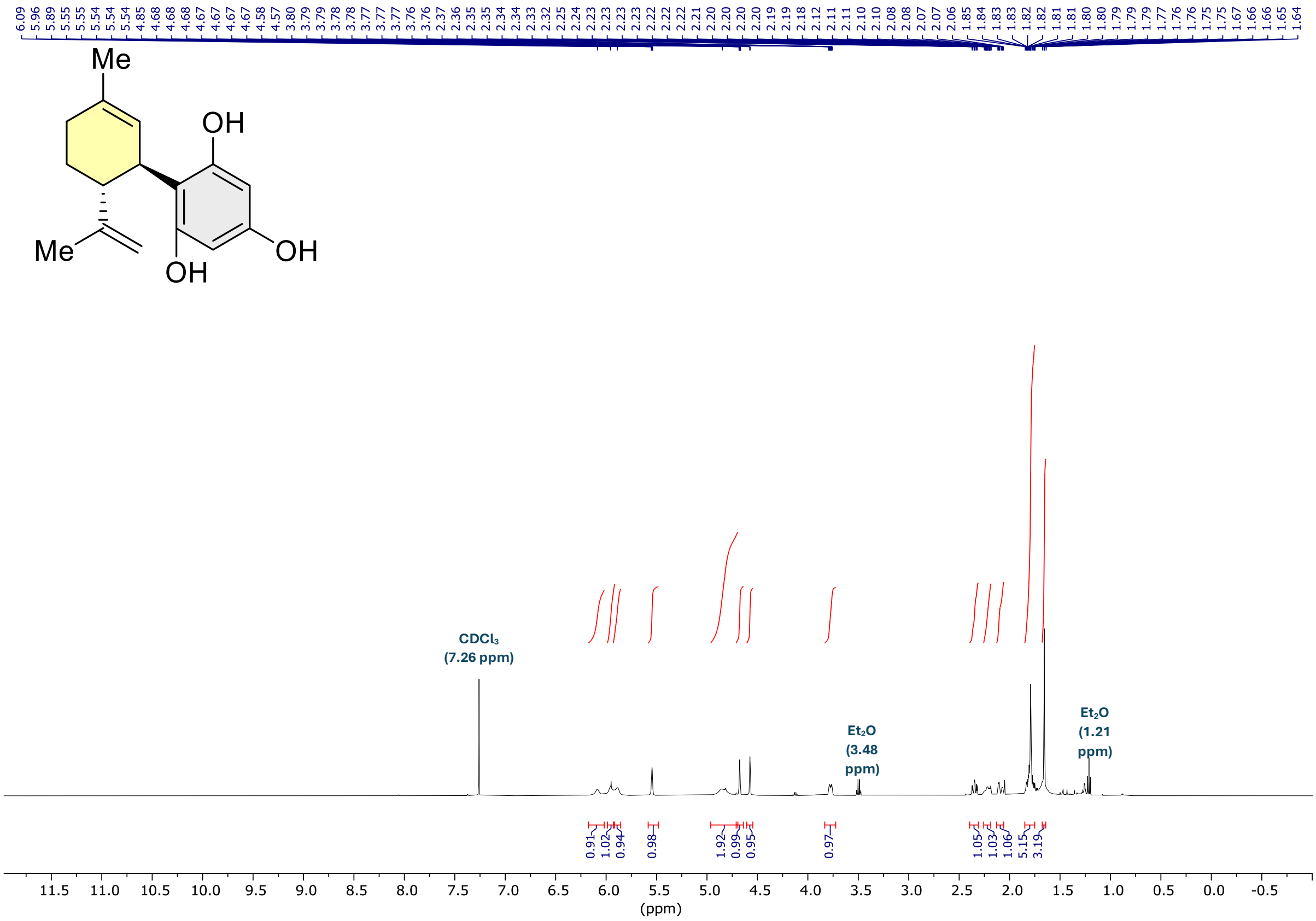

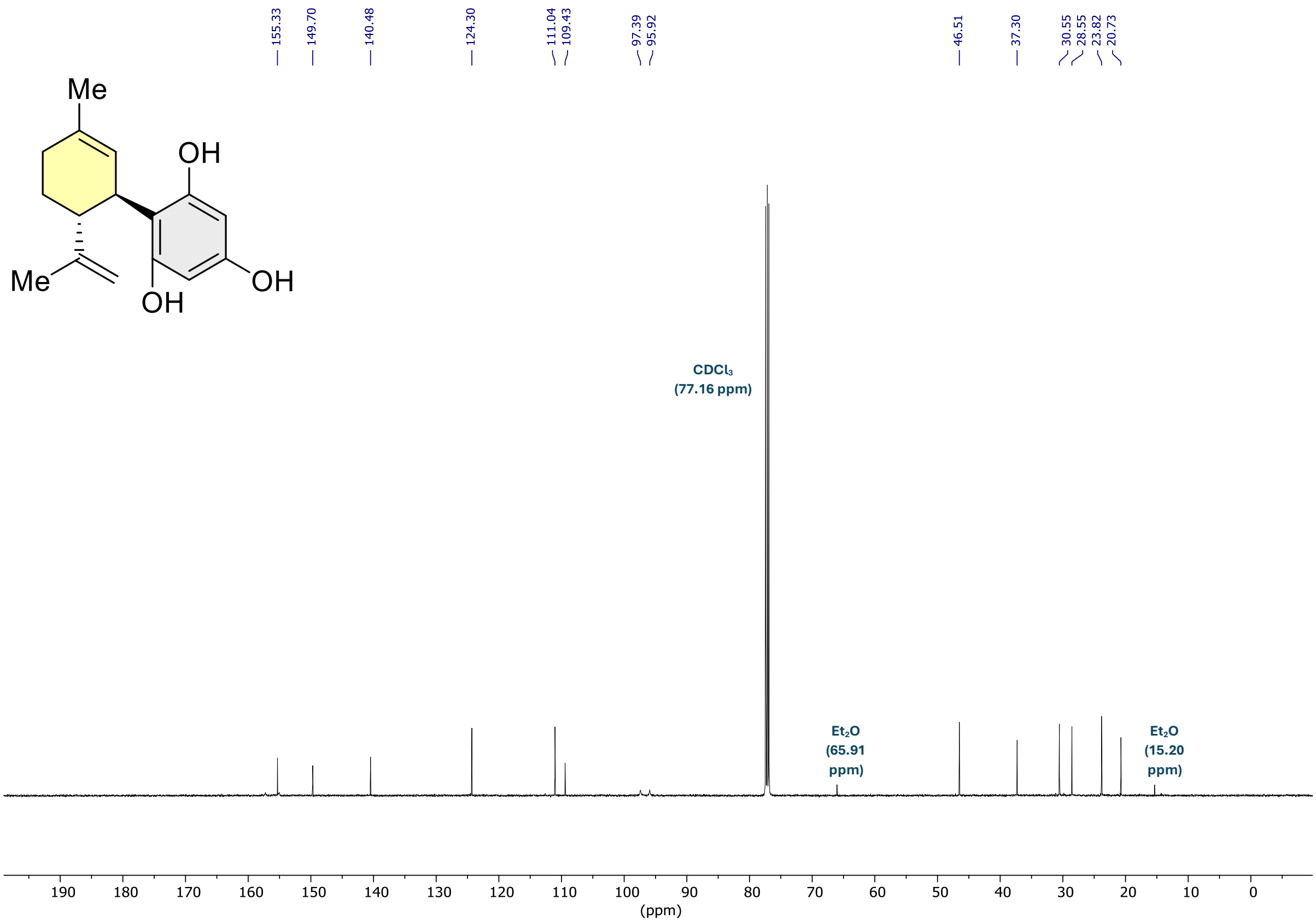

Compound 37. ^{1}HNMR (500 MHz, $CDCl_3$) and $^{13}C\{^1H\}$ NMR (125 MHz, $CDCl_3$).

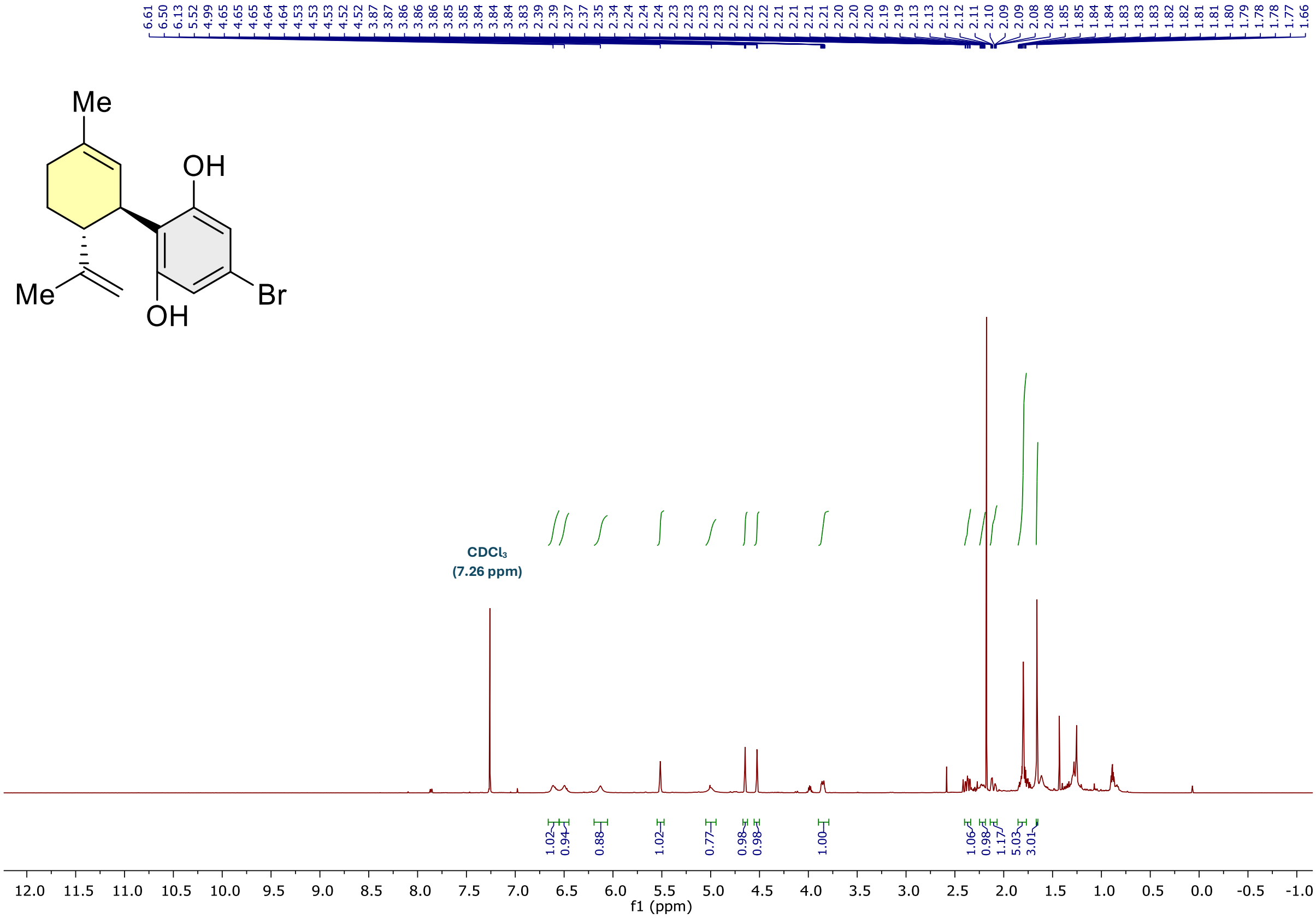

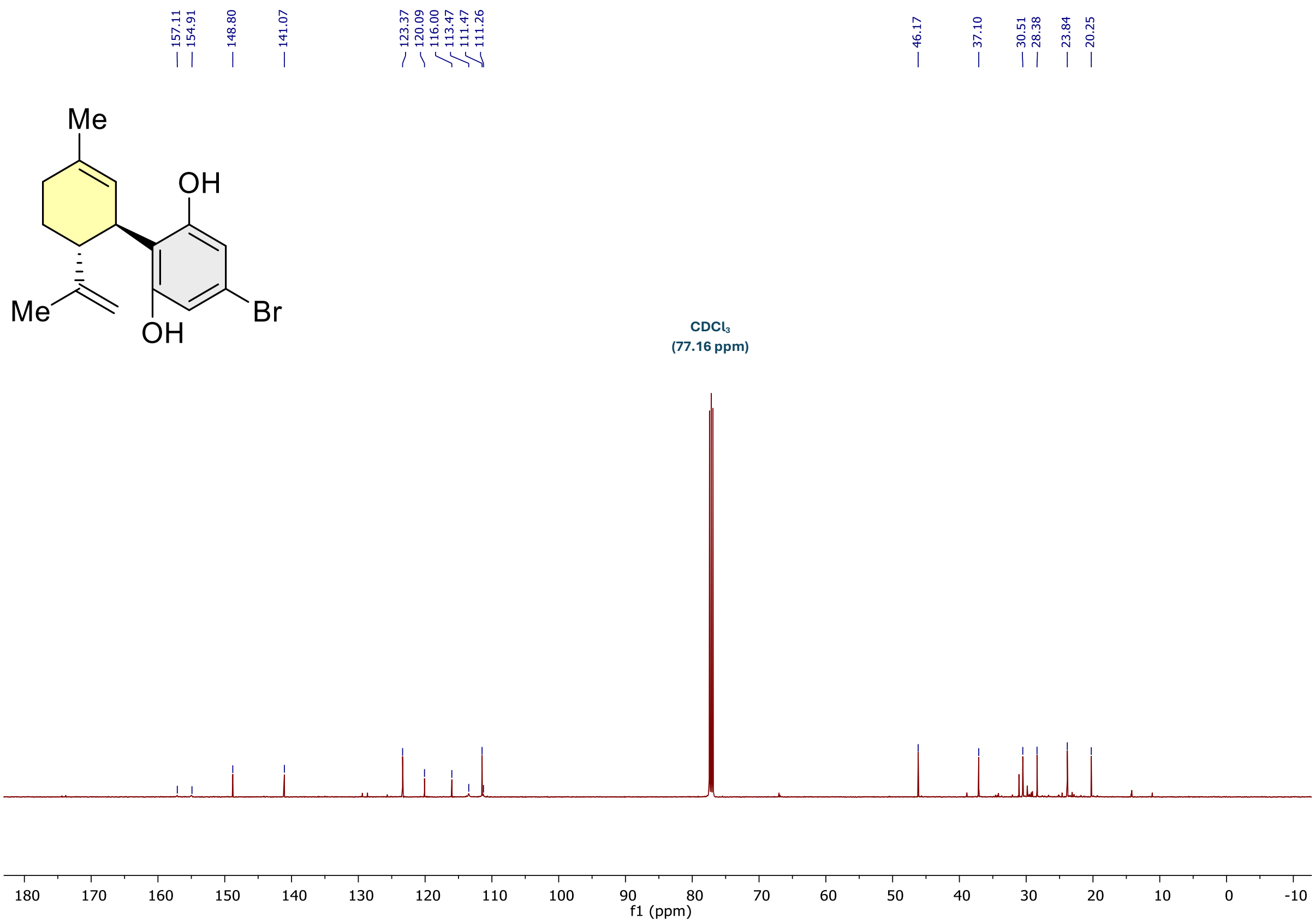

Compound 38. ^{1}HNMR (500 MHz, $CDCl_3$) and $^{13}C\{^{1}H\}$ NMR (125 MHz, $CDCl_3$).

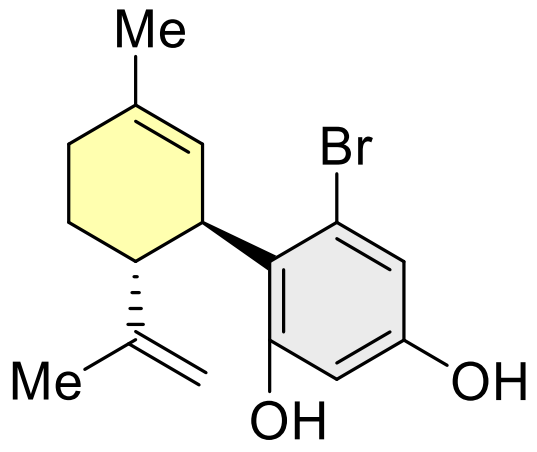

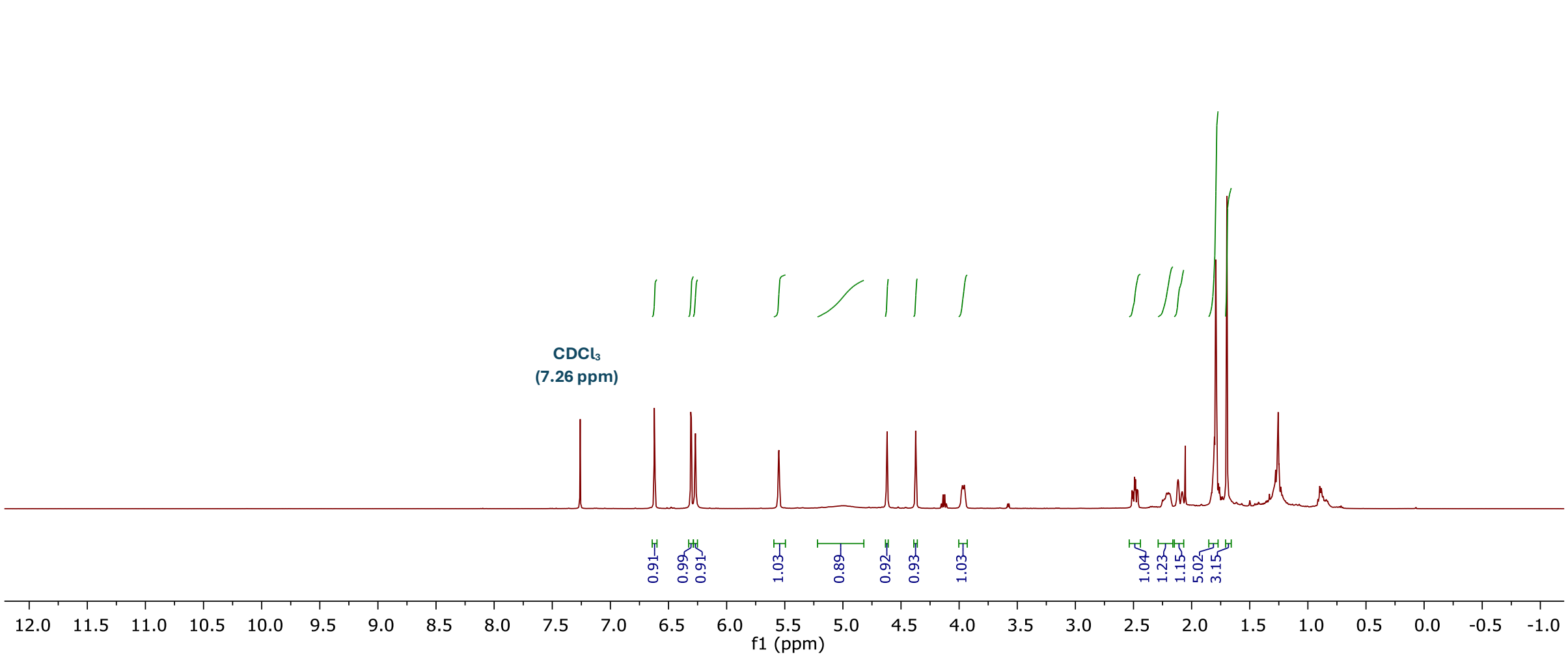

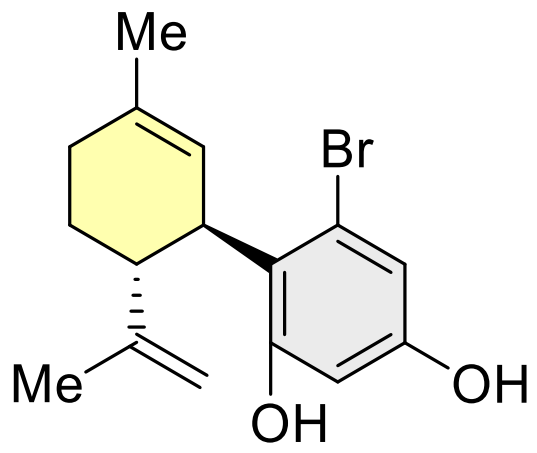

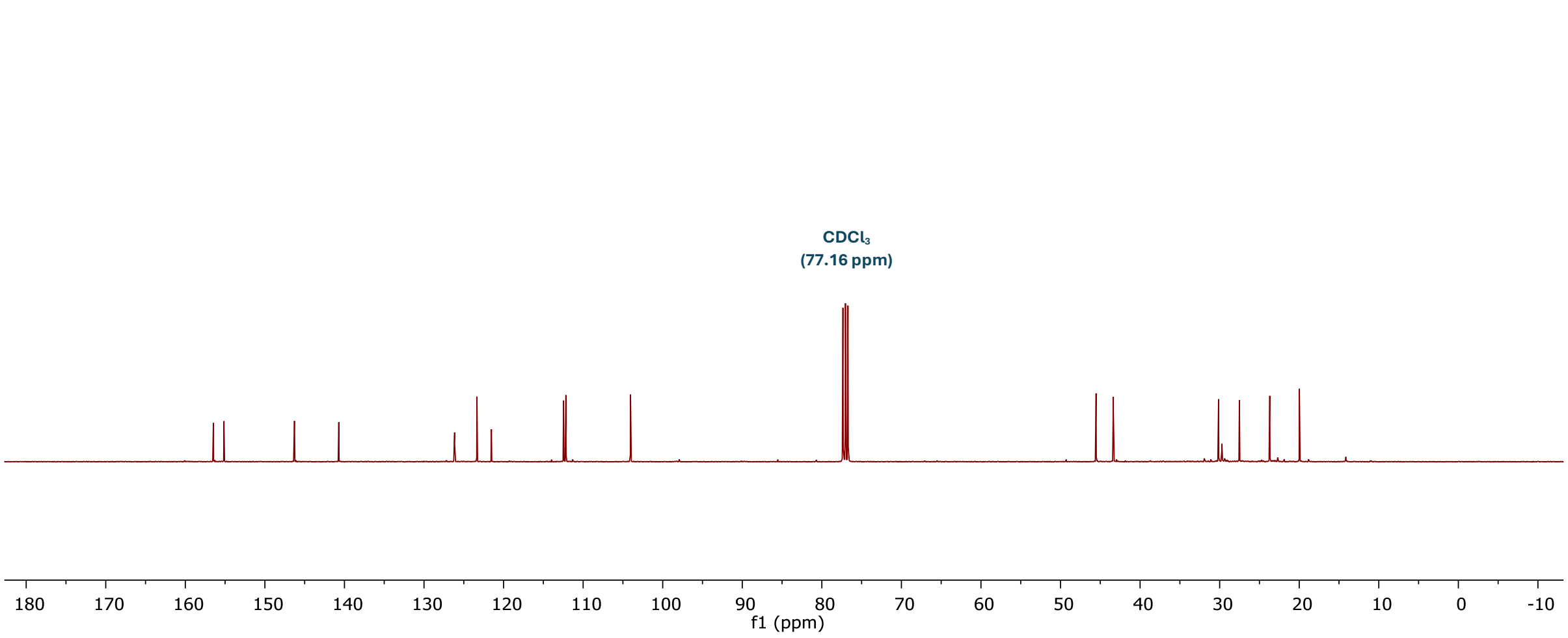

Compound 39. ^{1}HNMR (500 MHz, $CDCl_3$) and $^{13}C\{^1H\}$ NMR (125 MHz, $CDCl_3$).

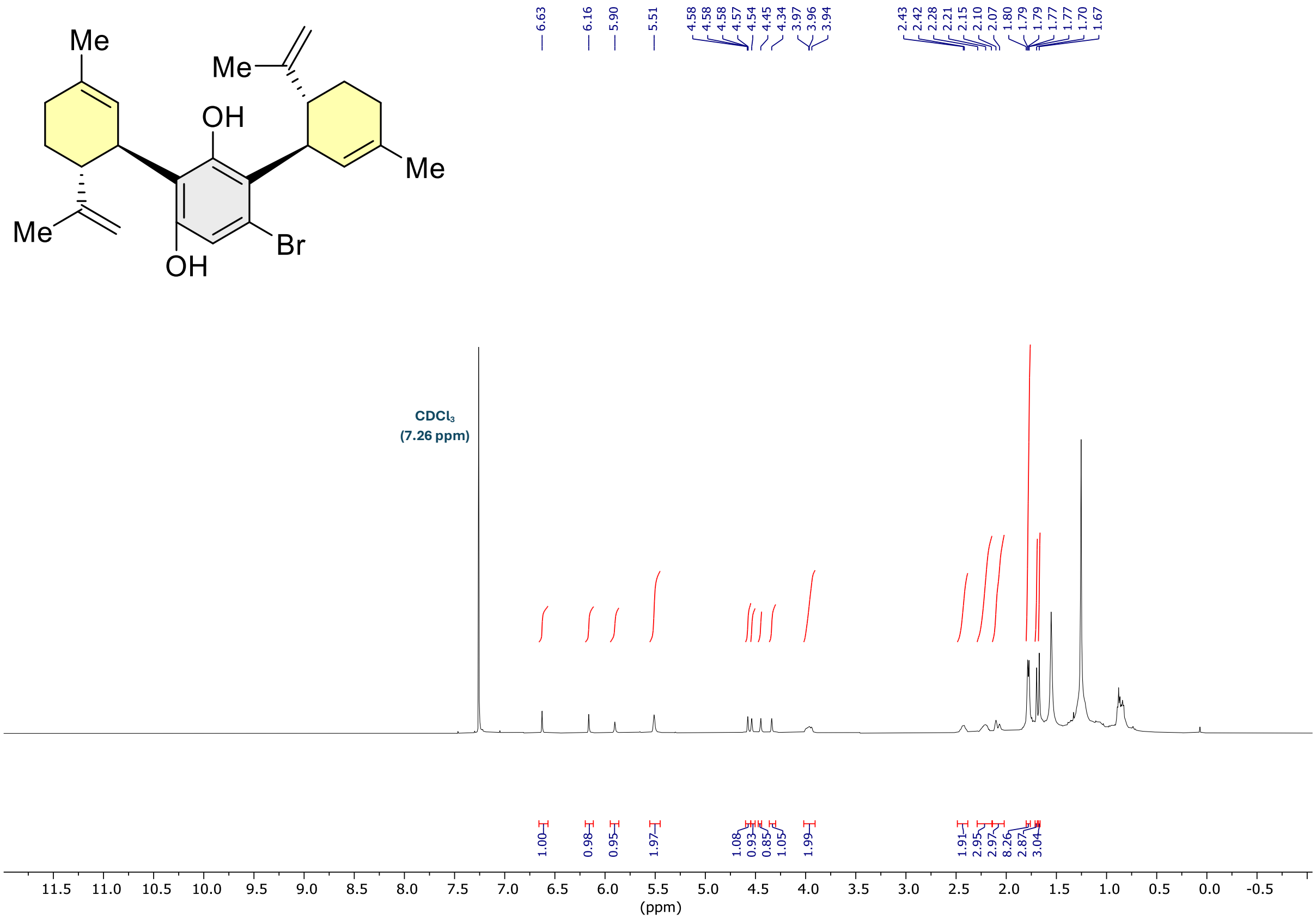

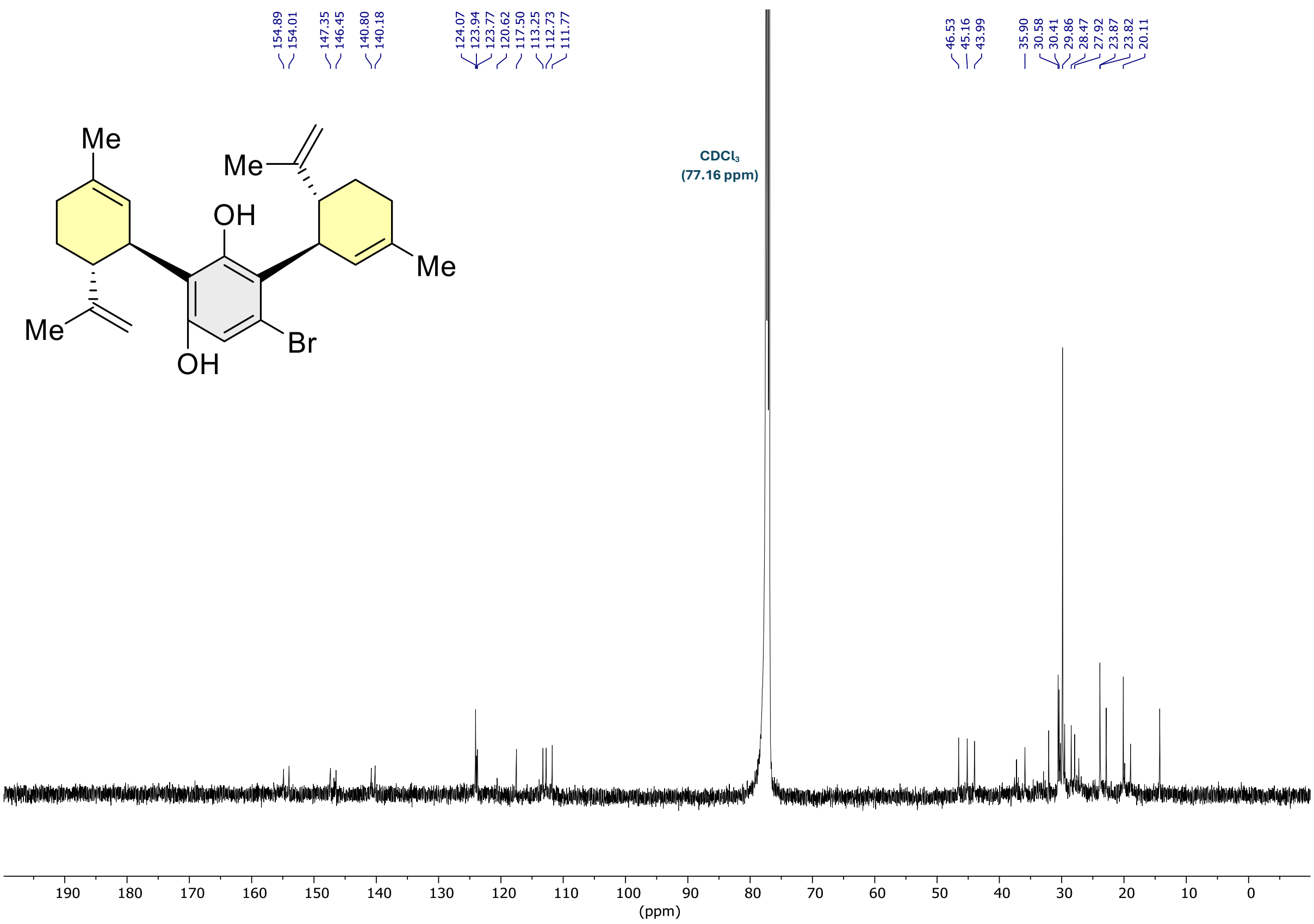

Compound 41. ^{1}HNMR (500 MHz, $CDCl_3$) and $^{13}C\{^{1}H\}$ NMR (125 MHz, $CDCl_3$).

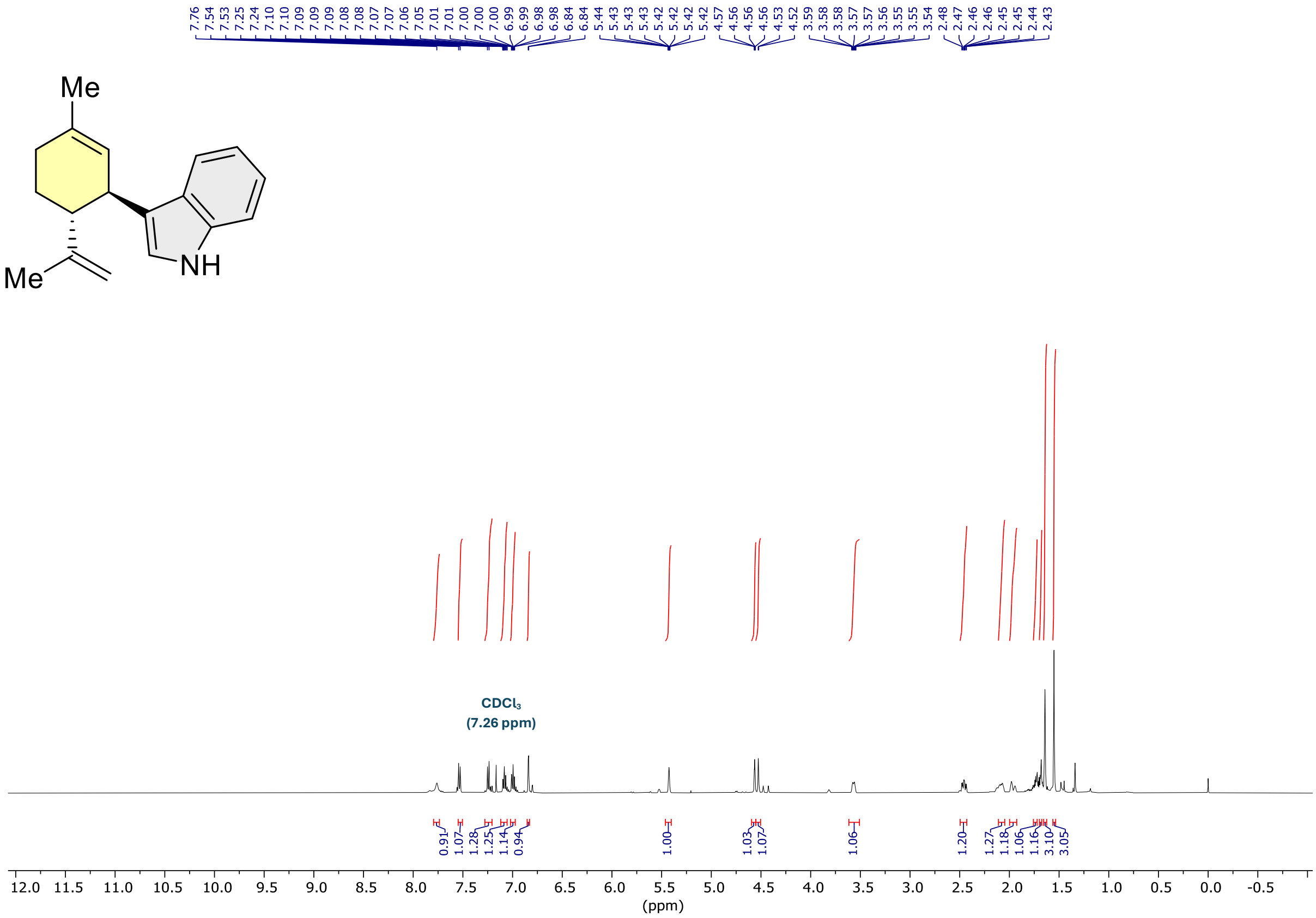

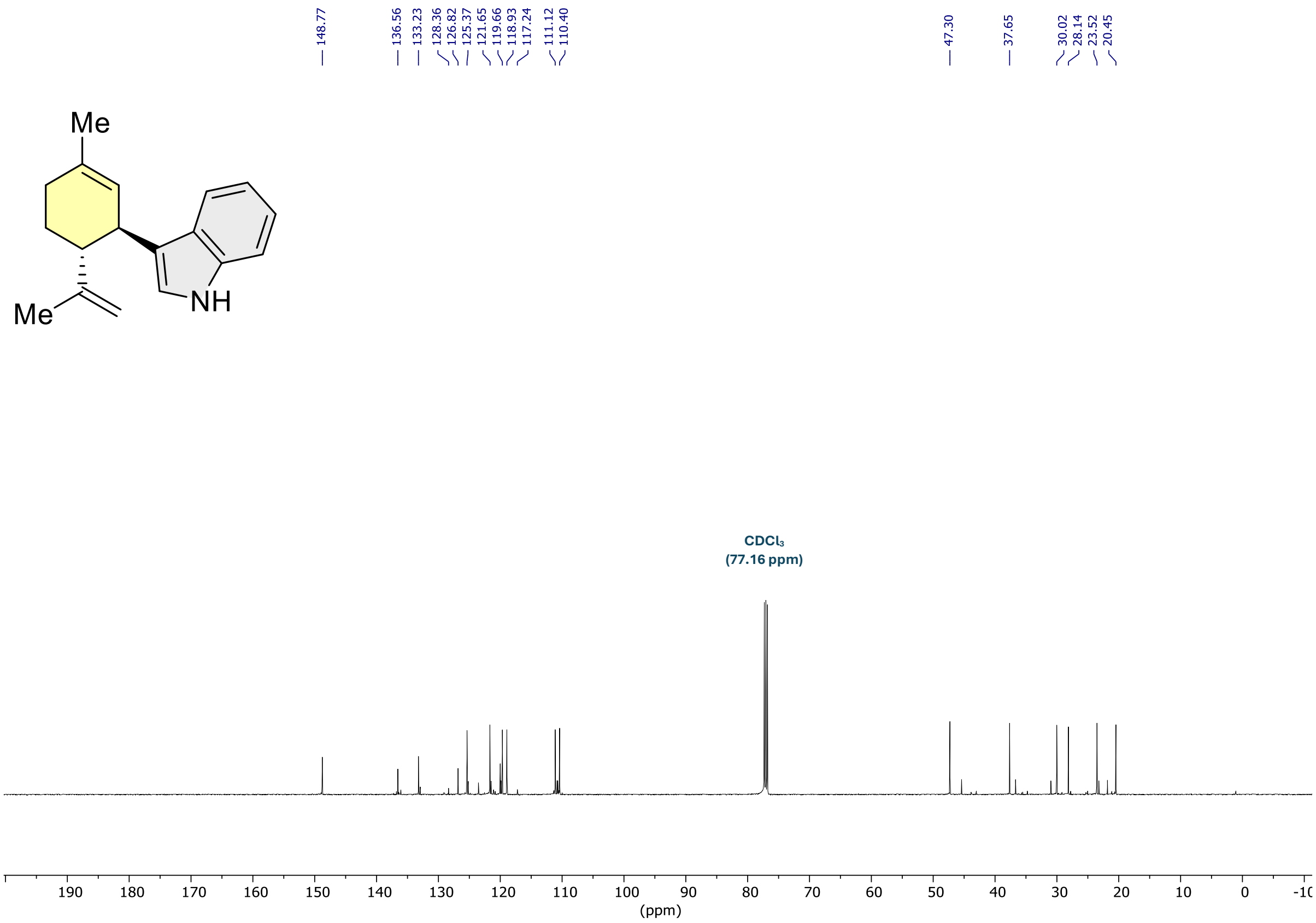

Compound 50. ^{1}HNMR (500 MHz, $CDCl_3$) and $^{13}C\{^{1}H\}$ NMR (125 MHz, $CDCl_3$).

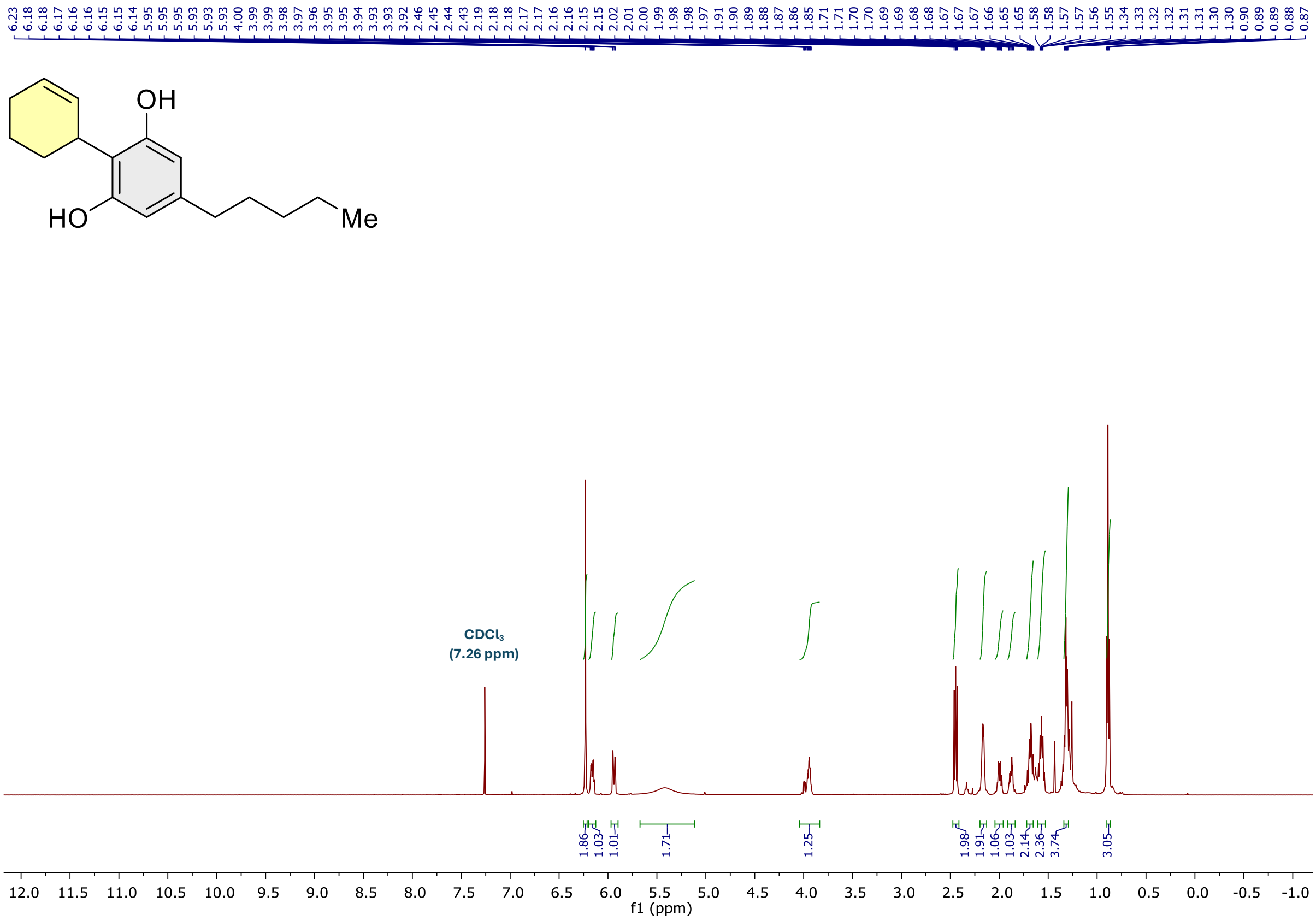

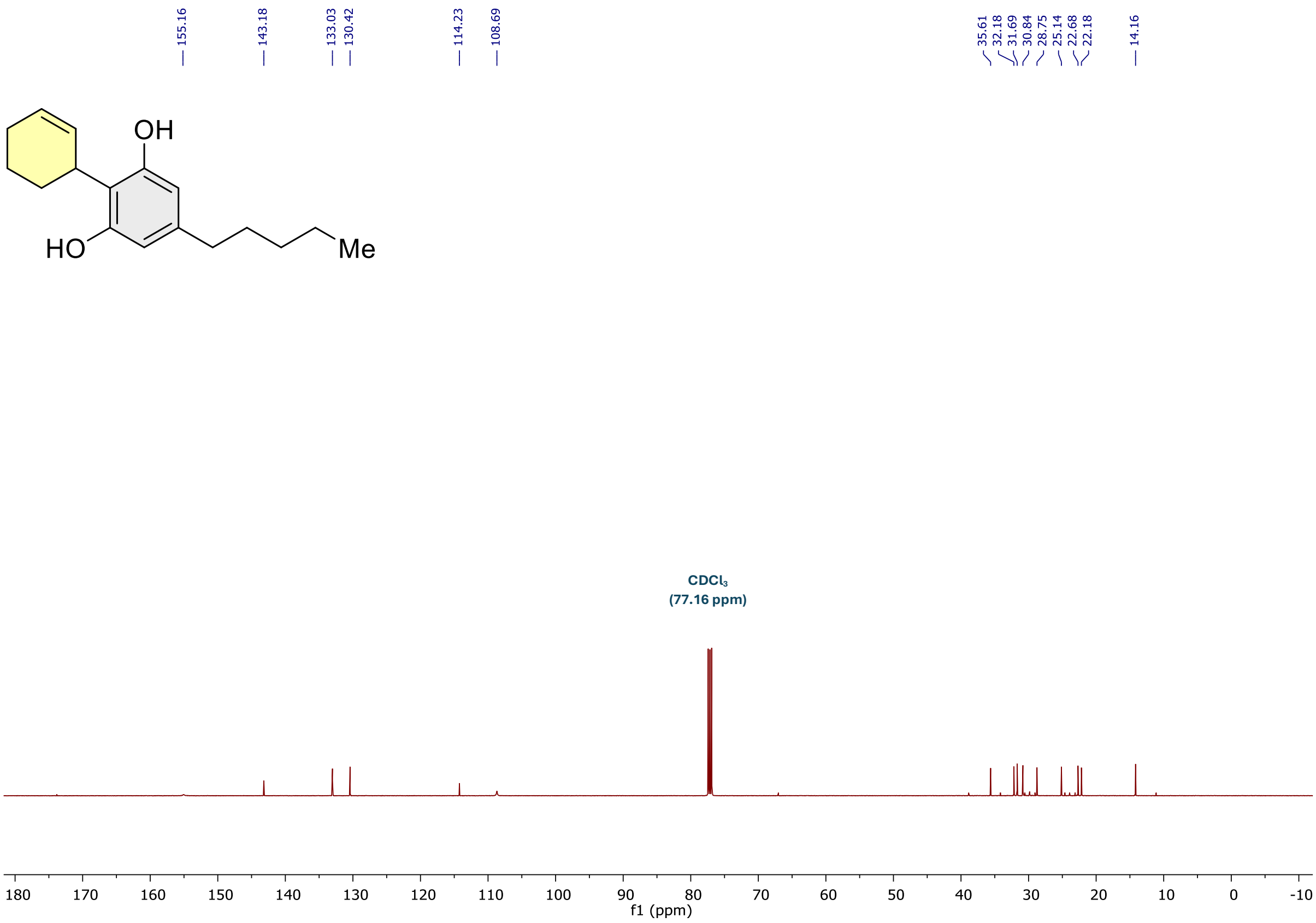

Compound 51 and **52.** ^{1}HNMR (500 MHz, $CDCl_3$) and $^{13}C\{^1H\}$ NMR (125 MHz, $CDCl_3$).

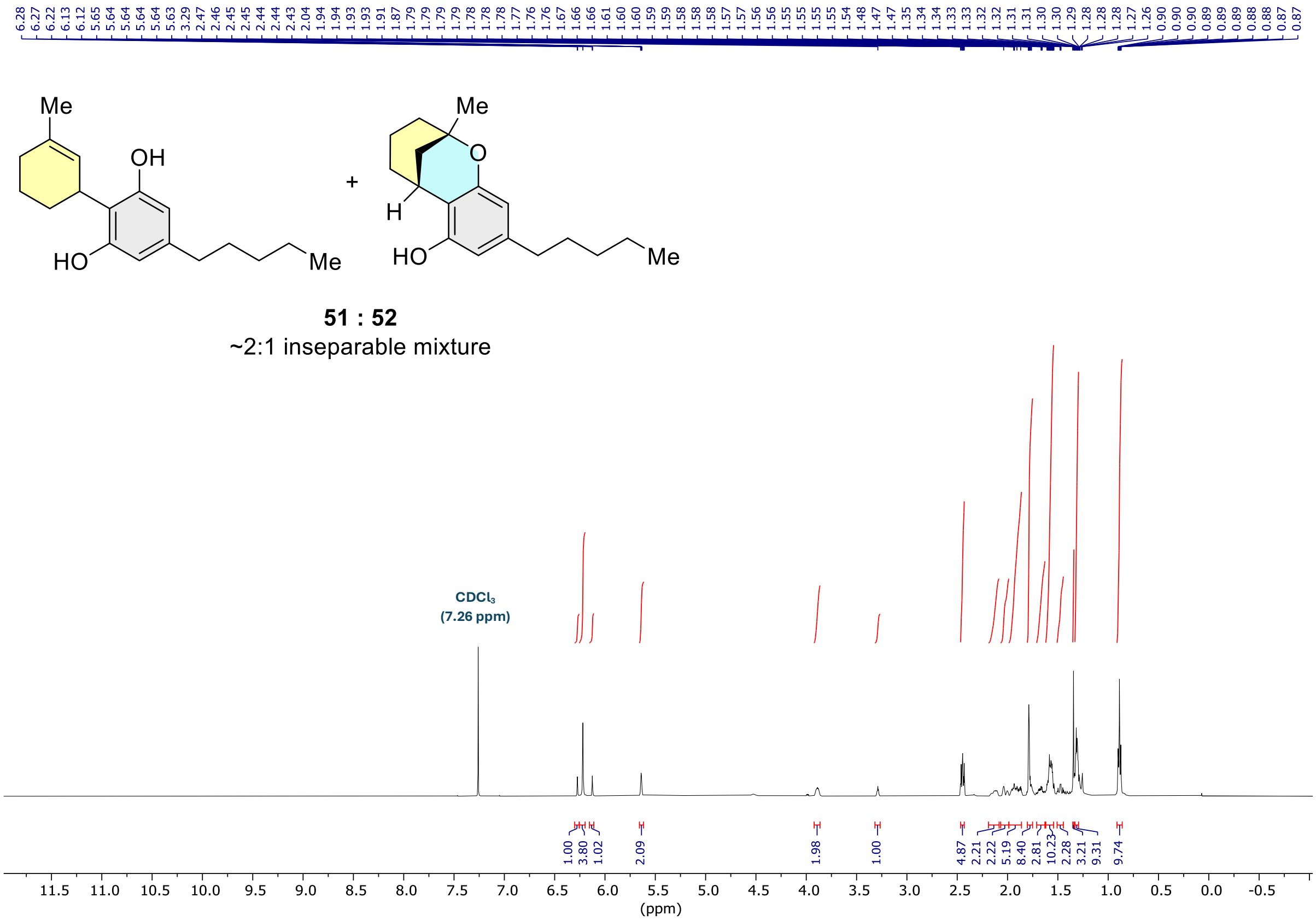

51 : 52
~2:1 inseparable mixture

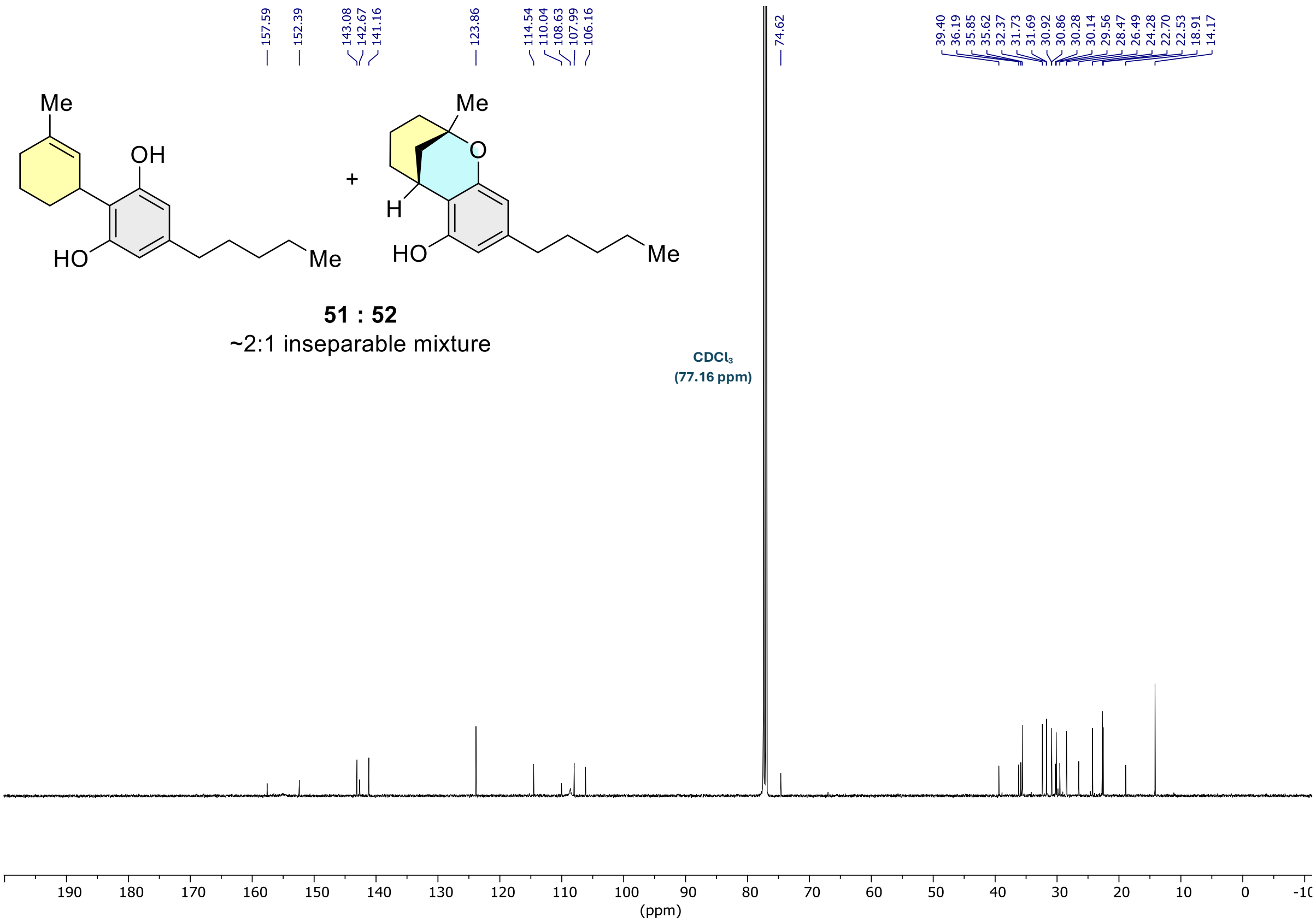

51 : 52
~2:1 inseparable mixture

Compound 53 and Δ8-THC (8). ^{1}HNMR (500 MHz, $CDCl_3$) and $^{13}C\{^{1}H\}$ NMR (125 MHz, $CDCl_3$).

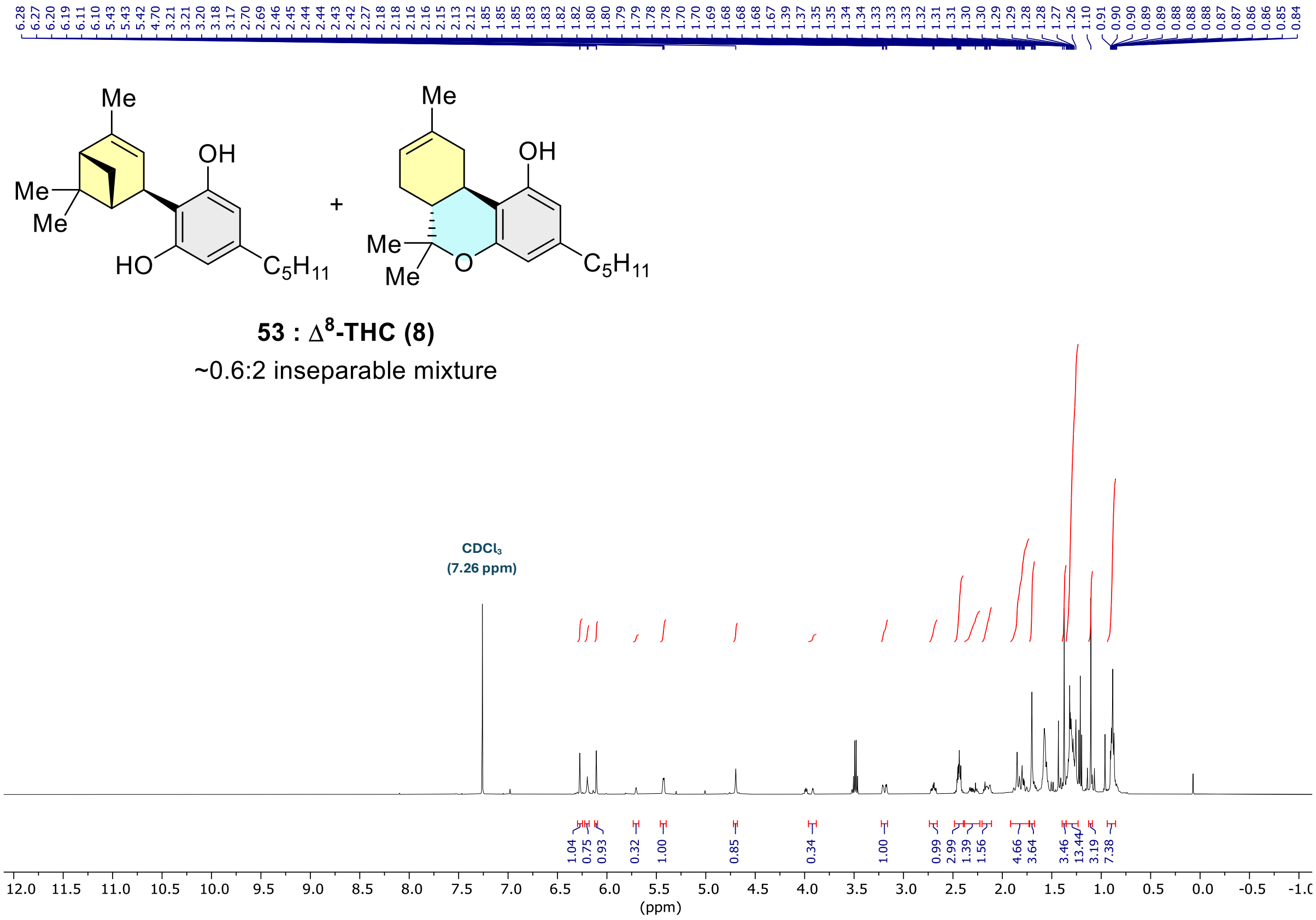

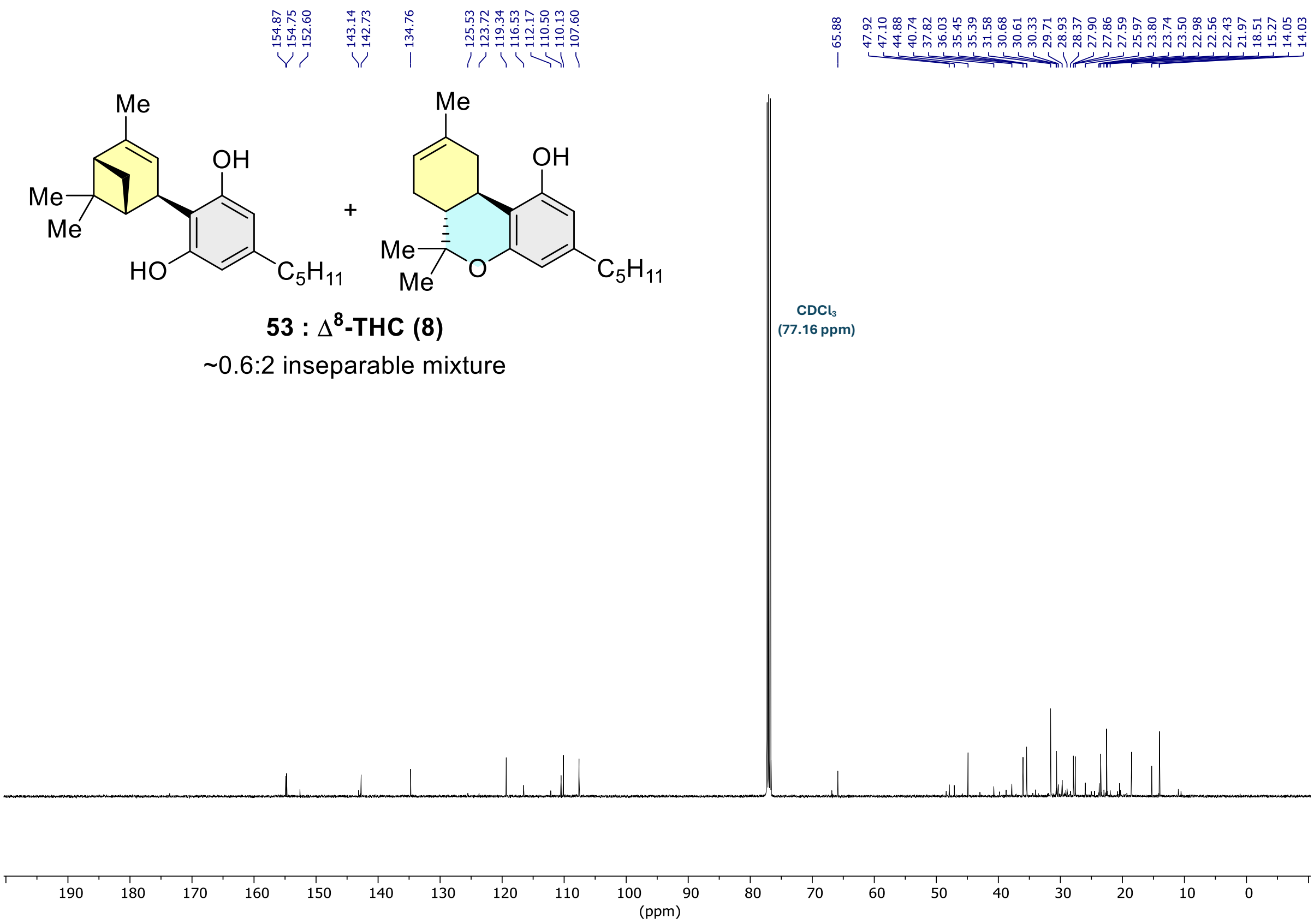

Compound 54 and **1'1-DMH-Δ⁸-THC (55).** ^{1}HNMR (500 MHz, $CDCl_3$) and $^{13}C\{^{1}H\}$ NMR (125 MHz, $CDCl_3$).

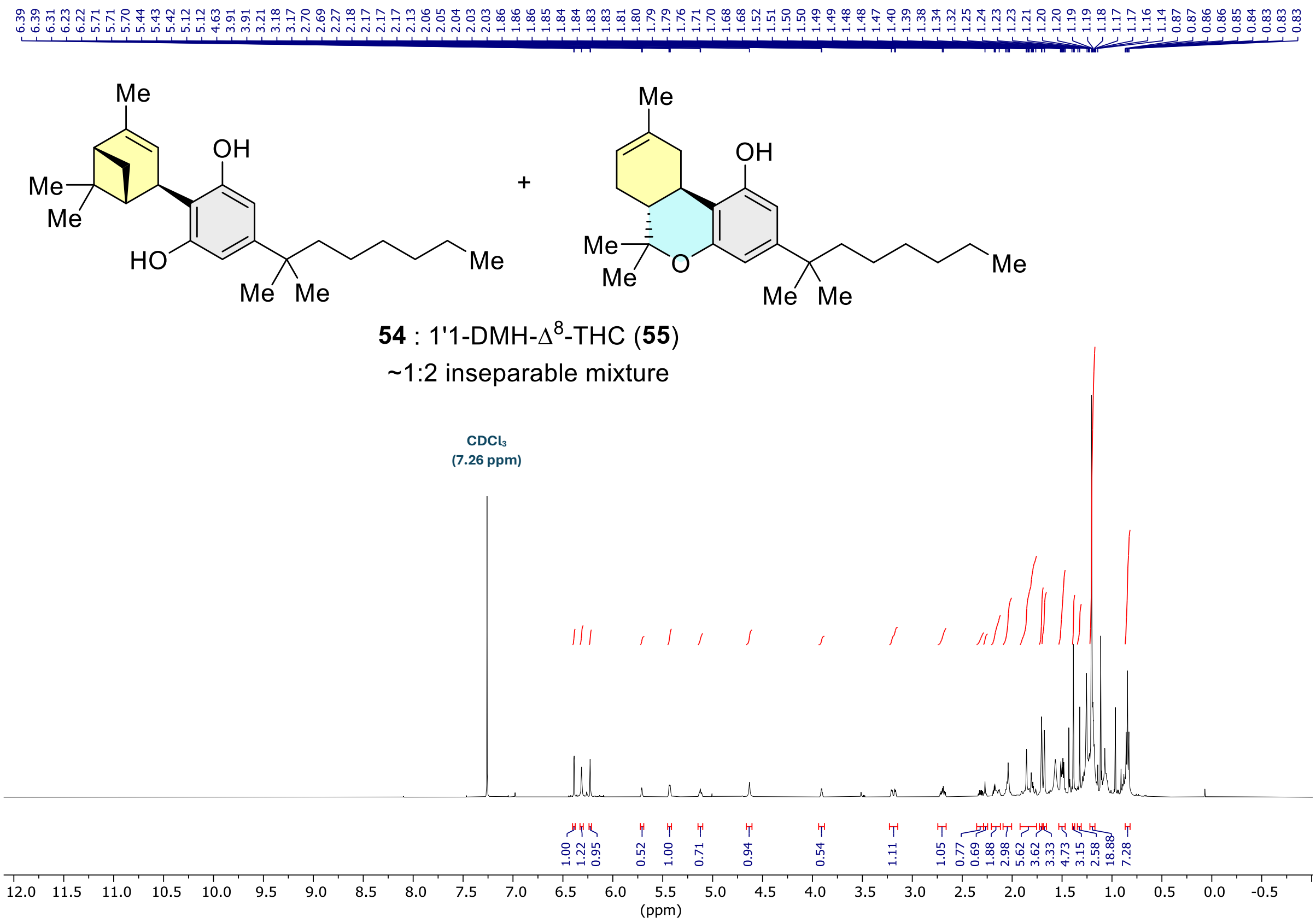

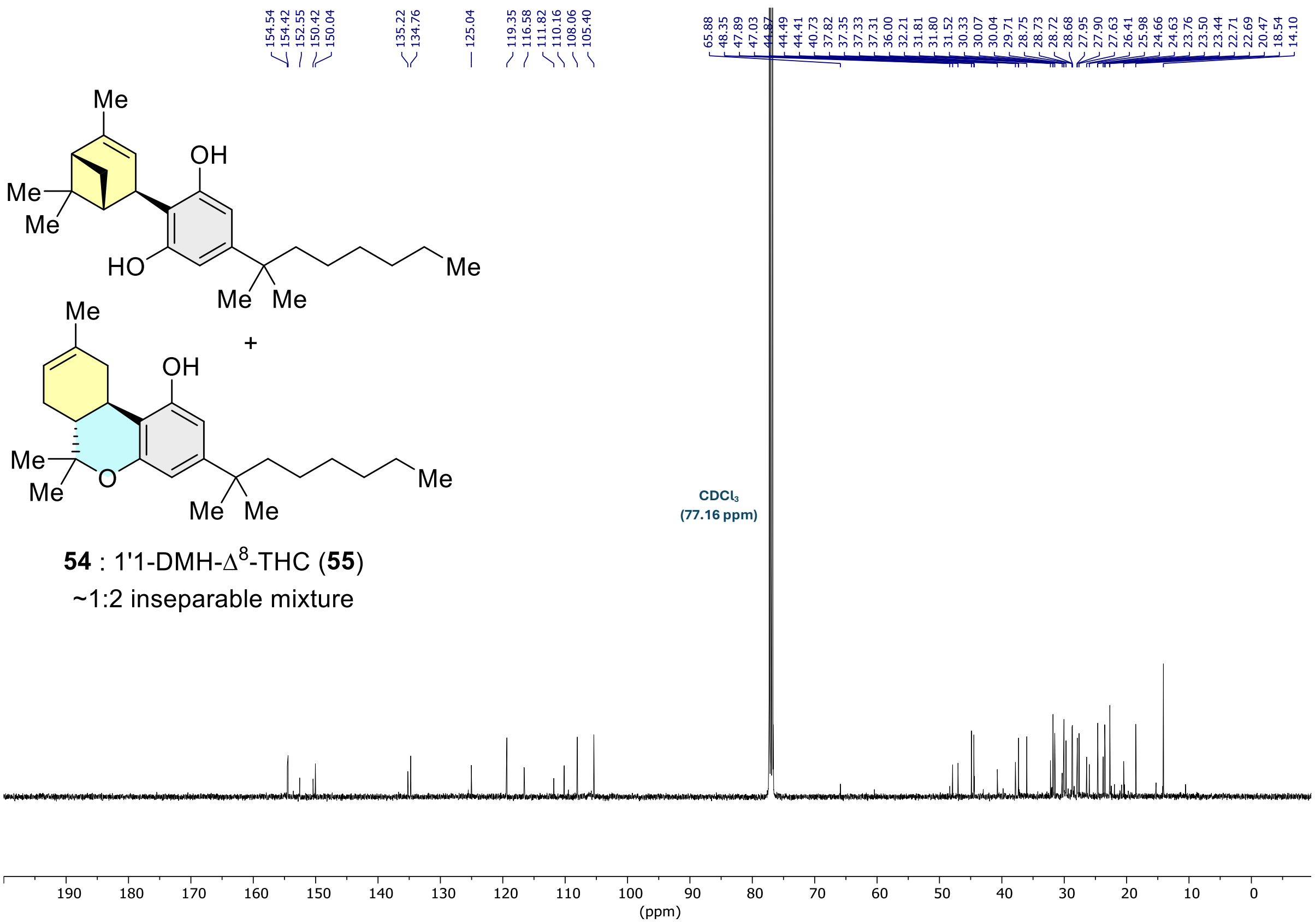

Compound 55 (1'1-DMH-Δ⁸-THC). ¹HNMR (500 MHz, $CDCl_3$) and $^{13}C\{^1H\}$ NMR (125 MHz, $CDCl_3$).

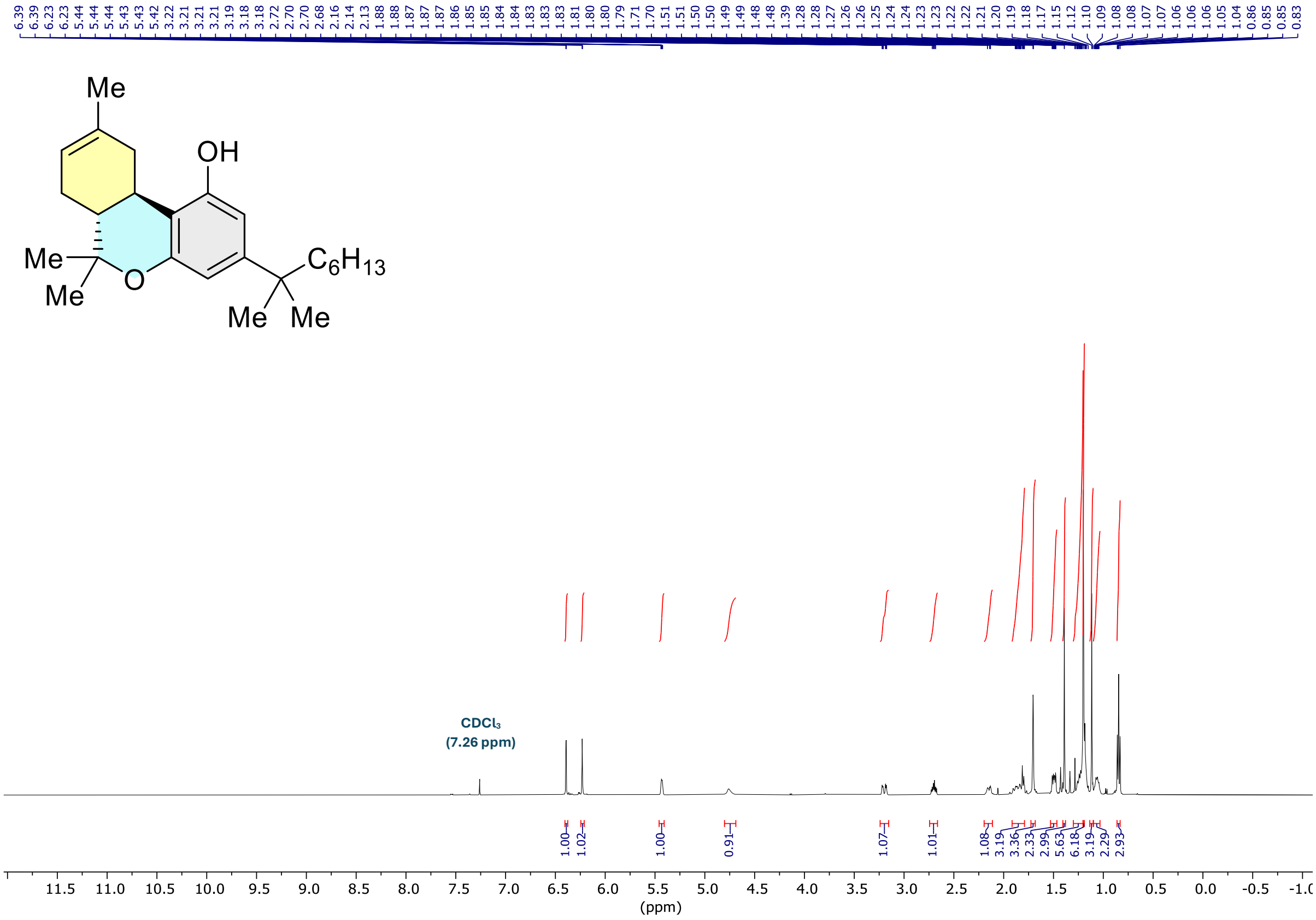

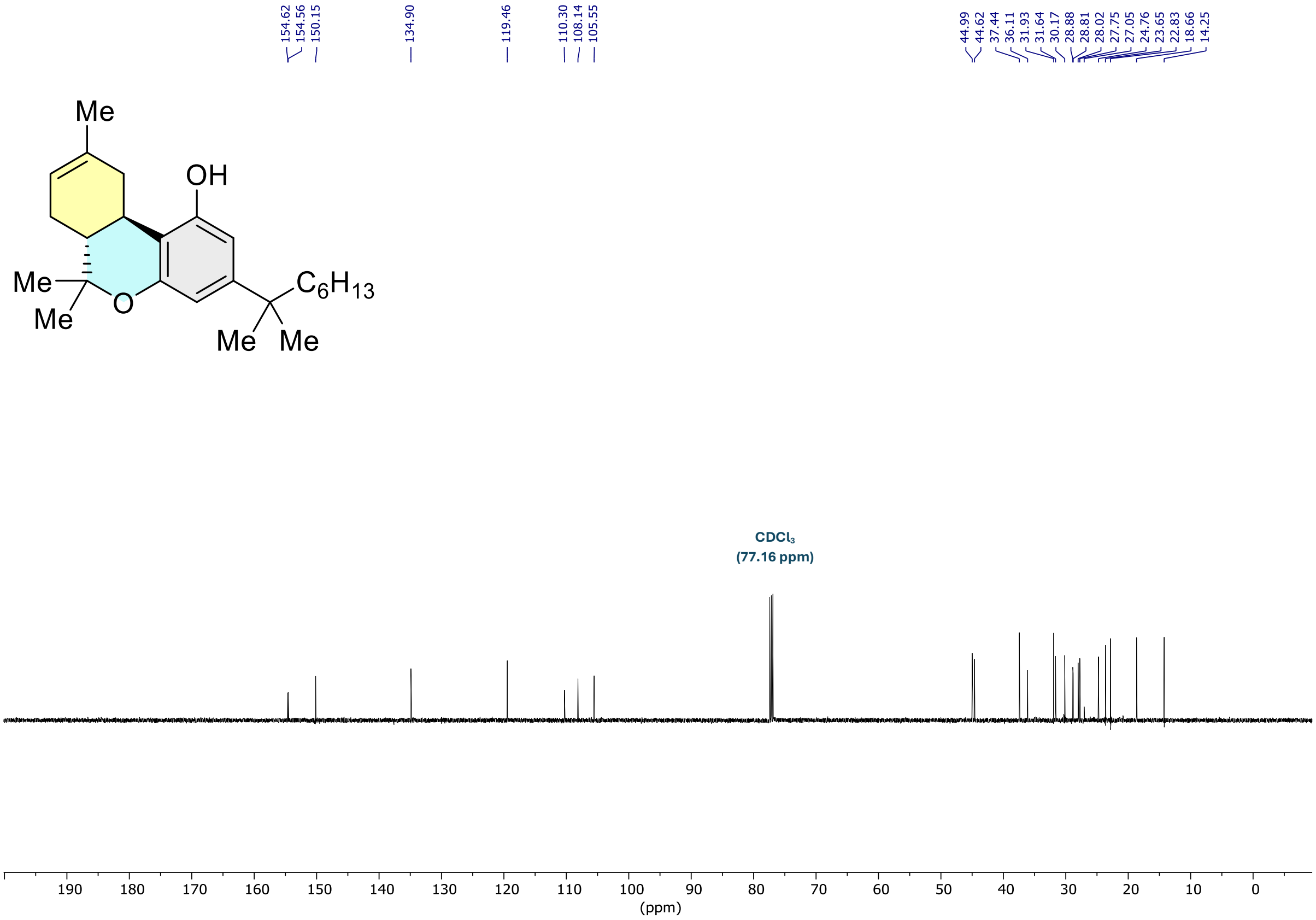

Compound 57 (1'1-DMH-Δ⁹-THC). ^{1}HNMR (400 MHz, $CDCl_3$) and $^{13}C\{^{1}H\}$ NMR (100 MHz, $CDCl_3$).

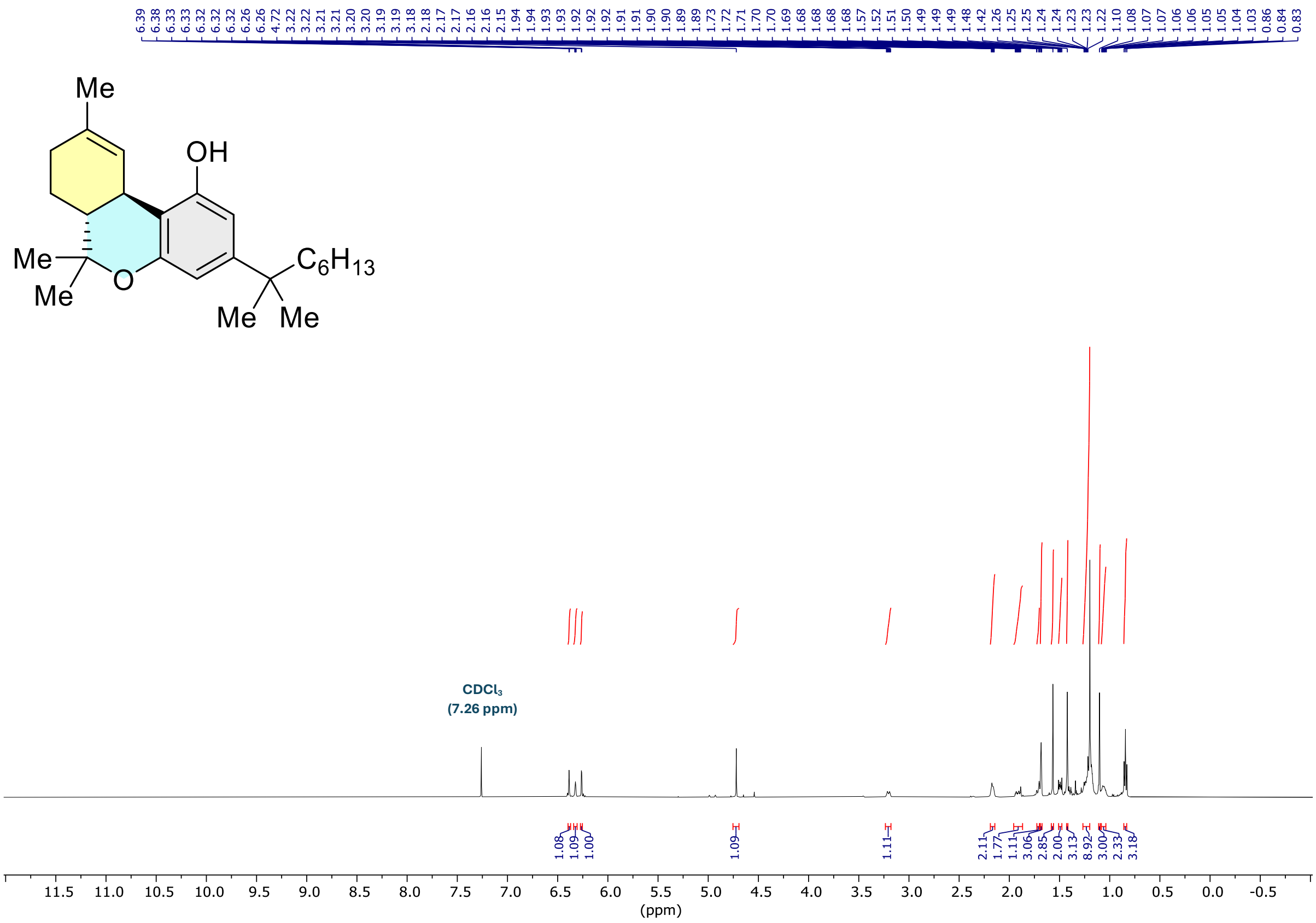

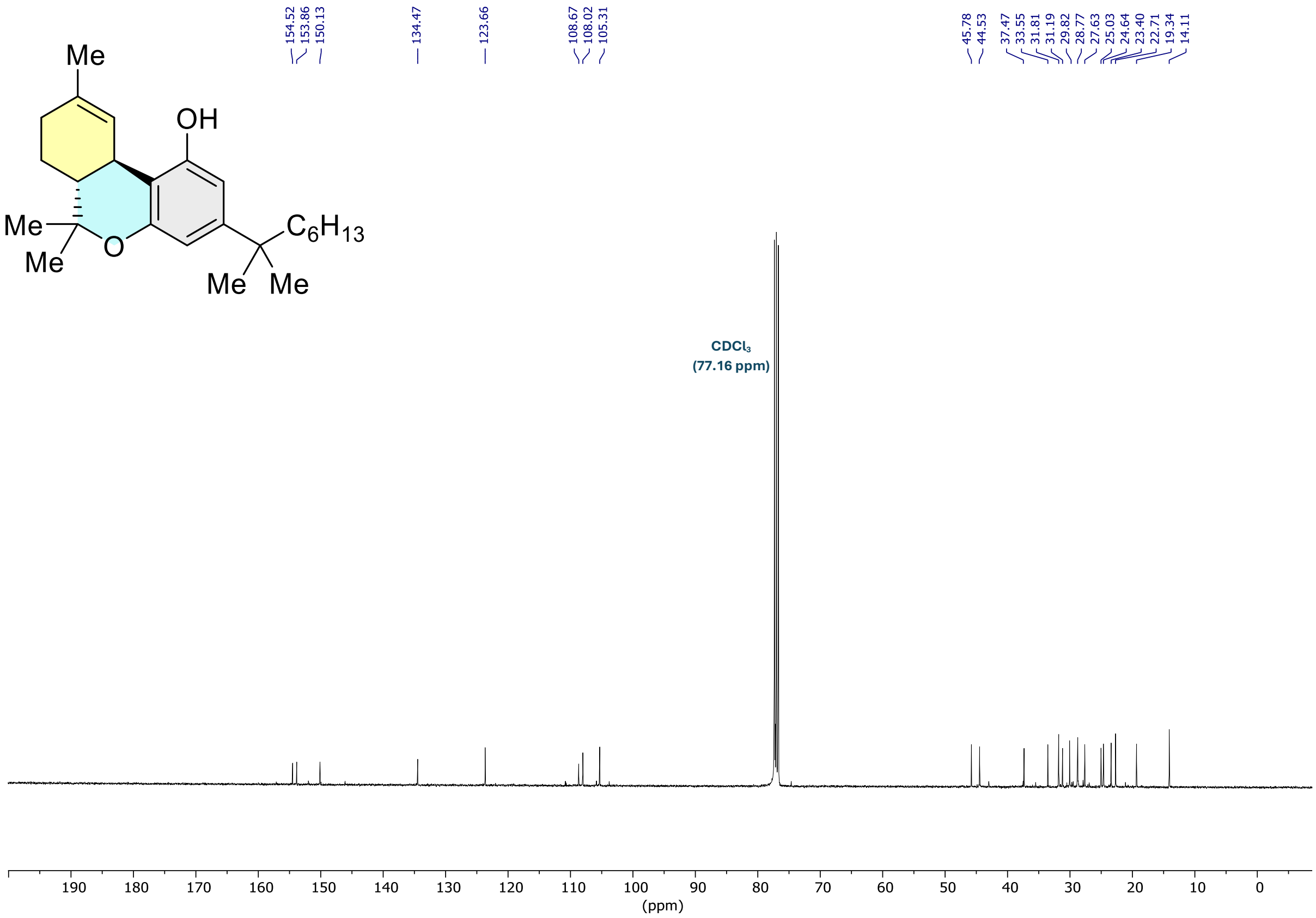

9. References

[1] Moya-Utrera, F.; Fuentes-Ríos, D.; Romero-Carrasco, A.; Doña-Flores, M.; Cheng-Sánchez, I.; Díaz-Morilla, A.; Soledad Pino-González, M.; Martínez-Ferez, A.; Moreno, J.; Mesas, C.; Melguizo, C.; Prados, J.; Sarabia, F.; López-Romero, J. M. Synthesis of (−)-Cannabidiol (CBD), (−)-Δ^9- and (−)-Δ^8-Tetrahydrocannabinols, Encapsulation of CBD with Nanoparticles for Controlled Delivery and Biological Evaluation *Chem. Eur. J.* **2024**, *30*, e202402496. DOI: 10.1002/chem.202402496

[2] Quílez del Moral, J. F.; Martínez, C. R.; Pérez del Pulgar, E.; Martín González, J. E.; Fernández, I.; López-Pérez, J. L.; Fernández-Arteaga, A.; Barrero, A. F. Synthesis of Cannabinoids: "In Water" and "On Water" Approaches: Influence of SDS Micelles. *J. Org. Chem.* **2021**, *86*, 3344–3355. DOI: 10.1021/acs.joc.0c02698

[3] (a) Cheng, Y.-C.; Kerrigan, S. Factors influencing the in situ formation of Δ^9-THC from cannabidiol during GC–MS analysis. *Drug Test Anal.* **2024**, *16*, 989-1001. DOI: 10.1002/dta.3617. (b) Tsujikawa, K.; Okada, Y.; Segawa, H.; Yamamuro, T.; Kuwayama, K.; Kanamori, T.; Iwata, Y. T. Thermal decomposition of CBD to Δ^9-THC during GC-MS analysis: A potential cause of Δ9-THC misidentification. *Forensic Sci. Int.* **2022**, *337*, 111366. DOI: 10.1016/j.forsciint.2022.111366

[4] Papan, P.; Kantapan, J.; Sangthong, P.; Meepowpan, P.; Dechsupa, N. Iron (III)-Quercetin Complex: Synthesis, Physicochemical Characterization, and MRI Cell Tracking toward Potential Applications in Regenerative Medicine. *Contrast Media Mol. Imaging* **2020**, 8877862. DOI: 10.1155/2020/8877862

[5] Neese, F. The ORCA quantum chemistry program package. *WIREs Comput. Mol. Sci.* **2012**, *2*, 73-78. DOI:10.1002/wcms.81

[6] Neese, F. Software update: The ORCA program system—Version 6.0. *WIREs Comput. Mol. Sci.* **2025**, *15*, e70019. DOI: 10.1002/wcms.70019

Caldeweyher, E.; Ehlert, S.; Hansen, A.; Neugebauer, H.; Spicher, S.; Bannwarth, C.; Grimme, S. A generally applicable atomic-charge dependent London dispersion correction. *J. Chem. Phys.* **2019**, *150*, 154122. DOI: 10.1063/1.5090222

Harding, L. B.; Georgievskii, Y.; Klippenstein, S. J. Roaming Radical Kinetics in the Decomposition of Acetaldehyde. *J. Phys. Chem. A* **2010**, *114*, 765–777. DOI: 10.1021/jp906919w

Grimme, S. Supramolecular Binding Thermodynamics by Dispersion-Corrected Density Functional Theory. *Chem. Eur. J.* **2012**, *18*, 9955–9964. DOI:10.1002/chem.201200497

[10] Ando, H.; Nakao, Y.; Sato, H.; Sakaki, S. Theoretical study of low-spin, high-spin, and intermediate-spin states of [FeIII(pap)2]+(pap = N-2-pyridylmethylidene-2-hydroxy-phenylaminato). Mechanism of light-induced excited spin state trapping. *J. Phys. Chem. A* **2007**, *111*, 5515–5522. DOI: 10.1021/jp0714163

[11] Swart, M.; Gruden, M. Spinning around in Transition-Metal Chemistry. *Acc. Chem. Res.* **2016**, *49*, 2690–2697. DOI: 10.1021/acs.accounts.6b00271

[12] Neese, F. Importance of direct spin−spin coupling and spin-flip excitations for the zero-field splittings of transition metal complexes: A case study. *J. Am. Chem. Soc.* **2006**, *128*, 10213–10222. DOI: 10.1021/ja061798a

[13] Neese, F. Definition of corresponding orbitals and the diradical character in broken symmetry DFT calculations on spin coupled systems. *J.Phys. Chem. Solids* **2004**, *65*, 781–785. DOI: 10.1016/j.jpcs.2003.11.015

[14] Safo, M. K.; Walker, F. A.; Raitsimring, A. M.; Walters, W. P.; Dolata, D. P.; Debrunner, P. G.; Scheidt, W. R. Axial Ligand Orientation in Iron(III) Porphyrinates: Effect of Axial π-Acceptors. Characterization of the Low-Spin Complex [Fe(TPP)(4-CNPy)2]ClO4. *J. Am. Chem. Soc.* **1994**, *116*, 7760–7770. DOI: 10.1021/ja00096a037

Esteves, P. M.; Carneiro, J. W. M.; Cardoso, S. P.; Bashford, D.; Nogueira, G. C. A Unified Continuum of Electrophilic Aromatic Substitution. *J. Am. Chem. Soc.* **2003**, *125*, 4836–4849. DOI: 10.1021/ja021307w

Bickelhaupt, F. M.; Houk, K. N. Analyzing Reaction Rates with the Distortion/Interaction-Activation Strain Model. *Angew. Chem. Int. Ed.* **2017**, *56*, 10070–10086. DOI: 10.1002/anie.201701486Digital Object Identifier (DOI)

Bowman, J. M.; Shepler, B. C. Roaming Radicals. *Annu. Rev. Phys. Chem.* **2011**, *62*, 531–553. DOI: 10.1146/annurev-physchem-032210-103518

Craescu, C. V.; David, C. D.; Heafner, E. D.; Raymond, K. N.; Bergman, R. G.; Toste, F. D. Attractive Noncovalent Interactions versus Steric Confinement in Asymmetric Supramolecular Catalysis. *J. Am. Chem. Soc.* **2026**, *148*, 1306–1315. DOI: 10.1021/jacs.5c17872

Davis, H. J.; Phipps, R. J. Harnessing Noncovalent Interactions to Exert Control Over Regioselectivity and Site-Selectivity in Transition Metal Catalysis. *Chem. Sci.* **2017**, *8*, 864–877. DOI: 10.1039/C6SC04157D

Zhu, K.; Achord, P. D.; Zhang, X.; Krogh-Jespersen, K.; Goldman, A. S. Highly Effective Pincer-Ligated Iridium Catalysts for Alkane Dehydrogenation. *J. Am. Chem. Soc.* **2004**, *126*, 13044–13053. DOI: 10.1021/ja047356l

Wheeler, S. E.; Houk, K. N.; Schleyer, P. v. R.; Allen, W. D. A Hierarchy of Homodesmotic Reactions for Thermochemistry. *J. Am. Chem. Soc.* **2009**, *131*, 2547–2560. DOI: 10.1021/ja805843n

[22] Waugh, T. M.; Masters, J.; Aliev, A. E.; Marson, C. M. Monocyclic Quinone Structure-Activity Patterns: Synthesisof Catalytic Inhibitors of Topoisomerase II with PotentAntiproliferative Activity. *ChemMedChem* **2020**, *15*, 114–124. DOI: 10.1002/cmdc.201900548

[23] Kang, S.; Davidsen, K.; Gomez-Castillo, L.; Jiang, H.; Fu, X.; Li, Z.; Liang, Y.; Jahn, M-; Moussa, M.; DiMaio, F.; Gu, L. COMBINES-CID: An Efficient Method for De Novo Engineering of Highly Specific Chemically Induced Protein Dimerization Systems. *J. Am. Chem. Soc.* **2019**, *141*, 10948–10952. DOI: 10.1021/jacs.9b03522

[24] Yukhet, P.; Faikhruea, K.; Mowang, R.; Naweephattana, P.; Ratanasak, M.; Shigeta, Y.; Rungrotmongkol, T.; Aonbangkhen, C.; Vilaivan, T. A Mild and Catalyst-Free Synthesis of Cannabidiols and Regioisomers Mediated by Fluorinated Alcohols. *Chem. Asian J.* **2025**, e202401648. DOI: 10.1002/asia.202401648
